# World Health Organization cardiovascular disease risk charts: revised models to estimate risk in 21 global regions

**DOI:** 10.1016/S2214-109X(19)30318-3

**Published:** 2019-09-02

**Authors:** The WHO CVD Risk Chart Working Group, Stephen Kaptoge, Lisa Pennells, Dirk De Bacquer, Marie Therese Cooney, Maryam Kavousi, Gretchen Stevens, Leanne Margaret Riley, Stefan Savin, Taskeen Khan, Servet Altay, Philippe Amouyel, Gerd Assmann, Steven Bell, Yoav Ben-Shlomo, Lisa Berkman, Joline W Beulens, Cecilia Björkelund, Michael Blaha, Dan G Blazer, Thomas Bolton, Ruth Bonita Beaglehole, Hermann Brenner, Eric J Brunner, Edoardo Casiglia, Parinya Chamnan, Yeun-Hyang Choi, Rajiv Chowdry, Sean Coady, Carlos J Crespo, Mary Cushman, Gilles R Dagenais, Ralph B D'Agostino Sr, Makoto Daimon, Karina W Davidson, Gunnar Engström, Ian Ford, John Gallacher, Ron T Gansevoort, Thomas Andrew Gaziano, Simona Giampaoli, Greg Grandits, Sameline Grimsgaard, Diederick E Grobbee, Vilmundur Gudnason, Qi Guo, Hanna Tolonen, Steve Humphries, Hiroyasu Iso, J Wouter Jukema, Jussi Kauhanen, Andre Pascal Kengne, Davood Khalili, Wolfgang Koenig, Daan Kromhout, Harlan Krumholz, TH Lam, Gail Laughlin, Alejandro Marín Ibañez, Tom W Meade, Karel G M Moons, Paul J Nietert, Toshiharu Ninomiya, Børge G Nordestgaard, Christopher O'Donnell, Luigi Palmieri, Anushka Patel, Pablo Perel, Jackie F Price, Rui Providencia, Paul M Ridker, Beatriz Rodriguez, Annika Rosengren, Ronan Roussel, Masaru Sakurai, Veikko Salomaa, Shinichi Sato, Ben Schöttker, Nawar Shara, Jonathan E Shaw, Hee-Choon Shin, Leon A Simons, Eleni Sofianopoulou, Johan Sundström, Henry Völzke, Robert B Wallace, Nicholas J Wareham, Peter Willeit, David Wood, Angela Wood, Dong Zhao, Mark Woodward, Goodarz Danaei, Gregory Roth, Shanthi Mendis, Oyere Onuma, Cherian Varghese, Majid Ezzati, Ian Graham, Rod Jackson, John Danesh, Emanuele Di Angelantonio

## Abstract

**Background:**

To help adapt cardiovascular disease risk prediction approaches to low-income and middle-income countries, WHO has convened an effort to develop, evaluate, and illustrate revised risk models. Here, we report the derivation, validation, and illustration of the revised WHO cardiovascular disease risk prediction charts that have been adapted to the circumstances of 21 global regions.

**Methods:**

In this model revision initiative, we derived 10-year risk prediction models for fatal and non-fatal cardiovascular disease (ie, myocardial infarction and stroke) using individual participant data from the Emerging Risk Factors Collaboration. Models included information on age, smoking status, systolic blood pressure, history of diabetes, and total cholesterol. For derivation, we included participants aged 40–80 years without a known baseline history of cardiovascular disease, who were followed up until the first myocardial infarction, fatal coronary heart disease, or stroke event. We recalibrated models using age-specific and sex-specific incidences and risk factor values available from 21 global regions. For external validation, we analysed individual participant data from studies distinct from those used in model derivation. We illustrated models by analysing data on a further 123 743 individuals from surveys in 79 countries collected with the WHO STEPwise Approach to Surveillance.

**Findings:**

Our risk model derivation involved 376 177 individuals from 85 cohorts, and 19 333 incident cardiovascular events recorded during 10 years of follow-up. The derived risk prediction models discriminated well in external validation cohorts (19 cohorts, 1 096 061 individuals, 25 950 cardiovascular disease events), with Harrell's C indices ranging from 0·685 (95% CI 0·629–0·741) to 0·833 (0·783–0·882). For a given risk factor profile, we found substantial variation across global regions in the estimated 10-year predicted risk. For example, estimated cardiovascular disease risk for a 60-year-old male smoker without diabetes and with systolic blood pressure of 140 mm Hg and total cholesterol of 5 mmol/L ranged from 11% in Andean Latin America to 30% in central Asia. When applied to data from 79 countries (mostly low-income and middle-income countries), the proportion of individuals aged 40–64 years estimated to be at greater than 20% risk ranged from less than 1% in Uganda to more than 16% in Egypt.

**Interpretation:**

We have derived, calibrated, and validated new WHO risk prediction models to estimate cardiovascular disease risk in 21 Global Burden of Disease regions. The widespread use of these models could enhance the accuracy, practicability, and sustainability of efforts to reduce the burden of cardiovascular disease worldwide.

**Funding:**

World Health Organization, British Heart Foundation (BHF), BHF Cambridge Centre for Research Excellence, UK Medical Research Council, and National Institute for Health Research.

## Introduction

By the year 2030, the UN Sustainable Development Goals^1^ aim to reduce premature mortality from non-communicable diseases by a third. Cardiovascular diseases (which include coronary heart disease and stroke) are the most common non-communicable diseases globally, responsible for an estimated 17·8 million deaths in 2017, of which more than three quarters were in low-income and middle-income countries.^2^ To help reduce the global burden of cardiovascular disease, WHO member states have committed to provide counselling and drug treatments for at least 50% of eligible people (defined as aged 40 years or older and at high risk of cardiovascular disease) by 2025.^3^ To support such expansion of cardiovascular disease prevention and control efforts, WHO has developed tools and guidance, including risk prediction charts.^4^, ^5^

Risk prediction models can be a component of cardiovascular disease prevention and control efforts, because they can help to identify people at high risk of cardiovascular disease who should benefit the most from preventive interventions.^6^, ^7^ Many such risk prediction models have been developed,^8^, ^9^, ^10^, ^11^, ^12^, ^13^ usually estimating individual risk over a 10-year period by use of measured levels of conventional risk factors for cardiovascular disease.^14^ However, available models have limitations for use in low-income and middle-income countries. Most models were derived and validated with use of a narrow set of studies, might be directly applicable only to specific populations (mainly in high-income countries), and might not predict the correct risk in the target population being screened (ie, poor calibration).^8^, ^13^, ^15^, ^16^, ^17^, ^18^

**Research in context**

**Evidence before this study**

To update the 2007 WHO and International Society of Hypertension's cardiovascular disease risk prediction approaches, WHO has convened an informal risk-chart working group. To inform this work, we searched PubMed, Scientific Citation Index Expanded, and Embase to identify existing risk prediction models for cardiovascular disease in the context of primary prevention published in any language up to May 15, 2019, using the relevant terms: “cardiovascular disease”, “risk score”, “risk equation”, “risk algorithm”, and “risk prediction”. We found many studies and reviews describing risk prediction models to estimate cardiovascular disease risk in a primary prevention context. However, none had combined the following key features necessary to develop reliable risk models relevant to low-income and middle-income countries: use of powerful and diverse global data, simple and generalisable methods to account for differences in populations (ie, to allow recalibration), and inclusion of information that is readily available in many low-income and middle-income countries.

**Added value of this study**

The newly developed risk models involve several features that should confer advantages compared with existing tools. First, they are underpinned by powerful, extensive, and complementary datasets of global relevance. Second, we used comprehensive contemporary estimates of cardiovascular disease incidence and risk factor values to adapt (ie, recalibrate) the risk models to many different populations using a simpler and more generalisable approach than that of previous studies. Third, these models provide estimates for the combined outcome of fatal and non-fatal events. Fourth, they include pragmatic models that do not assume availability of laboratory measurements (eg, serum lipid concentrations) that could be used as part of stepwise approaches to help target laboratory testing in people most likely to benefit from the extra information.

**Implications of all the available evidence**

We have derived, validated, and illustrated new WHO models for cardiovascular disease risk prediction adapted for the needs of low-income and middle-income countries, to support tools and guidance for cardiovascular disease prevention and control. The widespread use of these models could enhance the accuracy, practicability, and sustainability of efforts to reduce the burden of cardiovascular disease worldwide.

Here, we provide derivation, validation, and illustration of updated WHO models for cardiovascular disease risk prediction. To enhance targeting of efforts to reduce the burden of cardiovascular disease, we have statistically adapted (ie, recalibrated)^14^, ^19^ models to the contemporary circumstances of many different global regions using routinely available information. The aim of recalibration was to ensure that risk prediction models estimate risk for individuals in each region more accurately. To help make this approach more sustainable, we developed and describe here a method that can be used to regularly update risk prediction models using information about epidemiological trends in cardiovascular disease within different global regions. The WHO CVD Risk Chart Working Group, a cross-sectoral collaboration of academics, policy makers, and end users of risk scores, was convened to facilitate this development of revised models for prediction of cardiovascular disease risk more tailored to the needs of low-income and middle-income countries.

## Methods

### Study design

**Figure 1:**
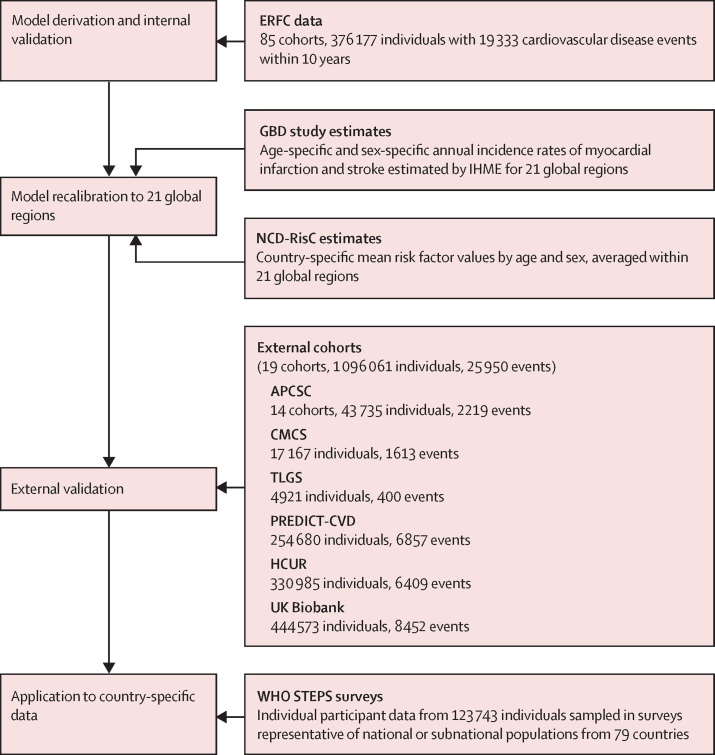
Study design ERFC=Emerging Risk Factors Collaboration. GBD=Global Burden of Disease. IHME=Institute for Health Metrics and Evaluation. NCD-RisC=Non-Communicable Diseases Risk Factor Collaboration. APCSC=Asia Pacific Cohort Studies Collaboration. CMCS=Chinese Multi-Provincial Cohort Study. TLGS=Tehran Lipids and Glucose Study. PREDICT-CVD=New Zealand primary care-based PREDICT-CVD cohort. HCUR=Health Checks Ubon Ratchathani Study in Thailand. WHO STEPS=WHO STEPwise Approach to Surveillance.

In our model revision initiative, several interrelated components were involved (figure 1). First, we derived risk prediction models using individual participant data from 85 prospective cohorts in the Emerging Risk Factors Collaboration (ERFC). Second, we adjusted models to the contemporary circumstances of multiple global regions, recalibrating models using age-specific and sex-specific incidences and risk factor values obtained from the Global Burden of Disease (GBD) studies^20^, ^21^ and the Non-Communicable Disease Risk Factor Collaboration (NCD-RisC).^22^, ^23^, ^24^ Third, we completed external validation using individual participant data from a further 19 prospective cohorts that did not contribute to the model derivation. Fourth, models were applied to individual participant data from 79 countries collected with the WHO STEPwise Approach to Surveillance (STEPS).^25^ Fifth, we used this sequence of analyses to assess the potential value of pragmatic risk models (eg, those that include information on body-mass index [BMI] instead of serum lipid values), because laboratory measurements are not widely available in many low-income and middle-income countries.^9^, ^15^, ^26^

### Data sources and procedures

The ERFC was selected for model derivation because it has collated and harmonised individual participant data from many long-term prospective cohort studies of cardiovascular disease risk factors and outcomes.^27^, ^28^ Prospective studies in the ERFC were included in our analysis if they met all the following criteria: had recorded baseline information on risk factors necessary to derive risk prediction models (ie, age, sex, smoking status [current *vs* other], history of diabetes, systolic blood pressure, and total cholesterol or BMI), were approximately population-based (ie, did not select participants on the basis of having previous disease), had recorded cause-specific deaths and non-fatal cardiovascular disease events (ie, non-fatal myocardial infarction or stroke) with well defined criteria, and had at least 1 year of follow-up after baseline (which was deemed to be sufficient for estimation of risk factor–disease associations in the absence of non-proportional hazards). We did not use prospective cohort studies analysed as nested case-control studies. Details of the contributing studies are described in appendix 1 (pp 3–5, 37–38).

For the recalibration of models, we obtained age-specific and sex-specific incidences of myocardial infarction and stroke from the 2017 update of the GBD study for each of 21 global regions defined by GBD to maximise between-region variability and minimise heterogeneity within each region in mortality and major drivers of health outcomes (appendix 1 p 39).^21^, ^29^ Age-specific and sex-specific risk factor values for each of these regions were estimated by averaging country-specific risk factor values provided by the NCD-RisC.^20^, ^22^, ^23^, ^24^, ^30^

We included prospective cohort studies in the external validation analysis if they met the following criteria: did not contribute to the model derivation stage, met the same methodological criteria as those described for the cohorts selected from the ERFC for the model derivation stage, and made individual participant data accessible for analysis to investigators in our working group. Studies used for external validation included the following: the Asia Pacific Cohort Studies Collaboration (APCSC),^31^ the New Zealand primary care-based PREDICT cardiovascular disease cohort (PREDICT-CVD),^12^ the Chinese Multi-Provincial Cohort Study,^32^ the Health Checks Ubon Ratchathani Study^33^ in Thailand, the Tehran Lipids and Glucose Study,^34^ and UK Biobank (appendix 1 p 6).^35^

To mirror the populations typically targeted in primary prevention efforts for cardiovascular disease, risk model derivation included participants aged 40–80 years without a known baseline history of cardiovascular disease. Follow-up was until the first myocardial infarction, fatal coronary heart disease, or stroke event; outcomes were censored if a participant was lost to follow-up, died from non-cardiovascular disease causes, or reached 10 years of follow-up. Conventional cardiovascular disease risk factors were considered for selection as variables in risk models if they were known to be predictive of cardiovascular disease in different populations, were recorded in available survey data to allow systematic recalibration within each global region,^20^, ^22^, ^23^, ^24^, ^30^ and had been shown to be measurable at low cost in low-income and middle-income countries.^20^ We derived two types of new WHO risk prediction models for cardiovascular disease: a laboratory-based model including age, smoking status, systolic blood pressure, history of diabetes, and total cholesterol; and a non-laboratory-based model including age, smoking status, systolic blood pressure, and BMI. Sex-specific models were derived separately for coronary heart disease (defined in the ERFC dataset as non-fatal myocardial infarction or fatal coronary heart disease), and stroke (any fatal or non-fatal cerebrovascular event) outcomes. Details of these endpoint definitions are shown in appendix 1 (p 7). Outcomes were modelled separately for coronary heart disease and stroke to allow separate recalibration to the disease-specific incidence in the target populations before combination in a single estimation equation for cardiovascular disease risk (appendix 1 pp 40–41). The assumption of independence between coronary heart disease and stroke risk was checked with data from ERFC cohorts (appendix 1 p 15).

### Statistical analysis

We estimated hazard ratios (HRs) using Cox proportional hazards models, stratified by study and with duration (ie, time from entry into the study) as the timescale (in subsidiary analyses, models were also fitted with age as the timescale). Interactions between baseline age and other predictors were included because outcome associations commonly vary with age.^36^, ^37^, ^38^ Continuous variables were centred to aid interpretation of regression model estimates and facilitate recalibration of the models to new populations, with age centred at 60 years (the midpoint of the defined 40–80 years age range), total cholesterol at 6 mmol/L, BMI at 25 kg/m^2^, and systolic blood pressure at 120 mm Hg. Deviation from the proportional hazards assumption was either minimal or non-existent, assessed by fitting models including time-varying covariates. Between-study heterogeneity was assessed using the *I*^2^ statistic.^39^ We used meta-regression to assess heterogeneity by geographical region and period of cohort enrolment.^40^

For internal validation, we assessed risk discrimination using Harrell's C index. This index estimates the probability of the model correctly predicting who will have a cardiovascular disease event first in a randomly selected pair of participants.^41^ To avoid optimism that might result from assessing risk discrimination in the data from which the model was derived, we used an internal–external validation approach in which each study was, in turn, left out of the model derivation and used to calculate a validation C index.^42^ The calibration of each model within studies with at least 10 years of follow-up in the derivation dataset was checked by comparing observed and predicted risk across deciles of predicted risk and by calculating a χ^2^ statistic to quantify any evidence of lack of agreement or fit (appendix 1 p 40).^43^

Recalibration was done separately for men and women (description in appendix 1 pp 16,40–41).^44^ This process involved the use of age-specific and sex-specific mean risk factor levels and annual incidence estimates of fatal or non-fatal myocardial infarction and stroke events in each of 21 global regions (appendix 1 p 43). Calibration of the new WHO models was assessed by comparing the predicted 10-year cardiovascular disease risk with the expected 10-year risk estimated from the 2017 GBD annual incidence estimates, across 5-year age groups. An additional external calibration assessment was completed in the PREDICT-CVD cohort (the only nationally representative validation cohort available to us). Because fewer than 10 years of follow-up were available in this cohort, we recalibrated models to estimate 5-year risk. We assessed discrimination using all external validation cohorts by calculating study-specific C indices before pooling by country, weighting by number of events.^45^ Additionally, we compared C indices for the same prediction models derived within datasets used for external validation with those calculated for the new WHO models. To compare the proportion of the population at different levels of cardiovascular disease risk, with the WHO models, across multiple countries, we applied the risk models to WHO STEPS surveys data. To allow comparison across countries, we restricted analysis to the latest survey year available for each country and to individuals aged 40–64 years, with total cholesterol between 2·6–10·3 mmol/L, and complete data on relevant variables (appendix 1 pp 8–9). These data were also used to compare risk estimates obtained with non-laboratory-based models with those obtained with laboratory-based models.

Our approach to model development and validation complies with the guideline for Transparent Reporting of a multivariable prediction model for Individual Prognosis Or Diagnosis (appendix 1 pp 44–45). Analyses were done with Stata, version 14, two-sided p values, and 95% CIs. The study was designed and done by the WHO CVD Risk Chart Working Group in collaboration with the ERFC academic coordinating centre and was approved by the Cambridgeshire Ethics Review Committee.

### Role of the funding source

The academic investigators and representatives of WHO participated in the design and oversight of the project. The academic investigators at the coordinating centre had full access to all the data and had final responsibility for the decision to submit for publication. All authors gave approval to submit for publication.

## Results

Our risk model derivation involved 376 177 participants without preceding cardiovascular disease, recruited between 1960 and 2013 (table 1, appendix 1 pp 3–5,10). Mean age was 54 years (SD 9) among men and 56 years (9) among women. 247 699 (66%) of 376 177 participants were recruited in European countries, 85 098 (23%) in North America, and the remainder mostly in Japan and Australia. During the initial 10 years of follow-up (3·2 million person-years at risk) 19 333 cardiovascular disease events were observed (table 1, appendix 1 pp 3–5). HRs for myocardial infarction or fatal coronary heart disease and stroke for each risk predictor included in the WHO models are provided in table 2. Associations of history of diabetes and current smoking status with cardiovascular disease diminished with age, particularly in women, among whom HRs for myocardial infarction or fatal coronary heart disease were reduced from 4·65 (95% CI 3·46–6·24) for history of diabetes and 5·58 (4·58–6·81) for smoking status at age 40 years to 2·31 (2·04–2·62) for history of diabetes and 2·05 (1·85–2·29) for smoking status at age 70 years (appendix 1 p 17). We found little to moderate heterogeneity in HRs across studies and no evidence to suggest differences in HRs acccording to geographical regions or period of cohort enrolment (appendix 1 p 11). Calibration and goodness of fit for the prediction models were good within the ERFC dataset, both overall (appendix 1 p 18) and within specific regions and recruitment time periods (appendix 1 p 19). Internally validated C indices ranged from 0·666 (95% CI 0·661–0·672) in men with the non-laboratory-based model to 0·757 (0·749–0·765) in women with the laboratory-based model (appendix 1 p 12).

**Table 1:** Summary of available data from the Emerging Risk Factors Collaboration used in WHO risk model derivation

	**Men**	**Women**
**Study-level characteristics**		
Number of studies	80	62
Year of recruitment*	1960–2008	1960–2013
**Baseline characteristics**		
Total participants	202 962	173 215
Age at baseline survey (years)	53 (48–60)	55 (49–63)
Systolic blood pressure (mm Hg)	132 (120–146)	130 (118–145)
Total cholesterol (mmol/L)	5·7 (5·0–6·5)	5·9 (5·2–6·7)
Current smoking status	76 943 (37·9%)	38 170 (22·0%)
History of diabetes	9939 (4·9%)	8008 (4·6%)
BMI (kg/m^2^)†	25·6 (23·5–28·0)	25·3 (22·8–28·6)
**Cardiovascular outcomes**‡		
Fatal or non-fatal MI or CHD death§	18 987	7226
Fatal or non-fatal stroke¶	8870	6682
Follow-up to first cardiovascular disease event (years; median [5–95th percentile range])	10·3 (3·4–30·4)	13·1 (4·4–27·0)

Data are n (%) or median (25–75th percentile range), unless otherwise specified. Data are from a total of 85 cohorts with 376 177 participants. BMI=body-mass index. MI=myocardial infarction. CHD=coronary heart disease.

* 41 cohorts (including 47% of total participants) had the median year of study baseline before 1990; 44 cohorts (including 53% of total participants) had the median year of study baseline of 1990 or after.

† Percentage of individuals in WHO-defined BMI categories were the following (in kg/m^2^): 1·3% with BMI lower than 18·5, 43·2% with BMI 18·5–24·9, 40·5% with BMI 25·0–29·9, 11·6% with BMI 30–34·9, 2·6% with BMI 35·0–40·0, and 0·8% with BMI higher than 40.

‡ Specific International Classification of Diseases codes are given for each endpoint in the appendix (p 7).

§ Number of fatal or non-fatal MI events or CHD deaths occurring during the first 10 years of follow-up: 9456 in men and 3151 in women.

¶ Number of fatal or non-fatal stroke events during the first 10 years of follow-up: 3722 in men and 3004 in women.

**Table 2:** Summary of HRs for predictor variables in the WHO risk models derived with use of Emerging Risk Factors Collaboration data

		**Men**		**Women**	
		Main effect	Age interaction term*	Main effect	Age interaction term*
**Laboratory-based models**					
Fatal or non-fatal MI or CHD death					
	Age at baseline per 5 years	1·43 (1·40–1·47)	..	1·67 (1·60–1·73)	..
	Current smoking status	1·76 (1·68–1·84)	0·91 (0·89–0·93)	2·87 (2·64–3·11)	0·85 (0·81–0·88)
	Systolic blood pressure per 20 mm Hg	1·30 (1·28–1·33)	0·98 (0·97–0·99)	1·37 (1·33–1·42)	0·99 (0·97–1·00)
	History of diabetes	1·90 (1·76–2·04)	0·94 (0·91–0·97)	2·92 (2·60–3·28)	0·89 (0·84–0·94)
	Total cholesterol per 1 mmol/L	1·26 (1·24–1·28)	0·98 (0·97–0·99)	1·23 (1·20–1·26)	0·97 (0·96–0·99)
	Baseline survival estimate at 10 years†	0·954	..	0·989	..
Fatal or non-fatal stroke					
	Age at baseline per 5 years	1·64 (1·58–1·70)	..	1·70 (1·63–1·76)	..
	Current smoking status	1·65 (1·53–1·77)	0·93 (0·89–0·96)	2·11 (1·92–2·31)	0·90 (0·86–0·95)
	Systolic blood pressure per 20 mm Hg	1·56 (1·51–1·61)	0·96 (0·95–0·97)	1·51 (1·46–1·56)	0·95 (0·94–0·97)
	History of diabetes	1·87 (1·67–2·10)	0·88 (0·83–0·93)	2·36 (2·06–2·70)	0·90 (0·84–0·96)
	Total cholesterol per 1 mmol/L	1·03 (1·00–1·06)	1·01 (0·99–1·02)	1·03 (0·99–1·06)	0·99 (0·97–1·01)
	Baseline survival estimate at 10 years†	0·985	..	0·989	..
**Non-laboratory-based models**					
Fatal or non-fatal MI or CHD death					
	Age at baseline per 5 years	1·44 (1·41–1·48)	..	1·69 (1·63–1·76)	..
	Current smoking status	1·81 (1·73–1·90)	0·90 (0·88–0·93)	2·98 (2·75–3·24)	0·84 (0·81–0·88)
	Systolic blood pressure per 20 mm Hg	1·31 (1·28–1·33)	0·98 (0·97–0·99)	1·40 (1·35–1·44)	0·98 (0·97–1·00)
	BMI per 1 kg/m^2^	1·18 (1·15–1·22)	0·97 (0·96–0·99)	1·14 (1·10–1·18)	0·98 (0·97–1·00)
	Baseline survival estimate at 10 years†	0·954	..	0·989	..
Fatal or non-fatal stroke					
	Age at baseline per 5 years	1·63 (1·57–1·69)	..	1·69 (1·63–1·75)	..
	Current smoking status	1·65 (1·53–1·78)	0·93 (0·89–0·96)	2·10 (1·91–2·30)	0·90 (0·86–0·95)
	Systolic blood pressure per 20 mm Hg	1·58 (1·53–1·62)	0·96 (0·94–0·97)	1·54 (1·49–1·60)	0·95 (0·93–0·96)
	BMI per kg/m^2^	1·08 (1·03–1·13)	0·99 (0·97–1·01)	1·02 (0·98–1·06)	1·00 (0·98–1·02)
	Baseline survival estimate at 10 years†	0·985	..	0·989	..

Data are HRs (95% CI) from sex-specific Cox-proportional hazards models, stratified by study. Log HRs and heterogeneity statistics are given in appendix 1 (p 11). Age was centred at 60 years, systolic blood pressure at 120 mm Hg, total cholesterol at 6 mmol/L, and BMI at 25 kg/m^2^. Smoking status was coded as current versus other, and history of diabetes as yes versus no. MI=myocardial infarction. CHD=coronary heart disease. BMI=body-mass index. HR=hazard ratio.

* Age at baseline.

† Baseline survival for each model was estimated by pooling the baseline survival at 10 years across studies with ≥10 years follow-up weighted by number of events by 10 years.

**Figure 2:**
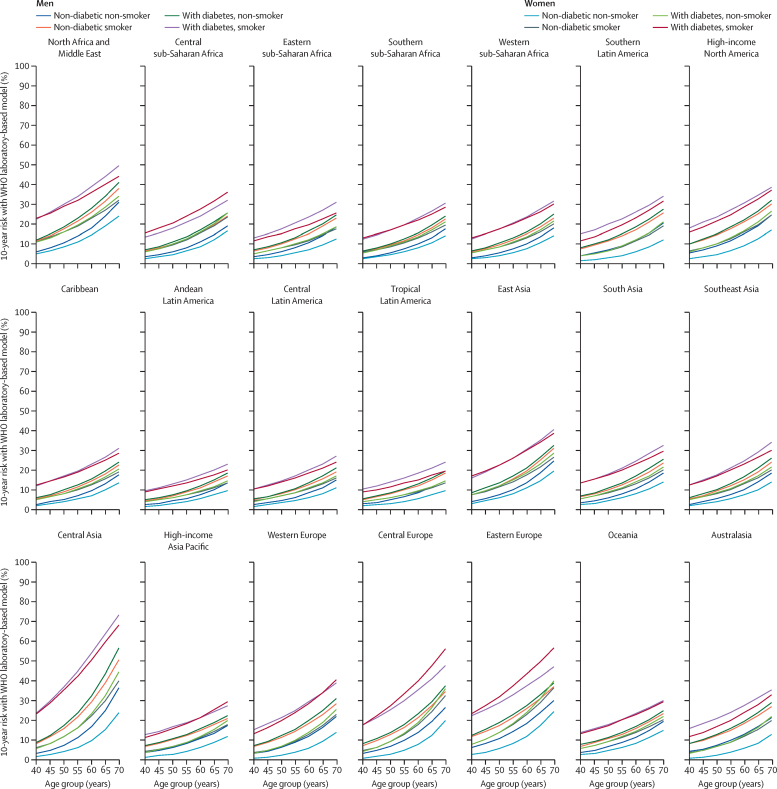
Predicted 10-year cardiovascular disease risks for an individual with total cholesterol concentrations of 5 mmol/L and systolic blood pressure of 140 mm Hg, with the WHO laboratory-based model, for each region Countries included in the 21 regions defined by the Global Burden of Disease Study are provided in appendix 1 (p 39).

According to 2017 GBD estimates, the relative contribution of myocardial infarction and stroke differed substantially by region and sex (appendix 1 pp 20–22), reinforcing the need for separate recalibration of individual models for each endpoint. Myocardial infarction incidence was greater for men than for women in all regions, but the incidence of stroke was more similar between sexes (appendix 1 pp 23–24). The age-specific and sex-specific mean risk factor levels used for recalibration are presented by region in appendix 1 (pp 25–29). The revised WHO charts for cardiovascular disease risk estimation in 21 global regions are shown in appendix 2 for the laboratory-based and non-laboratory-based models. The predicted 10-year cardiovascular disease risk estimated with the WHO models was within the expected 95% CI ranges, on the basis of uncertainty in GBD estimates (appendix 1 pp 30–31). Additionally, we observed a good agreement between 5-year predicted and observed risk in the PREDICT-CVD cohort (appendix 1 p 32). The estimated absolute risk for a given age and combination of risk factors differed substantially across regions (figure 2). For example, the estimated 10-year cardiovascular disease risk for a 60-year-old male smoker without diabetes and with systolic blood pressure of 140 mm Hg and total cholesterol of 5 mmol/L ranged from 11% in Andean Latin America to 30% in central Asia. Similarly, the 10-year risk for a 60-year-old woman with the same risk factor profile ranged from 9% in Andean Latin America to 23% in eastern Europe, north Africa, and the Middle East.

**Figure 3:**
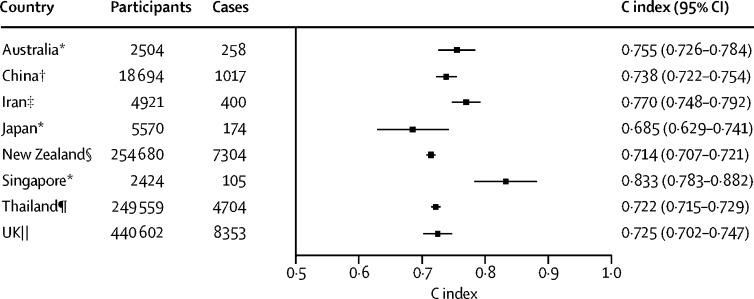
C index upon assessing ability of the laboratory-based WHO model to discriminate cardiovascular disease events in external validation cohorts Where multiple studies are used, country-specific estimates are the result of pooling study-specific C-index values, weighting by the number of events. APCSC=Asia Pacific Cohorts Studies Collaboration. *Calculated with data from studies from the APCSC. †Calculated with data from studies from the APCSC and the China Multi-Provincial Cohort Study. ‡Calculated with data from the Tehran Lipids and Glucose Study. §Calculated with data from studies from the APCSC and the PREDICT-CVD cohort. ¶Calculated with data from the Health Checks Ubon Ratchathani Study. ‖Calculated with data from the UK Biobank.

**Figure 4:**
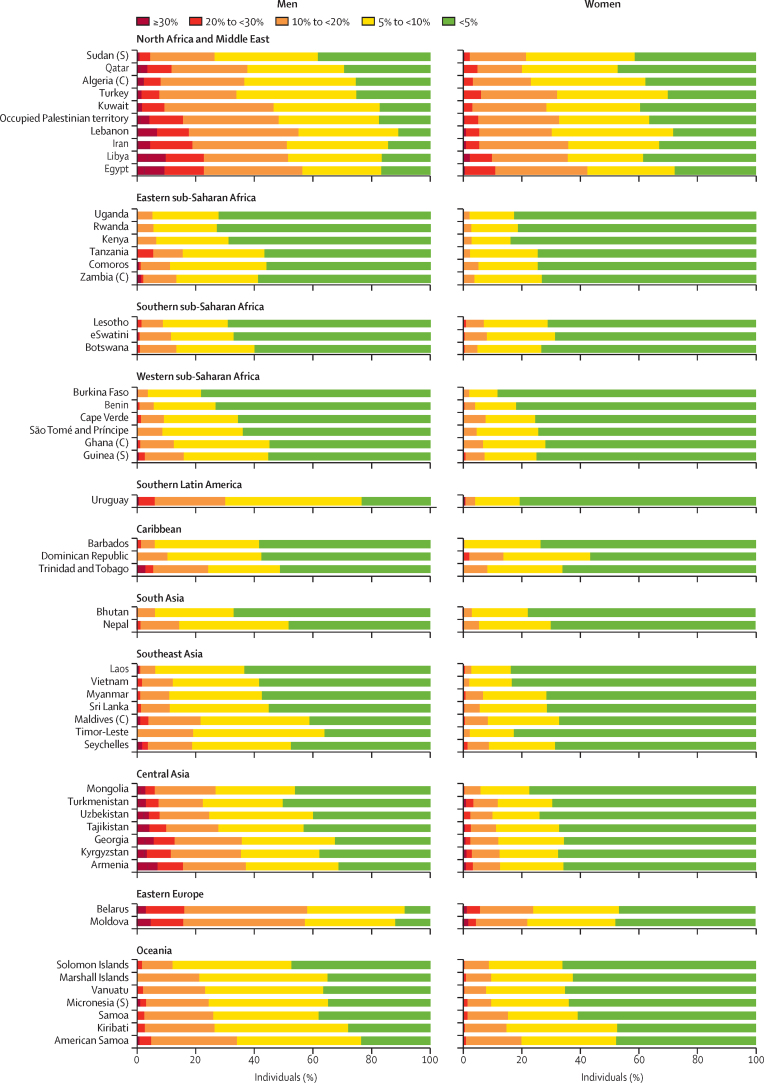
Distribution of 10-year cardiovascular disease risk according to recalibrated laboratory-based WHO risk prediction models for individuals aged 40–64 years from example countries Data from all countries are from adults aged 40–64 years with total cholesterol concentrations of 2·6–10·3 mmol/L and from samples representative of the national population, unless otherwise specified as subnational (S) or community based (C).

External validation of risk models involved calculation of C indices with use of data from 1 096 061 participants with no previous cardiovascular disease, recruited into 19 prospective cohorts (25 950 cardiovascular disease events observed; appendix 1 p 6). C indices indicated good discrimination, with values for the WHO laboratory-based risk model ranging from 0·685 (95% CI 0·629–0·741) to 0·833 (0·783–0·882; figure 3). Furthermore, deriving individual models of myocardial infarction or fatal coronary heart disease and stroke risk directly in the APCSC gave broadly similar HRs to those found in ERFC (appendix 1 p 13); C indices obtained with either the WHO or APCSC models were almost identical (appendix 1 p 12). When we applied recalibrated WHO laboratory-based models to data from the 79 countries in the WHO-STEPS surveys (54 of which had sufficient data for use with the laboratory-based model; appendix 1 pp 8–9), the proportion of individuals aged 40–64 years with an estimated risk greater than 20% varied by region and country, from less than 1% for Uganda to greater than 16% for Egypt (figure 4). We observed small reductions in the C-index when comparing the non-laboratory-based model with the laboratory-based risk model (appendix 1 p 33). The risk distributions according to the non-laboratory-based model are provided in appendix 1 (p 34).

Overall, we found moderate agreement between risk predictions based on laboratory and non-laboratory models. Of individuals at greater than 20% risk using the laboratory-based models, more than 97% of men and women were also identified as being at greater than 10% risk with the non-laboratory-based models (appendix 1 p 35). However, when using a 20% threshold with non-laboratory-based models, about 65% of men and 35% of women were identified. This discrepancy was largely due to poor performance of the non-laboratory models in people with diabetes (appendix 1 p 36). For example, among individuals with diabetes classified as being at greater than 20% risk with the laboratory-based models, about 45% of men and 25% of women were classified as being at greater than 20% risk with the non-laboratory-based models (whereas in individuals without diabetes, about 85% of men and 95% of women showed such agreement; appendix 1 p 36).

## Discussion

We have developed, evaluated, and illustrated the use of revised prediction models for cardiovascular disease risk adapted for low-income and middle-income countries (appendix 2), with the aim of their incorporation into the WHO HEARTS package.^4^ These models have been systematically recalibrated to contemporary risk factor levels and disease incidences across 21 global regions, thereby enabling more accurate identification of individuals at high risk of cardiovascular disease in different settings.^46^ Because the approach to recalibration that we used allows rapid revision of cardiovascular disease models, it should enable flexible updating of models as relevant new epidemiological data emerge about cardiovascular disease trends in particular geographical areas.

The risk models described here involve several features that should confer advantages compared with existing tools.^8^, ^9^, ^13^, ^47^, ^48^, ^49^ First, these models are underpinned by powerful, extensive, and complementary datasets of global relevance, used in a series of interrelated analyses for model derivation, recalibration, validation, and illustration of cardiovascular disease risk.^20^, ^21^, ^22^, ^23^, ^24^ In particular, the scale and geographical resolution of the datasets analysed have enhanced the validity and generalisability of risk models for each sex-specific and disease-specific (myocardial infarction and stroke) endpoint reported here.

A second feature is the simplicity of the recalibration approach we have developed. This approach entails fewer modelling steps and avoids reliance on sparse cohort or country-level data, providing recalibrated calculators tailored to the sex-specific cardiovascular disease rates and risk factor levels of each region.^48^, ^50^, ^51^ Because the approach can be used with aggregate (ie, group level) data on cardiovascular disease incidences and with average risk factor values for any target population to be screened, this means that descriptive epidemiological data can be readily incorporated to revise models according to country-specific cardiovascular disease incidence to reflect changes in disease incidences and risk factor profiles. To support periodic revisions, we have made openly accessible the statistical code needed to calculate, validate, and recalibrate these models using updated population data.

A third feature is that the risk models reported here provide estimates for the combined outcome of fatal and non-fatal events, thereby improving on risk calculators that predict fatal events alone.^8^ Although information on fatal event rates is often easier to obtain at a country-specific level, the use of mortality risk models might underestimate total cardiovascular disease risk, particularly for individuals in populations where the case-fatality rate is low (as is typically observed among younger individuals).^15^ Because the models reported here have been specifically derived for and recalibrated to the sex-specific and age-specific rates of myocardial infarction and stroke in each region, they should avoid inaccuracies that could arise from recalibration to overall cardiovascular disease rates,^48^ including inconsistencies in reporting softer endpoints (such as angina) across regions.

A fourth feature is the assessment of pragmatic models that do not assume availability of laboratory measurements (eg, serum lipid concentrations). Such simplified approaches could be used in resource-constrained settings as part of stepwise approaches to help target laboratory testing in people most likely to benefit from the extra information (eg, pre-selection tools),^26^ and used even when values for some risk factors are unavailable for individuals (when mean values from the relevant population can be used as crude surrogates).^13^ However, we found that an important limitation of such pragmatic scores was their poor performance among people with diabetes.

A fifth feature was that, because we could illustrate the performance of the new models with reference to surveillance data from 79 countries, our data have shown that the proportion of individuals across different risk categories is strikingly different across global regions. This finding suggests that our risk estimates should assist policy makers to make more appropriate and locally informed decisions about the allocation of prevention resources.

Finally, we have presented revised risk charts in an analogous manner to previous WHO–International Society of Hypertension (ISH) versions to help facilitate continuity of use. Nevertheless, the colour code has been revised to reflect the general lower estimated absolute risk levels compared with those of previous WHO–ISH models.^47^ Orange sections now indicate 10-year risk greater than 10%, whereas red sections indicate a risk greater than 20% (as opposed to >20% indicated in orange and >30% indicated in red previously).

The potential limitations of our study merit consideration. We derived risk prediction models from 85 cohorts mostly from high-income countries in the ERFC. Ideally, however, the derivation of risk models for low-income and middle-income countries would involve nationally representative, large-scale prospective cohort data from several of these countries, each cohort with long-term follow-up and validated fatal and non-fatal endpoints. Unfortunately, however, such data do not yet exist for most low-income and middle-income countries.^21^, ^29^, ^52^ Therefore, to inform recalibration, we used data from the GBD study and the NCD-RisC, acknowledging that these sources frequently do not have country-specific disease risk estimates because of the paucity or absence of such data.^21^, ^29^, ^52^

To provide external validation, we analysed data from 19 cohorts distinct from those used in model derivation. However, only one of them (PREDICT-CVD cohort) was nationally representative, whereas some of the other cohorts might have inadequately represented the epidemiology of cardiovascular disease in contemporary national populations of interest.^44^ Our risk models might have overestimated cardiovascular disease risk for primary prevention purposes because incidences from global regions used to recalibrate models were likely to include some recurrent events (although the extent of such overestimation is difficult to quantify).^53^ Conversely, our risk models might have underestimated cardiovascular disease risk because population data used to estimate incidences were likely to include some people already on cardiovascular disease prevention therapies (eg, statins or anti-hypertensive medication). However, data available to us were insufficient to explore this issue in detail. We could not compare the performance of our new risk models with risk equations already developed for use in specific high-income countries or regions because these equations typically contain some variables that are not available (or cannot be practicably measured) in low-income and middle-income countries.^6^, ^8^, ^12^, ^13^, ^16^, ^54^ Models were derived on participants with complete risk factor information, which, in principle, could cause a loss in efficiency and bias results. However, our analyses were well powered and should be unbiased under the reasonable assumption that the probability of an individual having complete risk factor information is independent of cardiovascular disease, given the variables included in the prediction model.^55^

In conclusion, we have derived, validated, and illustrated new WHO risk prediction models to estimate cardiovascular disease risk in 21 GBD regions. Because the risk prediction models reported here have been adapted to the contemporary circumstances of many different global regions and can be readily updated with routinely available information, their widespread use could enhance the accuracy, practicability, and sustainability of efforts to reduce the burden of cardiovascular disease worldwide.
