# Correction to *Lancet Glob Health* 2019; 7: e1332–45

**DOI:** 10.1016/S2214-109X(22)00522-8

**Published:** 2022-12-06

*The WHO CVD Risk Chart Working Group. World Health Organization cardiovascular disease risk charts: revised models to estimate risk in 21 global regions.* Lancet Glob Health *2019; **7:** e1332–45*—The appendix of this Article has been corrected as of Dec 6, 2022.

