# Supplementary material for: World Health Organization cardiovascular disease risk charts: revised models to estimate risk in 21 global regions
Source: Lancet Glob Health. 2019 Sep 2;7(10):e1332–45. doi: 10.1016/S2214-109X(19)30318-3 (PMC7025029; doi:10.1016/S2214-109X(19)30318-3)
Supplement: Supplementary appendix 1 [file mmc1.pdf]

THE LANCET
Global Health

Supplementary appendix 1

This appendix formed part of the original submission and has been peer reviewed. We post it as supplied by the authors.

Supplement to: The WHO CVD Risk Chart Working Group. World Health Organization cardiovascular disease risk charts: revised models to estimate risk in 21 global regions. *Lancet Glob Health* 2019; published online Sept 2. http://dx.doi.org/10.1016/S2214-109X(19)30318-3.

Supplementary material

World Health Organization cardiovascular disease risk charts: revised models to estimate risk in 21 global regions

Table of contents

1) Supplementary tables and figures

Table 1.1: Baseline characteristics and event summaries from available studies in the ERFC 3

Table 1.2: Baseline characteristics and event summaries from studies used in external validation 6

Table 1.3: Endpoint definitions used in model derivation and validation 7

Table 1.4: Summary of available country specific national survey data from WHO-STEPS 8

Table 1.5: Summary of available data from the ERFC used in WHO risk model derivation, by sex 10

Table 1.6: Log hazard ratios, standard errors and heterogeneity statistics for the WHO risk models 11

Table 1.7: Internal and external validation using the C-index 12

Table 1.8: Comparison of hazard ratios from the risk models obtained using ERFC and APCSC data 13

Figure 1.1: Data selection process for the ERFC datasets used in risk model derivation 14

Figure 1.2: Comparison of 10-year risk estimates using risk models with and without adjustment for competing risks 15

Figure 1.3: Process for recalibration of the WHO risk models 16

Figure 1.4: Changes in hazard ratios with age for the risk factors in the WHO risk models 17

Figure 1.5: Calibration of the WHO risk models within the ERFC dataset 18

Figure 1.6: Calibration of the WHO risk models within the ERFC dataset by region and year of baseline survey 19

Figure 1.7: Annual MI incidence by region sex and age group used for recalibration of WHO models 20

Figure 1.8: Annual stroke incidence by region sex and age group used for recalibration of WHO models 21

Figure 1.9: Standardised estimates of region-specific Stroke:MI incidence rate ratios 22

Figure 1.10: Standardised estimates of region-specific Female:Male MI incidence rate ratios 23

Figure 1.11: Standardised estimates of region-specific Female:Male stroke incidence rate ratios 24

Figure 1.12 : Total cholesterol values by region sex and age group used for recalibration of WHO models 25

Figure 1.13 : Systolic blood pressure values by region sex and age group used for recalibration of WHO models 26

Figure 1.14: Diabetes prevalence by region sex and age group used for recalibration of WHO models 27

Figure 1.15: Smoking prevalence by region sex and age group used for recalibration of WHO models 28

Figure 1.16: BMI levels by region sex and age group used for recalibration of WHO models 29

Figure 1.17: Comparison of expected 10-year CVD risks in men from 21 global regions vs risks estimated using un-calibrated and recalibrated WHO laboratory based model 30

Figure 1.18: Comparison of expected 10-year CVD risks in women from 21 global regions vs risks estimated using un-calibrated and recalibrated WHO laboratory based model 31

Figure 1.19: Calibration of 5-year WHO risk models in the PREDICT cohort 32

Figure 1.20: Ability of the laboratory and non-laboratory based WHO model to discriminate 33

Figure 1.21: Distribution of risk according to recalibrated non-laboratory based WHO CVD risk prediction models 34

Figure 1.22: Risk classification using laboratory vs non-lab based WHO risk models, by sex 35

Figure 1.23: Risk classification using laboratory vs non-lab based WHO risk models, by sex and diabetes status 36

2) List of ERFC studies and abbreviations 37
3) Countries included in the 21 regions defined by the Global Burden of Disease Study 39
4) Supplementary statistical methods 40
5) Endpoint definitions used for GBD estimated incidence rates 43
6) TRIPOD checklist for prediction model development and validation 44

1) Supplementary tables and figures

Table 1.1: Baseline characteristics and event summaries from available studies in the Emerging Risk Factors Collaboration

Country	Cohort	No of participants	Median year of study recruitment	Ages mean(sd)	Men n (%)	Current smoker n (%)	History of diabetes n (%)	Systolic BP (mmHg) mean(sd)	Total cholesterol (mmol/L) mean(sd)	BMI (kg/m²)	Median Follow-up (5th and 95th percentile)	MI or fatal CHD	Cerebro-vascular	MI or fatal CHD By 10yrs	Cerebro-vascular By 10yrs
Denmark	COPEN	6653	1993	61 (10)	2792 (42)	3355 (50)	220 (3.3)	141 (22)	6.3 (1.2)	26 (4)	16.4 (2.4 to 18.4)	577	638	364	383
Finland	FINE_FIN	206	1989	74 (3)	206 (100)	33 (16)	21 (10.2)	156 (22)	5.7 (1.1)	26 (4)	7.5 (2.2 to 10.0)	56	20	56	20
Finland	FINRISK92	3580	1992	52 (7)	1661 (46)	932 (26)	89 (2.5)	141 (20)	5.9 (1.1)	27 (4)	16.8 (2.3 to 16.9)	131	113	108	91
Finland	FINRISK97	5382	1997	55 (9)	2750 (51)	1313 (24)	221 (4.1)	141 (20)	5.8 (1.0)	27 (4)	11.8 (1.8 to 11.9)	127	114	126	111
Finland	KIHD	2037	1987	52 (5)	2037 (100)	622 (31)	89 (4.4)	131 (17)	5.9 (1.1)	27 (4)	20.9 (3.0 to 25.1)	398	149	191	75
France	DESIR	2852	1995	52 (7)	1394 (49)	431 (15)	153 (5.4)	133 (16)	5.9 (1.0)	25 (4)	9.0 (8.4 to 9.4)	23	13	23	13
France / NI	PRIME	9526	1992	55 (3)	9526 (100)	2529 (27)	312 (3.3)	134 (19)	5.7 (1.0)	27 (3)	5.2 (5.0 to 7.3)	145	42	145	42
Germany	ESTHER	7147	2001	61 (7)	3038 (43)	1216 (17)	844 (11.8)	139 (20)	5.7 (1.3)	28 (4)	5.0 (2.0 to 5.9)	76	131	76	131
Germany	GRIPS	5783	1982	48 (5)	5783 (100)	2177 (38)	113 (2.0)	132 (16)	5.6 (1.0)	26 (3)	9.8 (4.8 to 10.0)	299	103	299	103
Germany	MONICA_KORA1	874	1985	54 (6)	874 (100)	296 (34)	37 (4.2)	137 (18)	6.4 (1.2)	28 (3)	13.0 (3.6 to 13.4)	79	5	59	2
Germany	MONICA_KORA2	3261	1990	56 (10)	1619 (50)	706 (22)	191 (5.9)	136 (20)	6.4 (1.2)	27 (4)	7.8 (1.8 to 8.4)	100	6	100	6
Germany	MONICA_KORA3	3179	1995	56 (10)	1567 (49)	663 (21)	197 (6.2)	137 (20)	6.2 (1.1)	28 (4)	13.9 (3.4 to 14.7)	195	38	137	22
Germany	PROCAM	13062	1984	50 (6)	9704 (74)	4541 (35)	369 (2.8)	133 (19)	5.9 (1.1)	26 (3)	8.7 (3.6 to 18.5)	447	96	285	51
Germany	SHIP	1393	1999	54 (9)	673 (48)	326 (23)	116 (8.3)	138 (20)	6.0 (1.2)	28 (4)	10.8 (10.2 to 12.4)	27	27	26	20
Germany	WCWC	3206	1989	49 (5)	3206 (100)	1271 (40)	175 (5.5)	140 (19)	6.1 (1.3)	27 (3)	5.9 (2.1 to 14.6)	22	0	19	0
Iceland	REYK	15747	1975	53 (8)	7559 (48)	7370 (47)	310 (2.0)	138 (22)	6.5 (1.2)	25 (4)	24.0 (5.9 to 37.0)	3158	708	725	94
Italy	ATENA	4396	1995	51 (7)	0 (0)	1731 (39)	115 (2.6)	135 (21)	6.2 (1.2)	27 (4)	6.8 (5.2 to 8.1)	18	5	18	5
Italy	BRUN	794	1990	57 (11)	387 (49)	193 (24)	28 (3.5)	145 (22)	5.7 (1.0)	25 (4)	20.2 (4.4 to 20.5)	63	58	24	25
Italy	CASTEL	2152	1984	72 (4)	843 (39)	304 (14)	278 (12.9)	160 (24)	5.7 (1.1)	27 (4)	11.4 (2.7 to 14.0)	73	76	55	59
Italy	EMOFRI	360	1996	55 (6)	176 (49)	92 (26)	17 (4.7)	146 (18)	5.9 (1.1)	26 (4)	6.8 (6.5 to 7.2)	2	3	2	3
Italy	FINE_IT	445	1985	72 (4)	445 (100)	122 (27)	36 (8.1)	167 (22)	5.8 (1.1)	26 (4)	10.1 (1.8 to 21.4)	66	99	45	62
Italy	MATISS83	2192	1984	54 (8)	1036 (47)	630 (29)	121 (5.5)	142 (23)	5.9 (1.2)	28 (4)	18.6 (5.9 to 19.5)	79	102	38	28
Italy	MATISS87	1795	1987	54 (8)	790 (44)	377 (21)	73 (4.1)	143 (22)	5.7 (1.0)	29 (4)	15.6 (6.8 to 16.2)	42	59	26	30
Italy	MATISS93	981	1994	52 (8)	488 (50)	247 (25)	55 (5.6)	142 (23)	5.7 (1.1)	28 (5)	8.3 (7.0 to 9.3)	13	6	13	6
Italy	MONFRI86	926	1986	52 (7)	447 (48)	286 (31)	11 (1.2)	142 (21)	6.3 (1.2)	27 (4)	16.7 (6.7 to 16.9)	24	19	11	8
Italy	MONFRI89	901	1989	51 (7)	442 (49)	252 (28)	13 (1.4)	141 (22)	6.0 (1.2)	27 (4)	13.6 (6.6 to 13.7)	17	15	13	11
Italy	MONFRI94	1086	1994	51 (7)	519 (48)	273 (25)	54 (5.0)	140 (20)	5.9 (1.1)	27 (4)	8.5 (7.0 to 8.8)	10	16	10	16
Netherlands	HOORN	2218	1991	61 (7)	980 (44)	705 (32)	209 (9.4)	135 (20)	6.7 (1.2)	27 (4)	8.8 (3.5 to 9.9)	73	53	73	53
Netherlands	PREVEND	4810	1998	54 (10)	2326 (48)	1572 (33)	216 (4.5)	132 (21)	5.9 (1.1)	27 (4)	10.6 (3.2 to 11.2)	163	168	149	151
Netherlands	ProspectEPIC	14753	1995	57 (6)	0 (0)	3354 (23)	281 (1.9)	133 (20)	6.1 (1.1)	26 (4)	14.3 (8.0 to 17.2)	260	331	207	225
Netherlands	RS_I	4197	1992	66 (7)	1663 (40)	837 (20)	329 (7.8)	138 (22)	6.7 (1.2)	26 (4)	12.0 (3.8 to 14.2)	218	165	174	103
Netherlands	RS_II	2016	2000	63 (6)	896 (44)	499 (25)	197 (9.8)	142 (21)	5.9 (0.9)	27 (4)	10.1 (4.3 to 10.9)	70	35	68	33
Netherlands	ZUTE	735	1985	61 (12)	735 (100)	356 (48)	62 (8.4)	146 (21)	6.1 (1.1)	25 (3)	15.1 (2.1 to 40.0)	168	117	82	52
Norway	TROMSØ	9457	1987	51 (10)	4830 (51)	4256 (45)	128 (1.4)	135 (19)	6.4 (1.2)	25 (4)	18.8 (3.9 to 19.3)	741	476	386	231
Scotland	EAS	1005	1988	64 (6)	503 (50)	214 (21)	43 (4.3)	141 (22)	6.9 (1.3)	25 (4)	20.2 (2.8 to 21.3)	93	84	60	43
Scotland/Ireland/ Netherlands	PROSPER	1511	1998	74 (3)	647 (43)	542 (36)	181 (12.0)	156 (21)	5.7 (0.9)	27 (4)	3.2 (1.1 to 3.9)	127	51	127	51
Spain	ZARAGOZA	2534	1994	60 (10)	1089 (43)	412 (16)	350 (13.8)	135 (15)	5.9 (1.0)	29 (5)	5.1 (4.1 to 5.1)	42	35	42	35
Sweden	GOTO13	609	1967	54 (0)	609 (100)	320 (53)	13 (2.1)	143 (21)	7.0 (1.2)	25 (3)	24.0 (5.0 to 30.5)	166	91	45	12

Country	Cohort	No of participants	Median year of study recruitment	Ages mean(sd)	Men n (%)	Current smoker n (%)	History of diabetes n (%)	Systolic BP (mmHg) mean(sd)	Total cholesterol (mmol/L) mean(sd)	BMI (kg/m^2)	Median Follow-up (5th and 95th percentile)	MI or fatal CHD	Cerebro-vascular	MI or fatal CHD By 10yrs	Cerebro-vascular By 10yrs
Sweden	GOTO33	709	1984	51 (0)	709 (100)	260 (37)	24 (3.4)	134 (17)	6.1 (1.1)	26 (3)	12.8 (5.8 to 13.1)	27	8	19	4
Sweden	GOTO43	767	1993	50 (0)	767 (100)	234 (31)	15 (2.0)	130 (16)	5.9 (1.0)	26 (3)	11.0 (8.0 to 11.7)	27	16	25	12
Sweden	GOTOW	1021	1969	50 (4)	0 (0)	397 (39)	12 (1.2)	137 (23)	7.1 (1.2)	24 (4)	31.8 (9.4 to 32.8)	122	160	12	7
Sweden	MOSWEGOT	2838	1990	52 (7)	1309 (46)	811 (29)	80 (2.8)	132 (19)	6.1 (1.2)	25 (4)	13.8 (6.0 to 19.6)	140	121	92	70
Sweden	MPP	23093	1980	49 (5)	14794 (64)	10122 (44)	877 (3.8)	128 (16)	5.8 (1.1)	25 (4)	26.0 (7.6 to 34.1)	3440	2262	717	222
Sweden	ULSAM	2001	1972	52 (7)	2001 (100)	1161 (58)	127 (6.3)	134 (18)	6.8 (1.3)	25 (3)	24.8 (5.5 to 37.3)	511	287	115	49
UK	BRHS	6670	1979	50 (6)	6670 (100)	3558 (53)	75 (1.1)	145 (21)	6.3 (1.0)	25 (3)	24.5 (4.7 to 25.4)	1196	510	460	124
UK	BWHHS	2674	2000	68 (5)	0 (0)	313 (12)	104 (3.9)	147 (25)	6.7 (1.2)	27 (5)	12.2 (3.5 to 13.3)	99	120	75	97
UK	CAPS	2112	1981	52 (5)	2112 (100)	1155 (55)	30 (1.4)	141 (19)	5.7 (1.1)	26 (4)	13.0 (4.0 to 13.0)	248	17	184	11
UK	EPICNOR1	20301	1996	59 (9)	9024 (44)	2368 (12)	576 (2.8)	135 (18)	6.2 (1.2)	26 (4)	9.7 (5.9 to 12.0)	485	439	445	394
UK	LEADER	425	1997	67 (8)	425 (100)	168 (40)	63 (14.8)	149 (22)	5.6 (0.9)	26 (4)	4.3 (1.0 to 6.8)	53	19	53	19
UK	MIDCOLL	4332	1971	50 (6)	3718 (86)	2457 (57)	22 (0.5)	135 (18)	5.9 (1.0)	25 (3)	26.1 (4.9 to 39.9)	1182	574	261	62
UK	MIDFAM	1694	1996	47 (5)	735 (43)	427 (25)	22 (1.3)	128 (16)	5.3 (1.0)	26 (4)	17.4 (10.0 to 17.8)	50	43	23	17
UK	MIDRP	10796	1974	54 (6)	4843 (45)	5482 (51)	117 (1.1)	149 (24)	6.2 (1.1)	26 (4)	23.1 (4.2 to 36.8)	2707	1740	630	201
UK	NPHSII	2950	1991	57 (3)	2950 (100)	1070 (36)	71 (2.4)	138 (19)	5.7 (1.0)	26 (4)	8.3 (3.4 to 10.4)	192	70	162	55
UK	SHHEC	8988	1986	50 (6)	4564 (51)	3985 (44)	112 (1.2)	132 (19)	6.4 (1.2)	26 (4)	10.0 (6.6 to 10.0)	314	120	314	120
UK	SPEED	2098	1980	55 (4)	2098 (100)	995 (47)	41 (2.0)	140 (23)	5.9 (1.2)	26 (3)	16.7 (3.3 to 18.2)	251	77	174	27
UK	WHITEII	7374	1987	48 (5)	4790 (65)	1935 (26)	76 (1.0)	124 (15)	6.1 (1.2)	25 (4)	17.0 (4.8 to 18.8)	374	7	234	4
UK	WOSCOPS	3095	1990	55 (6)	3095 (100)	1360 (44)	33 (1.1)	136 (17)	7.0 (0.6)	26 (3)	4.8 (2.6 to 6.0)	220	37	220	37
TOTAL Europe		247699		54 (9)	138844 (56)	83613 (34)	8744 (3.5)	136 (21)	6.1 (1.2)	26 (4)	11.7 (3.8 to 31.8)	20026	10894	8622	3938
Canada	NSHS	864	1995	56 (11)	427 (49)	203 (23)	47 (5.4)	129 (17)	5.6 (1.0)	28 (5)	9.8 (4.7 to 10.0)	8	21	8	21
Canada	QUEBEC	2676	1974	49 (7)	2676 (100)	1845 (69)	106 (4.0)	141 (19)	5.0 (1.1)	26 (4)	26.4 (4.2 to 26.9)	498	158	204	39
USA	ARIC	13126	1988	54 (6)	5909 (45)	3663 (28)	1403 (10.7)	121 (18)	5.5 (1.1)	27 (5)	22.3 (5.1 to 24.6)	1117	867	517	276
USA	CHARL	1524	1961	53 (9)	703 (46)	823 (54)	71 (4.7)	147 (29)	6.2 (1.2)	25 (5)	22.4 (3.7 to 48.2)	442	230	120	44
USA	CHS1	3372	1989	71 (4)	1247 (37)	430 (13)	435 (12.9)	135 (21)	5.5 (1.0)	27 (5)	12.2 (2.4 to 12.9)	476	373	365	282
USA	CHS2	406	1993	71 (4)	154 (38)	69 (17)	94 (23.2)	142 (24)	5.4 (1.0)	29 (5)	9.1 (1.9 to 9.5)	41	36	41	36
USA	EPESEBOS	544	1988	75 (2)	188 (35)	71 (13)	111 (20.4)	137 (17)	5.6 (1.1)	27 (5)	4.0 (1.6 to 4.5)	26	19	26	19
USA	EPESEIOW	778	1988	75 (2)	251 (32)	54 (7)	99 (12.7)	138 (17)	5.7 (1.1)	27 (4)	4.8 (1.9 to 4.9)	22	39	22	39
USA	EPESENCA	689	1992	75 (2)	252 (37)	105 (15)	126 (18.3)	143 (21)	5.5 (1.0)	27 (6)	4.0 (1.4 to 4.6)	27	36	27	36
USA	EPESENHA	389	1988	75 (2)	161 (41)	65 (17)	58 (14.9)	139 (18)	5.7 (1.1)	26 (4)	4.4 (2.0 to 4.7)	10	14	10	14
USA	FRAMOFF	2645	1999	60 (9)	1159 (44)	324 (12)	275 (10.4)	126 (18)	5.2 (0.9)	28 (5)	5.2 (3.1 to 7.0)	50	22	50	22
USA	HONOL	1828	1992	76 (2)	1828 (100)	148 (8)	476 (26.0)	148 (22)	5.0 (0.8)	24 (3)	6.4 (1.6 to 7.6)	105	78	105	78
USA	MESA	6467	2001	61 (10)	3062 (47)	996 (15)	805 (12.4)	126 (21)	5.0 (0.9)	28 (6)	8.5 (2.9 to 8.9)	122	121	122	121
USA	MRFIT	5079	1974	48 (5)	5079 (100)	2935 (58)	48 (0.9)	146 (16)	6.5 (0.9)	28 (3)	7.1 (4.0 to 8.0)	346	32	346	32
USA	NHANESI	6586	1973	58 (11)	2847 (43)	1868 (28)	437 (6.6)	140 (24)	6.0 (1.2)	26 (5)	17.9 (3.5 to 20.9)	811	461	368	233
USA	RANCHO	1523	1985	67 (9)	613 (40)	230 (15)	76 (5.0)	135 (21)	5.7 (1.0)	25 (4)	15.0 (2.1 to 18.2)	171	144	118	85
USA	SHS	2643	1990	57 (8)	1077 (41)	1079 (41)	1022 (38.7)	126 (19)	5.1 (1.0)	30 (6)	12.5 (2.0 to 14.3)	318	129	281	92
USA	USPHS2	10715	1997	64 (8)	10715 (100)	507 (5)	25 (0.2)	128 (12)	5.3 (0.9)	25 (3)	10.9 (4.9 to 11.5)	311	259	305	254
USA	WHS	23244	1994	55 (7)	0 (0)	2686 (12)	727 (3.1)	127 (12)	5.5 (1.1)	26 (5)	19.1 (8.6 to 20.0)	409	525	241	262
TOTAL USA/Canada		85098		58 (10)	38348 (45)	18101 (21)	6441 (7.6)	130 (19)	5.5 (1.1)	27 (5)	12.4 (3.8 to 23.9)	5310	3564	3276	1985
Australia	AUSDIAB	7033	2000	55 (10)	3072 (44)	1080 (15)	336 (4.8)	131 (19)	5.8 (1.0)	27 (5)	5.0 (4.9 to 13.0)	65	36	65	36
Australia	DUBBO	1893	1989	67 (5)	810 (43)	317 (17)	124 (6.6)	146 (23)	6.6 (1.2)	26 (4)	14.1 (2.1 to 14.9)	253	180	181	129
Caribbean	PRHHP	6300	1967	54 (6)	6300 (100)	3284 (52)	631 (10.0)	132 (22)	5.3 (1.1)	25 (4)	8.3 (5.2 to 12.0)	211	84	205	73

Country	Cohort	No of participants	Median year of study recruitment	Ages mean(sd)	Men n (%)	Current smoker n (%)	History of diabetes n (%)	Systolic BP (mmHg) mean(sd)	Total cholesterol (mmol/L) mean(sd)	BMI (kg/m^2)	Median Follow-up (5th and 95th percentile)	MI or fatal CHD	Cerebro-vascular	MI or fatal CHD By 10yrs	Cerebro-vascular By 10yrs
Japan	FUNAGATA	963	1992	55 (11)	437 (45)	283 (29)	1 (0.1)	128 (18)	5.2 (1.0)	24 (3)	9.8 (5.3 to 10.2)	11	33	11	33
Japan	HISAYAMA	2451	1988	58 (10)	1042 (43)	332 (14)	198 (8.1)	132 (20)	5.4 (1.1)	23 (3)	14.0 (4.1 to 14.0)	61	194	47	134
Japan	IKNS	8039	1991	58 (10)	3299 (41)	1909 (24)	557 (6.9)	134 (19)	5.1 (0.9)	23 (3)	11.1 (5.1 to 18.6)	84	343	57	250
Japan	OSAKA	11231	1992	54 (8)	7605 (68)	4300 (38)	589 (5.2)	125 (18)	5.3 (0.9)	23 (3)	10.2 (3.9 to 18.8)	37	138	27	83
Japan	TOYAMA	3444	1996	48 (5)	2213 (64)	1301 (38)	194 (5.6)	121 (15)	5.4 (0.9)	23 (3)	12.7 (7.0 to 12.8)	31	45	22	34
Turkey	TARFS	2026	1990	53 (9)	992 (49)	593 (29)	132 (6.5)	133 (25)	4.9 (1.1)	28 (5)	9.8 (1.7 to 17.6)	124	41	94	31
TOTAL other countries		43380		55 (10)	25770 (59)	13399 (31)	2762 (6.4)	130 (20)	5.4 (1.0)	24 (4)	9.6 (3.9 to 18.6)	877	1094	709	803
TOTAL		376177		55 (9)	202962 (54)	115113 (31)	17947 (4.8)	134 (20)	5.9 (1.2)	26 (4)	11.5 (3.8 to 29.6)	26213	15552	12607	6726

Study abbreviations are listed in **Appendix section 2**

Table 1.2: Baseline characteristics and event summaries from studies used in external validation

Country	Cohort	No of participants	Median year of study recruitment	Ages mean(sd)	Men n (%)	Current smoker n (%)	History of diabetes n (%)	Systolic BP (mmHg) mean(sd)	Total cholesterol (mmol/L) mean(sd)	BMI (kg/m^2)	Median Follow-up (years) (5th and 95th percentile)	MI By 10yrs	Cerebro-Vascular By 10yrs
Australia	AAA[1]	7469	1996	71 (4)	7469 (100)	935 (13)	755 (10.1)	158 (21)	-	27 (4)	3.2 (1.5 to 4.4)	276	100
Australia	Busselton[1]	3610	1966	56 (10)	1782 (49)	1219 (34)	60 (2.3)	145 (25)	6.3 (1.2)	25 (4)	20.5 (4.4 to 35.5)	266	128
China	CISCH[1]	1324	1992	48 (6)	748 (56)	382 (29)	39 (3.0)	121 (17)	-	25 (3)	3.3 (3.1 to 3.5)	14	7
China	Capital Iron & Steel Company[1]	4300	1974	48 (6)	4300 (100)	3093 (72)	-	124 (19)	4.9 (1.0)	23 (3)	12.5 (4.5 to 18.5)	112	86
China	Fangshan[1]	635	1991	53 (10)	178 (28)	260 (41)	-	146 (29)	-	24 (3)	3.7 (3.6 to 3.7)	1	13
China	Huashan[1]	1558	1992	56 (10)	752 (48)	365 (23)	0 (0.0)	129 (21)	4.7 (0.9)	24 (3)	2.8 (0.0 to 3.0)	3	16
China	Seven Cities Cohorts[1]	7818	1987	56 (10)	3535 (45)	2819 (36)	88 (1.1)	131 (24)	5.0 (1.5)	23 (4)	2.7 (2.7 to 10.5)	0	234
Japan	Akabane[1]	1787	1985	54 (8)	781 (44)	503 (28)	25 (1.4)	124 (19)	5.0 (0.9)	22 (3)	11.0 (5.2 to 12.9)	60	33
Japan	Ohasama[1]	1921	1992	60 (10)	657 (34)	373 (19)	193 (10.0)	128 (17)	5.0 (0.9)	23 (3)	4.1 (3.8 to 4.4)	0	40
Japan	Shirakawa[1]	3785	1974	53 (8)	1699 (45)	1294 (34)	36 (1.0)	129 (22)	4.7 (0.9)	22 (3)	17.5 (8.6 to 19.5)	31	50
Japan	Hisayama[1]	1561	1961	55 (10)	690 (44)	677 (43)	-	134 (26)	4.1 (1.0)	22 (3)	22.6 (2.6 to 25.1)	38	140
New Zealand	Fletcher Challenge[1]	5524	1992	52 (10)	3895 (71)	1061 (19)	183 (3.3)	129 (18)	5.6 (1.1)	27 (4)	5.6 (3.6 to 6.4)	371	92
Singapore	Singapore Heart[1]	1078	1988	51 (9)	542 (50)	255 (24)	167 (15.5)	133 (23)	6.2 (1.2)	25 (4)	13.6 (3.1 to 15.8)	21	32
Singapore	Singapore NHS92[1]	1365	1992	51 (8)	636 (47)	227 (17)	252 (18.5)	127 (21)	5.7 (1.0)	24 (4)	6.2 (5.4 to 6.3)	23	32
TOTAL for cohorts of the APCSC		43735		57 (11)	27664 (63)	13463 (31)	1798 (5.0)	135 (24)	5.2 (1.3)	24 (4)	5.7 (2.5 to 23.5)	1216	1003
New Zealand	PREDICT-CVD[2]	254680	2013	55 (9)	134001 (54)	38506 (16)	28520 (11.4)	129 (17)	5.3 (1.0)	28.5 (6.1)	4.7 (1.5-7.5)	3,568	3,289
China	CMCS[3]	17167	1992	51 (8)	9161 (53)	4913 (29)	1481 (8.6)	126 (21)	4.9 (10)	24 (3)	14.2 (3.5 to 21.3)	522	1091
Iran	TLGS[4]	4921	1999-2005	53.3 (9.6)	2212 (45.0)	769 (15.6)	896 (18.2)	126.1 (20.9)	5.7 (1.2)	27.9 (4.6)	14.1 (6.4-14.9)	230	170
Thailand	HCUR[5]	330,985	2007	53.9 (10.6)	155974 (47.1)	44540 (13.5)	21049 (6.5)	120.2 (14.0)	4.1 (0.9)	22.8 (3.3)	5.82 (5.47-6.11)	2,736	3,673
United Kingdom	UKBiobank	444573	2009	57 (8)	198493 (45)	46274 (10)	19065 (4.3)	138 (19)	5.8 (1.1)	27 (5)	8.1 (6.7 to 9.4)	4628	3824

[1] Cohort of the Asia Pacific Cohorts Studies Collaboration. [2] PREDICT cardiovascular disease cohort, recruited from 2010 onwards. [3] China Multi-Provincial Cohort Study. [4] Tehran Lipids and Glucose Study. [5] Health Checks Ubon Ratchathani Study

Cohorts with fewer than 10 events for either MI or Stroke were excluded from validation of models involving that endpoint.

Table 1.3. Endpoint definitions used in model derivation and validation

	**MI/CHD**		**Stroke**	
	**ICD9**	**ICD10**	**ICD9**	**ICD10**
Definitions used in derivation of the risk models				
**ERFC**	fatal or non-fatal: 410,412<br>fatal: 411,414	fatal or non-fatal: I21-I23<br>fatal: I24-I25	430-434, 436-439	I60-I69
Definitions used by validation studies				
**APCSC**	fatal or non-fatal: 348.1,348.2, 348.9, 410,412<br>fatal: 411,414		430-438	
**PREDICT**	410-414.9	I20-I21.6, I21.9-I25.9	430-439.6	G45-G46.8, I60-I64, I64.1, I65-I69.998
**CMCS**	410,414	I21, I25	430-434, 436	I60-I64
**HCUR**	410-414.9	I20-I21.6, I21.9-I25.9	430-439.6	G45-G46.8, I60-I64, I64.1, I65-I69.998
**TLGS**		Deaths from IHD or sudden cardiac death (ICD10 codes I20-I25) and nonfatal myocardial infarction (ICD10 codes I21–22)		I60–I69
**UK Biobank**	410,412	I21-I23	430-434, 436-439	I60-I69

The endpoint used by the Global Burden of Disease study, and used in recalibration, is described in **Appendix 4**

Table 1.4: Summary of available country specific survey data from WHO-STEPS

GBD region	Country	Year of survey	Participants with data for non lab-based model	Participants with data for lab- based model	Survey type and setting*	Age at survey (yrs) mean (sd)	Sex = Male (%)	Diabetes = Yes (%)	Smoking = Yes (%)	BMI (kg/m^2) mean (sd)	SBP (mmHg) mean (sd)	Total cholesterol (mmol/l) mean (sd)	Median 10-year risk WHO lab-based model (%) (5th & 95th percentiles)	Laboratory based model; 10-yr risk >=20%	Median 10-year risk WHO non-lab model (%) (5th & 95th percentiles)	Non-laboratory based model; 10-yr risk >=20%
North Africa and Middle East	Algeria	2003	2077	2024	C-B	50 (7)	884 (43)	200 (10)	300 (14)	26 (5)	136 (20)	4.85 (0.96)	6.7 (2.8 to 20.5)	111 (5.5)	7.2 (3.0 to 19.2)	88 (4.2)
	Egypt	2011	2164	1072	N-B	51 (7)	818 (38)	497 (23)	431 (20)	32 (7)	141 (22)	5.04 (1.25)	10.0 (3.1 to 28.5)	180 (16.8)	9.6 (3.6 to 25.0)	242 (11.2)
	Iraq	2015	1658	1546	N-B	50 (7)	674 (41)	484 (30)	299 (18)	31 (7)	138 (20)	5.04 (1.25)	8.6 (2.9 to 27.2)	189 (12.2)	8.6 (3.5 to 23.8)	136 (8.2)
	Kuwait	2014	1320	998	N-B	49 (7)	496 (38)	384 (29)	188 (14)	32 (7)	127 (16)	5.21 (0.98)	7.7 (2.7 to 20.8)	59 (5.9)	7.0 (3.1 to 17.5)	36 (2.7)
	Lebanon	2017	1068	728	N-B	52 (7)	438 (41)	183 (17)	428 (40)	28 (5)	130 (17)	5.70 (1.12)	9.9 (3.2 to 26.7)	93 (12.8)	10.0 (3.6 to 22.4)	85 (8.0)
	Libya	2009	1736	1211	N-B	49 (7)	920 (53)	332 (19)	421 (24)	29 (6)	144 (23)	4.80 (1.00)	8.2 (2.8 to 30.3)	177 (14.6)	8.8 (3.4 to 26.6)	203 (11.7)
	Occupied Palestinian Territory	2010	3168	3079	N-B	51 (7)	1106 (35)	885 (28)	576 (18)	31 (7)	128 (19)	5.13 (1.08)	7.8 (2.6 to 23.6)	273 (8.9)	7.9 (3.0 to 19.9)	154 (4.9)
	Qatar	2012	1056	680	N-B	49 (7)	470 (45)	359 (34)	155 (15)	32 (7)	128 (20)	4.40 (0.94)	6.6 (2.3 to 23.3)	59 (8.7)	6.8 (2.9 to 20.7)	59 (5.6)
	Sudan	2017	2868	2473	S-B	49 (7)	1196 (42)	415 (14)	179 (6)	25 (6)	137 (21)	4.39 (1.11)	6.3 (2.5 to 18.6)	95 (3.8)	6.7 (2.8 to 18.3)	95 (3.3)
	Turkey	2017	2511	1419	N-B	52 (7)	970 (39)	516 (21)	717 (29)	31 (6)	128 (19)	4.57 (1.09)	8.7 (2.7 to 23.7)	126 (8.9)	9.1 (3.4 to 20.6)	143 (5.7)
Central Sub-Saharan Africa	Central African Republic	2010	1880	-	C-B	51 (7)	948 (50)	177 (9)	267 (14)	23 (5)	139 (25)	-	-	-	4.2 (1.2 to 13.1)	15 (0.8)
	Congo	2004	678	-	C-U	50 (7)	364 (54)	22 (3)	87 (13)	24 (5)	140 (24)	-	-	-	3.6 (1.2 to 12.6)	5 (0.7)
	DR Congo	2005	484	-	C-U	50 (7)	212 (44)	25 (5)	40 (8)	23 (5)	128 (23)	-	-	-	3.2 (1.0 to 10.3)	2 (0.4)
	Gabon	2009	1000	-	C-U	50 (7)	452 (45)	31 (3)	103 (10)	27 (6)	133 (21)	-	-	-	3.7 (1.2 to 11.5)	6 (0.6)
Eastern Sub-Saharan Africa	Comoros	2011	2538	874	N-B	50 (7)	780 (31)	146 (6)	221 (9)	26 (6)	136 (24)	4.73 (0.75)	3.6 (1.1 to 11.3)	2 (0.2)	3.2 (1.0 to 10.1)	9 (0.4)
	Eritrea	2010	2867	-	N-B	50 (7)	937 (33)	127 (4)	120 (4)	21 (4)	123 (20)	-	-	-	2.4 (0.9 to 7.2)	3 (0.1)
	Ethiopia	2006	2118	-	S-U	51 (7)	775 (37)	126 (6)	100 (5)	24 (5)	136 (25)	-	-	-	3.1 (1.1 to 10.8)	8 (0.4)
	Kenya	2015	1521	1232	N-B	50 (7)	637 (42)	84 (6)	166 (11)	24 (6)	134 (23)	4.15 (0.97)	3.0 (1.0 to 9.9)	3 (0.2)	3.1 (1.1 to 10.0)	6 (0.4)
	Madagascar	2005	2349	-	C-U	49 (7)	1172 (50)	21 (1)	466 (20)	21 (4)	135 (25)	-	-	-	3.2 (1.1 to 10.8)	14 (0.6)
	Malawi	2009	1600	-	N-B	51 (7)	519 (32)	21 (1)	237 (15)	23 (4)	142 (24)	-	-	-	3.6 (1.3 to 12.1)	10 (0.6)
	Mozambique	2005	1412	-	N-B	50 (7)	621 (44)	35 (2)	291 (21)	23 (5)	143 (26)	-	-	-	3.7 (1.2 to 12.4)	15 (1.1)
	Rwanda	2012	2432	1850	N-B	50 (7)	873 (36)	60 (2)	664 (27)	22 (4)	129 (20)	3.61 (0.80)	2.8 (1.0 to 9.2)	2 (0.1)	3.1 (1.1 to 10.3)	3 (0.1)
	Tanzania	2012	2890	1017	N-B	50 (7)	1428 (49)	137 (5)	471 (16)	23 (5)	138 (24)	4.69 (0.83)	3.8 (1.1 to 13.4)	14 (1.4)	3.5 (1.2 to 11.8)	27 (0.9)
	Uganda	2014	1176	931	N-B	49 (7)	478 (41)	39 (3)	166 (14)	23 (5)	134 (23)	3.95 (0.97)	2.7 (1.0 to 9.3)	0 (0.0)	2.9 (1.1 to 9.5)	1 (0.1)
	Zambia	2008	536	445	C-U	49 (7)	161 (30)	34 (6)	27 (5)	26 (6)	145 (27)	4.79 (1.00)	3.4 (1.2 to 11.7)	3 (0.7)	3.3 (1.2 to 11.7)	3 (0.6)
Southern Sub-Saharan Africa	Botswana	2014	1368	1104	N-B	51 (7)	416 (30)	116 (8)	201 (15)	26 (7)	138 (24)	4.27 (1.08)	3.7 (1.2 to 11.4)	10 (0.9)	3.8 (1.3 to 11.7)	6 (0.4)
	Lesotho	2012	1192	1018	N-B	52 (7)	376 (32)	76 (6)	176 (15)	27 (8)	139 (27)	3.88 (0.81)	3.9 (1.1 to 12.2)	7 (0.7)	4.4 (1.4 to 13.4)	12 (1.0)
	Swaziland	2014	1054	899	N-B	51 (7)	340 (32)	119 (11)	93 (9)	29 (8)	136 (24)	4.35 (1.05)	3.4 (1.1 to 11.9)	5 (0.6)	3.6 (1.3 to 11.3)	7 (0.7)
Western Sub-Saharan Africa	Benin	2015	1807	1660	N-B	49 (7)	930 (51)	164 (9)	139 (8)	24 (5)	136 (24)	4.25 (1.20)	2.9 (1.0 to 10.4)	10 (0.6)	2.8 (1.0 to 9.8)	4 (0.2)
	Burkina Faso	2013	1883	1162	N-B	50 (7)	982 (52)	56 (3)	184 (10)	22 (4)	127 (21)	3.63 (0.92)	2.4 (0.9 to 8.3)	3 (0.3)	2.5 (0.9 to 8.0)	5 (0.3)
	Cabo Verde	2007	978	521	N-B	49 (7)	340 (35)	72 (7)	111 (11)	25 (5)	141 (22)	4.38 (0.81)	3.2 (1.2 to 11.4)	5 (1.0)	3.2 (1.3 to 10.0)	1 (0.1)
	Cameroon	2003	2569	-	S-U	49 (7)	1113 (43)	156 (6)	231 (9)	27 (6)	132 (23)	-	-	-	2.8 (1.0 to 9.8)	11 (0.4)
	Chad	2008	798	-	C-U	50 (7)	425 (53)	36 (5)	87 (11)	26 (8)	133 (23)	-	-	-	3.1 (0.9 to 10.7)	4 (0.5)
	Cote d'Ivoire	2005	1267	-	S-B	50 (7)	623 (49)	29 (2)	224 (18)	25 (6)	140 (25)	-	-	-	3.6 (1.1 to 12.3)	11 (0.9)
	Gambia	2010	1450	-	N-B	49 (7)	822 (57)	23 (2)	256 (18)	25 (5)	139 (23)	-	-	-	3.4 (1.2 to 11.8)	10 (0.7)
	Ghana	2006	1208	812	C-U	50 (7)	401 (33)	95 (8)	41 (3)	28 (7)	142 (25)	4.87 (0.95)	3.4 (1.1 to 11.9)	4 (0.5)	3.3 (1.1 to 11.6)	9 (0.7)
	Guinea	2009	833	825	S-B	51 (7)	460 (55)	69 (8)	137 (16)	24 (6)	146 (28)	4.41 (0.64)	4.0 (1.1 to 14.6)	19 (2.3)	4.0 (1.1 to 14.3)	14 (1.7)
	Liberia	2011	917	-	N-B	50 (7)	421 (46)	77 (8)	109 (12)	28 (10)	137 (26)	-	-	-	3.2 (1.1 to 12.7)	15 (1.6)
	Mali	2007	812	-	C-B	52 (8)	306 (38)	55 (7)	132 (16)	26 (7)	129 (25)	-	-	-	3.3 (1.0 to 10.9)	5 (0.6)

GBD region	Country	Year of survey	Participants with data for non lab-based model	Participants with data for lab- based model	Survey type and setting*	Age at survey (yrs) mean (sd)	Sex = Male (%)	Diabetes = Yes (%)	Smoking = Yes (%)	BMI (kg/m^2) mean (sd)	SBP (mmHg) mean (sd)	Total cholesterol (mmol/l) mean (sd)	Median 10-year risk WHO lab-based model (%) (5th & 95th percentiles)	Laboratory based model; 10-yr risk >=20%	Median 10-year risk WHO non-lab model (%) (5th & 95th percentiles)	Non-laboratory based model; 10-yr risk >=20%
Western Sub-Saharan Africa, continued.	Niger	2007	1216	-	N-B	50 (7)	715 (59)	234 (19)	54 (4)	22 (5)	143 (25)	-	-	-	3.1 (1.1 to 10.4)	5 (0.4)
	Sao Tome and Principe	2009	1018	999	N-B	50 (7)	395 (39)	26 (3)	97 (10)	26 (6)	144 (26)	4.08 (0.64)	3.1 (1.1 to 11.0)	2 (0.2)	3.5 (1.2 to 11.8)	5 (0.5)
	Sierra Leone	2009	2005	-	N-B	50 (7)	1064 (53)	22 (1)	536 (27)	24 (7)	139 (24)	-	-	-	3.7 (1.1 to 12.7)	12 (0.6)
	Togo	2010	1386	-	N-B	49 (7)	721 (52)	53 (4)	206 (15)	23 (5)	130 (25)	-	-	-	2.8 (0.9 to 9.4)	5 (0.4)
Southern Latin America	Uruguay	2006	611	448	N-B	52 (7)	176 (29)	41 (7)	195 (32)	28 (6)	134 (22)	5.01 (1.01)	3.5 (0.6 to 16.3)	11 (2.5)	3.6 (0.8 to 16.2)	11 (1.8)
Caribbean	Barbados	2007	185	178	N-B	52 (7)	93 (50)	21 (11)	7 (4)	29 (8)	128 (17)	5.06 (0.99)	3.5 (1.2 to 9.9)	1 (0.6)	3.6 (1.4 to 10.0)	1 (0.5)
	Dominica	2007	526	70	N-B	51 (7)	248 (47)	76 (15)	43 (8)	27 (7)	142 (24)	5.35 (1.50)	4.3 (1.6 to 16.8)	2 (2.9)	3.9 (1.3 to 11.6)	3 (0.6)
	Grenada	2010	657	-	N-B	51 (7)	256 (39)	-	123 (19)	28 (6)	138 (22)	-	-	-	4.4 (1.4 to 11.8)	0 (0.0)
	Saint Lucia	2012	820	-	N-B	50 (7)	298 (36)	85 (10)	90 (11)	31 (7)	132 (20)	-	-	-	3.4 (1.4 to 10.1)	4 (0.5)
	Trinidad and Tobago	2011	1333	289	N-B	52 (7)	502 (38)	293 (22)	233 (17)	29 (7)	136 (22)	5.29 (1.51)	4.6 (1.5 to 15.9)	8 (2.8)	4.4 (1.5 to 12.2)	13 (1.0)
South Asia	Bhutan	2014	1242	1167	N-B	50 (7)	517 (42)	78 (6)	47 (4)	25 (4)	135 (22)	4.12 (0.92)	3.2 (1.3 to 9.5)	2 (0.2)	3.7 (1.6 to 10.2)	2 (0.2)
	Nepal	2013	1887	1717	N-B	50 (7)	686 (36)	156 (8)	478 (25)	23 (4)	135 (21)	4.52 (1.03)	4.0 (1.2 to 12.9)	11 (0.6)	4.3 (1.5 to 12.2)	9 (0.5)
	Pakistan	2014	2282	-	S-B	49 (7)	1108 (49)	161 (7)	439 (19)	25 (6)	136 (20)	-	-	-	4.0 (1.5 to 11.7)	5 (0.2)
Southeast Asia	Cambodia	2010	3181	-	N-B	51 (7)	1107 (35)	152 (5)	809 (25)	22 (4)	121 (19)	-	-	-	3.0 (1.0 to 9.4)	7 (0.2)
	Lao PDR	2013	1192	1130	N-B	50 (7)	500 (42)	112 (9)	396 (33)	23 (5)	124 (21)	4.57 (1.11)	3.2 (1.0 to 10.2)	12 (1.1)	3.3 (1.1 to 9.9)	6 (0.5)
	Maldives	2011	696	204	C-B	52 (7)	238 (34)	112 (16)	104 (15)	27 (5)	138 (24)	4.93 (1.10)	4.4 (1.1 to 14.0)	4 (2.0)	4.6 (1.2 to 13.1)	7 (1.0)
	Myanmar	2014	5320	5242	S-B	51 (7)	1840 (35)	643 (12)	1337 (25)	23 (5)	132 (23)	4.88 (1.04)	3.9 (1.1 to 12.8)	53 (1.0)	3.8 (1.2 to 12.0)	38 (0.7)
	Seychelles	2004	816	804	N-B	52 (7)	374 (46)	131 (16)	154 (19)	28 (6)	137 (21)	5.66 (1.29)	4.8 (1.3 to 16.8)	25 (3.1)	4.8 (1.5 to 13.5)	9 (1.1)
	Sri Lanka	2014	2597	2139	N-B	51 (7)	1052 (41)	499 (19)	375 (14)	24 (5)	132 (21)	4.38 (1.13)	3.8 (1.1 to 12.3)	20 (0.9)	3.6 (1.2 to 10.8)	7 (0.3)
	Timor-Leste	2014	1105	951	N-B	50 (8)	521 (47)	39 (4)	415 (38)	21 (4)	130 (21)	4.04 (0.95)	3.3 (1.1 to 11.4)	2 (0.2)	3.6 (1.2 to 11.5)	3 (0.3)
	Viet Nam	2015	1659	1623	N-B	51 (7)	706 (43)	122 (7)	421 (25)	23 (3)	127 (21)	4.83 (1.20)	3.5 (1.0 to 11.9)	13 (0.8)	3.6 (1.1 to 11.0)	6 (0.4)
Central Asia	Armenia	2016	1059	851	N-B	53 (7)	285 (27)	118 (11)	177 (17)	29 (6)	141 (25)	4.86 (1.08)	5.1 (0.9 to 26.8)	75 (8.8)	5.5 (1.1 to 24.1)	86 (8.1)
	Georgia	2016	2321	1818	N-B	53 (7)	679 (29)	265 (11)	512 (22)	30 (7)	137 (24)	4.87 (1.20)	5.1 (1.0 to 25.8)	157 (8.6)	5.9 (1.2 to 26.5)	195 (8.4)
	Kyrgyzstan	2013	1598	1543	N-B	51 (7)	592 (37)	173 (11)	306 (19)	29 (6)	144 (24)	4.66 (0.97)	5.0 (1.0 to 24.7)	121 (7.8)	5.6 (1.2 to 24.6)	133 (8.3)
	Mongolia	2013	2115	950	N-B	49 (6)	927 (44)	220 (10)	498 (24)	28 (5)	136 (22)	5.21 (0.77)	4.4 (1.0 to 21.8)	56 (5.9)	4.2 (1.0 to 20.9)	114 (5.4)
	Tajikistan	2016	1236	1129	N-B	50 (7)	535 (43)	155 (13)	71 (6)	28 (6)	143 (23)	4.39 (0.96)	3.8 (1.0 to 21.5)	64 (5.7)	4.2 (1.2 to 20.8)	68 (5.5)
	Turkmenistan	2013	2174	1453	N-B	50 (7)	840 (39)	366 (17)	196 (9)	28 (6)	135 (19)	4.90 (1.57)	3.8 (0.9 to 22.8)	97 (6.7)	3.6 (1.1 to 15.9)	65 (3.0)
	Uzbekistan	2014	1572	1485	N-B	50 (7)	634 (40)	148 (9)	223 (14)	29 (6)	138 (22)	5.06 (0.97)	3.9 (1.0 to 20.4)	78 (5.3)	4.2 (1.1 to 20.2)	79 (5.0)
Eastern Europe	Belarus	2016	2992	2880	N-B	52 (7)	1234 (41)	283 (9)	790 (26)	29 (5)	143 (22)	5.08 (1.02)	8.9 (1.8 to 26.1)	363 (12.6)	9.3 (2.0 to 25.7)	357 (11.9)
	Moldova	2013	2605	2105	N-B	53 (7)	1008 (39)	371 (14)	485 (19)	29 (6)	144 (24)	4.87 (0.95)	9.1 (1.7 to 28.9)	290 (13.8)	9.6 (2.0 to 26.4)	333 (12.8)
Oceania	American Samoa	2004	1131	838	N-B	50 (7)	545 (48)	413 (37)	410 (36)	35 (7)	138 (20)	4.92 (0.77)	6.6 (2.0 to 17.9)	30 (3.6)	6.8 (2.4 to 17.3)	33 (2.9)
	Fiji	2011	1586	-	N-B	51 (7)	716 (45)	394 (25)	412 (26)	29 (7)	139 (23)	-	-	-	6.0 (2.2 to 15.6)	25 (1.6)
	Kiribati	2004	757	474	N-B	50 (7)	337 (45)	174 (23)	478 (63)	31 (7)	126 (18)	4.73 (0.68)	5.8 (2.2 to 14.8)	7 (1.5)	6.3 (2.4 to 14.4)	6 (0.8)
	Marshall Islands	2002	588	365	N-B	49 (6)	228 (39)	239 (41)	90 (15)	31 (7)	123 (21)	5.08 (1.33)	4.9 (1.6 to 13.5)	2 (0.5)	4.1 (1.6 to 11.1)	2 (0.3)
	Micronesia (Federated States of)	2009	851	338	S-B	51 (7)	347 (41)	290 (34)	152 (18)	33 (11)	134 (22)	4.30 (0.72)	4.9 (1.8 to 15.2)	8 (2.4)	5.8 (2.1 to 16.1)	16 (1.9)
	Papua New Guinea	2007	998	-	N-B	51 (7)	527 (53)	67 (7)	392 (39)	25 (5)	125 (17)	-	-	-	5.4 (2.0 to 11.6)	3 (0.3)
	Samoa	2013	747	727	N-B	51 (7)	291 (39)	242 (32)	178 (24)	34 (7)	134 (22)	4.60 (0.81)	5.5 (1.5 to 16.2)	13 (1.8)	6.0 (2.0 to 15.3)	11 (1.5)
	Solomon Islands	2015	829	737	N-B	49 (7)	408 (49)	94 (11)	267 (32)	28 (7)	132 (23)	4.76 (1.11)	4.6 (1.5 to 12.7)	9 (1.2)	5.3 (1.9 to 14.4)	10 (1.2)
	Tonga	2011	1419	-	N-B	49 (7)	558 (39)	386 (27)	348 (25)	34 (8)	135 (19)	-	-	-	5.6 (2.2 to 15.0)	13 (0.9)
	Vanuatu	2011	2188	2088	N-B	50 (7)	1168 (53)	310 (14)	425 (19)	27 (6)	139 (22)	5.05 (0.84)	5.2 (1.8 to 15.5)	35 (1.7)	5.4 (2.1 to 14.8)	33 (1.5)

*N, S and C imply nationally, sub-nationally and community representative surveys respectively; U, R and B imply urban, rural or both settings are covered.

Table 1.5: Summary of available data from the Emerging Risk Factors Collaboration used in WHO risk model derivation, by sex

	**Men**	**Women**
**Study level characteristics**		
No. of studies	80	62
Year of recruitment*	1960-2008	1960-2013
**Baseline characteristics**		
Total participants	202,962	173,215
Age (years) at baseline survey	53(48, 60)	55 (49, 63)
Systolic blood pressure (mmHg)	132 (120, 146)	130 (118, 145)
Total cholesterol (mmol/l)	5.7 (5,0, 6.5)	5.9 (5.2, 6.7)
Current smoking	76943 (38)	38170 (22)
History of diabetes	9939 (4.9)	8008 (4.6)
Body mass index (kg/m^2)~	25.6 (23.5 28.0)	25.3 (22.8, 28.6)
**Cardiovascular outcomes**$		
Fatal/non-fatal MI or CHD death†	18987	7226
Fatal/non-fatal stroke‡	8870	6682
Follow up to first CVD event (years) median (5th, 95th percentiles)	10.3 (3.4, 30.4)	13.1 (4.4, 27.0)

Data are n (%), or median (25th, 75th percentile), unless otherwise indicated. Data are from a total of 85 cohorts with 376,177 participants

~Percentage of individuals in WHO defined BMI categories of <18.5, 18.5-24.9, 25-29.9, 30-34.9 and >40 were: 1.3, 43.2, 40.5, 11.6, 2.6 and 0.8 respectively

*41 cohorts (including 47% of total participants) had median year of study baseline <1990, 44 cohorts (including 53% of total participants) had median year of study baseline ≥1990

$Specific ICD codes are given for each endpoint in **Table 1.3.**

† Number of non-fatal or fatal MI events occurring during first 10 years of follow-up: 9456 in men, and 3151 in women.

‡ Number of fatal or non-fatal stroke events during first 10 years of follow-up: 3722 in men, and 3004 in women.

Table 1.6: Log hazard ratios, standard errors and heterogeneity statistics for the laboratory and non-laboratory based WHO risk models

	Men					Women				
	log HR	se logHR	I^2	P-value for heterogeneity by: region*	P-value for heterogeneity by: calendar year$	Log HR	se logHR	I^2	P-value for heterogeneity by: region*	P-value for heterogeneity by: calendar year$
**Laboratory-based model: Fatal/non-fatal MI or CHD death**										
Age at baseline per year	0.0719227	0.0023323	3% (0%, 28%)	0.013	0.247	0.1020713	0.0039237	24% (0%, 44%)	0.015	0.851
Total cholesterol per 1 mmol/L	0.2284944	0.0087117	13% (0%, 41%)	0.214	0.723	0.2050377	0.0132977	12% (0%, 35%)	0.955	0.876
Systolic blood pressure per 1 mmHg	0.0132183	0.0005011	4% (0%, 30%)	0.010	0.583	0.015823	0.0008139	22% (0%, 42%)	0.437	0.627
History of diabetes	0.6410114	0.0377504	0% (0%, 35%)	0.511	0.709	1.070358	0.0592361	0% (0%, 28%)	0.965	0.135
Current smoking	0.5638109	0.0239946	35% (6%, 55%)	0.277	0.041	1.053223	0.0414629	28% (2%, 46%)	0.366	0.622
T. cholesterol interaction with age‡	-0.0045806	0.0008194	35% (6%, 56%)	0.467	0.205	-0.0051932	0.0012386	0% (0%, 28%)	0.908	0.687
SBP interaction with age‡	-0.0001576	0.000051	0% (0%, 35%)	0.409	0.547	-0.0001378	0.0000805	0% (0%, 28%)	0.888	0.571
Diabetes interaction with age‡	-0.0124966	0.0037293	0% (0%, 35%)	0.520	0.168	-0.0234174	0.0058091	0% (0%, 28%)	0.932	0.088
Smoking interaction with age‡	-0.0182545	0.0024031	26% (0%, 49%)	0.789	0.505	-0.0332666	0.0042843	16% (0%, 38%)	0.150	0.532
**Laboratory-based model: Fatal/non-fatal stroke**										
Age at baseline per year	0.0986578	0.0035568	3% (0%, 28%)	0.478	0.540	0.1056632	0.0038126	0% (0%, 31%)	0.868	0.510
Total cholesterol per 1 mmol/L	0.029526	0.0160403	13% (0%, 41%)	0.749	0.587	0.0257782	0.0183081	16% (0%, 40%)	0.357	0.914
Systolic blood pressure per 1 mmHg	0.0222629	0.0007668	4% (0%, 30%)	0.277	0.348	0.0206278	0.0008984	26% (0%, 47%)	0.122	0.406
History of diabetes	0.6268712	0.0580946	0% (0%, 35%)	0.016	0.429	0.8581998	0.0689703	25% (0%, 46%)	0.263	0.188
Current smoking	0.4981217	0.0373988	35% (6%, 55%)	0.066	0.103	0.7443627	0.0468753	16% (0%, 40%)	0.846	0.628
T. cholesterol interaction with age‡	0.00142	0.0015228	35% (6%, 56%)	0.295	0.870	-0.0021387	0.0017473	0% (0%, 31%)	0.652	0.769
SBP interaction with age‡	-0.0004147	0.0000745	0% (0%, 35%)	0.015	0.780	-0.0004897	0.0000862	25% (0%, 46%)	0.769	0.160
Diabetes interaction with age‡	-0.026302	0.0056366	0% (0%, 35%)	0.502	0.835	-0.0209826	0.0067253	7% (0%, 33%)	0.743	0.153
Smoking interaction with age‡	-0.0150561	0.0036925	26% (0%, 49%)	0.562	0.446	-0.0200822	0.0047554	10% (0%, 35%)	0.325	0.867
**Non-laboratory-based model: Fatal/non-fatal MI or CHD death**										
Age at baseline per year	0.073593	0.002364	0% (0%, 35%)	0.003	0.164	0.1049418	0.0038628	8% (0%, 32%)	0.019	0.548
BMI per 1 kg/m^2	0.0337219	0.0029849	0% (0%, 35%)	0.234	0.360	0.0257616	0.0037581	0% (0%, 28%)	0.466	0.088
Systolic blood pressure per 1 mmHg	0.0133937	0.0005155	3% (0%, 29%)	0.060	0.494	0.016726	0.0008287	16% (0%, 38%)	0.099	0.465
Current smoking	0.5954767	0.0245956	36% (7%, 56%)	0.164	0.088	1.093132	0.0419601	38% (18%, 54%)	0.571	0.360
BMI interaction with age‡	-0.0010432	0.0002944	27% (0%, 50%)	0.864	0.176	-0.0006537	0.0003743	4% (0%, 28%)	0.398	0.578
SBP interaction with age‡	-0.0001837	0.000052	0% (0%, 35%)	0.106	0.279	-0.0001966	0.0000798	0% (0%, 28%)	0.856	0.653
Smoking interaction with age‡	-0.0200831	0.0024515	24% (0%, 48%)	0.958	0.635	-0.0343739	0.00426	13% (0%, 36%)	0.220	0.499
**Non-laboratory-based model: Fatal/non-fatal stroke**										
Age at baseline per year	0.097674	0.0035366	32% (1%, 53%)	0.869	0.563	0.1046105	0.0037452	9% (0%, 34%)	0.945	0.306
BMI per 1 kg/m^2	0.0159518	0.0047759	14% (0%, 41%)	0.019	0.528	0.0036406	0.0043984	8% (0%, 33%)	0.335	0.162
Systolic blood pressure per 1 mmHg	0.0227294	0.0007844	9% (0%, 36%)	0.263	0.246	0.0216741	0.0009094	29% (2%, 49%)	0.117	0.808
Current smoking	0.4999862	0.0381793	47% (26%, 63%)	0.057	0.086	0.7399405	0.0474139	14% (0%, 39%)	0.944	0.817
BMI interaction with age‡	-0.0003516	0.000456	23% (0%, 47%)	0.672	0.471	-0.0000129	0.0004273	0% (0%, 31%)	0.217	0.195
SBP interaction with age‡	-0.0004374	0.0000752	0% (0%, 34%)	0.067	0.839	-0.0005311	0.0000857	25% (0%, 46%)	0.901	0.236
Smoking interaction with age‡	-0.0153895	0.0037523	22% (0%, 47%)	0.662	0.347	-0.0203997	0.0047773	6% (0%, 32%)	0.328	0.614

* By region implies ERFC studies from America/Canada vs Western Europe vs other. $ By calendar year implies before and after 1990.
‡ age implies age at baseline. Age was centred at 60 years, SBP at 120mmHg, total cholesterol at 6mmol/l and BMI at $25kg/m^2$
WHO: World Health Organisation

Table 1.7: Ability of the risk models to discriminate: internal and external validation

	C-index (95% CI)							
	WHO risk model tested in original ERFC data[1]		APCSC model tested in APCSC data[2]		WHO risk model tested in APCSC data[2]		Difference between APCSC and WHO models in APCSC data[3]	
	Men	Women	Men	Women	Men	Women	Men	Women
**Laboratory-based models**								
Fatal /non-fatal MI or CHD death	0.6890 (0.6839, 0.6941)	0.7570 (0.7492, 0.7648)	0.7230 (0.7016, 0.7443)	0.7376 (0.7058, 0.7693)	0.7155 (0.6935, 0.7375)	0.7358 (0.7037, 0.7678)	-0.0075 (-0.0143, -0.0006)	-0.0018 (-0.0085, 0.0048)
Fatal/non-fatal stroke	0.7265 (0.7186, 0.7345)	0.7442 (0.7357, 0.7527)	0.7920 (0.7676, 0.8163)	0.7882 (0.7621, 0.8143)	0.7883 (0.7634, 0.8131)	0.7842 (0.7583, 0.8101)	-0.0037 (-0.0091, 0.0017)	-0.0044 (-0.0106, 0.0027)
**Non-laboratory based models**								
Fatal/non-fatal MI or CHD death	0.6660 (0.6606, 0.6715)	0.7382 (0.7301, 0.7463)	0.6688 (0.6529, 0.6847)	0.7404 (0.7127, 0.7681)	0.6628 (0.6465, 0.6791)	0.7356 (0.7077, 0.7636)	-0.0060 (-0.0126, 0.0006)	-0.0048 (-0.0111, 0.0016)
Fatal/non-fatal stroke	0.7233 (0.7152, 0.7315)	0.7367 (0.7282, 0.7453)	0.7579 (0.7405, 0.7753)	0.7944 (0.7735, 0.8152)	0.7543 (0.7364, 0.7722)	0.7904 (0.7694, 0.8114)	-0.0036 (-0.0079, 0.0006)	-0.0040 (-0.0085, 0.0006)

[1] Calculated using an internal/eternal validation approach in which each study was in turn left out of the model fit and used in validation. C-index shown is the results of pooling the C-index from each external study. Comprison of this to the C-index calculated for remaining studies revealed no evidence for optimism in pooled C-index estimates (p>0.999 for all models).
[2] Calculated by pooling the within study C-index values weighting by the number of contributing events.
[3] Calculated by pooling the within study differences in C-index values obtained using the two models, weighting by the number of contributing events.
ERFC: Emerging Risk Factors Collaboration
APCSC: Asia Pacific Cohort Studies Collaboration

Table 1.8: Comparison of Hazard Ratios from the risk models obtained using ERFC vs APCSC data

	Men				Women			
	ERFC		APCSC		ERFC		APCSC	
	HR(95% CI)	p-value	HR(95% CI)	p-value	HR(95% CI)	p-value	HR(95% CI)	p-value
**Laboratory-based model: Fatal/non-fatal MI or CHD death**								
Age at baseline per 5 years	1.43 (1.40, 1.46)	<0.001	1.52 (1.42, 1.64)	<0.001	1.66 (1.60, 1.73)	<0.001	1.42 (1.28, 1.57)	<0.001
Total cholesterol per 1mmol/L	1.26 (1.24, 1.28)	<0.001	1.21 (1.11, 1.31)	<0.001	1.23 (1.20, 1.26)	<0.001	1.14 (1.01, 1.29)	0.034
SBP per 20mmHg	1.30 (1.28, 1.33)	<0.001	1.25 (1.15, 1.37)	<0.001	1.38 (1.34, 1.42)	<0.001	1.26 (1.11, 1.42)	<0.001
History of diabetes	1.89 (1.75, 2.04)	<0.001	1.56 (1.09, 2.22)	0.015	2.91 (2.59, 3.27)	<0.001	2.23 (1.20, 4.13)	0.011
Current smoking	1.76 (1.68, 1.85)	<0.001	1.24 (1.01, 1.54)	0.042	2.83 (2.61, 3.08)	<0.001	1.70 (1.15, 2.51)	0.008
T. cholesterol interaction with age‡	0.98 (0.97, 0.99)	<0.001	0.99 (0.95, 1.02)	0.440	0.98 (0.96, 0.99)	<0.001	0.99 (0.93, 1.04)	0.616
SBP interaction with age‡	0.99 (0.98, 1.00)	0.004	0.91 (0.88, 0.95)	<0.001	0.99 (0.97, 1.00)	0.104	0.99 (0.95, 1.04)	0.811
Diabetes interaction with age‡	0.94 (0.91, 0.98)	0.001	0.93 (0.78, 1.12)	0.446	0.89 (0.84, 0.94)	<0.001	1.04 (0.78, 1.39)	0.787
Smoking interaction with age‡	0.91 (0.89, 0.94)	<0.001	0.94 (0.85, 1.04)	0.206	0.84 (0.81, 0.88)	<0.001	1.08 (0.90, 1.30)	0.416
**Laboratory-based model: Fatal/non-fatal stroke**								
Age at baseline per 5 years	1.64 (1.58, 1.70)	<0.001	1.57 (1.40, 1.76)	<0.001	1.70 (1.64, 1.76)	<0.001	1.60 (1.42, 1.81)	<0.001
Total cholesterol per 1mmol/L	1.03 (1.00, 1.06)	0.063	1.05 (0.95, 1.16)	0.369	1.03 (0.99, 1.06)	0.145	1.11 (1.00, 1.23)	0.052
SBP per 20mmHg	1.56 (1.52, 1.61)	<0.001	1.73 (1.59, 1.89)	<0.001	1.51 (1.46, 1.56)	<0.001	1.62 (1.48, 1.77)	0.000
History of diabetes	1.88 (1.68, 2.11)	<0.001	1.90 (1.25, 2.88)	0.003	2.35 (2.06, 2.70)	<0.001	2.49 (1.45, 4.26)	0.001
Current smoking	1.65 (1.53, 1.77)	<0.001	1.38 (1.08, 1.76)	0.010	2.11 (1.92, 2.31)	<0.001	1.72 (1.21, 2.45)	0.003
T. cholesterol interaction with age‡	1.01 (0.99, 1.02)	0.377	0.99 (0.94, 1.04)	0.677	0.99 (0.97, 1.01)	0.195	1.02 (0.97, 1.08)	0.404
SBP interaction with age‡	0.96 (0.95, 0.97)	<0.001	0.91 (0.87, 0.96)	<0.001	0.95 (0.94, 0.97)	<0.001	0.92 (0.88, 0.97)	0.001
Diabetes interaction with age‡	0.88 (0.83, 0.92)	<0.001	1.07 (0.94, 1.21)	0.318	0.90 (0.84, 0.96)	0.002	0.88 (0.73, 1.07)	0.211
Smoking interaction with age‡	0.93 (0.90, 0.96)	<0.001	1.02 (0.82, 1.28)	0.846	0.91 (0.87, 0.95)	<0.001	0.96 (0.72, 1.27)	0.778
**Non-laboratory-based model: Fatal/non-fatal MI or CHD death**								
Age at baseline per 5 years	1.44 (1.41, 1.48)	<0.001	1.54 (1.45, 1.63)	<0.001	1.69 (1.62, 1.75)	<0.001	1.52 (1.38, 1.66)	<0.001
BMI per 5KG/M^2	1.18 (1.15, 1.22)	<0.001	1.17 (1.07, 1.28)	<0.001	1.14 (1.10, 1.18)	<0.001	1.20 (1.03, 1.39)	0.017
SBP per 20mmHg	1.31 (1.28, 1.33)	<0.001	1.30 (1.22, 1.39)	<0.001	1.40 (1.36, 1.45)	<0.001	1.21 (1.08, 1.35)	0.001
Current smoking	1.81 (1.73, 1.90)	<0.001	1.27 (1.08, 1.48)	0.003	2.94 (2.71, 3.20)	<0.001	1.93 (1.38, 2.70)	<0.001
BMI interaction with age‡	0.97 (0.96, 0.99)	<0.001	0.96 (0.92, 0.99)	0.017	0.98 (0.97, 1.00)	0.100	1.02 (0.95, 1.09)	0.633
SBP interaction with age‡	0.98 (0.97, 0.99)	0.001	0.92 (0.90, 0.95)	<0.001	0.98 (0.97, 1.00)	0.016	0.98 (0.94, 1.03)	0.412
Smoking interaction with age‡	0.90 (0.88, 0.93)	<0.001	0.93 (0.86, 0.99)	0.030	0.84 (0.80, 0.87)	<0.001	1.06 (0.91, 1.25)	0.446
**Non-laboratory-based model: Fatal/non-fatal stroke**								
Age at baseline per 5 years	1.63 (1.57, 1.69)	<0.001	1.68 (1.55, 1.82)	<0.001	1.69 (1.63, 1.75)	<0.001	1.65 (1.50, 1.82)	<0.001
BMI per 5KG/M^2	1.08 (1.03, 1.13)	0.001	1.12 (0.99, 1.27)	0.061	1.02 (0.98, 1.06)	0.409	1.01 (0.87, 1.18)	0.865
SBP per 20mmHg	1.58 (1.53, 1.63)	<0.001	1.67 (1.57, 1.78)	<0.001	1.54 (1.49, 1.60)	<0.001	1.65 (1.53, 1.79)	<0.001
Current smoking	1.65 (1.53, 1.78)	<0.001	1.27 (1.06, 1.53)	0.009	2.10 (1.91, 2.31)	<0.001	1.47 (1.10, 1.97)	0.010
BMI interaction with age‡	0.99 (0.97, 1.01)	0.443	0.99 (0.94, 1.04)	0.720	1.00 (0.98, 1.02)	0.899	1.04 (0.97, 1.12)	0.276
SBP interaction with age‡	0.96 (0.94, 0.97)	<0.001	0.89 (0.86, 0.92)	<0.001	0.95(0.93,0.96)	<0.001	0.92 (0.88, 0.95)	<0.001
Smoking interaction with age‡	0.93 (0.89, 0.96)	<0.001	1.04 (0.96, 1.14)	0.352	0.91 (0.86, 0.95)	<0.001	0.91 (0.78, 1.07)	0.259

ERFC: Emerging Risk Factors Collaboration; APCSC: Asia Pacific Cohort Studies Collaboration

Figure 1.1: Data selection process for the ERFC datasets used in WHO risk model derivation

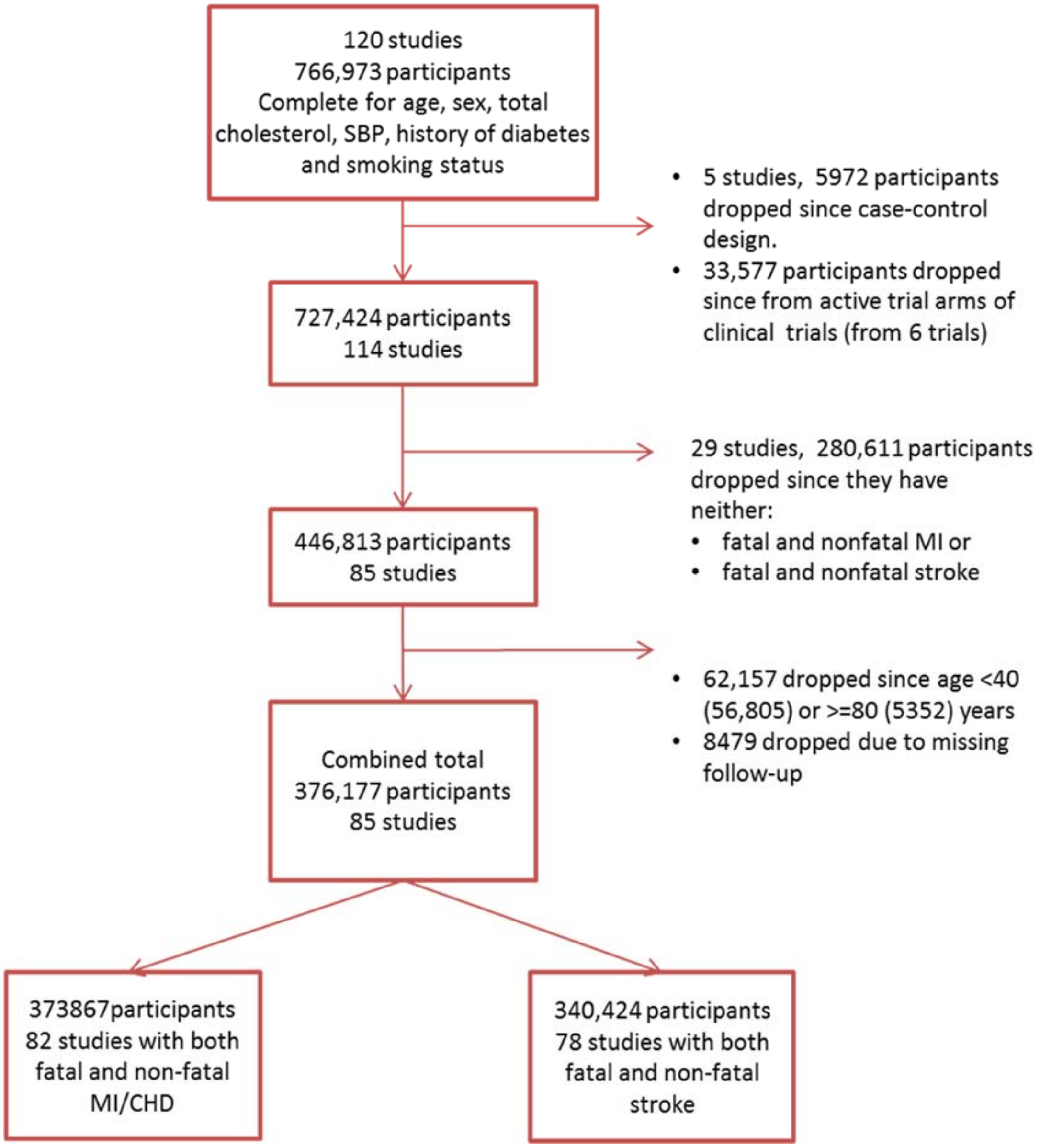

Figure 1.2:Comparison of CHD, Stroke and CVD 10-year risk estimates, with and without considering CHD and Stroke as competing events

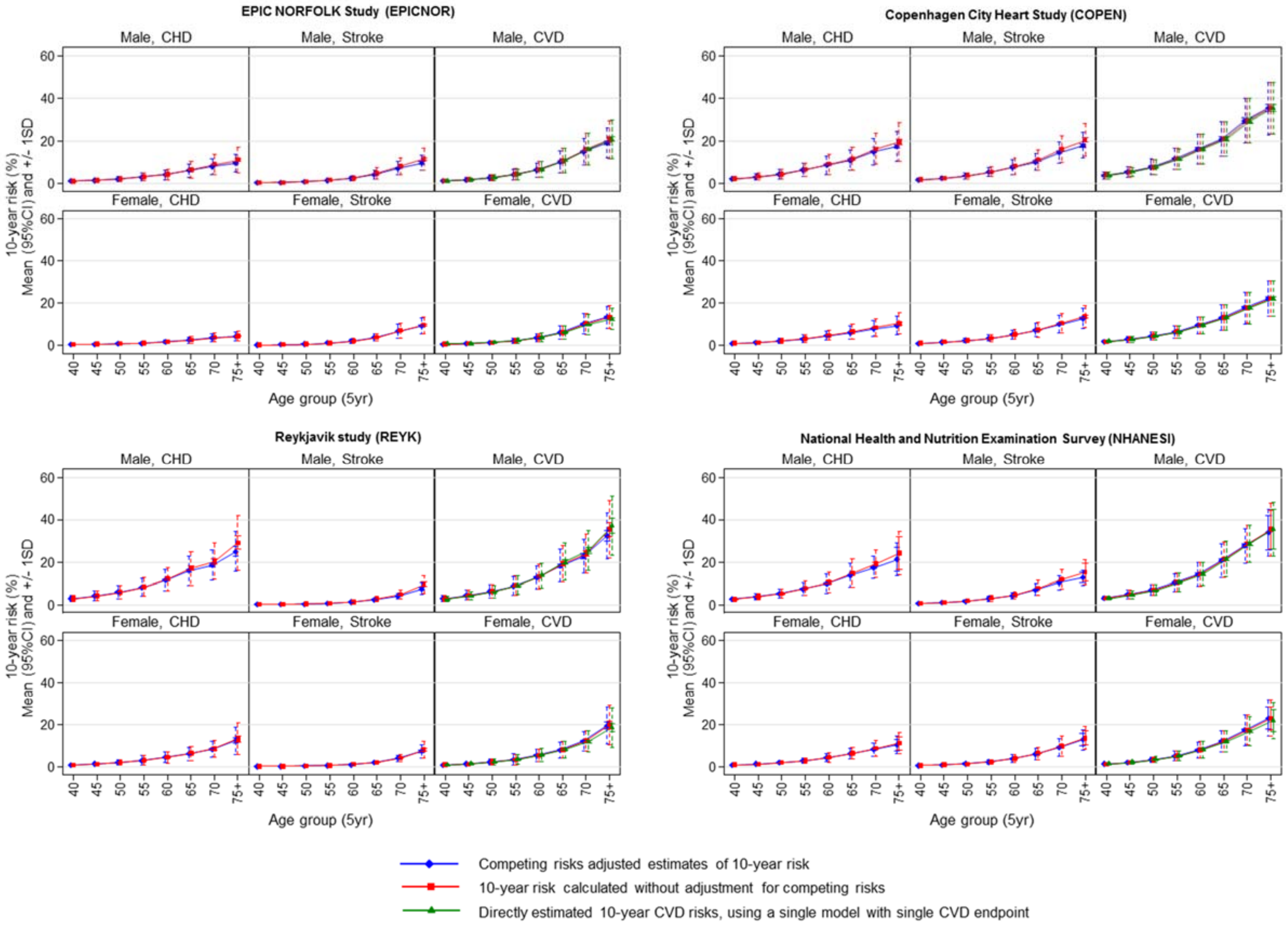

For competing risk adjusted 10-year risks: the CHD model is adjusted for competing stroke events, the stroke model for competing CHD events and the CVD model is the combination of the resulting independent (or competing risk adjusted) 10-year risks using the combination $p_{cvd} = 1 - (1 - p_{MI}) * (1 - p_{stroke})$. A similar combination of unadjusted CHD and Stroke 10-year risks is used to give the 10-year risk of CVD without adjustment for competing risks.

Figure 1.3: Process for recalibration of the WHO risk models for each region and sex

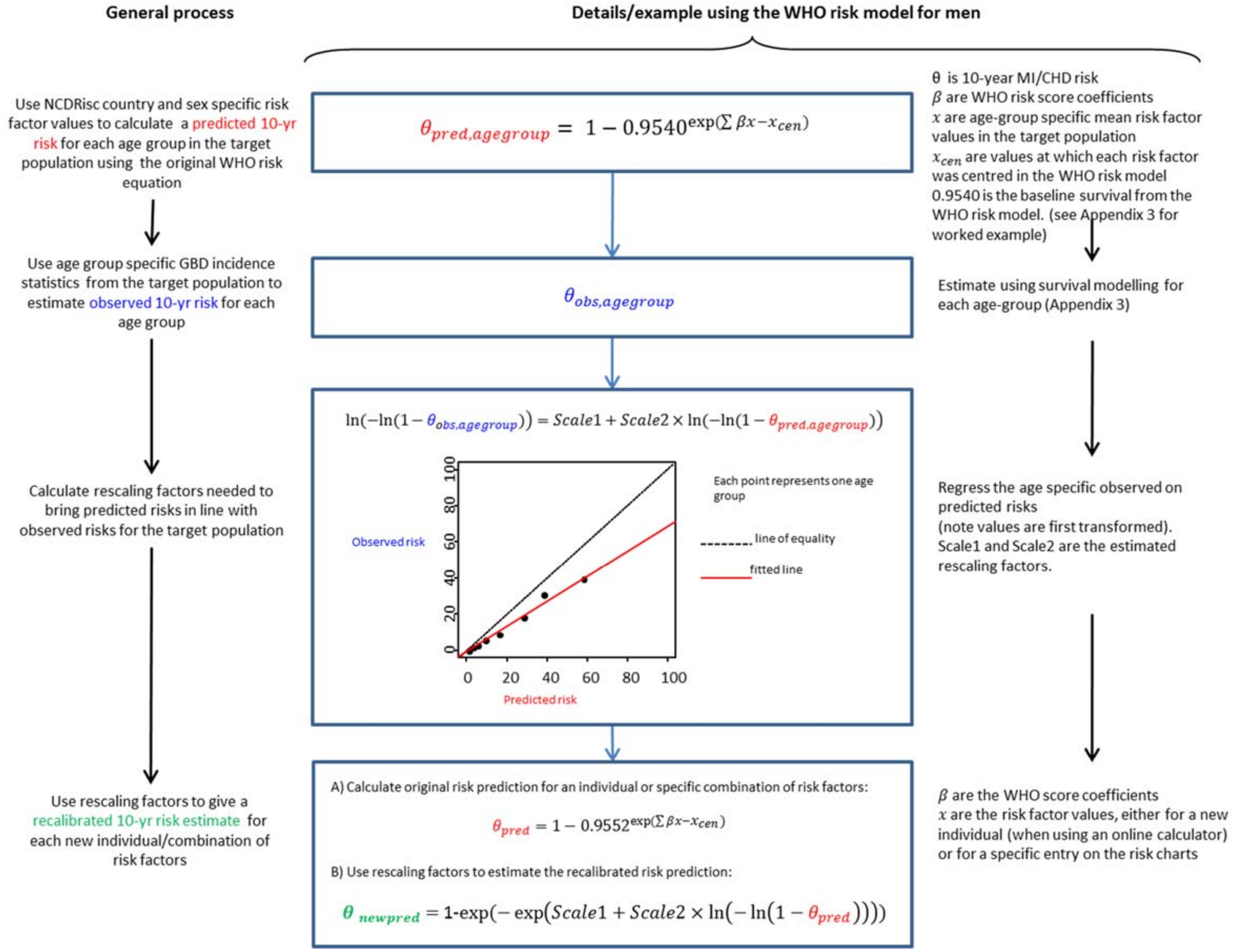

WHO: World Health Organisation. Appendix 3 gives further detail on methods for estimated predicted and observed risks

Figure 1.4: Changes in hazard ratios with age for the risk factors in the WHO risk models

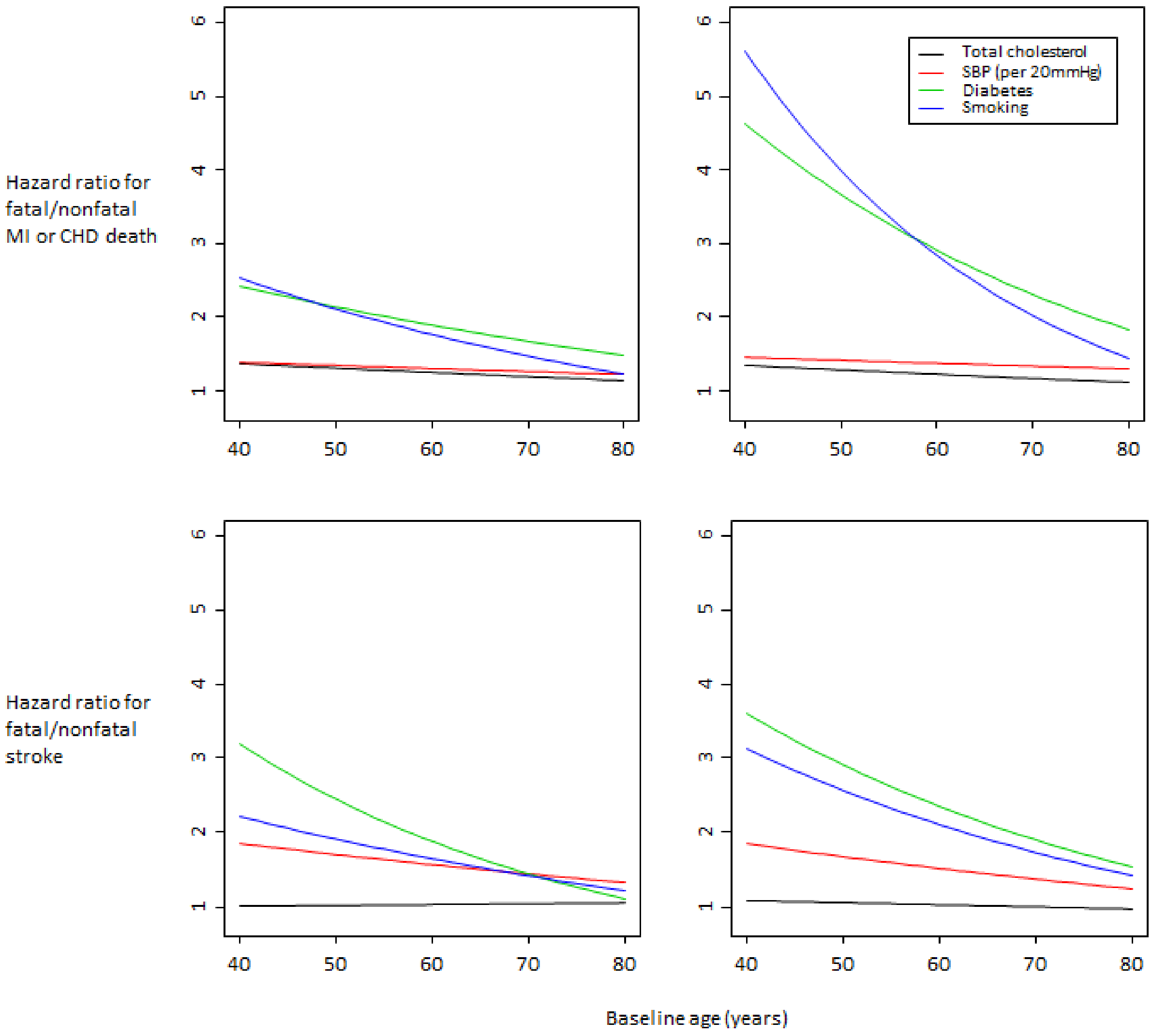

Figure 1.5: Calibration of the WHO risk models within the ERFC derivation dataset

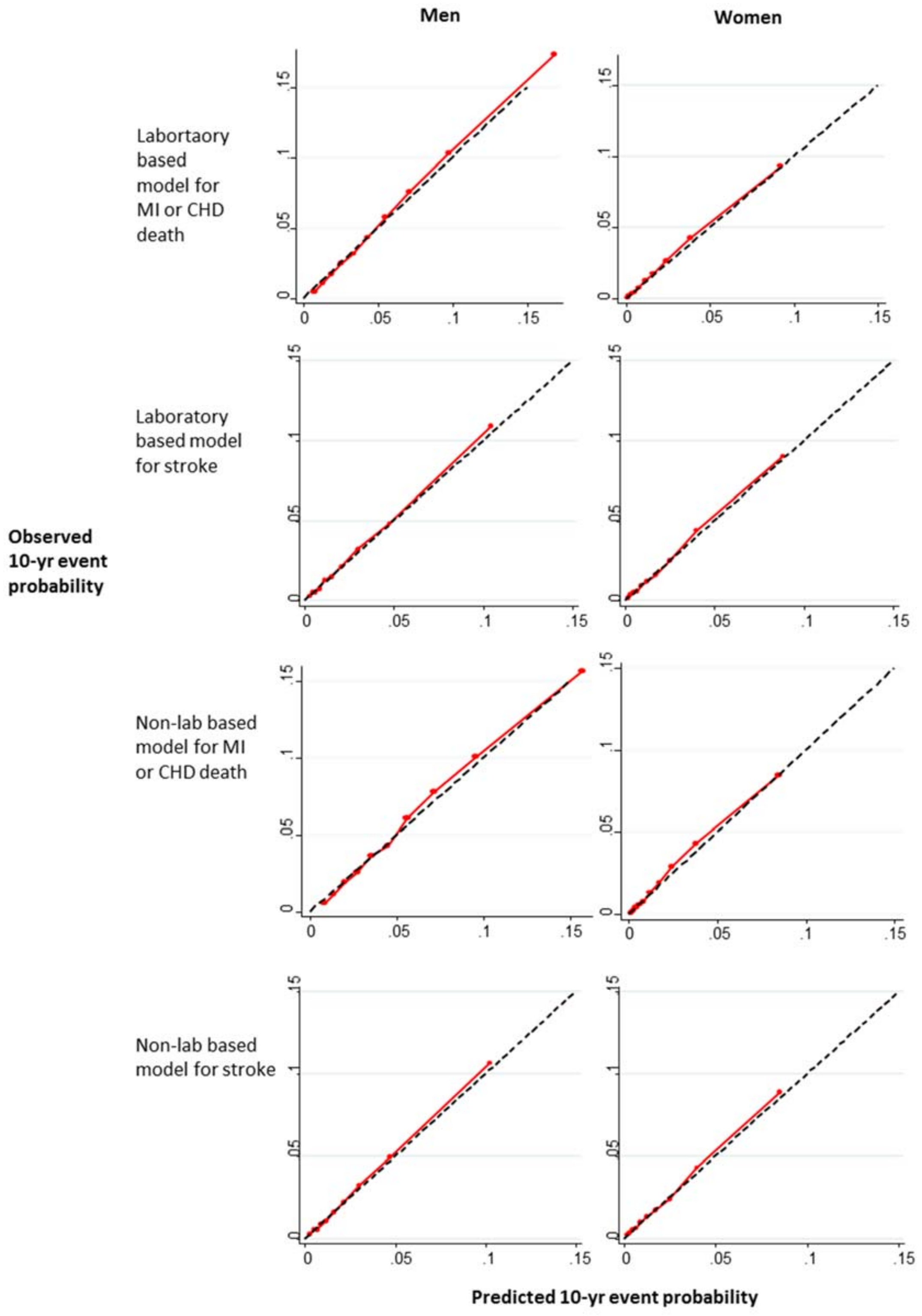

Figure 1.6: Calibration of the WHO risk models within subgroups of the ERFC derivation dataset defined by region and year of baseline survey

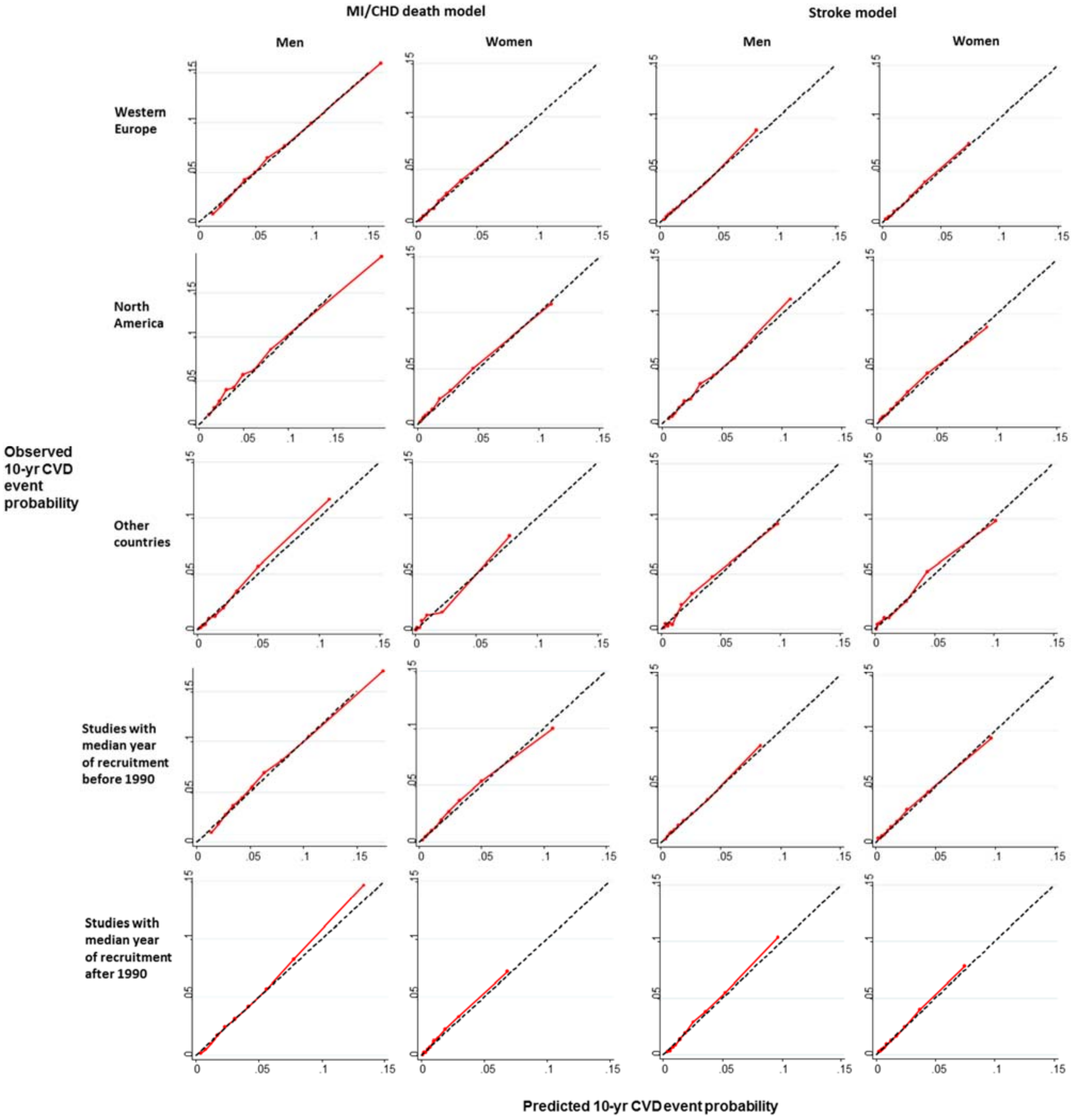

Figure 1.7: Annual MI incidence by region sex and age group used for recalibration of WHO models

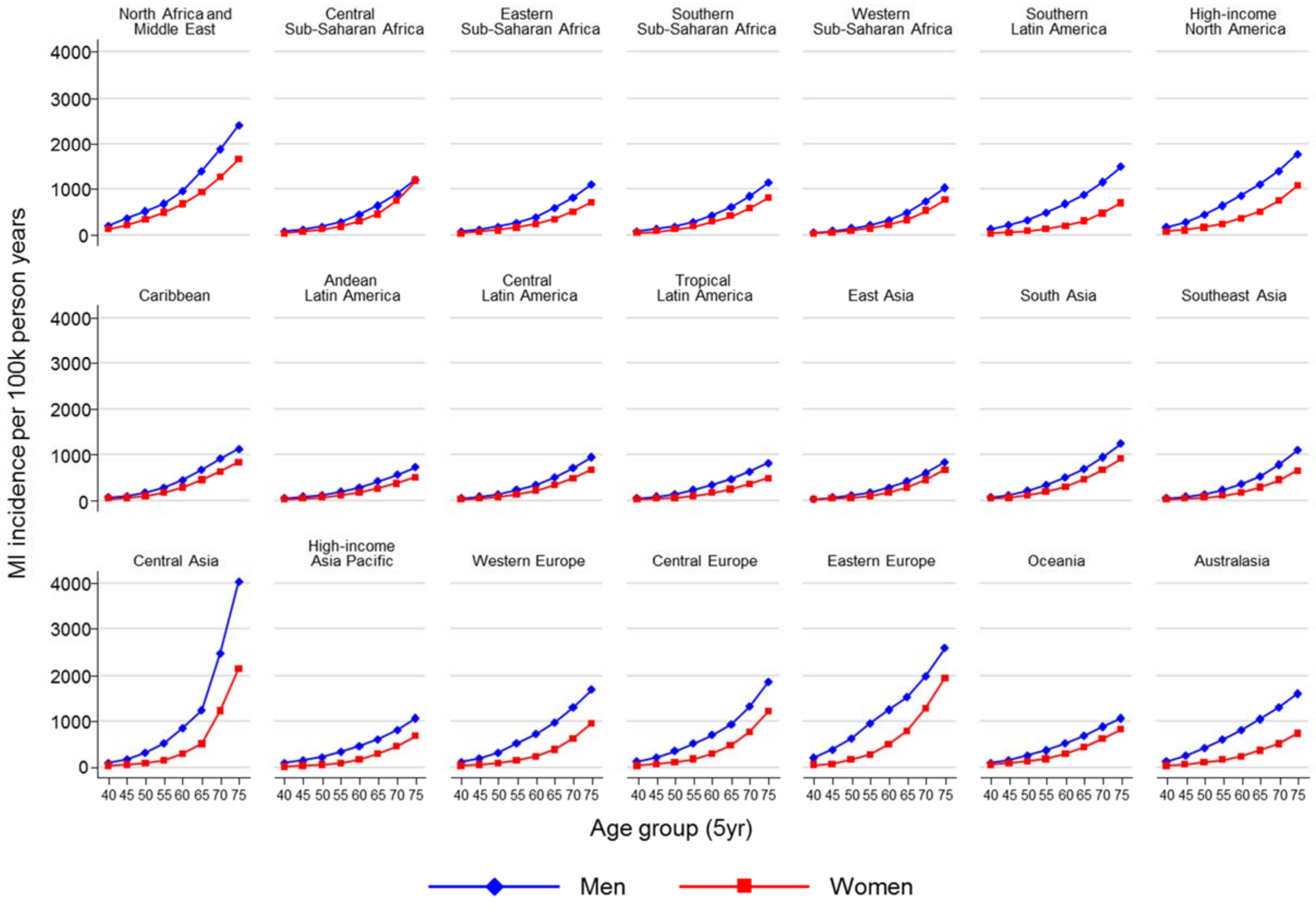

Figure 1.8: Annual stroke incidence by region sex and age group used for recalibration of WHO models

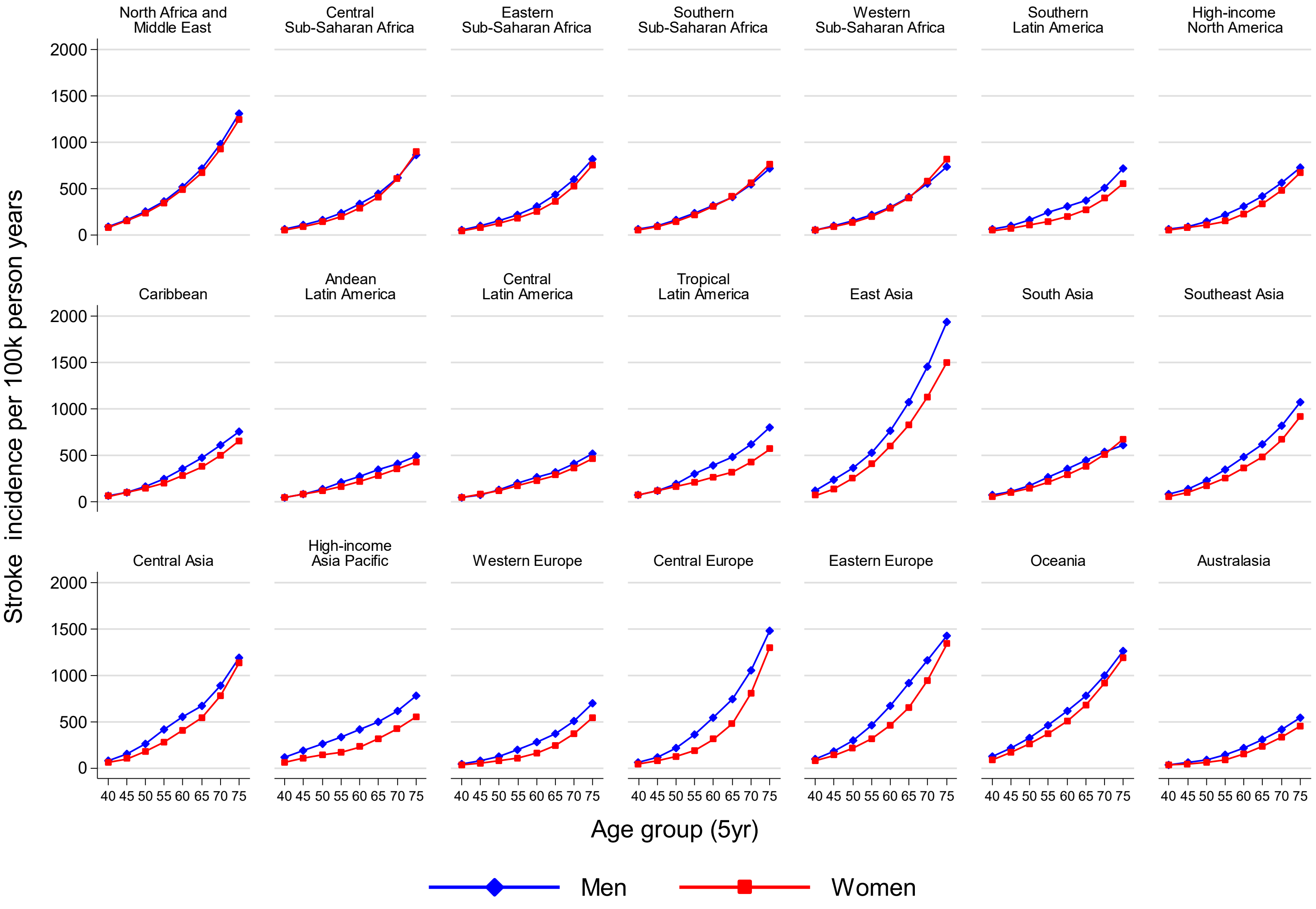

Figure 1.9: Standardised estimates of region-specific Stroke:MI incidence rate ratios using GBD data

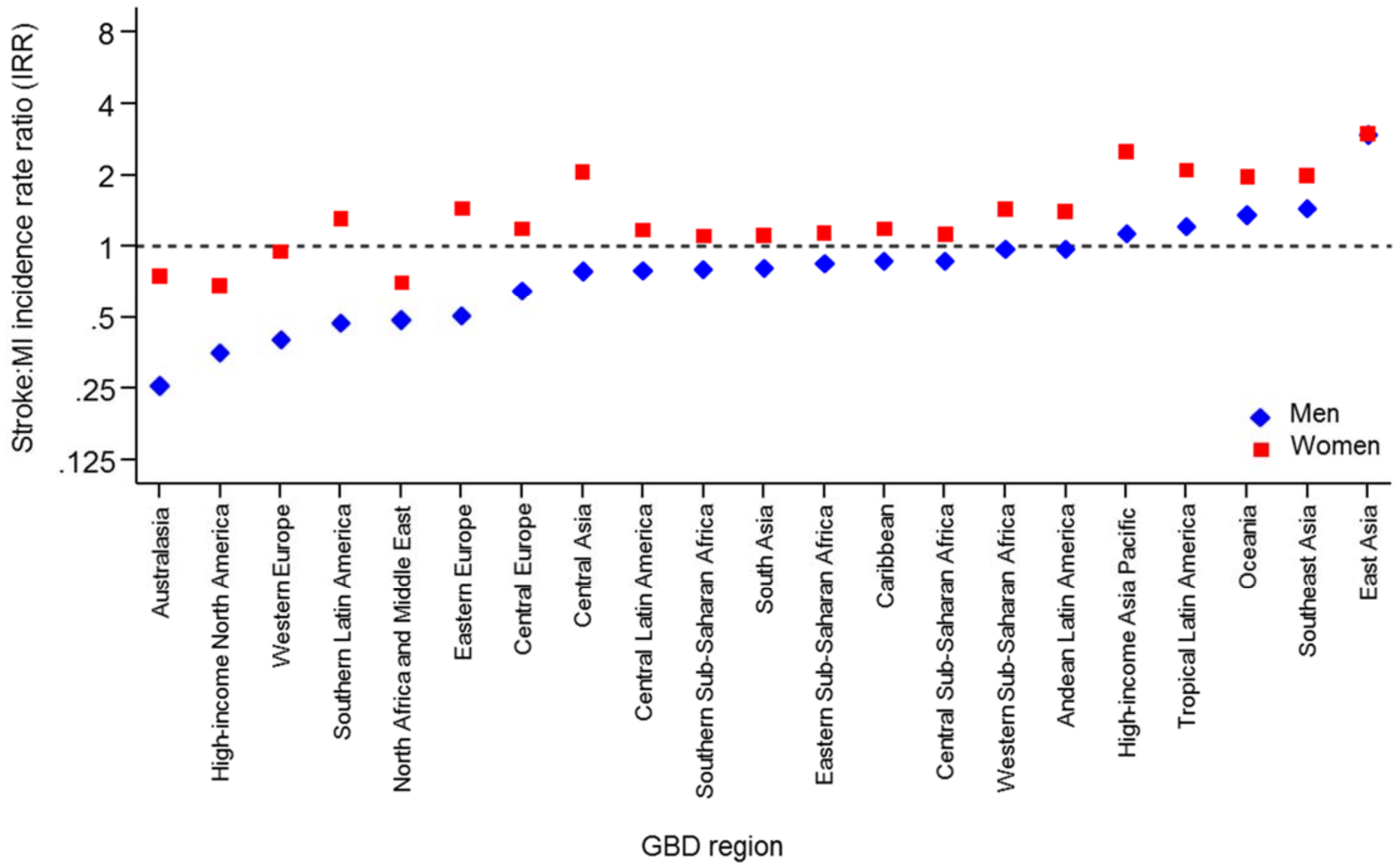

Standardised according to the world standard population (World Health Organisation 2000-2025)

Figure 1.10: Standardised estimates of region-specific Female:Male MI incidence rate ratios using GBD data

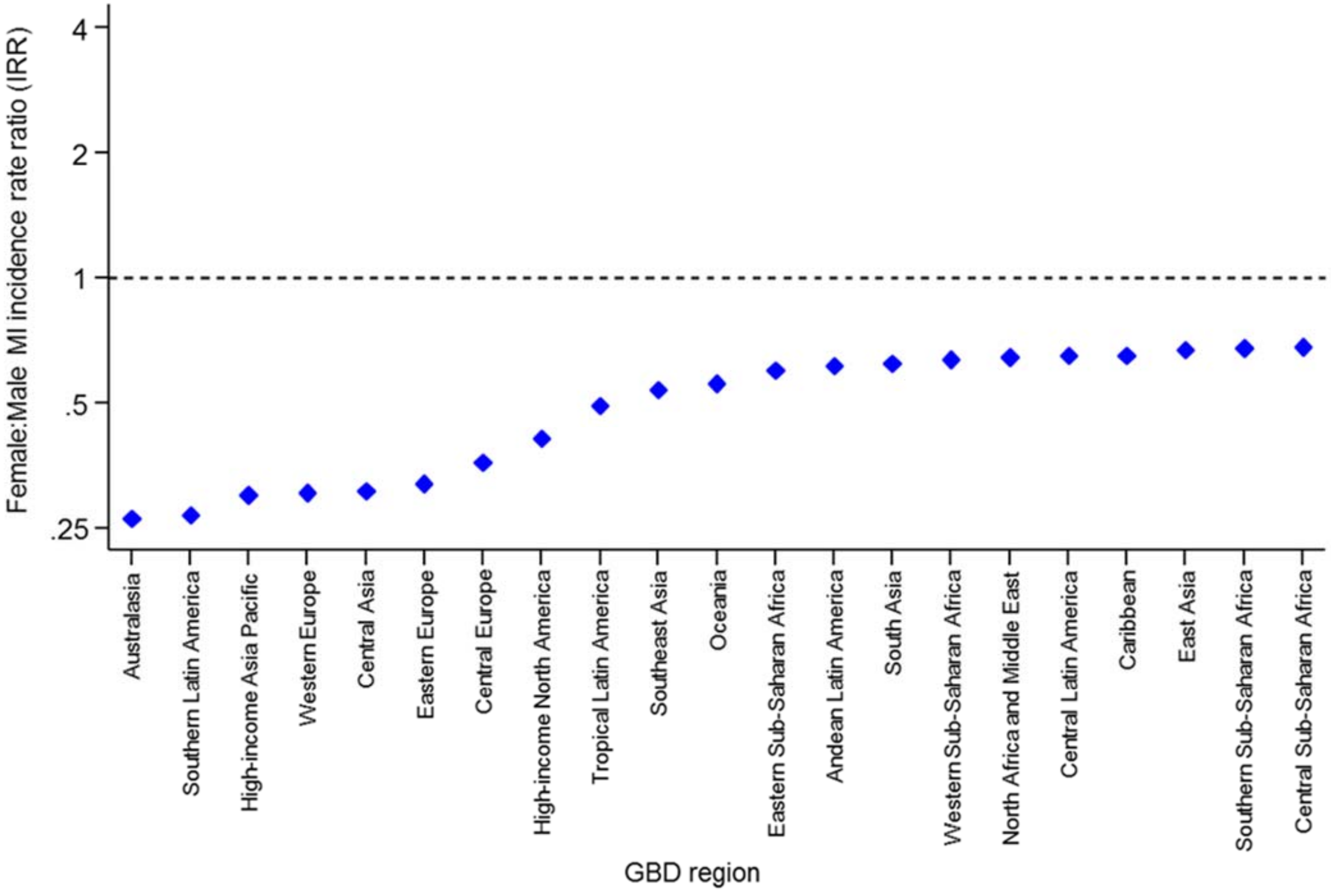

Standardised according to the world standard population (World Health Organisation 2000-2025)

Figure 1.11: Standardised estimates of region-specific Female:Male stroke incidence rate ratios using GBD data

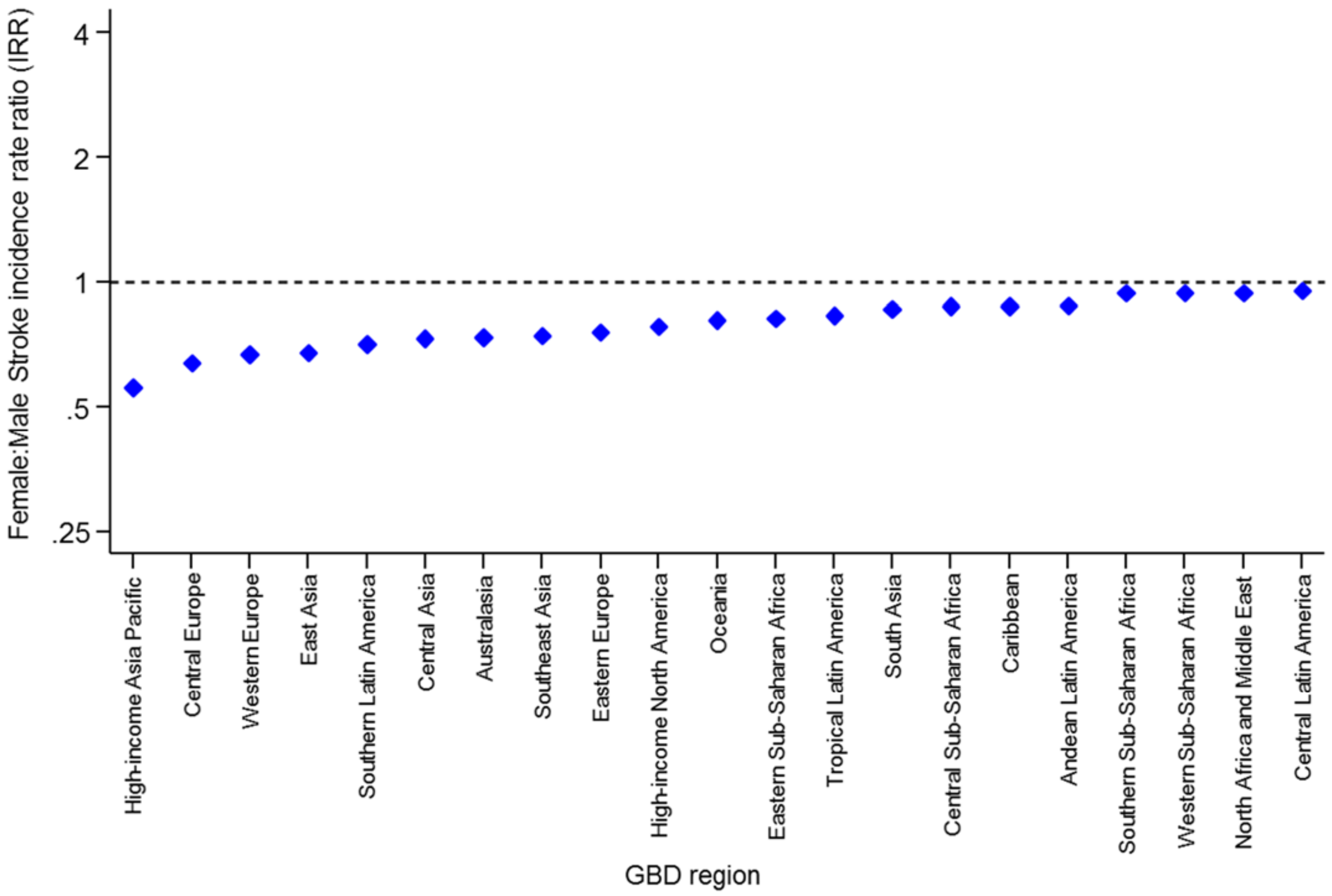

Standardised according to the world standard population (World Health Organisation 2000-2025)

Figure 1.12: Total cholesterol values by region, sex and age group used for recalibration of WHO models

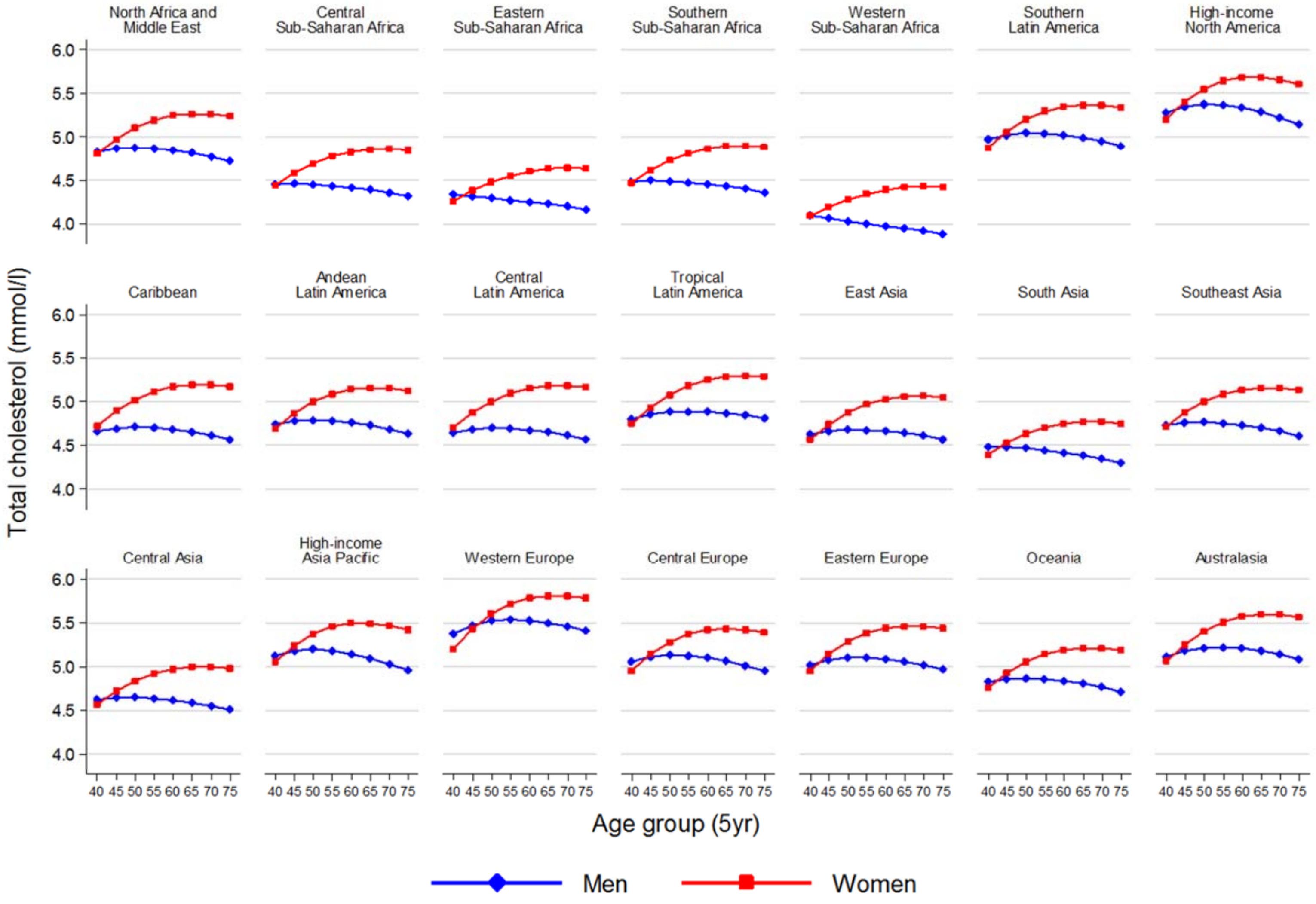

Figure 1.13: Systolic blood pressure values by region, sex and age group used for recalibration of WHO models

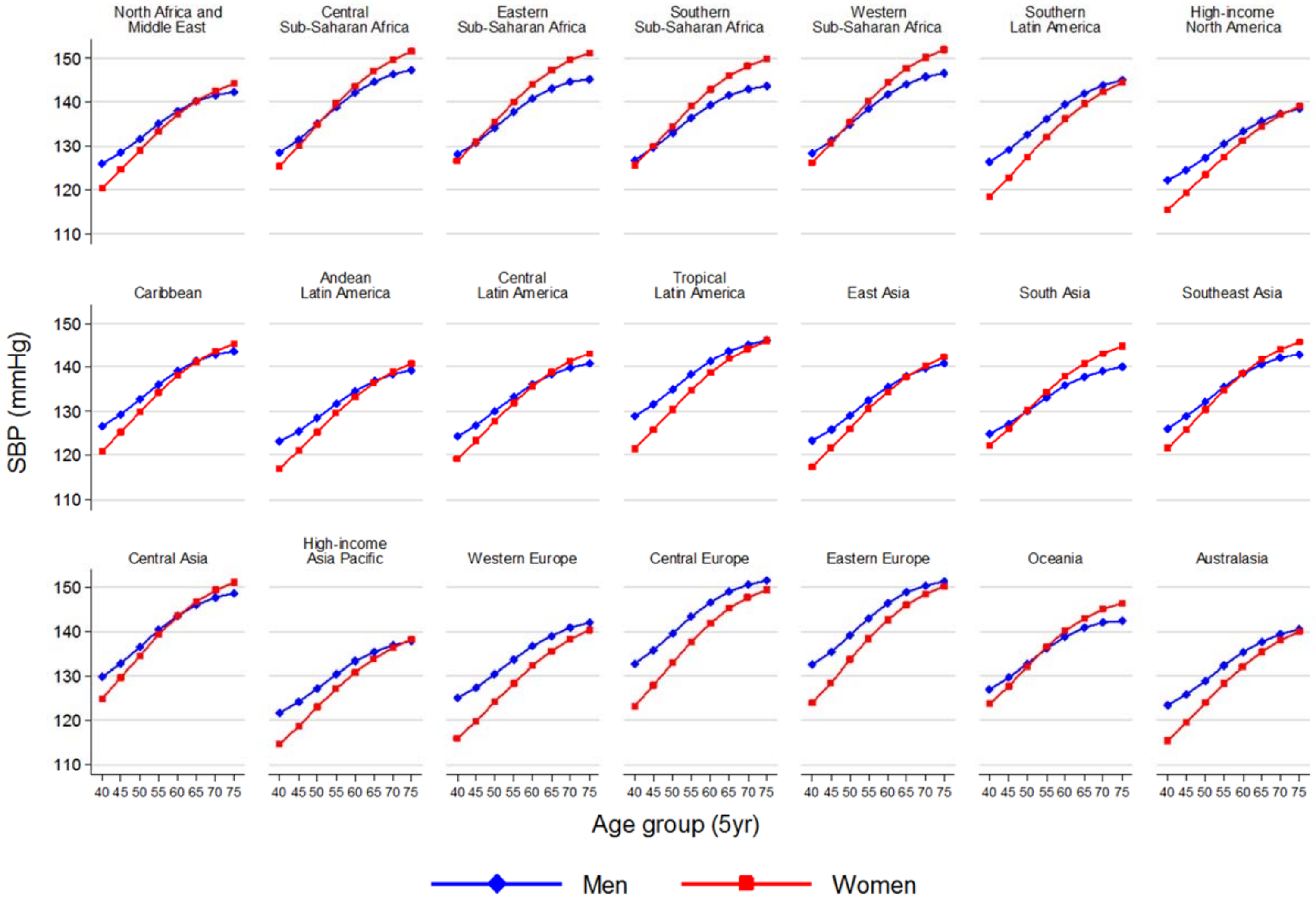

Figure 1.14: Diabetes prevalence by region, sex and age group used for recalibration of WHO models

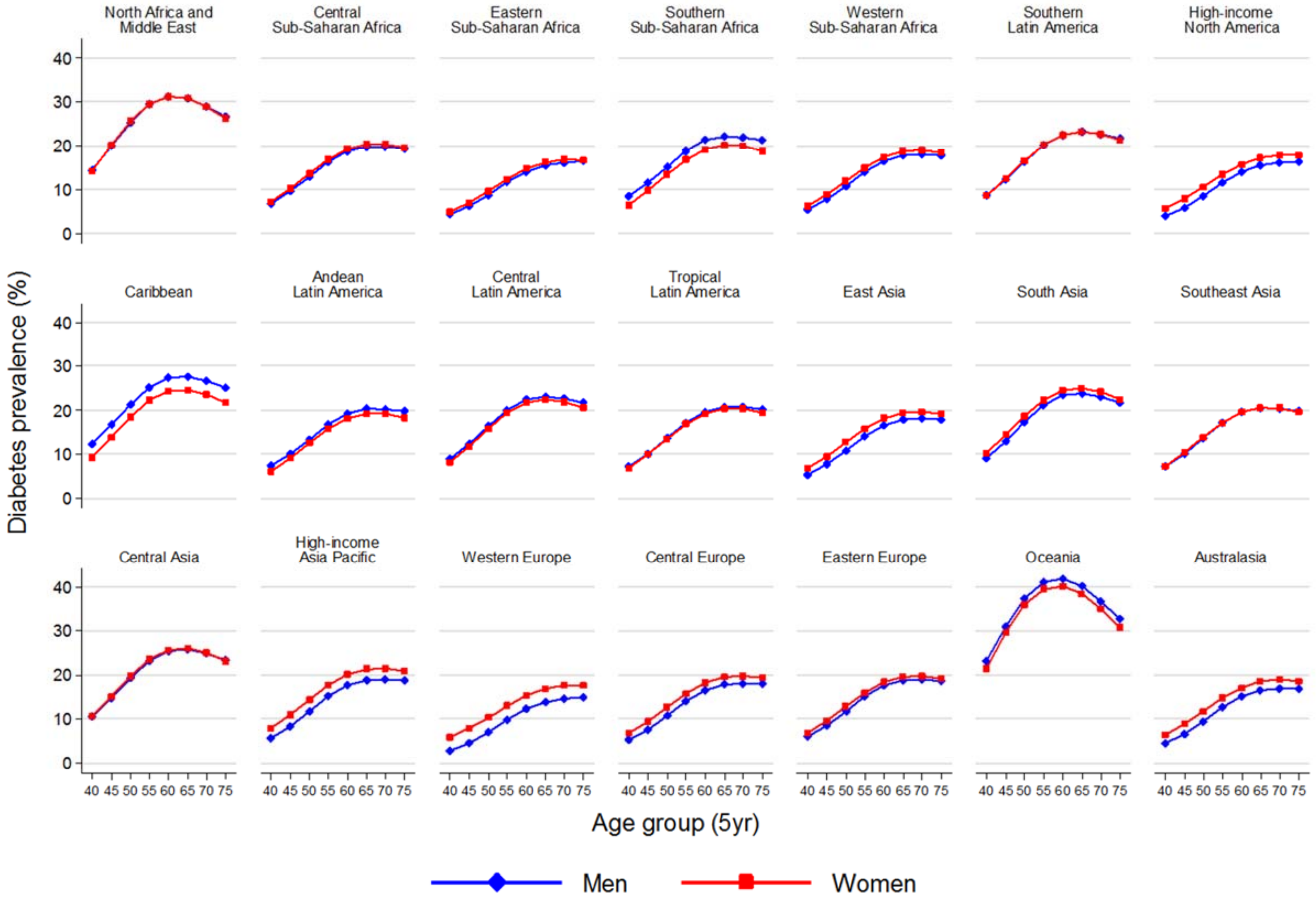

Figure 1.15: Smoking prevalence by region, sex and age group used for recalibration of WHO models

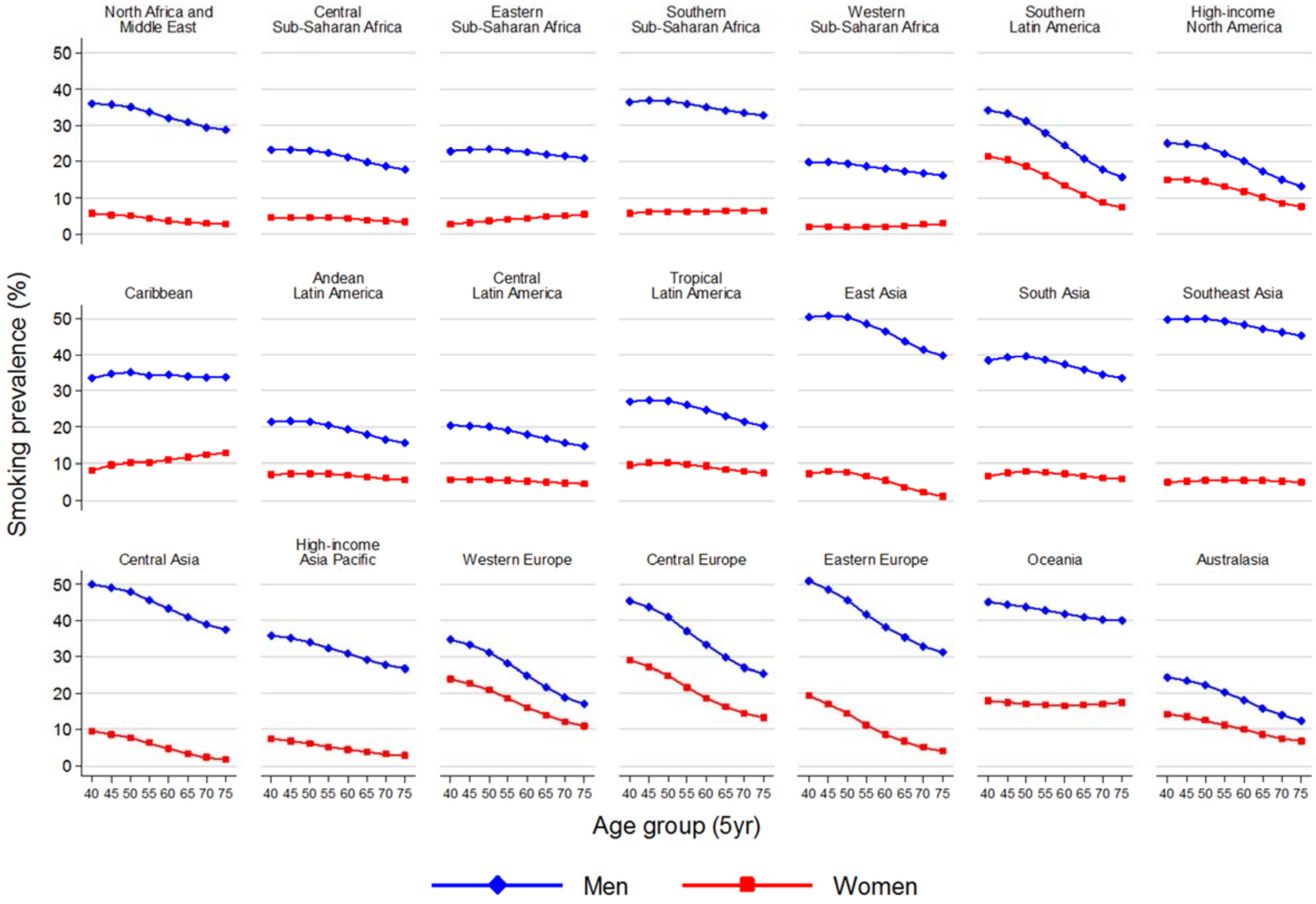

Figure 1.16: BMI levels by region, sex and age group used for recalibration of WHO models

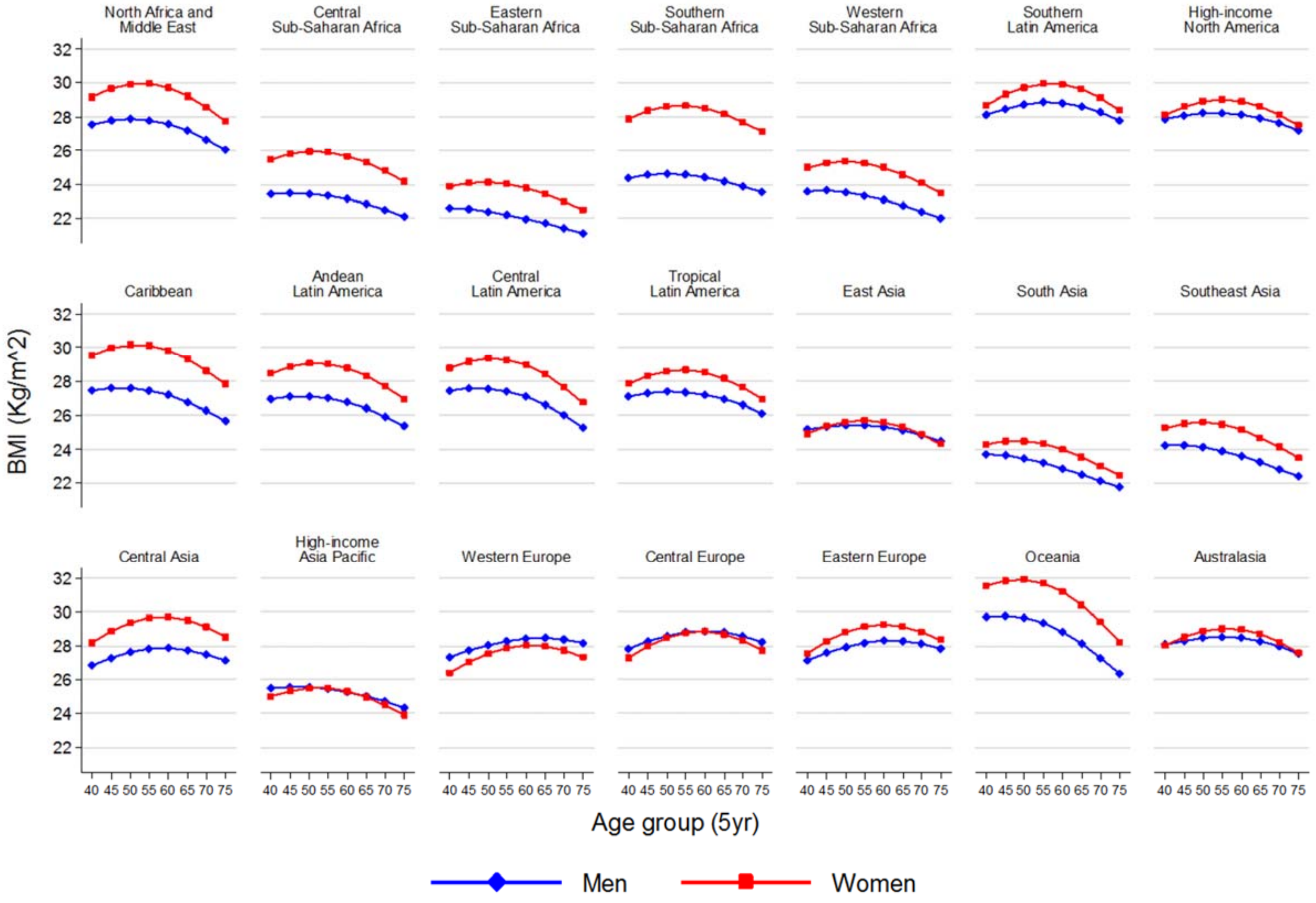

Figure 1.17: Comparison of expected 10-year CVD risks in men from 21 global regions vs risks estimated using un-calibrated and recalibrated WHO laboratory based model, including uncertainty in recalibrated risk estimates

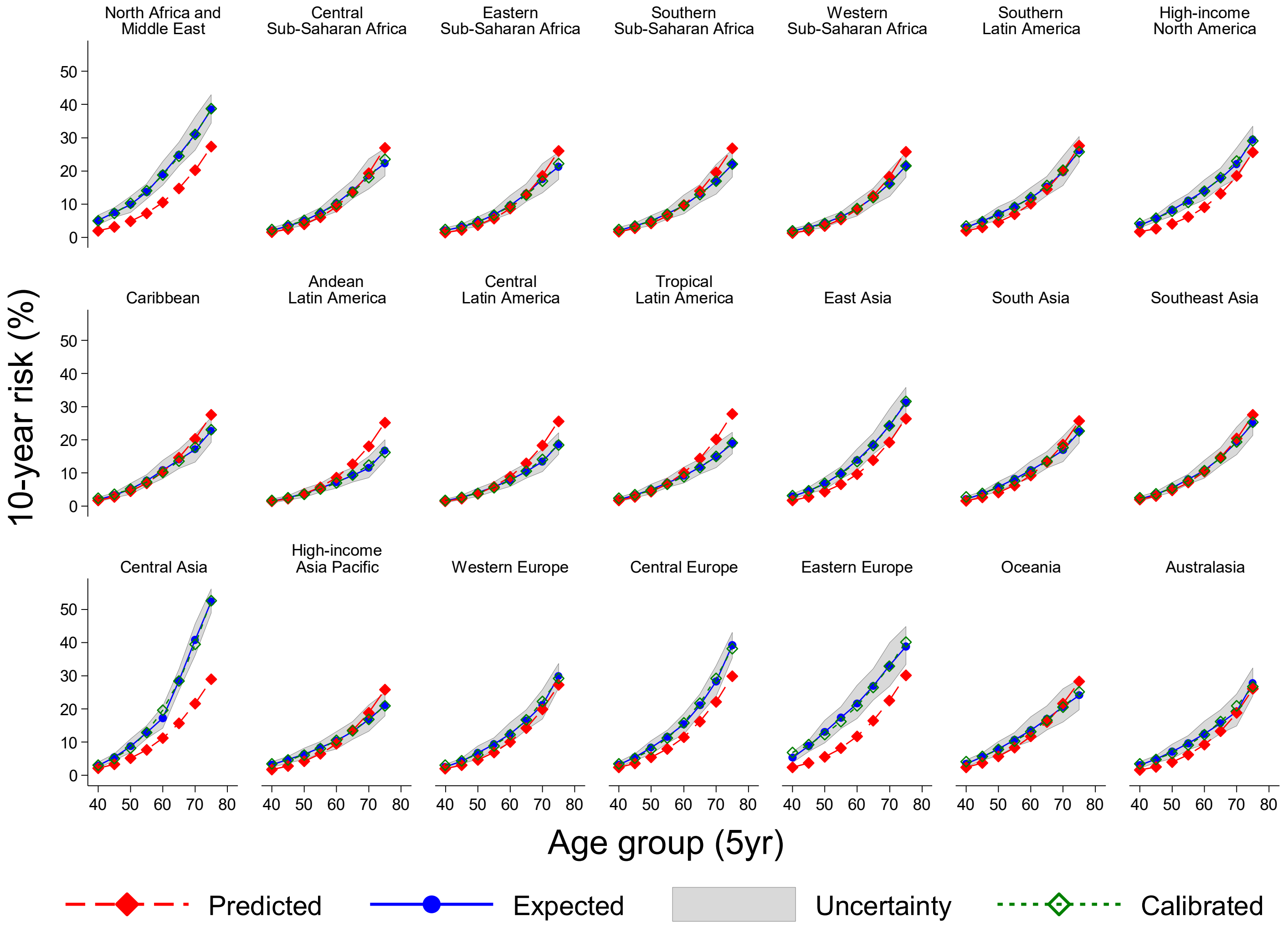

Uncertainty bands represent the possible range of recalibrated risk predictions using the upper and lower 95% confidence limits on the Global Burden of Disease incidence estimates

Figure 1.18: Comparison of expected 10-year CVD risks in women from 21 global regions vs risks estimated using un-calibrated and recalibrated WHO laboratory based model, including uncertainty in recalibrated risk estimates

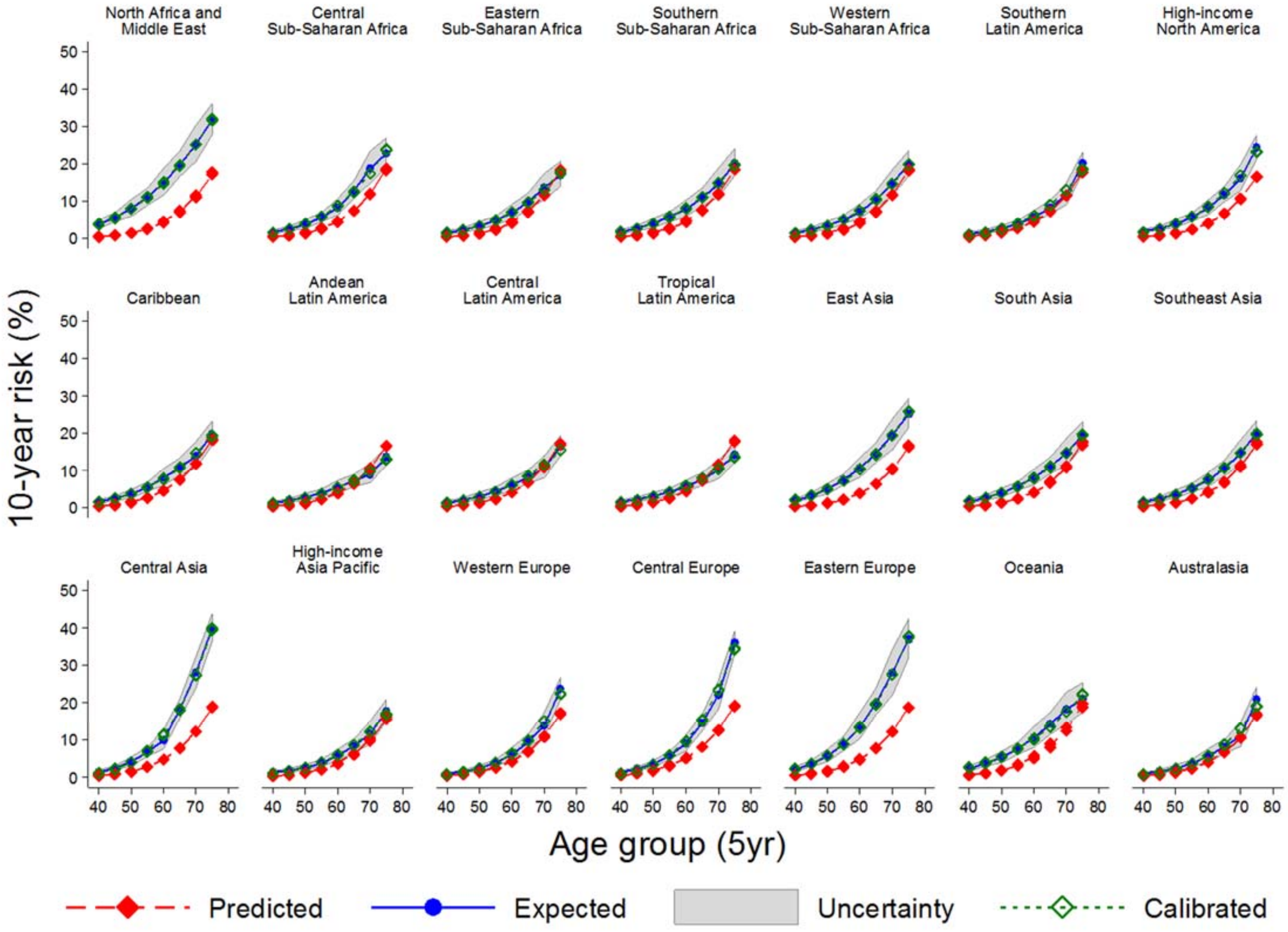

Uncertainty bands represent the possible range of recalibrated risk predictions using the upper and lower 95% confidence limits on the Global Burden of Disease incidence estimates

Figure 1.19: Calibration of 5-year WHO risk models in the PREDICT cohort

a) Laboratory-based model

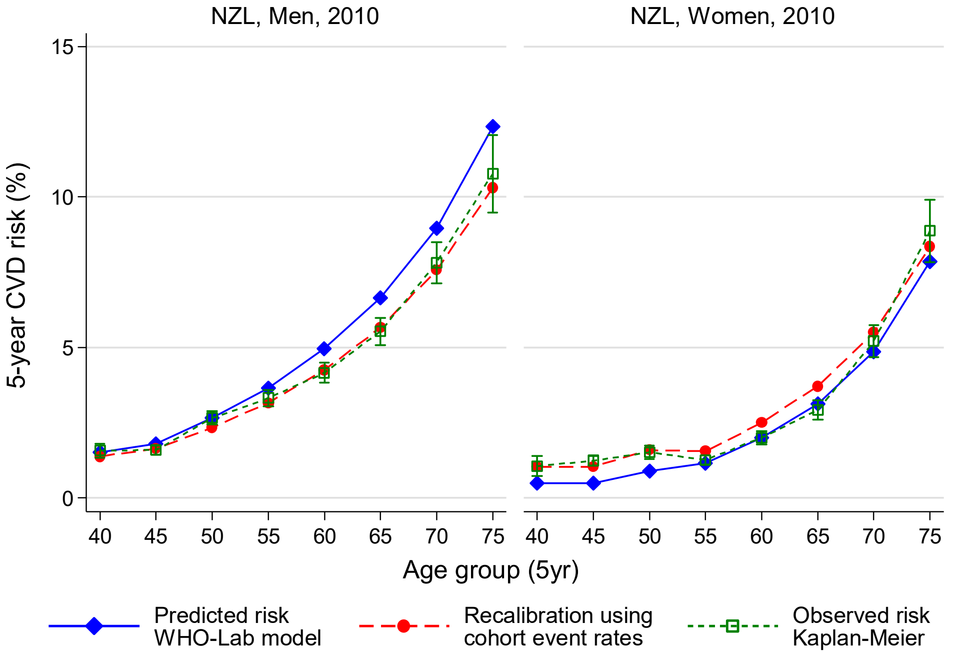

b) Non laboratory-based model

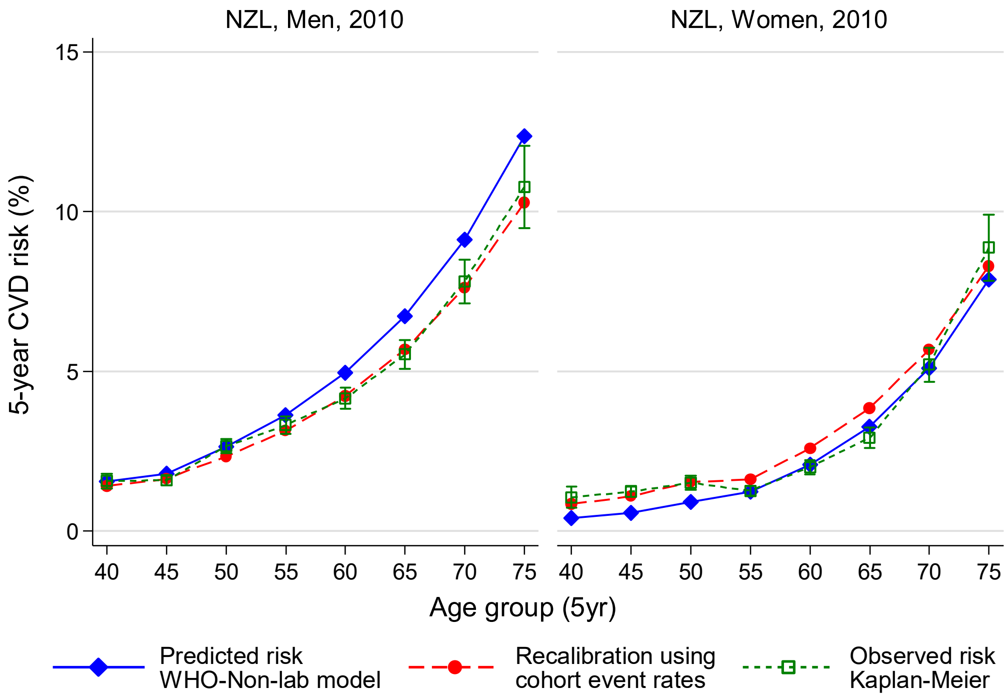

Figure 1.20: Ability of the non-laboratory based WHO model to discriminate, compared to the laboratory based model: C-index upon external validation

A) C-index for the non-laboratory-based model

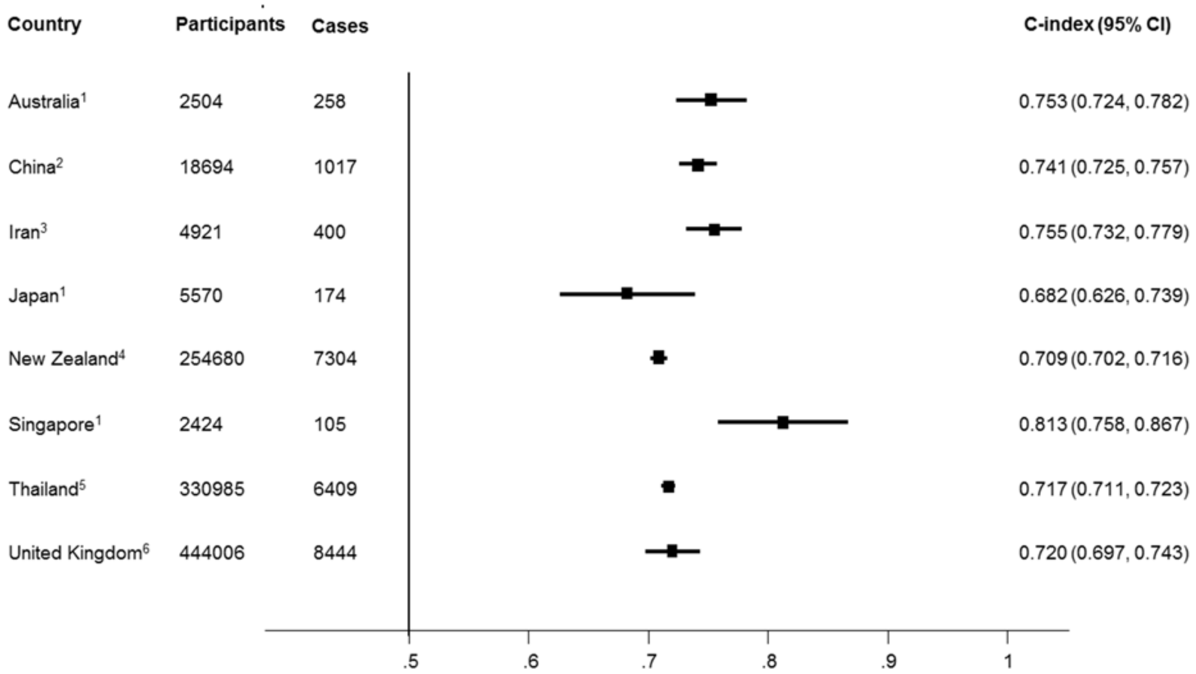

B) Difference between C-index: laboratory vs non-laboratory based models

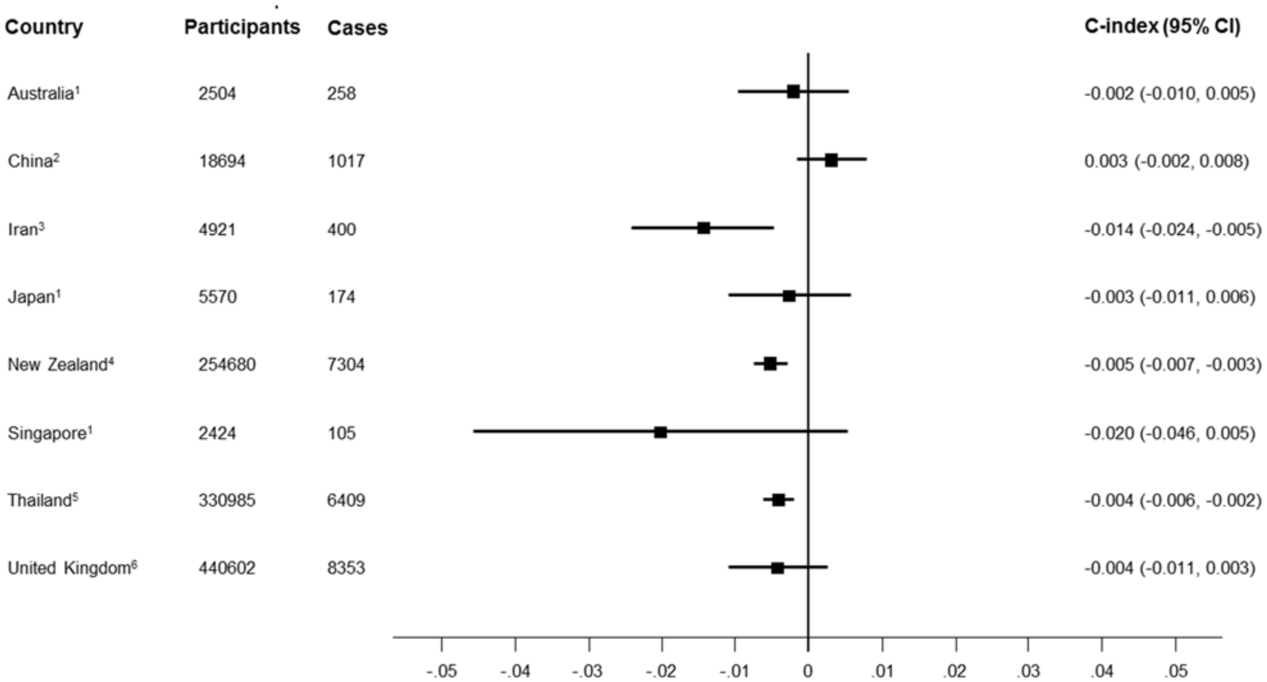

Country specific results are a result of pooling the within study C-index values weighting by the number of contributing events. Only studies recording both MI and Stroke were used. Study specific data is from the following sources:

[1] Calculated using relevant studies from the Asia Pacific Cohorts Studies Collaboration (APCSC)
[2] Calculated using relevant studies from the APCSC and the China Multi-Provincial Cohort Study (CMCS)
[3] Calculated using relevant studies from the APCSC and the PREDICT-CVD cohort
[4] Calculated using the Tehran Lipids and Glucose Study (TLGS)
[5] Calculated using the Health Checks Ubon Ratchathani Study (HCUR)
[6] Calculated using UK Biobank

Figure 1.21: Distribution of 10-year CVD risk according to recalibrated non-laboratory based WHO CVD risk model for individuals aged between 40 and 64 years from example countries.

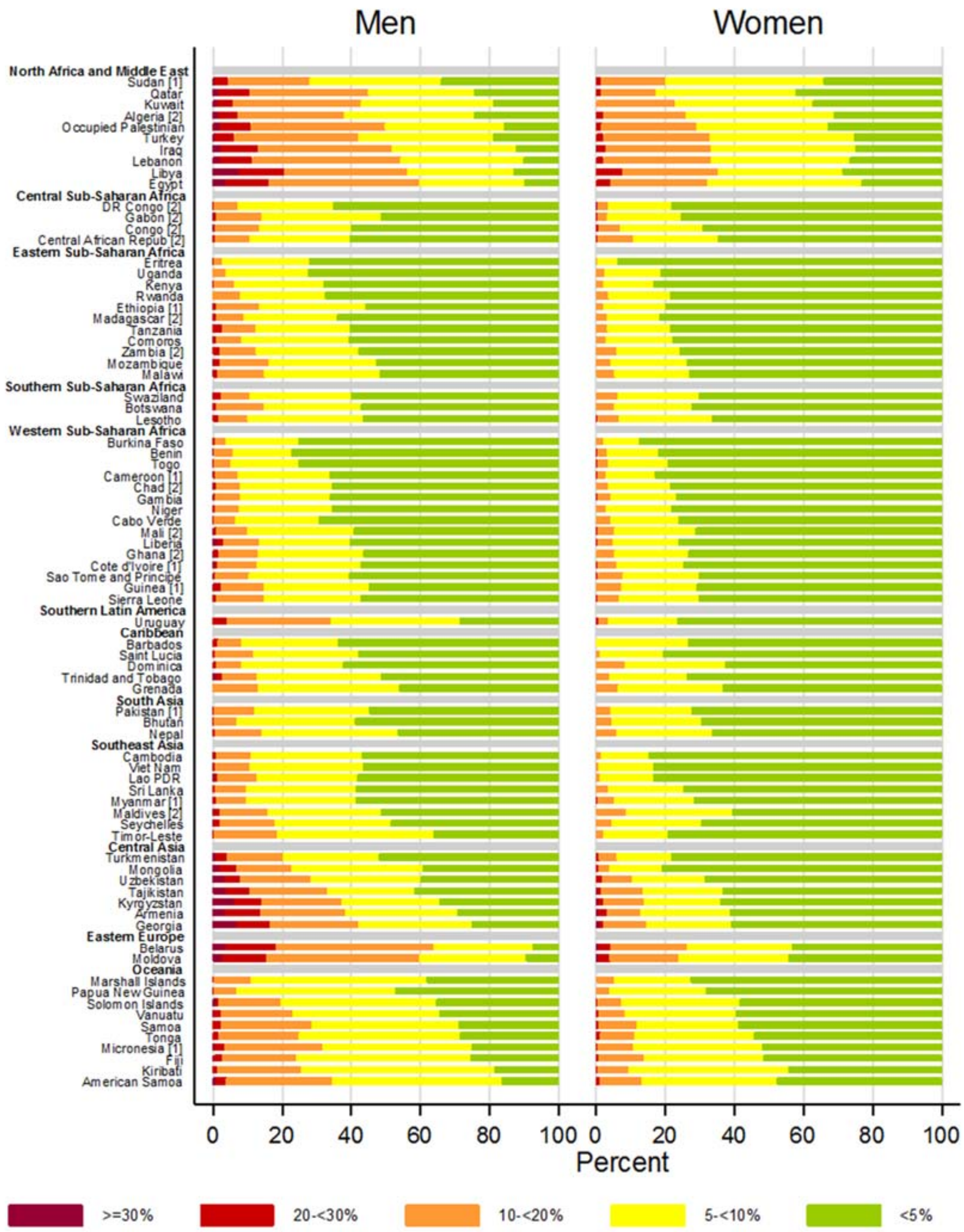

Data from all countries are from adults aged between 40 and 64 years and sampling representative of the national population unless otherwise indicated as 1) Subnational, or 2) Community based

Figure 1.22. Comparison of risk predictions estimated using recalibrated laboratory vs non-laboratory based WHO risk models in participants from WHO STEPS national surveys

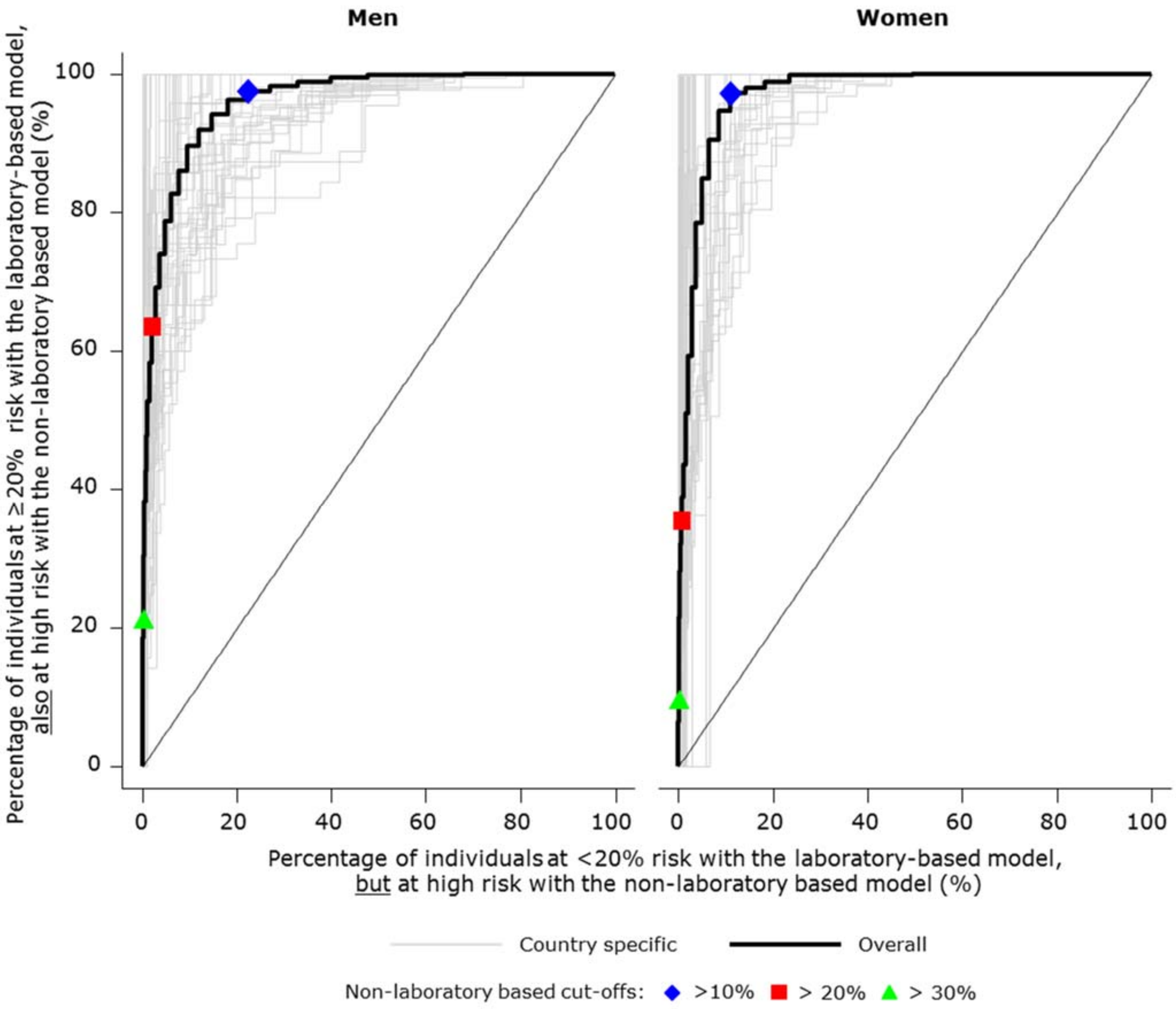

Figure 1.23: Comparison of 10-year CVD risk classification using recalibrated laboratory vs non-lab based WHO risk models in participants from WHO STEPS national surveys, by sex and diabetes status

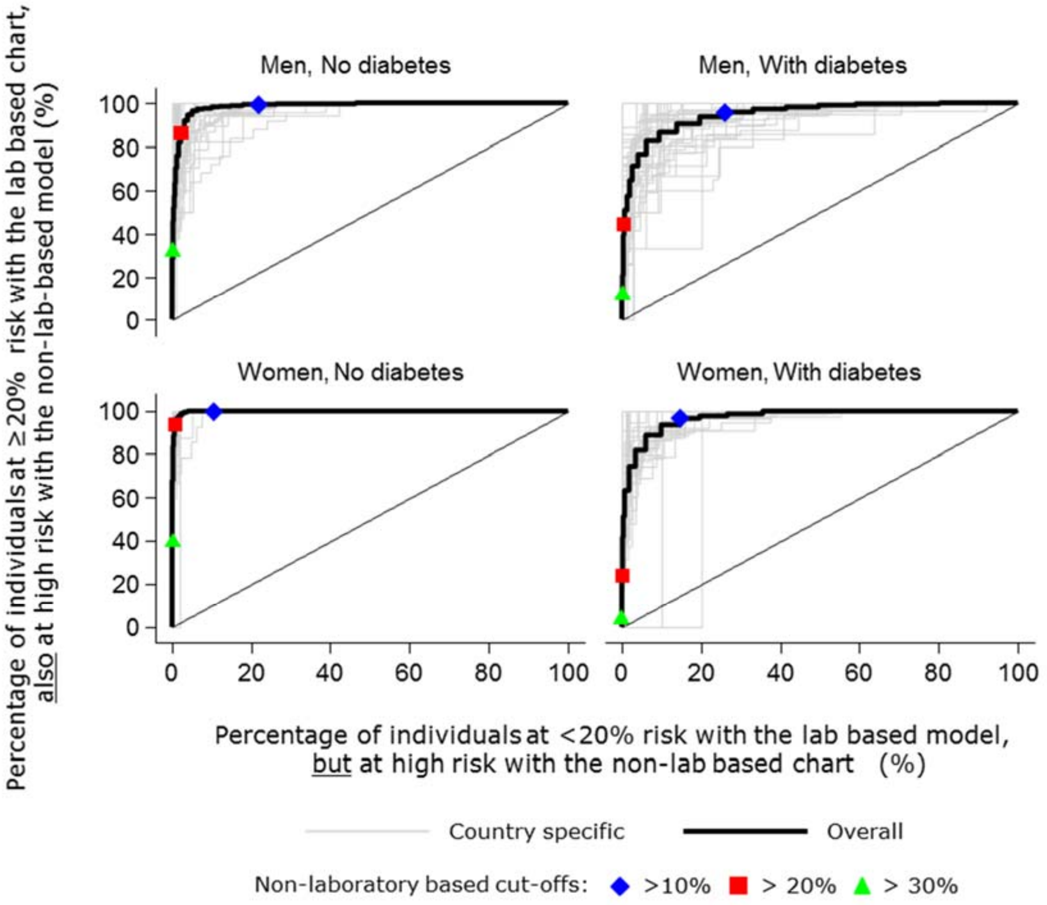

2) List of ERFC studies and abbreviations

ARIC, Atherosclerosis Risk in Communities Study
ATENA, cohort of Progetto CUORE
AUSDIAB, Australian Diabetes, Obesity and Lifestyle Study
BRHS, British Regional Heart Study
BRUN, Bruneck Study
BWHHS, British Women's Heart and Health Study
CAPS, Caerphilly Prospective Study
CASTEL, Cardiovascular Study in the Elderly
CHARL, Charleston Heart Study
CHS, Cardiovascular Health Study
COPEN, Copenhagen City Heart Study
DESIR, Data from an Epidemiological Study on the Insulin Resistance Syndrome
DUBBO, Dubbo Study of the Elderly
EAS, Edinburgh Artery Study
EMOFRI, part of CUORE
EPESEBOS, The Established Populations for the Epidemiologic Study of the Elderly Studies, Boston
EPESEIOW, The Established Populations for the Epidemiologic Study of the Elderly Studies, Iowa
EPESENCA, The Established Populations for the Epidemiologic Study of the Elderly Studies, North Carolina
EPESENHA, The Established Populations for the Epidemiologic Study of the Elderly Studies, New Haven
EPICNOR, European Prospective Investigation of Cancer Norfolk Study
ESTHER, Epidemiologische Studie zu Chancen der Verhütung und optimierten Therapie chronischer Erkrankungen in der älteren Bevölkerung
FINE_FIN, Finland, Italy and Netherlands Elderly Study – Finland cohort
FINE_IT, Finland, Italy and Netherlands Elderly Study – Italian cohort
FINRISK-92, Finrisk Cohort 1992
FINRISK-97, Finrisk Cohort 1997
FRAMOFF, Framingham Offspring Cohort
FUNAGATA, Funagata Study
GOTO, Göteborg Study
GOTOW, Population Study of Women in Göteborg, Sweden
GRIPS, Göttingen Risk Incidence and Prevalence Study
HISAYAMA, Hisayama Study
HONOL, Honolulu Heart Program
HOORN, Hoorn Study
IKNS, Ikawa, Kyowa, and Noichi Study
KIHD, Kuopio Ischaemic Heart Disease Study
LEADER, Lower Extremity Arterial Disease Event Reduction Trial
MATISS-83, 87, 93, cohort of Progetto CUORE
MESA, Multi-Ethnic Study of Atherosclerosis
MIDCOLL, MIDSPAN Collaborative study
MIDFAM, MIDSPAN Family Study
MIDRP, MIDSPAN Renfrew/Paisley Study
MONFRI86, cohort of Progetto CUORE
MONFRI89, cohort of Progetto CUORE
MONFRI94, cohort of Progetto CUORE
MONICA_KORA2, MONICA/KORA Augsburg Surveys S1
MONICA_KORA2, MONICA/KORA Augsburg Surveys S2
MONICA_KORA3, MONICA/KORA Augsburg Surveys S3
MOSWEGOT, MONICA Göteborg Study
MPP, Malmö Preventive Project
MRFIT, Multiple Risk Factor Intervention Trial 1
NHANES I, National Health and Nutrition Examination Survey
NPHSII, Northwick Park Heart Study II
NSHS, Nova Scotia Health Survey
OSAKA, Osaka Study
PREVEND, Prevention of Renal and Vascular End Stage Disease Study
PRHHP, Puerto Rico Heart Health Program
PRIME, Prospective Epidemiological Study of Myocardial Infarction
PROCAM, Prospective Cardiovascular Münster Study
ProspectEPIC, Prospect-EPIC Utrecht
PROSPER, Prospective Study of Pravastatin in the Elderly at Risk
QUEBEC, Quebec Cardiovascular Study

RANCHO, Rancho Bernardo Study
REYK, Reykjavik Study
RS_I, The Rotterdam Study I
RS_II, The Rotterdam Study II
SHHEC, Scottish Heart Health Extended Cohort
SHIP, Study of Health in Pomerania
SHS, Strong Heart Study
SPEED, Speedwell Study
TARFS, Turkish Adult Risk Factor Study
TOYAMA, Toyama Study
TROMSØ, Tromsø Study
ULSAM, Uppsala Longitudinal Study of Adult Men
USPHS2, U.S. Physicians Health Study 2
WCWC, Württemberg Construction Worker Cohort
WHITE II, Whitehall II Study
WHS, Women's Health Study
WOSCOPS, West of Scotland Coronary Prevention Study
ZARAGOZA, Zaragoza Study
ZUTE, Zutphen Elderly Study

3) Countries included in the 21 regions defined by the Global Burden of Disease Study

Region	Country	GBD Region
Africa	Angola	Central Sub-Saharan Africa
Africa	Central African Republic	Central Sub-Saharan Africa
Africa	DR Congo	Central Sub-Saharan Africa
Africa	Congo	Central Sub-Saharan Africa
Africa	Gabon	Central Sub-Saharan Africa
Africa	Equatorial Guinea	Central Sub-Saharan Africa
Africa	Burundi	Eastern Sub-Saharan Africa
Africa	Comoros	Eastern Sub-Saharan Africa
Africa	Djibouti	Eastern Sub-Saharan Africa
Africa	Eritrea	Eastern Sub-Saharan Africa
Africa	Ethiopia	Eastern Sub-Saharan Africa
Africa	Kenya	Eastern Sub-Saharan Africa
Africa	Madagascar	Eastern Sub-Saharan Africa
Africa	Mozambique	Eastern Sub-Saharan Africa
Africa	Malawi	Eastern Sub-Saharan Africa
Africa	Rwanda	Eastern Sub-Saharan Africa
Africa	Somalia	Eastern Sub-Saharan Africa
Africa	Tanzania	Eastern Sub-Saharan Africa
Africa	Uganda	Eastern Sub-Saharan Africa
Africa	Zambia	Eastern Sub-Saharan Africa
Africa	Algeria	North Africa and Middle East
Africa	Egypt	North Africa and Middle East
Africa	Libya	North Africa and Middle East
Africa	Morocco	North Africa and Middle East
Africa	South Sudan	North Africa and Middle East
Africa	Sudan	North Africa and Middle East
Africa	Tunisia	North Africa and Middle East
Africa	Mauritius	Southeast Asia
Africa	Seychelles	Southeast Asia
Africa	Botswana	Southern Sub-Saharan Africa
Africa	Lesotho	Southern Sub-Saharan Africa
Africa	Namibia	Southern Sub-Saharan Africa
Africa	Swaziland	Southern Sub-Saharan Africa
Africa	South Africa	Southern Sub-Saharan Africa
Africa	Zimbabwe	Southern Sub-Saharan Africa
Africa	Benin	Western Sub-Saharan Africa
Africa	Burkina Faso	Western Sub-Saharan Africa
Africa	Cote d'Ivoire	Western Sub-Saharan Africa
Africa	Cameroon	Western Sub-Saharan Africa
Africa	Cabo Verde	Western Sub-Saharan Africa
Africa	Ghana	Western Sub-Saharan Africa
Africa	Guinea	Western Sub-Saharan Africa
Africa	Gambia	Western Sub-Saharan Africa
Africa	Guinea Bissau	Western Sub-Saharan Africa
Africa	Liberia	Western Sub-Saharan Africa
Africa	Mali	Western Sub-Saharan Africa
Africa	Mauritania	Western Sub-Saharan Africa
Africa	Niger	Western Sub-Saharan Africa
Africa	Nigeria	Western Sub-Saharan Africa
Africa	Senegal	Western Sub-Saharan Africa
Africa	Sierra Leone	Western Sub-Saharan Africa
Africa	Sao Tome and Principe	Western Sub-Saharan Africa
Africa	Chad	Western Sub-Saharan Africa
Africa	Togo	Western Sub-Saharan Africa
Americas	Bolivia	Andean Latin America
Americas	Ecuador	Andean Latin America
Americas	Peru	Andean Latin America
Americas	Antigua and Barbuda	Caribbean
Americas	Bahamas	Caribbean
Americas	Belize	Caribbean
Americas	Bermuda	Caribbean
Americas	Barbados	Caribbean
Americas	Cuba	Caribbean
Americas	Dominica	Caribbean
Americas	Dominican Republic	Caribbean
Americas	Grenada	Caribbean
Americas	Guyana	Caribbean
Americas	Haiti	Caribbean
Americas	Jamaica	Caribbean
Americas	Saint Lucia	Caribbean
Americas	Puerto Rico	Caribbean
Americas	Suriname	Caribbean
Americas	Trinidad and Tobago	Caribbean
Americas	Saint Vincent and the Grenadines	Caribbean
Americas	Virgin Islands, U.S.	Caribbean
Americas	Colombia	Central Latin America
Americas	Costa Rica	Central Latin America
Americas	Guatemala	Central Latin America
Americas	Honduras	Central Latin America
Americas	Mexico	Central Latin America
Americas	Nicaragua	Central Latin America
Americas	Panama	Central Latin America
Americas	El Salvador	Central Latin America
Americas	Venezuela	Central Latin America
Americas	Canada	High-income North America
Americas	Greenland	High-income North America
Americas	United States of America	High-income North America
Americas	Argentina	Southern Latin America
Americas	Chile	Southern Latin America
Americas	Uruguay	Southern Latin America
Americas	Brazil	Tropical Latin America
Americas	Paraguay	Tropical Latin America
Asia	Armenia	Central Asia
Asia	Azerbaijan	Central Asia
Asia	Georgia	Central Asia
Asia	Kazakhstan	Central Asia
Asia	Kyrgyzstan	Central Asia
Asia	Mongolia	Central Asia

Region	Country	GBD Region
Asia	Tajikistan	Central Asia
Asia	Turkmenistan	Central Asia
Asia	Uzbekistan	Central Asia
Asia	China	East Asia
Asia	North Korea	East Asia
Asia	Taiwan	East Asia
Asia	Brunei Darussalam	High-income Asia Pacific
Asia	Japan	High-income Asia Pacific
Asia	South Korea	High-income Asia Pacific
Asia	Singapore	High-income Asia Pacific
Asia	Afghanistan	North Africa and Middle East
Asia	United Arab Emirates	North Africa and Middle East
Asia	Bahrain	North Africa and Middle East
Asia	Iran	North Africa and Middle East
Asia	Iraq	North Africa and Middle East
Asia	Jordan	North Africa and Middle East
Asia	Kuwait	North Africa and Middle East
Asia	Lebanon	North Africa and Middle East
Asia	Oman	North Africa and Middle East
Asia	Occupied Palestinian Territory	North Africa and Middle East
Asia	Qatar	North Africa and Middle East
Asia	Saudi Arabia	North Africa and Middle East
Asia	Syrian Arab Republic	North Africa and Middle East
Asia	Turkey	North Africa and Middle East
Asia	Yemen	North Africa and Middle East
Asia	Bangladesh	South Asia
Asia	Bhutan	South Asia
Asia	India	South Asia
Asia	Nepal	South Asia
Asia	Pakistan	South Asia
Asia	Indonesia	Southeast Asia
Asia	Cambodia	Southeast Asia
Asia	Lao PDR	Southeast Asia
Asia	Sri Lanka	Southeast Asia
Asia	Maldives	Southeast Asia
Asia	Myanmar	Southeast Asia
Asia	Malaysia	Southeast Asia
Asia	Philippines	Southeast Asia
Asia	Thailand	Southeast Asia
Asia	Timor-Leste	Southeast Asia
Asia	Viet Nam	Southeast Asia
Asia	Cyprus	Western Europe
Asia	Israel	Western Europe
Europe	Albania	Central Europe
Europe	Bulgaria	Central Europe
Europe	Bosnia and Herzegovina	Central Europe
Europe	Czech Republic	Central Europe
Europe	Croatia	Central Europe
Europe	Hungary	Central Europe
Europe	Macedonia (TFYR)	Central Europe
Europe	Montenegro	Central Europe
Europe	Poland	Central Europe
Europe	Romania	Central Europe
Europe	Serbia	Central Europe
Europe	Slovakia	Central Europe
Europe	Slovenia	Central Europe
Europe	Belarus	Eastern Europe
Europe	Estonia	Eastern Europe
Europe	Lithuania	Eastern Europe
Europe	Latvia	Eastern Europe
Europe	Moldova	Eastern Europe
Europe	Russian Federation	Eastern Europe
Europe	Ukraine	Eastern Europe
Europe	Andorra	Western Europe
Europe	Austria	Western Europe
Europe	Belgium	Western Europe
Europe	Switzerland	Western Europe
Europe	Germany	Western Europe
Europe	Denmark	Western Europe
Europe	Spain	Western Europe
Europe	Finland	Western Europe
Europe	France	Western Europe
Europe	United Kingdom	Western Europe
Europe	Greece	Western Europe
Europe	Ireland	Western Europe
Europe	Iceland	Western Europe
Europe	Italy	Western Europe
Europe	Luxembourg	Western Europe
Europe	Malta	Western Europe
Europe	Netherlands	Western Europe
Europe	Norway	Western Europe
Europe	Portugal	Western Europe
Europe	Sweden	Western Europe
Oceania	Australia	Australasia
Oceania	New Zealand	Australasia
Oceania	American Samoa	Oceania
Oceania	Fiji	Oceania
Oceania	Guam	Oceania
Oceania	Micronesia (Federated States of)	Oceania
Oceania	Kiribati	Oceania
Oceania	Marshall Islands	Oceania
Oceania	Northern Mariana Islands	Oceania
Oceania	Papua New Guinea	Oceania
Oceania	Solomon Islands	Oceania
Oceania	Tonga	Oceania
Oceania	Vanuatu	Oceania
Oceania	Samoa	Oceania

4) Supplementary statistical methods

Assessment of calibration within the derivation dataset

Mean predicted and observed 10-year risks were calculated and compared within deciles of predicted risk for each sex specific CHD and Stroke model separately using studies that had at least 10-years follow-up. Predicted risks were estimated using the fitted cox model using the study specific baseline survival estimates at 10-years and common hazard ratios as presented in **Table 2** and **Table 1.5**. Mean observed 10-year CVD risk within each decile and study ($\theta_{d,s}$) for decile d and study s in 1:S), was obtained using the following general Cox model:

$$S(t|d,s) = S_{0,s}(t)^{\exp(\beta' d)}$$

where β is a vector, and d a set of dummy variables representing the deciles, $S(t|d,s)$ is the probability of not having a CVD event by time t given category d and study s, and $S_{0,s}(t)$ is the baseline survival function for study s. Observed decile- and study-specific 10-year CVD risk was then estimated using the following translation:

$$\hat{\theta}_{d,s} = 1 - \hat{S}(10|d,s) = 1 - \hat{S}_{0,s}(10)^{\exp(\beta' d)}$$

This method assumes proportional hazards across deciles, and the process was completed separately for men and women. Study-specific estimates of observed risk in each decile $\hat{\theta}_{d,s}$ were pooled across studies weighting by the number of study and decile-specific contributing events:

$$\widehat{\theta_d} = \frac{\sum_s w_{d,s}\, \hat{\theta}_{d,s}}{\sum_s w_{d,s}}$$

Study and decile-specific predicted risks were similarly pooled and calibration was assessed visually using plots of the pooled predicted and observed risks, and formally by quantifying goodness of fit as proposed by Parzen and Lipsitz (Biometrics 1999;55:580-4).

Methods used for recalibration

Recalibration of the core models was completed separately for each target country, sex and endpoint (MI and stroke) using the general process described in **Figure 1.3**. This involved the use of country-sex-specific mean risk factor levels (from NCDRisc) and country-sex-specific estimates of annual incidence of MI and stroke events within 5-year age groups, from GBD 2017. We used the core WHO risk models to estimate 10-year predicted risk of each endpoint for each of the age groups using the mean risk factor values (**Table 4.1** shows a worked example for the laboratory based MI and Stroke models for a single age group 40-45). We then calculated expected 10-year risk (p_{10}) for each age group based on annual incidence using the following

relation, where IR_{mid} is the incidence for the mid-point of the 10 year interval ahead i.e. for the 40 to 44 year age-group the rate for 45 to 49 years was used (**Table 4.1**):

Expected 10-year risk: p_{10} = 1 - exp$(-IR_{mid} \times 10)$. (1)

Having completed this process for each age group, as shown in **Figure 1.3** we then regressed transformed expected 10-year risk across age groups on that predicted by the WHO risk models to derive recalibration factors (the intercept and slope of the resulting regression line). The WHO risk models, rescaled using the recalibration factors were then used to estimate appropriate risks for each potential risk factor combination. Finally for construction of region specific 10-year CVD risk charts, the separately recalibrated risks of MI and stroke were combined using **Equation 2** for each risk factor combination, where p_{MI}, p_{stroke} and p_{cvd} are the probabilities of having a MI, stroke or CVD (MI or stroke) event over the next 10-years.

$$p_{cvd} = 1 - (1 - p_{MI}) * (1 - p_{stroke}) \quad (2)$$

We completed the following sensitivity analyses regarding the recalibration procedure: 1) Rather than using a single incidence rate to estimate expected risk over 10-years for each age group (**Equation 1**) we interpolated GBD rates obtained from the IHME to get the expected incidence at the midpoint of each 1-year risk period (say $IR_{mid,j}$) and estimated expected 10-year risk as one minus probability of surviving each subsequent 1-year interval: $p_{10} = 1 - \prod_{j=1}^{j=10} \exp(-IR_{mid,j})$; 2) We derived a single risk model to estimate 10-year CVD risk and recalibrated this directly to CVD rates (MI + stroke incidence) rather than allowing separate models for MI and stroke components and recalibration to individual MI or stroke rates.

Table 4.1 Example calculation of predicted and observed 10-year risk of MI and stroke for a single age group based on aggregate level data

	Calculation of predicted risk using laboratory based models				Calculation of observed risk	
	**Mean or prevalence of risk factor values for age group 40-45**	**logHRs from ERFC fitted model**		**Calculation of risk using ERFC model**	**Annual incidence for age group 45-50**	**Expected 10-year risk for the 40-45 year age group**
MI	Age = 42.5	Age	0.0719	$\sum \beta(x - x_{cen})$ = 0.0719 x (42.5-60)	Annual incidence per	Expected risk = 1-exp(-0.0045*10)
	Total cholesterol = 5.5mmol/l	Total cholesterol	0.2285	+ 0.2285 x (5.5-6)	100000 person	= 1-0.9560
	SBP = 125mmHg	SBP	0.0132	+ 0.0132 x (125-120)	years=450	= 0.0440
	Diabetes=0.1	Diabetes	0.6410	+ 0.6410 x 0.1		= 4.4%
	Smoking=0.3	Smoking	0.5638	+ 0.5638 x 0.3	Incidence per 1 person	
		T. cholesterol interaction with age	-0.0046	-0.0046 x (42.5-60) x (5.5-6)	year: 0.0045	
		SBP interaction with age	-0.0002	-0.0002 x (42.5-60) x (125-120)		
		Diabetes interaction with age	-0.0125	-0.0125 x (42.5-60) x 0.1		
		Smoking interaction with age	-0.0183	-0.0183 x (42.5-60) x 0.3		
				= -1.0946		
				10-yr risk = 1-0.9540^exp($\sum \beta \, (x - x_{cen})$)		
				= 1-0.9540^exp(-1.0946)		
				= 0.0156= 1.6%		
Stroke	Age = 42.5	Age	0.0987	$\sum \beta(x - x_{cen})$ = 0.0987 x (42.5-60)	Annual incidence per	Expected risk = 1-exp(-0.0037*10)
	Total cholesterol = 5.5mmol/l	Total cholesterol	0.0295	+ 0.0295 x (5.5-6)	100000 person	= 1-0.9637
	SBP = 125mmHg	SBP	0.0223	+ 0.0223 x (125-120)	years=370	= 0.0363
	Diabetes=0.1	Diabetes	0.6269	+ 0.6269 x 0.1		= 3.6%
	Smoking=0.3	Smoking	0.4981	+ 0.4981 x 0.3	Incidence per 1 person	
		T. cholesterol interaction with age	0.0014	-0.0014 x (42.5-60) x (5.5-6)	year: 0.0037	
		SBP interaction with age	-0.0004	-0.0004 x (42.5-60) x (125-120)		
		Diabetes interaction with age	-0.0263	-0.0263 x (42.5-60) x 0.1		
		Smoking interaction with age	-0.0151	-0.0151 x (42.5-60) x 0.3		
				= -1.2703		
				10-yr risk = 1-0.9849^exp($\sum \beta \, (x - x_{cen})$)		
				= 1-0.9849^exp(-1.2703)		
				= 0.0042= 0.4%		

5) Endpoint definitions used for GBD estimated incidence rates

MI

Case definitions:

1) Acute myocardial infarction (MI): Definite and possible MI according to the third universal definition of myocardial infarction:
    a. When there is clinical evidence of myocardial necrosis in a clinical setting consistent with myocardial ischemia or
    b. Detection of a rise and/or fall of cardiac biomarker values and with at least one of the following: i) symptoms of ischaemia, ii) new or presumed new ST-segment-T wave changes or new left bundle branch block, iii) development of pathological Q waves in the ECG, iv) imaging evidence of new loss of viable myocardium or new regional wall motion abnormality, or v) identification of an intracoronary thrombus by angiography or autopsy.
    c. Sudden (abrupt) unexplained cardiac death, involving cardiac arrest or no evidence of a noncoronary cause of death
    d. Prevalent MI is considered to last from the onset of the event to 28 days after the event and is divided into an acute phase (0–2 days) and subacute (3–28 days).

Stroke

Case definition

Stroke was defined according to WHO criteria – rapidly developing clinical signs of focal (at times global) disturbance of cerebral function lasting more than 24 hours or leading to death with no apparent cause other than that of vascular origin(1). Data on transient ischaemic attack (TIA) were not included.

Acute stroke: Stroke cases are considered acute from the day of incidence of a first-ever stroke through day 28 following the event.

Ischaemic stroke: an episode of neurological dysfunction caused by focal cerebral, spinal, or retinal infarction

Intracerebral haemorrhage: a focal collection of blood within the brain parenchyma or ventricular system that is not caused by trauma

Reference

Definitions are taken from Supplement 1 to: GBD 2017 Disease and Injury Incidence and Prevalence Collaborators. Global, regional, and national incidence, prevalence, and years lived with disability for 354 diseases and injuries for 195 countries and territories, 1990–2017: a systematic analysis for the Global Burden of Disease Study 2017. Lancet 2018; 392:1789–858

6) TRIPOD checklist for prediction model development and validation

Section/Topic	Item		Checklist Item	Page
**Title and abstract**				
Title	1	D;V	Identify the study as developing and/or validating a multivariable prediction model, the target population, and the outcome to be predicted.	1
Abstract	2	D;V	Provide a summary of objectives, study design, setting, participants, sample size, predictors, outcome, statistical analysis, results, and conclusions.	2
**Introduction**				
Background and objectives	3a	D;V	Explain the medical context (including whether diagnostic or prognostic) and rationale for developing or validating thet multivariable prediction model, including references to existing models.	3
	3b	D;V	Specify the objectives, including whether the study describes the development or validation of the model or both.	3
**Methods**				
Source of data	4a	D;V	Describe the study design or source of data (e.g., randomized trial, cohort, or registry data), separately for the development and validation data sets, if applicable.	4-5
	4b	D;V	Specify the key study dates, including start of accrual; end of accrual; and, if applicable, end of follow-up.	4, 8 and suppl tables 1, 2 and 4
Participants	5a	D;V	Specify key elements of the study setting (e.g., primary care, secondary care, general population) including number and location of centres.	4, 8 and suppl tables 1, 2 and 4
	5b	D;V	Describe eligibility criteria for participants.	4, 5 and eFig 1
	5c	D;V	Give details of treatments received, if relevant.	n/a
Outcome	6a	D;V	Clearly define the outcome that is predicted by the prediction model, including how and when assessed.	5 and Table 1.3
	6b	D;V	Report any actions to blind assessment of the outcome to be predicted.	n/a
Predictors	7a	D;V	Clearly define all predictors used in developing or validating the multivariable prediction model, including how and when they were measured.	4 and tables 1,2 and etables 1,2 and 4
	7b	D;V	Report any actions to blind assessment of predictors for the outcome and other predictors.	n/a
Sample size	8	D;V	Explain how the study size was arrived at.	n/a
Missing data	9	D;V	Describe how missing data were handled (e.g., complete-case analysis, single imputation, multiple imputation) with details of any imputation method.	12
Statistical analysis methods	10a	D	Describe how predictors were handled in the analyses.	5, 6 and table 2
	10b	D	Specify type of model, all model-building procedures (including any predictor selection), and method for internal validation.	5, 6
	10c	V	For validation, describe how the predictions were calculated.	6/7, table 2, eFig 3 and Appendix 3
	10d	D;V	Specify all measures used to assess model performance and, if relevant, to compare multiple models.	6, 7
	10e	V	Describe any model updating (e.g., recalibration) arising from the validation, if done.	6, efig 2 and appendix 3
Risk groups	11	D;V	Provide details on how risk groups were created, if done.	6, 7
Development vs. validation	12	V	For validation, identify any differences from the development data in setting, eligibility criteria, outcome, and predictors.	4/5, Table 1.1,2,3 and 4
**Results**				
Participants	13a	D;V	Describe the flow of participants through the study, including the number of participants with and without the outcome and, if applicable, a summary of the follow-up time. A diagram may be helpful.	8, Fig 1 table 1, etables 1,2 and 4
	13b	D;V	Describe the characteristics of the participants (basic demographics, clinical features, available predictors), including the number of participants with missing data for predictors and outcome.	8,, table 1 and etables 1, 2, and 4
	13c	V	For validation, show a comparison with the development data of the distribution of important variables (demographics, predictors and outcome).	etables 1, 2, 4 and 7
Model development	14a	D	Specify the number of participants and outcome events in each analysis.	8,, Fig 1, table 1 and etables 1, 2, and 4
	14b	D	If done, report the unadjusted association between each candidate predictor and outcome.	n/a
	15a	D	Present the full prediction model to allow predictions for individuals (i.e., all regression coefficients, and model intercept or baseline survival at a given time point).	Table 2, Table 1.5

Model specification				and appendix 3
	15b	D	Explain how to the use the prediction model.	Table 2, figure 2, efig2 and Appendix 3
Model performance	16	D;V	Report performance measures (with CIs) for the prediction model.	8/9, fig 5, Table 1.6 and efigs 4,5, 17-20
Model-updating	17	V	If done, report the results from any model updating (i.e., model specification, model performance).	8, fig 3, efigs 2,and 17-20
**Discussion**				
Limitations	18	D;V	Discuss any limitations of the study (such as nonrepresentative sample, few events per predictor, missing data).	12
Interpretation	19a	V	For validation, discuss the results with reference to performance in the development data, and any other validation data.	8-11
	19b	D;V	Give an overall interpretation of the results, considering objectives, limitations, results from similar studies, and other relevant evidence.	10, 11, 12
Implications	20	D;V	Discuss the potential clinical use of the model and implications for future research.	10, 11,12
**Other information**				
Supplementary information	21	D;V	Provide information about the availability of supplementary resources, such as study protocol, Web calculator, and data sets.	Throughout paper
Funding	22	D;V	Give the source of funding and the role of the funders for the present study.	12, 7

*Items relevant only to the development of a prediction model are denoted by D, items relating solely to a validation of a prediction model are denoted by V, and items relating to both are denoted D;V. We recommend using the TRIPOD Checklist in conjunction with the TRIPOD Explanation and Elaboration document.
