# Supplementary material for: World Health Organization cardiovascular disease risk charts: revised models to estimate risk in 21 global regions
Source: Lancet Glob Health. 2019 Sep 2;7(10):e1332–45. doi: 10.1016/S2214-109X(19)30318-3 (PMC7025029; doi:10.1016/S2214-109X(19)30318-3)
Supplement: Supplementary appendix 2 [file mmc2.pdf]

THE LANCET
Global Health

Supplementary appendix 2

This appendix formed part of the original submission and has been peer reviewed. We post it as supplied by the authors.

Supplement to: The WHO CVD Risk Chart Working Group. World Health Organization cardiovascular disease risk charts: revised models to estimate risk in 21 global regions. *Lancet Glob Health* 2019; published online Sept 2. http://dx.doi.org/10.1016/S2214-109X(19)30318-3.

WHO cardiovascular disease risk laboratory-based charts

North Africa and Middle East

Risk Level: <5% | 5% to <10% | 10% to <20% | 20% to <30% | ≥30%

People without Diabetes

Age (years)	SBP (mmHg)	Men Non-smoker <4	4-4.9	5-5.9	6-6.9	≥ 7	Men Smoker <4	4-4.9	5-5.9	6-6.9	≥ 7	Women Non-smoker <4	4-4.9	5-5.9	6-6.9	≥ 7	Women Smoker <4	4-4.9	5-5.9	6-6.9	≥ 7
70-74	≥180	37	40	43	46	50	45	48	52	56	60	29	31	32	33	34	39	40	41	43	45
	160-179	31	33	36	40	43	38	41	45	48	52	26	27	28	29	30	34	35	36	38	39
	140-159	26	28	31	34	37	32	35	38	42	45	22	23	24	25	26	30	31	32	33	35
	120-139	21	23	26	28	31	27	29	32	35	39	19	20	21	22	23	26	27	28	29	30
	<120	18	19	21	24	26	22	24	27	30	33	16	17	18	19	20	22	23	24	25	26
65-69	≥180	29	32	35	38	42	38	41	45	49	54	23	25	26	27	29	33	35	36	38	40
	160-179	24	26	29	32	35	31	35	38	42	46	20	21	22	23	24	29	30	31	33	35
	140-159	19	21	24	26	29	26	29	32	35	39	17	18	19	20	21	24	26	27	28	30
	120-139	16	17	19	22	24	21	23	26	29	32	14	15	16	17	18	21	22	23	24	26
	<120	13	14	16	18	20	17	19	21	24	27	12	13	13	14	15	18	19	20	21	22
60-64	≥180	23	25	28	31	35	32	35	39	43	48	19	20	21	22	23	28	30	32	34	36
	160-179	18	20	23	25	29	26	29	32	36	40	15	16	17	18	20	24	25	27	29	30
	140-159	14	16	18	20	23	21	23	26	29	33	13	14	14	15	16	20	21	23	24	26
	120-139	11	13	14	16	19	16	19	21	24	27	11	11	12	13	14	17	18	19	20	22
	<120	9	10	11	13	15	13	15	17	19	22	9	9	10	11	11	14	15	16	17	18
55-59	≥180	18	20	22	25	29	27	30	34	38	42	15	16	17	18	19	24	26	28	30	32
	160-179	14	16	18	20	23	21	24	27	30	35	12	13	14	15	16	20	21	23	25	27
	140-159	11	12	14	16	18	16	19	21	24	28	10	10	11	12	13	16	18	19	20	22
	120-139	8	9	11	12	14	13	15	17	19	22	8	8	9	10	11	13	14	16	17	18
	<120	6	7	8	10	11	10	11	13	15	18	6	7	7	8	9	11	12	13	14	15
50-54	≥180	14	16	18	20	23	22	25	29	33	37	11	12	13	14	16	20	22	24	26	28
	160-179	10	12	14	16	18	17	19	22	26	30	9	10	11	12	13	17	18	19	21	23
	140-159	8	9	10	12	14	13	15	17	20	24	7	8	9	9	10	13	14	16	17	19
	120-139	6	7	8	9	11	10	11	13	16	18	6	6	7	7	8	11	12	13	14	15
	<120	4	5	6	7	8	7	9	10	12	14	5	5	5	6	7	9	9	10	11	13
45-49	≥180	11	12	14	16	19	19	21	24	28	33	9	10	11	12	13	17	19	21	23	25
	160-179	8	9	10	12	14	14	16	18	22	25	7	8	8	9	10	14	15	16	18	20
	140-159	6	7	8	9	11	10	12	14	17	20	5	6	7	7	8	11	12	13	15	16
	120-139	4	5	6	7	8	8	9	11	13	15	4	5	5	6	6	9	9	10	12	13
	<120	3	4	4	5	6	6	7	8	10	12	3	4	4	4	5	7	7	8	9	10
40-44	≥180	8	9	11	13	15	15	18	20	24	28	7	8	8	9	10	15	16	18	20	22
	160-179	6	7	8	9	11	11	13	15	18	22	5	6	6	7	8	11	13	14	16	17
	140-159	4	5	6	7	8	8	9	11	14	16	4	4	5	6	6	9	10	11	12	14
	120-139	3	4	4	5	6	6	7	8	10	12	3	3	4	4	5	7	8	9	10	11
	<120	2	3	3	4	5	4	5	6	8	9	2	3	3	3	4	5	6	7	8	9

Total cholesterol (mmol/l)

North Africa and Middle East

Risk Level: <5% | 5% to <10% | 10% to <20% | 20% to <30% | ≥30%

People with Diabetes

Age (years)	SBP (mmHg)	Men Non-smoker <4	4-4.9	5-5.9	6-6.9	≥ 7	Men Smoker <4	4-4.9	5-5.9	6-6.9	≥ 7	Women Non-smoker <4	4-4.9	5-5.9	6-6.9	≥ 7	Women Smoker <4	4-4.9	5-5.9	6-6.9	≥ 7
70-74	≥180	47	51	55	59	63	57	61	65	69	73	41	42	44	45	47	52	54	55	57	59
	160-179	40	44	48	52	56	49	53	57	61	66	36	37	39	40	42	46	48	50	51	53
	140-159	34	37	41	44	48	42	46	50	54	58	31	33	34	35	37	41	42	44	46	47
	120-139	29	31	35	38	42	36	39	43	47	51	27	28	30	31	32	36	37	39	40	42
	<120	24	26	29	32	35	30	33	36	40	44	24	25	26	27	28	31	33	34	35	37
65-69	≥180	40	44	48	52	57	51	56	60	65	70	35	36	38	40	42	48	50	52	54	56
	160-179	34	37	40	45	49	43	48	52	56	61	30	31	33	35	36	42	43	45	48	50
	140-159	28	31	34	37	41	36	40	44	48	53	26	27	28	30	31	36	38	40	42	44
	120-139	23	25	28	31	35	30	33	37	41	46	22	23	24	26	27	31	33	34	36	38
	<120	18	21	23	26	29	25	28	31	34	38	19	20	21	22	23	27	28	30	31	33
60-64	≥180	34	38	42	46	51	46	51	55	61	66	29	31	33	35	37	43	46	48	51	53
	160-179	28	31	34	38	42	38	42	47	52	57	25	26	28	30	31	37	39	42	44	47
	140-159	22	25	28	31	35	31	35	39	43	48	21	22	23	25	27	32	34	36	38	40
	120-139	18	20	22	25	29	25	28	32	36	41	17	18	20	21	23	27	29	30	32	35
	<120	14	16	18	21	23	20	23	26	30	34	14	15	16	18	19	23	24	26	28	30
55-59	≥180	29	32	36	40	45	42	46	51	56	62	25	26	28	30	32	40	42	45	47	51
	160-179	23	25	29	32	37	34	38	42	47	53	20	22	23	25	27	33	36	38	41	43
	140-159	18	20	23	26	30	27	30	34	39	44	17	18	19	21	23	28	30	32	34	37
	120-139	14	16	18	21	24	21	24	27	31	36	14	15	16	17	19	23	25	27	29	31
	<120	11	12	14	16	19	17	19	22	25	29	11	12	13	14	15	19	21	22	24	26
50-54	≥180	24	27	30	34	39	37	42	47	52	58	21	22	24	26	28	36	38	41	44	48
	160-179	18	21	24	27	31	29	33	38	43	48	17	18	20	21	23	30	32	35	37	40
	140-159	14	16	18	21	25	23	26	30	34	40	13	15	16	17	19	24	26	29	31	34
	120-139	11	12	14	16	19	18	20	23	27	32	11	12	13	14	15	20	22	24	26	28
	<120	8	9	11	13	15	13	16	18	21	25	9	9	10	11	13	16	18	19	21	23
45-49	≥180	20	23	26	30	34	33	38	42	48	55	17	19	21	23	25	32	35	38	41	45
	160-179	15	17	20	23	27	26	29	33	38	44	14	15	16	18	20	26	29	31	34	38
	140-159	11	13	15	17	21	19	22	26	30	36	11	12	13	14	16	21	23	26	28	31
	120-139	8	10	11	13	16	15	17	20	24	28	8	9	10	11	13	17	19	21	23	26
	<120	6	7	8	10	12	11	13	15	18	22	7	7	8	9	10	14	15	17	19	21
40-44	≥180	17	19	22	25	30	30	34	39	44	51	15	16	18	19	21	29	32	35	39	42
	160-179	12	14	16	19	22	22	26	30	35	41	11	12	14	15	17	23	26	28	31	35
	140-159	9	10	12	14	17	16	19	22	27	32	9	10	11	12	13	18	20	23	25	28
	120-139	6	7	9	10	13	12	14	17	20	25	7	7	8	9	10	14	16	18	20	23
	<120	5	5	6	8	10	9	10	13	15	19	5	6	6	7	8	11	13	14	16	19

Total cholesterol (mmol/l)

Central Sub-Saharan Africa

Risk Level: <5% | 5% to <10% | 10% to <20% | 20% to <30% | ≥30%

People without Diabetes

Age (years)	SBP (mmHg)	Men										Women									
		Non-smoker					Smoker					Non-smoker					Smoker				
		<4	4-4.9	5-5.9	6-6.9	≥ 7	<4	4-4.9	5-5.9	6-6.9	≥ 7	<4	4-4.9	5-5.9	6-6.9	≥ 7	<4	4-4.9	5-5.9	6-6.9	≥ 7
70-74	≥180	23	25	27	30	33	29	31	34	37	40	21	22	23	25	26	30	31	33	35	37
	160-179	19	21	23	25	27	24	26	28	31	34	18	19	20	21	22	25	27	28	30	31
	140-159	16	17	19	21	23	20	22	24	26	29	15	15	16	17	18	21	22	24	25	26
	120-139	13	14	15	17	19	16	18	20	22	24	12	13	13	14	15	18	19	20	21	22
	<120	10	11	13	14	15	13	15	16	18	20	10	11	11	12	13	15	15	16	17	18
65-69	≥180	18	20	22	24	27	24	26	29	32	35	16	17	18	19	20	25	26	28	30	32
	160-179	14	16	18	19	22	19	21	24	26	29	13	14	15	16	17	20	22	23	25	26
	140-159	12	13	14	16	18	16	17	19	21	24	10	11	12	13	14	17	18	19	20	22
	120-139	9	10	11	13	14	13	14	16	17	20	8	9	10	10	11	13	14	15	17	18
	<120	7	8	9	10	11	10	11	13	14	16	7	7	8	8	9	11	12	13	14	15
60-64	≥180	14	15	17	19	21	20	22	25	27	31	12	13	14	15	16	20	22	23	25	27
	160-179	11	12	14	15	17	16	18	20	22	25	9	10	11	12	13	16	17	19	20	22
	140-159	9	9	11	12	14	12	14	16	18	20	7	8	9	9	10	13	14	15	16	18
	120-139	7	7	8	9	11	10	11	12	14	16	6	6	7	7	8	10	11	12	13	14
	<120	5	6	7	7	8	8	9	10	11	13	5	5	5	6	6	8	9	10	11	12
55-59	≥180	11	12	13	15	17	16	18	21	23	27	9	9	10	11	12	16	18	19	21	23
	160-179	8	9	10	12	13	13	14	16	18	21	7	7	8	9	10	13	14	15	17	19
	140-159	6	7	8	9	10	10	11	13	14	17	5	6	6	7	7	10	11	12	13	15
	120-139	5	5	6	7	8	7	8	10	11	13	4	4	5	5	6	8	9	9	10	12
	<120	4	4	5	5	6	6	7	7	9	10	3	3	4	4	5	6	7	7	8	9
50-54	≥180	8	9	11	12	14	14	15	17	20	23	6	7	8	9	9	13	15	16	18	20
	160-179	6	7	8	9	11	10	12	13	15	18	5	5	6	6	7	10	11	12	14	16
	140-159	5	5	6	7	8	8	9	10	12	14	4	4	4	5	5	8	9	10	11	12
	120-139	3	4	4	5	6	6	7	8	9	10	3	3	3	4	4	6	7	7	8	9
	<120	3	3	3	4	5	4	5	6	7	8	2	2	3	3	3	4	5	6	6	7
45-49	≥180	6	7	8	9	11	11	13	15	17	20	5	5	6	7	7	11	12	13	15	17
	160-179	5	5	6	7	8	8	9	11	13	15	4	4	4	5	5	8	9	10	11	13
	140-159	3	4	4	5	6	6	7	8	9	11	3	3	3	4	4	6	7	8	9	10
	120-139	2	3	3	4	5	4	5	6	7	8	2	2	2	3	3	4	5	6	6	7
	<120	2	2	2	3	3	3	4	4	5	6	1	2	2	2	2	3	4	4	5	6
40-44	≥180	5	6	6	7	9	9	10	12	14	17	4	4	4	5	6	9	10	11	13	14
	160-179	4	4	5	5	6	7	8	9	10	13	3	3	3	4	4	6	7	8	9	11
	140-159	2	3	3	4	5	5	5	6	8	9	2	2	2	3	3	5	5	6	7	8
	120-139	2	2	2	3	3	3	4	5	6	7	1	1	2	2	2	3	4	4	5	6
	<120	1	1	2	2	2	2	3	3	4	5	1	1	1	1	2	2	3	3	4	4

Total cholesterol (mmol/l)

Central Sub-Saharan Africa

Risk Level: <5% | 5% to <10% | 10% to <20% | 20% to <30% | ≥30%

People with Diabetes

Age (years)	SBP (mmHg)	Men										Women									
		Non-smoker					Smoker					Non-smoker					Smoker				
		<4	4-4.9	5-5.9	6-6.9	≥ 7	<4	4-4.9	5-5.9	6-6.9	≥ 7	<4	4-4.9	5-5.9	6-6.9	≥ 7	<4	4-4.9	5-5.9	6-6.9	≥ 7
70-74	≥180	31	33	36	40	43	38	41	45	48	52	32	34	36	38	40	44	47	49	51	54
	160-179	25	28	30	33	37	32	35	38	41	45	27	29	30	32	34	38	40	42	44	47
	140-159	21	23	25	28	31	27	29	32	35	39	23	24	25	27	28	32	34	36	38	40
	120-139	17	19	21	23	26	22	24	27	30	33	19	20	21	23	24	27	29	31	32	34
	<120	14	16	17	19	21	18	20	22	25	27	16	17	18	19	20	23	24	26	27	29
65-69	≥180	26	28	31	34	38	34	37	40	44	49	26	28	30	32	34	40	42	45	47	50
	160-179	21	23	25	28	31	28	31	34	37	41	21	23	24	26	28	33	35	38	40	43
	140-159	17	19	21	23	26	23	25	28	31	35	18	19	20	22	23	28	29	32	34	36
	120-139	13	15	17	19	21	18	20	23	26	29	14	15	16	18	19	23	24	26	28	30
	<120	11	12	14	15	17	15	17	19	21	23	12	13	13	14	16	19	20	22	23	25
60-64	≥180	21	24	26	29	33	30	33	37	41	45	21	23	24	26	29	35	38	40	44	47
	160-179	17	19	21	24	27	24	27	30	33	38	17	18	20	21	23	29	31	34	36	39
	140-159	13	15	17	19	21	19	21	24	27	31	14	15	16	17	19	23	25	27	30	33
	120-139	10	12	13	15	17	15	17	19	22	25	11	12	13	14	15	19	20	22	24	27
	<120	8	9	10	12	14	12	14	15	18	20	9	9	10	11	12	15	17	18	20	22
55-59	≥180	18	20	22	25	28	27	30	33	37	42	17	18	20	22	24	31	34	37	40	44
	160-179	14	15	17	20	22	21	23	26	30	34	13	14	16	17	19	25	27	30	33	36
	140-159	11	12	13	15	18	16	18	21	24	27	10	11	12	14	15	20	22	24	26	29
	120-139	8	9	10	12	14	13	14	16	19	22	8	9	10	11	12	16	17	19	21	23
	<120	6	7	8	9	11	10	11	13	15	17	6	7	8	8	9	12	14	15	17	19
50-54	≥180	15	17	19	21	24	24	26	30	34	39	14	15	16	18	20	27	30	33	37	41
	160-179	11	13	14	16	19	18	20	23	27	31	10	11	13	14	16	21	24	26	29	33
	140-159	8	9	11	12	15	14	16	18	21	24	8	9	10	11	12	17	18	21	23	26
	120-139	6	7	8	9	11	10	12	14	16	19	6	7	7	8	9	13	14	16	18	21
	<120	5	5	6	7	8	8	9	11	12	15	5	5	6	6	7	10	11	12	14	16
45-49	≥180	12	14	16	18	21	21	24	27	31	36	11	12	13	15	17	24	27	30	33	38
	160-179	9	10	12	13	16	16	18	20	24	28	8	9	10	11	13	18	20	23	26	30
	140-159	7	7	9	10	12	12	13	15	18	21	6	7	8	8	10	14	16	18	20	23
	120-139	5	5	6	7	9	9	10	12	14	16	4	5	6	6	7	10	12	14	16	18
	<120	3	4	5	6	7	6	7	9	10	12	3	4	4	5	5	8	9	10	12	14
40-44	≥180	10	12	13	15	18	19	21	24	28	33	9	10	11	12	14	21	24	27	30	35
	160-179	7	8	10	11	13	14	15	18	21	25	6	7	8	9	10	16	18	20	23	27
	140-159	5	6	7	8	10	10	11	13	16	19	5	5	6	7	8	12	13	15	18	21
	120-139	4	4	5	6	7	7	8	10	12	14	3	4	4	5	6	9	10	11	13	16
	<120	3	3	4	4	5	5	6	7	9	11	2	3	3	4	4	6	7	9	10	12

Total cholesterol (mmol/l)

Eastern Sub-Saharan Africa

Risk Level: <5% | 5% to <10% | 10% to <20% | 20% to <30% | ≥30%

People without Diabetes

Age (years)	SBP (mmHg)	Men Non-smoker <4	4-4.9	5-5.9	6-6.9	≥ 7	Men Smoker <4	4-4.9	5-5.9	6-6.9	≥ 7	Women Non-smoker <4	4-4.9	5-5.9	6-6.9	≥ 7	Women Smoker <4	4-4.9	5-5.9	6-6.9	≥ 7
70-74	≥180	22	24	27	29	32	28	30	33	36	39	16	17	17	18	19	22	23	24	25	26
	160-179	18	20	22	24	26	23	25	28	30	33	14	14	15	15	16	19	20	20	21	22
	140-159	15	17	18	20	22	19	21	23	25	28	12	12	13	13	14	16	17	17	18	19
	120-139	12	14	15	16	18	16	17	19	21	23	10	10	11	11	12	14	14	15	15	16
	<120	10	11	12	14	15	13	14	16	17	19	8	9	9	9	10	12	12	12	13	14
65-69	≥180	18	19	21	23	26	23	26	28	31	34	12	13	14	14	15	19	19	20	21	23
	160-179	14	16	17	19	21	19	21	23	25	28	10	11	11	12	13	15	16	17	18	19
	140-159	11	12	14	15	17	15	17	19	21	23	9	9	9	10	10	13	13	14	15	16
	120-139	9	10	11	12	14	12	14	15	17	19	7	7	8	8	9	11	11	12	12	13
	<120	7	8	9	10	11	10	11	12	14	15	6	6	6	7	7	9	9	10	10	11
60-64	≥180	14	15	17	19	21	19	21	24	27	30	10	10	11	11	12	15	16	17	18	20
	160-179	11	12	13	15	17	15	17	19	21	24	8	8	9	9	10	13	13	14	15	16
	140-159	8	9	10	12	13	12	13	15	17	19	6	7	7	8	8	10	11	12	12	13
	120-139	7	7	8	9	10	9	11	12	14	15	5	5	6	6	7	8	9	9	10	11
	<120	5	6	6	7	8	7	8	9	11	12	4	4	5	5	5	7	7	8	8	9
55-59	≥180	11	12	13	15	17	16	18	20	23	26	7	8	8	9	10	13	14	15	16	17
	160-179	8	9	10	11	13	12	14	16	18	20	6	6	7	7	8	10	11	12	13	14
	140-159	6	7	8	9	10	10	11	12	14	16	5	5	5	6	6	8	9	9	10	11
	120-139	5	5	6	7	8	7	8	9	11	13	4	4	4	5	5	6	7	8	8	9
	<120	4	4	5	5	6	6	6	7	8	10	3	3	3	4	4	5	6	6	7	7
50-54	≥180	8	9	10	12	13	13	15	17	19	22	6	6	7	7	8	11	12	12	14	15
	160-179	6	7	8	9	10	10	11	13	15	17	4	5	5	6	6	8	9	10	11	12
	140-159	5	5	6	7	8	8	9	10	11	13	3	4	4	4	5	6	7	8	8	9
	120-139	3	4	4	5	6	6	6	7	9	10	3	3	3	3	4	5	5	6	7	7
	<120	3	3	3	4	5	4	5	6	7	8	2	2	2	3	3	4	4	5	5	6
45-49	≥180	6	7	8	9	11	11	12	14	16	19	4	5	5	6	6	9	10	11	12	13
	160-179	5	5	6	7	8	8	9	11	12	14	3	4	4	4	5	7	7	8	9	10
	140-159	3	4	4	5	6	6	7	8	9	11	2	3	3	3	4	5	6	6	7	8
	120-139	2	3	3	4	4	4	5	6	7	8	2	2	2	2	3	4	4	5	5	6
	<120	2	2	2	3	3	3	4	4	5	6	1	2	2	2	2	3	3	4	4	5
40-44	≥180	5	6	6	7	9	9	10	12	14	16	3	4	4	4	5	7	8	9	10	11
	160-179	3	4	4	5	6	6	7	9	10	12	2	3	3	3	4	5	6	7	8	8
	140-159	2	3	3	4	5	5	5	6	7	9	2	2	2	2	3	4	5	5	6	6
	120-139	2	2	2	3	3	3	4	5	5	7	1	1	2	2	2	3	3	4	4	5
	<120	1	1	2	2	2	2	3	3	4	5	1	1	1	1	2	2	3	3	3	4

Total cholesterol (mmol/l)

Eastern Sub-Saharan Africa

Risk Level: <5% | 5% to <10% | 10% to <20% | 20% to <30% | ≥30%

People with Diabetes

Age (years)	SBP (mmHg)	Men Non-smoker <4	4-4.9	5-5.9	6-6.9	≥ 7	Men Smoker <4	4-4.9	5-5.9	6-6.9	≥ 7	Women Non-smoker <4	4-4.9	5-5.9	6-6.9	≥ 7	Women Smoker <4	4-4.9	5-5.9	6-6.9	≥ 7
70-74	≥180	30	32	35	38	42	37	40	43	47	51	24	25	25	26	28	32	33	34	36	37
	160-179	25	27	29	32	35	31	34	37	40	43	20	21	22	23	24	27	28	30	31	32
	140-159	20	22	25	27	30	26	28	31	34	37	17	18	19	19	20	24	24	25	26	28
	120-139	17	19	20	23	25	21	23	26	28	31	15	15	16	16	17	20	21	22	23	24
	<120	14	15	17	19	21	18	19	22	24	26	12	13	13	14	15	17	18	19	19	20
65-69	≥180	25	27	30	33	36	33	36	39	43	47	20	21	22	23	24	29	30	31	33	34
	160-179	20	22	25	27	30	27	30	33	36	40	16	17	18	19	20	24	25	27	28	29
	140-159	16	18	20	22	25	22	24	27	30	33	14	14	15	16	17	20	21	22	24	25
	120-139	13	15	16	18	20	18	20	22	25	27	11	12	13	13	14	17	18	19	20	21
	<120	11	12	13	15	17	14	16	18	20	23	9	10	10	11	12	14	15	16	17	18
60-64	≥180	21	23	25	28	32	29	32	35	39	44	16	17	18	19	20	26	27	29	30	32
	160-179	16	18	20	23	26	23	26	29	32	36	13	14	15	16	17	21	22	24	25	27
	140-159	13	14	16	18	21	19	21	23	26	30	11	11	12	13	14	17	18	20	21	22
	120-139	10	11	13	15	17	15	17	19	21	24	9	9	10	11	11	14	15	16	17	19
	<120	8	9	10	12	13	12	13	15	17	19	7	8	8	9	9	12	12	13	14	15
55-59	≥180	17	19	22	24	27	26	29	32	36	40	13	14	15	16	18	23	24	26	28	30
	160-179	13	15	17	19	22	20	23	26	29	33	11	11	12	13	14	18	20	21	23	25
	140-159	10	12	13	15	17	16	18	20	23	26	8	9	10	11	11	15	16	17	19	20
	120-139	8	9	10	12	13	12	14	16	18	21	7	7	8	8	9	12	13	14	15	16
	<120	6	7	8	9	10	9	11	12	14	17	5	6	6	7	7	10	10	11	12	13
50-54	≥180	15	16	18	21	23	23	26	29	33	37	11	12	13	14	15	20	22	24	26	28
	160-179	11	12	14	16	18	18	20	23	26	30	9	9	10	11	12	16	17	19	21	23
	140-159	8	9	11	12	14	13	15	17	20	23	7	7	8	9	9	13	14	15	16	18
	120-139	6	7	8	9	11	10	12	13	16	18	5	6	6	7	7	10	11	12	13	14
	<120	5	5	6	7	8	8	9	10	12	14	4	4	5	5	6	8	9	9	10	12
45-49	≥180	12	14	15	17	20	21	23	26	30	34	9	10	11	12	13	18	20	22	24	26
	160-179	9	10	11	13	15	15	17	20	23	27	7	7	8	9	10	14	15	17	19	21
	140-159	6	7	8	10	12	11	13	15	18	21	5	6	6	7	8	11	12	13	15	16
	120-139	5	5	6	7	9	8	10	11	13	16	4	4	5	5	6	8	9	10	11	13
	<120	3	4	5	5	7	6	7	8	10	12	3	3	4	4	5	6	7	8	9	10
40-44	≥180	10	11	13	15	17	18	21	23	27	32	7	8	9	10	11	16	18	20	22	24
	160-179	7	8	9	11	13	13	15	17	20	24	5	6	7	7	8	12	13	15	17	19
	140-159	5	6	7	8	9	10	11	13	15	18	4	4	5	6	6	9	10	11	13	15
	120-139	4	4	5	6	7	7	8	9	11	14	3	3	4	4	5	7	8	9	10	11
	<120	3	3	4	4	5	5	6	7	8	10	2	3	3	3	4	5	6	7	8	9

Total cholesterol (mmol/l)

Southern Sub-Saharan Africa

Risk Level: <5% | 5% to <10% | 10% to <20% | 20% to <30% | ≥30%

People without Diabetes

Age (years)	SBP (mmHg)	Men Non-smoker <4	4-4.9	5-5.9	6-6.9	≥ 7	Men Smoker <4	4-4.9	5-5.9	6-6.9	≥ 7	Women Non-smoker <4	4-4.9	5-5.9	6-6.9	≥ 7	Women Smoker <4	4-4.9	5-5.9	6-6.9	≥ 7
70-74	≥180	21	23	26	28	31	27	29	32	35	39	18	19	19	20	21	25	26	27	28	29
	160-179	18	19	21	23	26	22	24	27	30	33	15	16	17	17	18	21	22	23	24	25
	140-159	14	16	17	19	21	18	20	22	25	27	13	13	14	15	15	18	19	19	20	21
	120-139	12	13	14	16	18	15	17	18	20	23	11	11	12	12	13	15	16	16	17	18
	<120	10	11	12	13	14	12	14	15	17	19	9	10	10	10	11	13	13	14	15	15
65-69	≥180	17	18	20	22	25	22	25	27	30	34	14	15	15	16	17	21	22	23	24	25
	160-179	13	15	16	18	20	18	20	22	25	28	12	12	13	13	14	17	18	19	20	21
	140-159	11	12	13	15	16	14	16	18	20	23	10	10	11	11	12	14	15	16	17	18
	120-139	8	9	10	12	13	12	13	14	16	18	8	8	9	9	10	12	13	13	14	15
	<120	7	7	8	9	11	9	10	12	13	15	6	7	7	8	8	10	10	11	12	12
60-64	≥180	13	14	16	18	20	18	21	23	26	29	11	11	12	13	14	17	18	19	21	22
	160-179	10	11	12	14	16	15	16	18	21	23	9	9	10	10	11	14	15	16	17	18
	140-159	8	9	10	11	13	11	13	14	16	19	7	7	8	8	9	11	12	13	14	15
	120-139	6	7	8	9	10	9	10	11	13	15	6	6	6	7	7	9	10	11	11	12
	<120	5	5	6	7	8	7	8	9	10	12	5	5	5	6	6	8	8	9	9	10
55-59	≥180	10	11	12	14	16	15	17	19	22	25	8	9	9	10	11	14	15	16	18	19
	160-179	8	8	10	11	13	12	13	15	17	20	6	7	7	8	9	11	12	13	14	15
	140-159	6	6	7	8	10	9	10	12	13	16	5	5	6	6	7	9	10	11	11	12
	120-139	4	5	6	6	7	7	8	9	10	12	4	4	5	5	5	7	8	8	9	10
	<120	3	4	4	5	6	5	6	7	8	9	3	3	4	4	4	6	6	7	7	8
50-54	≥180	8	9	10	11	13	12	14	16	19	22	6	7	7	8	9	12	13	14	15	17
	160-179	6	6	7	8	10	9	11	12	14	17	5	5	6	6	7	9	10	11	12	13
	140-159	4	5	5	6	7	7	8	9	11	13	4	4	4	5	5	7	8	9	9	10
	120-139	3	3	4	5	6	5	6	7	8	10	3	3	3	4	4	6	6	7	7	8
	<120	2	3	3	4	4	4	4	5	6	7	2	2	3	3	3	4	5	5	6	6
45-49	≥180	6	7	7	9	10	10	12	13	16	19	5	5	6	6	7	10	11	12	13	14
	160-179	4	5	5	6	8	7	9	10	12	14	4	4	4	5	5	8	8	9	10	11
	140-159	3	3	4	5	6	5	6	7	9	10	3	3	3	4	4	6	6	7	8	9
	120-139	2	3	3	3	4	4	5	5	6	8	2	2	2	3	3	4	5	5	6	7
	<120	2	2	2	3	3	3	3	4	5	6	2	2	2	2	2	3	4	4	5	5
40-44	≥180	5	5	6	7	8	8	10	11	13	16	4	4	4	5	5	8	9	10	11	12
	160-179	3	4	4	5	6	6	7	8	10	12	3	3	3	4	4	6	7	7	8	9
	140-159	2	3	3	3	4	4	5	6	7	9	2	2	2	3	3	5	5	6	6	7
	120-139	2	2	2	3	3	3	4	4	5	6	1	2	2	2	2	3	4	4	5	5
	<120	1	1	2	2	2	2	3	3	4	5	1	1	1	2	2	3	3	3	4	4

Total cholesterol (mmol/l)

Southern Sub-Saharan Africa

Risk Level: <5% | 5% to <10% | 10% to <20% | 20% to <30% | ≥30%

People with Diabetes

Age (years)	SBP (mmHg)	Men Non-smoker <4	4-4.9	5-5.9	6-6.9	≥ 7	Men Smoker <4	4-4.9	5-5.9	6-6.9	≥ 7	Women Non-smoker <4	4-4.9	5-5.9	6-6.9	≥ 7	Women Smoker <4	4-4.9	5-5.9	6-6.9	≥ 7
70-74	≥180	29	31	35	38	42	36	39	43	47	51	26	27	28	30	31	35	37	38	40	41
	160-179	24	26	29	32	35	30	33	36	40	44	23	23	24	25	27	31	32	33	34	36
	140-159	20	22	24	27	29	25	28	30	34	37	19	20	21	22	23	26	27	28	30	31
	120-139	16	18	20	22	25	21	23	25	28	31	16	17	18	18	19	22	23	24	25	27
	<120	13	15	16	18	20	17	19	21	23	26	14	14	15	16	16	19	20	21	22	23
65-69	≥180	24	26	29	32	36	32	35	39	43	47	22	23	24	25	27	32	33	35	37	39
	160-179	19	21	24	27	30	26	29	32	36	40	18	19	20	21	22	27	28	30	31	33
	140-159	16	17	19	22	24	21	24	26	30	33	15	16	17	18	19	23	24	25	26	28
	120-139	12	14	16	18	20	17	19	21	24	27	13	13	14	15	16	19	20	21	22	24
	<120	10	11	13	14	16	14	15	17	20	22	10	11	12	12	13	16	17	18	19	20
60-64	≥180	20	22	25	28	31	28	31	35	39	44	18	19	20	22	23	28	30	32	34	36
	160-179	16	17	20	22	25	22	25	28	32	36	15	16	17	18	19	24	25	27	28	30
	140-159	12	14	16	18	20	18	20	23	26	29	12	13	14	15	16	19	21	22	24	25
	120-139	10	11	12	14	16	14	16	18	21	24	10	10	11	12	13	16	17	18	19	21
	<120	7	8	10	11	13	11	13	14	17	19	8	8	9	10	10	13	14	15	16	17
55-59	≥180	16	18	21	23	27	25	28	31	36	40	15	16	17	18	20	25	27	29	31	34
	160-179	13	14	16	18	21	19	22	25	28	33	12	13	14	15	16	21	22	24	26	28
	140-159	10	11	12	14	17	15	17	20	23	26	9	10	11	12	13	17	18	19	21	23
	120-139	7	8	10	11	13	12	13	15	18	21	7	8	9	9	10	13	14	16	17	18
	<120	6	6	7	9	10	9	10	12	14	16	6	6	7	8	8	11	12	13	14	15
50-54	≥180	14	15	17	20	23	22	25	28	32	37	12	13	14	15	17	23	24	26	29	31
	160-179	10	11	13	15	18	17	19	22	25	29	10	10	11	12	13	18	19	21	23	25
	140-159	8	9	10	12	14	13	14	17	20	23	7	8	9	10	10	14	15	17	19	20
	120-139	6	6	7	9	10	10	11	13	15	18	6	6	7	7	8	11	12	13	15	16
	<120	4	5	6	7	8	7	8	10	12	14	4	5	5	6	7	9	10	11	12	13
45-49	≥180	11	13	14	17	20	19	22	25	29	34	10	11	12	13	14	20	22	24	26	29
	160-179	8	9	11	12	15	14	16	19	22	27	8	8	9	10	11	16	17	19	21	23
	140-159	6	7	8	9	11	11	12	14	17	20	6	6	7	8	9	12	13	15	16	18
	120-139	4	5	6	7	8	8	9	11	13	15	4	5	5	6	7	9	10	12	13	14
	<120	3	4	4	5	6	6	7	8	10	12	3	4	4	5	5	7	8	9	10	11
40-44	≥180	9	11	12	14	17	17	20	23	26	31	8	9	10	11	12	18	20	22	24	27
	160-179	7	8	9	10	12	12	14	17	20	24	6	7	7	8	9	14	15	17	19	21
	140-159	5	5	6	7	9	9	10	12	15	18	5	5	6	6	7	10	11	13	15	16
	120-139	3	4	5	5	7	6	8	9	11	13	3	4	4	5	5	8	9	10	11	13
	<120	2	3	3	4	5	5	5	7	8	10	2	3	3	4	4	6	7	8	9	10

Total cholesterol (mmol/l)

Western Sub-Saharan Africa

Risk Level: <5% | 5% to <10% | 10% to <20% | 20% to <30% | ≥30%

People without Diabetes

Age (years)	SBP (mmHg)	Men Non-smoker					Men Smoker					Women Non-smoker					Women Smoker				
		<4	4-4.9	5-5.9	6-6.9	≥ 7	<4	4-4.9	5-5.9	6-6.9	≥ 7	<4	4-4.9	5-5.9	6-6.9	≥ 7	<4	4-4.9	5-5.9	6-6.9	≥ 7
70-74	≥180	22	24	26	29	32	28	30	33	36	40	18	19	20	21	22	25	27	28	29	30
70-74	160-179	18	20	22	24	27	23	25	28	31	34	15	16	17	17	18	22	22	23	25	26
70-74	140-159	15	16	18	20	22	19	21	23	25	28	13	13	14	15	15	18	19	20	21	22
70-74	120-139	12	13	15	16	18	15	17	19	21	23	11	11	12	12	13	15	16	17	17	18
70-74	<120	10	11	12	13	15	13	14	15	17	19	9	9	10	10	11	13	13	14	15	15
65-69	≥180	17	19	21	23	26	23	25	28	31	35	14	15	15	16	17	21	22	23	25	26
65-69	160-179	14	15	17	19	21	18	21	23	26	29	11	12	13	13	14	17	18	19	21	22
65-69	140-159	11	12	13	15	17	15	17	18	21	23	9	10	10	11	12	14	15	16	17	18
65-69	120-139	9	10	11	12	14	12	13	15	17	19	8	8	8	9	10	12	12	13	14	15
65-69	<120	7	8	9	10	11	9	11	12	13	15	6	6	7	7	8	10	10	11	12	12
60-64	≥180	13	15	16	18	21	19	21	24	27	30	10	11	12	13	14	17	18	20	21	23
60-64	160-179	10	11	13	14	16	15	17	19	21	24	8	9	9	10	11	14	15	16	17	19
60-64	140-159	8	9	10	11	13	12	13	15	17	19	7	7	8	8	9	11	12	13	14	15
60-64	120-139	6	7	8	9	10	9	10	12	13	15	5	6	6	7	7	9	10	10	11	12
60-64	<120	5	5	6	7	8	7	8	9	10	12	4	5	5	5	6	7	8	8	9	10
55-59	≥180	10	11	13	14	17	16	17	20	23	26	8	8	9	10	11	14	15	17	18	20
55-59	160-179	8	9	10	11	13	12	13	15	18	20	6	7	7	8	8	11	12	13	14	16
55-59	140-159	6	7	7	9	10	9	10	12	14	16	5	5	6	6	7	9	10	10	11	12
55-59	120-139	4	5	6	7	8	7	8	9	11	12	4	4	4	5	5	7	8	8	9	10
55-59	<120	3	4	4	5	6	5	6	7	8	10	3	3	3	4	4	5	6	6	7	8
50-54	≥180	8	9	10	11	13	13	14	17	19	22	6	6	7	8	8	12	13	14	15	17
50-54	160-179	6	6	7	8	10	10	11	13	15	17	5	5	5	6	6	9	10	11	12	13
50-54	140-159	4	5	5	6	7	7	8	9	11	13	3	4	4	4	5	7	8	8	9	10
50-54	120-139	3	4	4	5	6	5	6	7	8	10	3	3	3	3	4	5	6	6	7	8
50-54	<120	2	3	3	4	4	4	5	5	6	8	2	2	2	3	3	4	4	5	5	6
45-49	≥180	6	7	8	9	10	10	12	14	16	19	5	5	5	6	7	10	11	12	13	14
45-49	160-179	4	5	6	6	8	8	9	10	12	14	3	4	4	4	5	7	8	9	10	11
45-49	140-159	3	3	4	5	6	6	6	7	9	11	2	3	3	3	4	5	6	7	7	8
45-49	120-139	2	3	3	3	4	4	5	6	7	8	2	2	2	2	3	4	4	5	6	6
45-49	<120	2	2	2	3	3	3	3	4	5	6	1	1	2	2	2	3	3	4	4	5
40-44	≥180	5	5	6	7	8	9	10	11	14	16	3	4	4	5	5	8	9	10	11	12
40-44	160-179	3	4	4	5	6	6	7	8	10	12	2	3	3	3	4	6	6	7	8	9
40-44	140-159	2	3	3	4	4	4	5	6	7	9	2	2	2	2	3	4	5	5	6	7
40-44	120-139	2	2	2	3	3	3	4	4	5	6	1	1	2	2	2	3	3	4	4	5
40-44	<120	1	1	2	2	2	2	3	3	4	5	1	1	1	1	1	2	3	3	3	4

Total cholesterol (mmol/l)

Western Sub-Saharan Africa

Risk Level: <5% | 5% to <10% | 10% to <20% | 20% to <30% | ≥30%

People with Diabetes

Age (years)	SBP (mmHg)	Men Non-smoker					Men Smoker					Women Non-smoker					Women Smoker				
		<4	4-4.9	5-5.9	6-6.9	≥ 7	<4	4-4.9	5-5.9	6-6.9	≥ 7	<4	4-4.9	5-5.9	6-6.9	≥ 7	<4	4-4.9	5-5.9	6-6.9	≥ 7
70-74	≥180	30	32	36	39	43	37	41	44	48	53	27	28	30	31	33	37	39	41	42	44
70-74	160-179	25	27	30	33	36	31	34	38	41	45	23	24	25	26	28	32	33	35	37	38
70-74	140-159	20	22	25	27	31	26	28	31	35	39	19	20	21	22	24	27	29	30	31	33
70-74	120-139	17	18	20	23	25	21	24	26	29	32	16	17	18	19	20	23	24	25	27	28
70-74	<120	14	15	17	19	21	17	19	22	24	27	14	14	15	16	17	19	20	21	23	24
65-69	≥180	25	27	30	34	37	33	36	40	44	49	22	24	25	26	28	33	35	37	39	41
65-69	160-179	20	22	25	28	31	27	30	33	37	41	18	19	21	22	23	28	29	31	33	35
65-69	140-159	16	18	20	22	25	22	24	27	31	34	15	16	17	18	19	23	25	26	28	30
65-69	120-139	13	14	16	18	21	18	20	22	25	28	12	13	14	15	16	19	20	22	23	25
65-69	<120	10	11	13	15	17	14	16	18	20	23	10	11	12	12	13	16	17	18	19	21
60-64	≥180	20	23	25	29	32	29	32	36	40	45	18	19	21	22	24	30	32	34	36	39
60-64	160-179	16	18	20	23	26	23	26	29	33	37	15	16	17	18	19	24	26	28	30	32
60-64	140-159	13	14	16	18	21	18	21	23	27	31	12	13	14	15	16	20	21	23	25	27
60-64	120-139	10	11	13	14	17	14	16	19	21	25	9	10	11	12	13	16	17	19	20	22
60-64	<120	8	9	10	11	13	11	13	15	17	20	8	8	9	9	10	13	14	15	16	18
55-59	≥180	17	19	21	24	28	26	29	32	37	42	15	16	17	19	20	26	28	31	33	36
55-59	160-179	13	15	16	19	22	20	23	26	29	34	12	13	14	15	16	21	23	25	27	29
55-59	140-159	10	11	13	15	17	15	18	20	23	27	9	10	11	12	13	17	18	20	22	24
55-59	120-139	7	9	10	11	13	12	14	16	18	21	7	8	8	9	10	13	14	16	17	19
55-59	<120	6	6	7	9	10	9	10	12	14	17	6	6	7	7	8	10	11	13	14	15
50-54	≥180	14	16	18	20	24	23	25	29	33	39	12	13	14	16	17	23	25	28	30	33
50-54	160-179	10	12	13	16	18	17	20	23	26	31	9	10	11	12	13	18	20	22	24	27
50-54	140-159	8	9	10	12	14	13	15	17	20	24	7	8	8	9	10	14	16	17	19	21
50-54	120-139	6	7	8	9	11	10	11	13	16	18	5	6	7	7	8	11	12	13	15	17
50-54	<120	4	5	6	7	8	7	8	10	12	14	4	5	5	6	6	8	9	10	12	13
45-49	≥180	12	13	15	17	20	20	23	26	30	35	10	11	12	13	14	20	23	25	28	31
45-49	160-179	8	10	11	13	15	15	17	20	23	27	7	8	9	10	11	16	17	19	22	24
45-49	140-159	6	7	8	9	11	11	13	15	18	21	5	6	7	7	8	12	13	15	17	19
45-49	120-139	4	5	6	7	8	8	9	11	13	16	4	4	5	6	6	9	10	11	13	15
45-49	<120	3	4	4	5	6	6	7	8	10	12	3	3	4	4	5	7	8	9	10	11
40-44	≥180	10	11	12	14	17	18	20	23	27	32	8	9	10	11	12	18	20	22	25	28
40-44	160-179	7	8	9	10	13	13	15	17	20	25	6	6	7	8	9	13	15	17	19	22
40-44	140-159	5	5	6	8	9	9	11	13	15	18	4	5	5	6	7	10	11	13	15	17
40-44	120-139	3	4	5	5	7	7	8	9	11	14	3	3	4	4	5	7	8	10	11	13
40-44	<120	2	3	3	4	5	5	6	7	8	10	2	2	3	3	4	6	6	7	8	10

Total cholesterol (mmol/l)

Southern Latin America

Risk Level: <5% | 5% to <10% | 10% to <20% | 20% to <30% | ≥30%

People without Diabetes

Total cholesterol (mmol/l)

Age (years)	SBP (mmHg)	Men Non-smoker					Men Smoker					Women Non-smoker					Women Smoker				
		<4	4-4.9	5-5.9	6-6.9	≥ 7	<4	4-4.9	5-5.9	6-6.9	≥ 7	<4	4-4.9	5-5.9	6-6.9	≥ 7	<4	4-4.9	5-5.9	6-6.9	≥ 7
70-74	≥180	24	26	29	31	34	30	32	35	38	42	16	17	19	20	21	25	26	28	30	33
	160-179	20	22	24	26	29	25	27	30	33	36	13	14	15	16	17	20	22	23	25	27
	140-159	17	18	20	22	24	21	23	25	28	30	11	11	12	13	14	16	18	19	20	22
	120-139	14	15	17	19	21	18	19	21	23	26	9	9	10	10	11	13	14	15	16	18
	<120	12	13	14	16	17	15	16	18	20	22	7	7	8	8	9	11	11	12	13	14
65-69	≥180	19	21	23	25	28	25	27	30	33	37	12	12	13	15	16	20	21	23	25	27
	160-179	15	17	19	21	23	21	23	25	28	31	9	10	11	11	13	16	17	18	20	22
	140-159	13	14	16	17	19	17	19	21	23	26	7	8	8	9	10	12	13	15	16	17
	120-139	10	11	13	14	16	14	15	17	19	22	6	6	7	7	8	10	11	11	13	14
	<120	8	9	10	12	13	11	13	14	16	18	4	5	5	6	6	8	8	9	10	11
60-64	≥180	15	16	18	21	23	21	23	26	29	32	8	9	10	11	12	15	17	19	21	23
	160-179	12	13	15	17	19	17	19	21	24	27	6	7	7	8	9	12	13	14	16	18
	140-159	9	11	12	13	15	14	15	17	19	22	5	5	6	6	7	9	10	11	12	14
	120-139	8	8	10	11	12	11	12	14	16	18	4	4	4	5	5	7	8	9	10	11
	<120	6	7	8	9	10	9	10	11	13	15	3	3	3	4	4	5	6	7	7	8
55-59	≥180	12	13	15	17	19	17	20	22	25	28	6	6	7	8	9	12	13	15	17	19
	160-179	9	10	12	13	15	14	16	18	20	23	4	5	5	6	7	9	10	11	13	14
	140-159	7	8	9	10	12	11	12	14	16	19	3	4	4	4	5	7	8	9	10	11
	120-139	5	6	7	8	10	8	10	11	13	15	2	3	3	3	4	5	6	6	7	8
	<120	4	5	6	6	8	7	8	9	10	12	2	2	2	2	3	4	4	5	5	6
50-54	≥180	9	10	12	13	15	14	16	19	22	25	4	5	5	6	6	9	11	12	14	16
	160-179	7	8	9	10	12	11	13	15	17	20	3	3	4	4	5	7	8	9	10	12
	140-159	5	6	7	8	9	9	10	11	13	16	2	2	3	3	3	5	6	7	7	9
	120-139	4	5	5	6	7	7	8	9	10	12	2	2	2	2	3	4	4	5	6	6
	<120	3	4	4	5	6	5	6	7	8	10	1	1	1	2	2	3	3	4	4	5
45-49	≥180	7	8	9	11	12	12	14	16	18	22	3	3	4	4	5	7	8	10	11	13
	160-179	5	6	7	8	10	9	10	12	14	17	2	2	3	3	3	5	6	7	8	9
	140-159	4	4	5	6	7	7	8	9	11	13	2	2	2	2	2	4	4	5	6	7
	120-139	3	3	4	5	6	5	6	7	8	10	1	1	1	2	2	3	3	4	4	5
	<120	2	3	3	4	4	4	5	5	6	8	1	1	1	1	1	2	2	3	3	4
40-44	≥180	5	6	7	8	10	10	11	13	16	19	2	3	3	3	4	6	7	8	9	11
	160-179	4	5	5	6	8	7	9	10	12	14	2	2	2	2	2	4	5	5	6	8
	140-159	3	3	4	5	6	5	6	8	9	11	1	1	1	2	2	3	3	4	4	5
	120-139	2	2	3	4	4	4	5	6	7	8	1	1	1	1	1	2	2	3	3	4
	<120	2	2	2	3	3	3	4	4	5	6	0	1	1	1	1	1	2	2	2	3

Southern Latin America

Risk Level: <5% | 5% to <10% | 10% to <20% | 20% to <30% | ≥30%

People with Diabetes

Total cholesterol (mmol/l)

Age (years)	SBP (mmHg)	Men Non-smoker					Men Smoker					Women Non-smoker					Women Smoker				
		<4	4-4.9	5-5.9	6-6.9	≥ 7	<4	4-4.9	5-5.9	6-6.9	≥ 7	<4	4-4.9	5-5.9	6-6.9	≥ 7	<4	4-4.9	5-5.9	6-6.9	≥ 7
70-74	≥180	32	34	38	41	45	39	42	46	49	54	27	29	31	33	36	40	43	46	49	52
	160-179	27	29	32	35	38	33	36	39	43	47	22	24	25	27	29	33	36	38	41	44
	140-159	23	25	27	30	33	28	31	34	37	40	18	19	21	22	24	28	29	32	34	37
	120-139	19	21	23	25	28	24	26	29	32	35	15	16	17	18	19	22	24	26	28	30
	<120	16	17	19	21	24	20	22	24	27	30	12	13	13	15	16	18	20	21	23	25
65-69	≥180	27	29	32	36	39	35	38	42	46	50	21	23	25	27	29	35	38	41	44	48
	160-179	22	24	27	30	33	29	32	35	39	43	17	18	20	21	23	28	31	33	36	40
	140-159	18	20	22	25	28	24	27	30	33	37	13	14	16	17	19	23	25	27	29	32
	120-139	15	17	18	21	23	20	22	25	28	31	10	11	12	13	15	18	20	22	24	26
	<120	12	14	15	17	19	16	18	20	23	26	8	9	10	11	12	14	16	17	19	21
60-64	≥180	22	25	28	31	34	31	34	38	42	47	16	18	19	22	24	30	33	36	40	44
	160-179	18	20	23	25	29	25	28	32	35	39	13	14	15	17	19	24	26	29	32	36
	140-159	14	16	18	21	23	21	23	26	29	33	10	11	12	13	15	19	21	23	25	28
	120-139	12	13	15	17	19	17	19	21	24	27	7	8	9	10	11	14	16	18	20	22
	<120	9	11	12	14	16	13	15	17	20	22	6	6	7	8	9	11	12	14	16	17
55-59	≥180	19	21	23	27	30	27	31	34	39	44	13	14	15	17	19	26	29	32	36	40
	160-179	15	17	19	21	24	22	25	28	32	36	9	10	12	13	15	20	22	25	28	32
	140-159	12	13	15	17	20	18	20	23	26	30	7	8	9	10	11	15	17	19	22	25
	120-139	9	10	12	14	16	14	16	18	21	24	5	6	7	7	8	12	13	15	17	19
	<120	7	8	9	11	13	11	13	14	17	19	4	4	5	6	6	9	10	11	13	15
50-54	≥180	16	18	20	23	26	24	28	31	36	40	10	11	12	14	16	22	25	28	32	37
	160-179	12	14	16	18	21	19	22	25	29	33	7	8	9	10	12	17	19	22	25	29
	140-159	9	11	12	14	16	15	17	20	23	27	5	6	7	8	9	12	14	16	19	22
	120-139	7	8	9	11	13	12	13	15	18	21	4	4	5	6	6	9	10	12	14	16
	<120	5	6	7	9	10	9	10	12	14	17	3	3	4	4	5	7	8	9	10	12
45-49	≥180	13	15	17	19	23	22	25	28	33	38	8	8	10	11	13	19	22	25	29	34
	160-179	10	11	13	15	18	17	19	22	26	30	5	6	7	8	9	14	16	18	22	25
	140-159	7	8	10	12	14	13	15	17	20	24	4	4	5	6	7	10	12	14	16	19
	120-139	5	6	7	9	11	10	11	13	16	19	3	3	4	4	5	7	8	10	12	14
	<120	4	5	6	7	8	7	9	10	12	15	2	2	3	3	3	5	6	7	9	10
40-44	≥180	11	12	14	17	20	19	22	25	30	35	6	7	8	9	10	16	19	22	26	31
	160-179	8	9	11	13	15	14	17	19	23	27	4	5	5	6	7	11	13	16	19	23
	140-159	6	7	8	9	11	11	13	15	18	21	3	3	4	4	5	8	10	11	14	17
	120-139	4	5	6	7	9	8	9	11	13	16	2	2	3	3	4	6	7	8	10	12
	<120	3	4	4	5	6	6	7	8	10	13	1	2	2	2	3	4	5	6	7	9

High-income North America

Risk Level: <5% | 5% to <10% | 10% to <20% | 20% to <30% | ≥30%

People without Diabetes

Age (years)	SBP (mmHg)	Men										Women									
		Non-smoker					Smoker					Non-smoker					Smoker				
		<4	4-4.9	5-5.9	6-6.9	≥ 7	<4	4-4.9	5-5.9	6-6.9	≥ 7	<4	4-4.9	5-5.9	6-6.9	≥ 7	<4	4-4.9	5-5.9	6-6.9	≥ 7
70-74	≥180	29	31	34	36	39	35	38	41	44	47	22	23	24	26	27	31	33	34	36	38
	160-179	24	26	29	31	34	30	32	35	38	41	18	19	20	21	23	26	28	29	31	32
	140-159	20	22	24	26	29	25	27	30	32	35	15	16	17	18	19	22	23	24	26	27
	120-139	17	19	21	22	25	21	23	25	28	30	13	13	14	15	16	18	19	20	22	23
	<120	14	16	17	19	21	18	20	21	24	26	10	11	12	12	13	15	16	17	18	19
65-69	≥180	23	25	27	30	33	30	32	35	38	42	16	17	19	20	21	26	27	29	31	33
	160-179	19	21	23	25	28	25	27	30	33	36	13	14	15	16	17	21	22	24	25	27
	140-159	16	17	19	21	23	21	23	25	27	30	11	11	12	13	14	17	18	20	21	23
	120-139	13	14	16	18	19	17	19	21	23	26	9	9	10	11	11	14	15	16	17	18
	<120	11	12	13	15	16	14	16	17	19	21	7	7	8	9	9	11	12	13	14	15
60-64	≥180	18	20	22	25	27	25	28	31	34	37	12	13	14	15	16	21	23	24	26	28
	160-179	15	16	18	20	23	21	23	25	28	31	10	10	11	12	13	17	18	20	21	23
	140-159	12	13	15	17	19	17	19	21	23	26	8	8	9	10	10	13	14	16	17	19
	120-139	10	11	12	14	15	14	15	17	19	21	6	6	7	8	8	11	12	13	14	15
	<120	8	9	10	11	13	11	12	14	16	18	5	5	6	6	7	8	9	10	11	12
55-59	≥180	15	16	18	20	23	21	24	26	29	33	9	10	11	12	13	17	19	20	22	24
	160-179	11	13	14	16	18	17	19	21	24	27	7	7	8	9	10	13	15	16	18	19
	140-159	9	10	12	13	15	14	15	17	20	22	5	6	6	7	8	10	11	12	14	15
	120-139	7	8	9	10	12	11	12	14	16	18	4	4	5	5	6	8	9	10	11	12
	<120	6	6	7	8	10	9	10	11	13	15	3	3	4	4	5	6	7	8	8	9
50-54	≥180	11	13	15	16	19	18	20	23	26	29	7	7	8	9	10	14	15	17	19	21
	160-179	9	10	11	13	15	14	16	18	21	24	5	5	6	7	7	10	12	13	14	16
	140-159	7	8	9	10	12	11	12	14	16	19	4	4	5	5	6	8	9	10	11	13
	120-139	5	6	7	8	9	9	10	11	13	15	3	3	3	4	4	6	7	8	9	10
	<120	4	5	5	6	7	7	8	9	10	12	2	2	3	3	3	5	5	6	7	7
45-49	≥180	9	10	12	13	15	15	17	19	22	26	5	5	6	7	7	11	12	14	16	18
	160-179	7	8	9	10	12	11	13	15	18	20	4	4	4	5	6	8	9	10	12	13
	140-159	5	6	7	8	9	9	10	12	14	16	3	3	3	4	4	6	7	8	9	10
	120-139	4	5	5	6	7	7	8	9	11	13	2	2	2	3	3	5	5	6	7	8
	<120	3	3	4	5	6	5	6	7	8	10	1	2	2	2	2	3	4	4	5	6
40-44	≥180	7	8	9	11	13	13	14	17	19	23	4	4	4	5	6	9	10	11	13	15
	160-179	5	6	7	8	10	9	11	13	15	18	3	3	3	4	4	6	7	8	10	11
	140-159	4	4	5	6	7	7	8	10	11	14	2	2	2	3	3	5	5	6	7	8
	120-139	3	3	4	5	6	5	6	7	9	11	1	1	2	2	2	3	4	5	5	6
	<120	2	3	3	4	4	4	5	6	7	8	1	1	1	1	2	2	3	3	4	5

Total cholesterol (mmol/l)

High-income North America

Risk Level: <5% | 5% to <10% | 10% to <20% | 20% to <30% | ≥30%

People with Diabetes

Age (years)	SBP (mmHg)	Men										Women									
		Non-smoker					Smoker					Non-smoker					Smoker				
		<4	4-4.9	5-5.9	6-6.9	≥ 7	<4	4-4.9	5-5.9	6-6.9	≥ 7	<4	4-4.9	5-5.9	6-6.9	≥ 7	<4	4-4.9	5-5.9	6-6.9	≥ 7
70-74	≥180	37	40	43	46	50	44	48	51	54	58	34	35	37	39	41	46	48	50	52	55
	160-179	32	34	37	40	43	38	41	44	48	51	28	30	31	33	35	40	41	43	45	48
	140-159	27	29	32	35	37	33	36	39	42	45	24	25	26	28	29	34	35	37	39	41
	120-139	23	25	27	30	32	28	31	33	36	39	20	21	22	23	25	28	30	31	33	35
	<120	19	21	23	25	28	24	26	28	31	34	17	17	18	19	21	24	25	27	28	30
65-69	≥180	32	34	37	41	44	40	43	47	51	55	27	29	31	33	35	41	43	46	48	51
	160-179	26	29	32	35	38	34	37	40	44	48	22	24	25	27	29	34	37	39	41	44
	140-159	22	24	27	29	32	29	31	34	38	41	18	20	21	22	24	29	30	32	35	37
	120-139	18	20	22	25	27	24	26	29	32	35	15	16	17	18	20	24	25	27	29	31
	<120	15	17	19	21	23	20	22	24	27	30	12	13	14	15	16	19	21	22	24	26
60-64	≥180	27	29	32	36	39	36	40	43	47	52	22	24	25	27	29	36	39	42	45	48
	160-179	22	24	27	30	33	30	33	36	40	44	18	19	20	22	24	30	32	35	37	40
	140-159	18	20	22	25	28	25	27	30	34	37	14	15	16	18	19	24	26	28	31	33
	120-139	14	16	18	20	23	20	23	25	28	31	11	12	13	14	16	20	21	23	25	27
	<120	12	13	15	17	19	17	19	21	23	26	9	10	10	11	12	16	17	19	20	22
55-59	≥180	23	25	28	31	35	33	36	40	44	48	18	19	21	23	25	32	35	38	41	45
	160-179	18	20	23	25	29	26	29	33	36	41	14	15	16	18	20	26	28	31	33	37
	140-159	14	16	18	21	23	21	24	27	30	34	11	12	13	14	16	20	22	25	27	30
	120-139	11	13	15	17	19	17	19	22	25	28	8	9	10	11	12	16	18	20	22	24
	<120	9	10	12	14	15	14	16	18	20	23	6	7	8	9	10	13	14	16	17	19
50-54	≥180	19	21	24	27	31	29	33	36	41	45	14	15	17	19	21	28	31	34	38	41
	160-179	15	17	19	22	25	23	26	29	33	37	11	12	13	15	16	22	24	27	30	33
	140-159	12	13	15	17	20	18	21	24	27	31	8	9	10	11	13	17	19	21	24	27
	120-139	9	10	12	14	16	14	16	19	22	25	6	7	8	9	10	13	15	17	19	21
	<120	7	8	9	11	13	11	13	15	17	20	5	5	6	7	7	10	11	13	15	17
45-49	≥180	16	18	21	23	27	26	29	33	37	42	11	12	14	16	17	25	28	31	34	38
	160-179	12	14	16	18	21	20	23	26	30	34	8	9	10	12	13	19	21	24	27	30
	140-159	9	11	12	14	17	16	18	21	24	28	6	7	8	9	10	14	16	18	21	24
	120-139	7	8	10	11	13	12	14	16	19	22	5	5	6	7	8	11	12	14	16	18
	<120	5	6	7	9	10	9	11	13	15	18	3	4	4	5	6	8	9	11	12	14
40-44	≥180	14	15	18	20	23	24	27	30	34	39	9	10	11	13	15	22	25	28	31	35
	160-179	10	12	13	16	18	18	20	23	27	32	6	7	8	9	11	16	18	21	24	28
	140-159	8	9	10	12	14	14	16	18	21	25	5	5	6	7	8	12	14	16	18	21
	120-139	6	7	8	9	11	10	12	14	17	20	3	4	4	5	6	9	10	12	14	16
	<120	4	5	6	7	8	8	9	11	13	15	2	3	3	4	4	7	8	9	10	12

Total cholesterol (mmol/l)

Caribbean

Risk Level: <5% | 5% to <10% | 10% to <20% | 20% to <30% | ≥30%

People without Diabetes

Age (years)	SBP (mmHg)	Men Non-smoker					Men Smoker					Women Non-smoker					Women Smoker				
	Total cholesterol (mmol/l)	<4	4-4.9	5-5.9	6-6.9	≥ 7	<4	4-4.9	5-5.9	6-6.9	≥ 7	<4	4-4.9	5-5.9	6-6.9	≥ 7	<4	4-4.9	5-5.9	6-6.9	≥ 7
70-74	≥180	22	24	26	29	32	27	30	33	36	40	17	18	19	20	21	24	25	27	28	29
	160-179	18	20	22	24	26	23	25	27	30	33	15	15	16	17	18	20	21	23	24	25
	140-159	15	16	18	20	22	19	20	23	25	28	12	13	14	14	15	17	18	19	20	21
	120-139	12	13	14	16	18	15	17	19	21	23	10	11	11	12	13	15	15	16	17	18
	<120	10	11	12	13	15	12	14	15	17	19	9	9	10	10	11	12	13	14	14	15
65-69	≥180	17	19	21	23	26	23	25	28	31	34	13	14	15	16	17	20	21	23	24	26
	160-179	13	15	17	19	21	18	20	23	25	28	11	12	12	13	14	17	18	19	20	21
	140-159	11	12	13	15	17	15	16	18	21	23	9	10	10	11	11	14	15	16	17	18
	120-139	9	9	11	12	13	12	13	15	17	19	7	8	8	9	9	11	12	13	14	15
	<120	7	8	8	10	11	9	10	12	13	15	6	6	7	7	8	9	10	11	11	12
60-64	≥180	13	14	16	18	21	19	21	23	26	30	10	11	11	12	13	17	18	19	20	22
	160-179	10	11	13	14	16	15	16	19	21	24	8	9	9	10	11	13	14	16	17	18
	140-159	8	9	10	11	13	12	13	15	17	19	7	7	7	8	9	11	12	13	14	15
	120-139	6	7	8	9	10	9	10	12	13	15	5	6	6	7	7	9	10	10	11	12
	<120	5	5	6	7	8	7	8	9	10	12	4	5	5	5	6	7	8	8	9	10
55-59	≥180	10	11	13	14	16	15	17	20	22	26	8	8	9	10	10	14	15	16	17	19
	160-179	8	9	10	11	13	12	13	15	17	20	6	6	7	8	8	11	12	13	14	15
	140-159	6	6	7	8	10	9	10	12	14	16	5	5	6	6	7	9	9	10	11	12
	120-139	4	5	6	6	7	7	8	9	10	12	4	4	4	5	5	7	7	8	9	10
	<120	3	4	4	5	6	5	6	7	8	9	3	3	3	4	4	5	6	6	7	8
50-54	≥180	8	9	10	11	13	13	14	16	19	22	6	6	7	7	8	11	12	13	15	16
	160-179	6	6	7	8	10	9	11	12	14	17	4	5	5	6	6	9	10	11	12	13
	140-159	4	5	5	6	7	7	8	9	11	13	3	4	4	5	5	7	7	8	9	10
	120-139	3	4	4	5	6	5	6	7	8	10	3	3	3	3	4	5	6	6	7	8
	<120	2	3	3	4	4	4	4	5	6	7	2	2	2	3	3	4	5	5	6	6
45-49	≥180	6	7	8	9	10	10	12	14	16	19	4	5	5	6	6	9	10	11	13	14
	160-179	4	5	6	6	8	8	9	10	12	14	3	4	4	4	5	7	8	9	10	11
	140-159	3	3	4	5	6	6	6	7	9	11	3	3	3	3	4	5	6	7	8	8
	120-139	2	3	3	3	4	4	5	5	7	8	2	2	2	3	3	4	5	5	6	7
	<120	2	2	2	3	3	3	3	4	5	6	1	2	2	2	2	3	3	4	4	5
40-44	≥180	5	5	6	7	8	9	10	11	13	16	3	4	4	5	5	8	8	9	11	12
	160-179	3	4	4	5	6	6	7	8	10	12	3	3	3	3	4	6	6	7	8	9
	140-159	2	3	3	4	4	4	5	6	7	9	2	2	2	3	3	4	5	5	6	7
	120-139	2	2	2	3	3	3	4	4	5	6	1	1	2	2	2	3	4	4	5	5
	<120	1	1	2	2	2	2	3	3	4	5	1	1	1	1	2	2	3	3	3	4

Total cholesterol (mmol/l)

Caribbean

Risk Level: <5% | 5% to <10% | 10% to <20% | 20% to <30% | ≥30%

People with Diabetes

Age (years)	SBP (mmHg)	Men Non-smoker					Men Smoker					Women Non-smoker					Women Smoker				
	Total cholesterol (mmol/l)	<4	4-4.9	5-5.9	6-6.9	≥ 7	<4	4-4.9	5-5.9	6-6.9	≥ 7	<4	4-4.9	5-5.9	6-6.9	≥ 7	<4	4-4.9	5-5.9	6-6.9	≥ 7
70-74	≥180	29	32	35	39	43	37	40	44	48	52	26	27	28	30	32	35	37	39	41	43
	160-179	24	27	29	33	36	31	34	37	41	45	22	23	24	26	27	30	32	34	35	37
	140-159	20	22	24	27	30	25	28	31	34	38	19	20	21	22	23	26	27	29	30	32
	120-139	16	18	20	22	25	21	23	26	29	32	16	17	17	18	19	22	23	25	26	27
	<120	13	15	17	19	21	17	19	21	24	27	13	14	15	16	16	19	20	21	22	23
65-69	≥180	24	27	30	33	37	32	36	40	44	49	21	22	24	25	27	32	33	35	38	40
	160-179	20	22	24	27	31	26	29	33	37	41	18	19	20	21	23	27	28	30	32	34
	140-159	16	18	20	22	25	22	24	27	30	34	15	16	17	18	19	22	24	25	27	29
	120-139	13	14	16	18	20	17	19	22	25	28	12	13	14	15	16	19	20	21	23	24
	<120	10	11	13	14	16	14	16	18	20	23	10	11	11	12	13	15	16	18	19	20
60-64	≥180	20	22	25	28	32	29	32	36	40	45	17	18	20	21	23	28	30	32	35	37
	160-179	16	18	20	23	26	23	26	29	33	37	14	15	16	17	19	23	25	27	29	31
	140-159	12	14	16	18	21	18	20	23	26	30	11	12	13	14	15	19	21	22	24	26
	120-139	10	11	12	14	16	14	16	18	21	24	9	10	11	12	13	16	17	18	20	22
	<120	8	9	10	11	13	11	13	15	17	19	7	8	9	9	10	13	14	15	16	18
55-59	≥180	17	19	21	24	27	25	28	32	36	41	14	15	16	18	20	25	27	29	32	35
	160-179	13	14	16	19	22	20	22	25	29	33	11	12	13	14	16	20	22	24	26	29
	140-159	10	11	13	15	17	15	17	20	23	27	9	10	11	12	13	16	18	19	21	23
	120-139	7	8	10	11	13	12	13	15	18	21	7	8	8	9	10	13	14	16	17	19
	<120	6	6	7	9	10	9	10	12	14	16	6	6	7	7	8	10	11	13	14	15
50-54	≥180	14	15	18	20	23	22	25	29	33	38	11	12	14	15	17	22	24	26	29	32
	160-179	10	12	13	15	18	17	19	22	26	30	9	10	11	12	13	17	19	21	24	26
	140-159	8	9	10	12	14	13	15	17	20	24	7	8	8	9	10	14	15	17	19	21
	120-139	6	7	8	9	10	10	11	13	15	18	5	6	7	7	8	11	12	13	15	17
	<120	4	5	6	7	8	7	8	10	12	14	4	5	5	6	6	8	9	11	12	13
45-49	≥180	11	13	15	17	20	20	22	26	30	35	9	10	11	13	14	19	21	24	27	30
	160-179	8	9	11	13	15	15	17	19	23	27	7	8	9	10	11	15	17	19	21	24
	140-159	6	7	8	9	11	11	12	15	17	21	5	6	7	7	8	12	13	15	17	19
	120-139	4	5	6	7	8	8	9	11	13	16	4	5	5	6	6	9	10	11	13	15
	<120	3	4	4	5	6	6	7	8	10	12	3	3	4	4	5	7	8	9	10	12
40-44	≥180	10	11	12	14	17	17	20	23	27	32	8	8	9	10	12	17	19	22	24	28
	160-179	7	8	9	10	12	13	14	17	20	24	6	6	7	8	9	13	15	17	19	22
	140-159	5	5	6	8	9	9	11	12	15	18	4	5	5	6	7	10	11	13	15	17
	120-139	3	4	5	5	7	6	8	9	11	14	3	3	4	4	5	7	8	10	11	13
	<120	2	3	3	4	5	5	5	7	8	10	2	3	3	3	4	6	6	7	9	10

Total cholesterol (mmol/l)

Andean Latin America

Risk Level: <5% | 5% to <10% | 10% to <20% | 20% to <30% | ≥30%

People without Diabetes

Age (years)	SBP (mmHg)	Men Non-smoker					Men Smoker					Women Non-smoker					Women Smoker				
	Total cholesterol (mmol/l)	<4	4-4.9	5-5.9	6-6.9	≥ 7	<4	4-4.9	5-5.9	6-6.9	≥ 7	<4	4-4.9	5-5.9	6-6.9	≥ 7	<4	4-4.9	5-5.9	6-6.9	≥ 7
70-74	≥180	16	18	20	22	24	21	23	25	27	30	12	13	14	14	15	17	18	19	20	21
	160-179	14	15	16	18	20	17	19	21	23	25	11	11	12	12	13	15	15	16	17	18
	140-159	11	12	14	15	17	14	16	17	19	21	9	9	10	10	11	12	13	14	14	15
	120-139	9	10	11	12	14	12	13	14	16	18	8	8	8	9	9	11	11	12	12	13
	<120	8	8	9	10	11	10	11	12	13	15	7	7	7	7	8	9	9	10	10	11
65-69	≥180	13	14	16	17	19	17	19	21	23	26	10	10	11	11	12	14	15	16	17	18
	160-179	10	12	13	14	16	14	15	17	19	21	8	9	9	10	10	12	13	13	14	15
	140-159	8	9	10	12	13	11	13	14	16	18	7	7	8	8	8	10	11	11	12	13
	120-139	7	8	8	9	10	9	10	11	13	14	6	6	6	7	7	8	9	9	10	11
	<120	6	6	7	8	8	7	8	9	10	12	5	5	5	6	6	7	7	8	8	9
60-64	≥180	10	11	12	14	16	14	16	18	20	23	7	8	8	9	10	12	13	14	15	16
	160-179	8	9	10	11	13	11	13	14	16	18	6	7	7	7	8	10	11	11	12	13
	140-159	6	7	8	9	10	9	10	11	13	15	5	5	6	6	7	8	9	9	10	11
	120-139	5	6	6	7	8	7	8	9	10	12	4	4	5	5	5	7	7	8	8	9
	<120	4	4	5	6	6	6	6	7	8	9	3	4	4	4	4	5	6	6	7	7
55-59	≥180	8	9	10	11	13	12	13	15	17	19	6	6	7	7	8	10	11	12	13	14
	160-179	6	7	8	9	10	9	10	12	13	15	5	5	5	6	6	8	9	9	10	11
	140-159	5	5	6	7	8	7	8	9	11	12	4	4	4	5	5	7	7	8	8	9
	120-139	4	4	5	5	6	6	6	7	8	10	3	3	3	4	4	5	6	6	7	7
	<120	3	3	4	4	5	4	5	6	6	7	2	3	3	3	3	4	5	5	5	6
50-54	≥180	6	7	8	9	10	10	11	13	14	17	5	5	5	6	6	8	9	10	11	12
	160-179	5	5	6	7	8	8	8	10	11	13	4	4	4	5	5	7	7	8	9	10
	140-159	4	4	5	5	6	6	6	7	9	10	3	3	3	4	4	5	6	6	7	8
	120-139	3	3	3	4	5	4	5	6	7	8	2	2	3	3	3	4	5	5	5	6
	<120	2	2	3	3	3	3	4	4	5	6	2	2	2	2	2	3	4	4	4	5
45-49	≥180	5	5	6	7	8	8	9	11	12	14	4	4	4	5	5	7	8	8	9	10
	160-179	4	4	5	5	6	6	7	8	9	11	3	3	3	4	4	5	6	7	7	8
	140-159	3	3	3	4	5	5	5	6	7	8	2	2	2	3	3	4	5	5	6	6
	120-139	2	2	3	3	3	3	4	5	5	6	2	2	2	2	2	3	4	4	4	5
	<120	1	2	2	2	3	3	3	3	4	5	1	1	1	2	2	3	3	3	3	4
40-44	≥180	4	4	5	6	6	7	8	9	10	12	3	3	3	4	4	6	6	7	8	9
	160-179	3	3	4	4	5	5	6	7	8	9	2	2	2	3	3	4	5	5	6	7
	140-159	2	2	3	3	4	4	4	5	6	7	2	2	2	2	2	3	4	4	5	5
	120-139	1	2	2	2	3	3	3	4	4	5	1	1	1	2	2	3	3	3	4	4
	<120	1	1	1	2	2	2	2	3	3	4	1	1	1	1	1	2	2	2	3	3

Total cholesterol (mmol/l)

Andean Latin America

Risk Level: <5% | 5% to <10% | 10% to <20% | 20% to <30% | ≥30%

People with Diabetes

Age (years)	SBP (mmHg)	Men Non-smoker					Men Smoker					Women Non-smoker					Women Smoker				
	Total cholesterol (mmol/l)	<4	4-4.9	5-5.9	6-6.9	≥ 7	<4	4-4.9	5-5.9	6-6.9	≥ 7	<4	4-4.9	5-5.9	6-6.9	≥ 7	<4	4-4.9	5-5.9	6-6.9	≥ 7
70-74	≥180	22	24	27	29	32	28	30	33	36	40	18	19	20	21	22	25	26	28	29	31
	160-179	18	20	22	25	27	23	25	28	31	34	16	16	17	18	19	22	23	24	25	26
	140-159	15	17	19	21	23	19	21	24	26	29	13	14	15	16	16	18	19	20	21	23
	120-139	13	14	15	17	19	16	18	20	22	24	11	12	13	13	14	16	17	17	18	19
	<120	10	12	13	14	16	13	15	16	18	20	10	10	11	11	12	13	14	15	16	17
65-69	≥180	18	20	22	25	28	24	27	30	33	37	15	16	17	18	19	22	24	25	27	28
	160-179	15	17	18	21	23	20	22	25	28	31	13	13	14	15	16	19	20	21	23	24
	140-159	12	14	15	17	19	16	18	20	23	26	11	11	12	13	14	16	17	18	19	20
	120-139	10	11	12	14	16	13	15	17	19	21	9	9	10	11	11	13	14	15	16	17
	<120	8	9	10	11	13	11	12	14	15	17	7	8	8	9	9	11	12	13	14	14
60-64	≥180	15	17	19	21	24	21	24	27	30	34	12	13	14	15	16	20	21	23	25	26
	160-179	12	14	15	17	20	17	19	22	25	28	10	11	12	13	14	16	18	19	21	22
	140-159	10	11	12	14	16	14	16	18	20	23	8	9	10	10	11	14	15	16	17	18
	120-139	8	9	10	11	13	11	12	14	16	18	7	7	8	9	9	11	12	13	14	15
	<120	6	7	8	9	10	9	10	11	13	15	6	6	7	7	8	9	10	11	12	13
55-59	≥180	13	14	16	18	21	19	21	24	27	31	10	11	12	13	14	18	19	21	23	25
	160-179	10	11	13	14	16	15	17	19	22	25	8	9	10	10	11	14	16	17	19	20
	140-159	8	9	10	11	13	12	13	15	18	20	7	7	8	8	9	12	13	14	15	17
	120-139	6	7	8	9	10	9	10	12	14	16	5	6	6	7	7	10	10	11	12	14
	<120	5	5	6	7	8	7	8	9	11	13	4	5	5	6	6	8	8	9	10	11
50-54	≥180	11	12	13	15	18	17	19	22	25	29	8	9	10	11	12	16	17	19	21	23
	160-179	8	9	10	12	14	13	15	17	20	23	7	7	8	9	10	13	14	15	17	19
	140-159	6	7	8	9	11	10	11	13	15	18	5	6	6	7	8	10	11	12	13	15
	120-139	5	5	6	7	8	8	9	10	12	14	4	5	5	5	6	8	9	10	11	12
	<120	4	4	5	5	6	6	7	8	9	11	3	4	4	4	5	6	7	8	9	10
45-49	≥180	9	10	11	13	15	15	17	19	23	27	7	8	8	9	10	14	15	17	19	21
	160-179	7	8	9	10	12	11	13	15	17	21	5	6	7	7	8	11	12	13	15	17
	140-159	5	6	6	7	9	8	10	11	13	16	4	5	5	6	6	9	10	11	12	13
	120-139	4	4	5	6	7	6	7	9	10	12	3	4	4	4	5	7	7	8	9	11
	<120	3	3	4	4	5	5	6	6	8	9	3	3	3	3	4	5	6	7	7	8
40-44	≥180	8	8	10	11	13	13	15	17	20	24	6	6	7	8	9	12	14	15	17	20
	160-179	6	6	7	8	10	10	11	13	15	19	4	5	5	6	7	10	11	12	14	15
	140-159	4	5	5	6	7	7	8	10	12	14	3	4	4	5	5	7	8	9	11	12
	120-139	3	3	4	4	5	5	6	7	9	10	3	3	3	4	4	6	6	7	8	9
	<120	2	2	3	3	4	4	5	5	6	8	2	2	2	3	3	4	5	6	6	7

Total cholesterol (mmol/l)

Central Latin America

Risk Level: <5% | 5% to <10% | 10% to <20% | 20% to <30% | ≥30%

People without Diabetes

Age (years)	SBP (mmHg)	Men Non-smoker <4	4-4.9	5-5.9	6-6.9	≥ 7	Men Smoker <4	4-4.9	5-5.9	6-6.9	≥ 7	Women Non-smoker <4	4-4.9	5-5.9	6-6.9	≥ 7	Women Smoker <4	4-4.9	5-5.9	6-6.9	≥ 7
70-74	≥180	18	20	22	25	27	23	26	28	31	35	14	15	16	17	18	20	21	22	24	25
	160-179	15	17	18	21	23	19	21	24	26	29	12	13	13	14	15	17	18	19	20	21
	140-159	12	14	15	17	19	16	18	20	22	24	10	11	11	12	13	14	15	16	17	18
	120-139	10	11	12	14	16	13	14	16	18	20	9	9	10	10	11	12	13	14	14	15
	<120	8	9	10	11	13	11	12	13	15	17	7	8	8	8	9	10	11	11	12	13
65-69	≥180	14	16	18	20	22	19	21	24	27	30	11	12	12	13	14	17	18	19	20	22
	160-179	11	13	14	16	18	16	17	19	22	25	9	10	10	11	12	14	15	16	17	18
	140-159	9	10	11	13	15	13	14	16	18	20	7	8	8	9	10	12	12	13	14	15
	120-139	7	8	9	10	12	10	11	13	14	16	6	7	7	7	8	10	10	11	12	13
	<120	6	7	7	8	9	8	9	10	12	13	5	5	6	6	7	8	8	9	10	10
60-64	≥180	11	12	14	16	18	16	18	20	23	26	8	9	10	10	11	14	15	16	17	19
	160-179	9	10	11	12	14	13	14	16	18	21	7	7	8	8	9	11	12	13	14	15
	140-159	7	8	9	10	11	10	11	13	15	17	5	6	6	7	7	9	10	11	12	13
	120-139	5	6	7	8	9	8	9	10	12	13	4	5	5	6	6	7	8	9	9	10
	<120	4	5	5	6	7	6	7	8	9	11	4	4	4	4	5	6	7	7	8	8
55-59	≥180	8	9	11	12	14	13	15	17	19	22	6	7	7	8	9	11	12	13	15	16
	160-179	6	7	8	10	11	10	11	13	15	18	5	5	6	6	7	9	10	11	12	13
	140-159	5	6	6	7	9	8	9	10	12	14	4	4	5	5	6	7	8	9	10	10
	120-139	4	4	5	6	7	6	7	8	9	11	3	3	4	4	4	6	6	7	8	8
	<120	3	3	4	4	5	5	5	6	7	8	3	3	3	3	4	5	5	6	6	7
50-54	≥180	7	7	8	10	11	11	12	14	16	19	5	5	6	6	7	9	10	11	12	14
	160-179	5	6	6	7	9	8	9	11	13	15	4	4	4	5	5	7	8	9	10	11
	140-159	4	4	5	6	7	6	7	8	10	11	3	3	3	4	4	6	6	7	8	9
	120-139	3	3	4	4	5	5	5	6	7	9	2	2	3	3	3	4	5	5	6	7
	<120	2	2	3	3	4	3	4	5	6	7	2	2	2	2	3	4	4	4	5	5
45-49	≥180	5	6	7	8	9	9	10	12	14	17	4	4	4	5	5	8	8	9	11	12
	160-179	4	4	5	6	7	6	7	9	10	12	3	3	3	4	4	6	7	7	8	9
	140-159	3	3	4	4	5	5	6	7	8	9	2	2	3	3	3	5	5	6	6	7
	120-139	2	2	3	3	4	4	4	5	6	7	2	2	2	2	2	3	4	4	5	6
	<120	1	2	2	2	3	3	3	4	4	5	1	1	2	2	2	3	3	3	4	4
40-44	≥180	4	4	5	6	7	7	8	10	12	14	3	3	3	4	4	6	7	8	9	10
	160-179	3	3	4	4	5	5	6	7	9	10	2	2	3	3	3	5	5	6	7	8
	140-159	2	2	3	3	4	4	4	5	6	8	2	2	2	2	2	4	4	5	5	6
	120-139	1	2	2	2	3	3	3	4	5	6	1	1	1	2	2	3	3	3	4	5
	<120	1	1	1	2	2	2	2	3	3	4	1	1	1	1	1	2	2	3	3	3

Total cholesterol (mmol/l)

Central Latin America

Risk Level: <5% | 5% to <10% | 10% to <20% | 20% to <30% | ≥30%

People with Diabetes

Age (years)	SBP (mmHg)	Men Non-smoker <4	4-4.9	5-5.9	6-6.9	≥ 7	Men Smoker <4	4-4.9	5-5.9	6-6.9	≥ 7	Women Non-smoker <4	4-4.9	5-5.9	6-6.9	≥ 7	Women Smoker <4	4-4.9	5-5.9	6-6.9	≥ 7
70-74	≥180	25	28	31	34	38	32	35	38	42	47	22	23	24	26	27	30	32	33	35	37
	160-179	21	23	26	28	32	26	29	32	36	40	18	19	21	22	23	26	27	29	30	32
	140-159	17	19	21	24	26	22	24	27	30	34	16	16	17	18	20	22	23	24	26	28
	120-139	14	16	18	20	22	18	20	23	25	28	13	14	15	16	16	19	20	21	22	23
	<120	12	13	14	16	18	15	17	19	21	24	11	12	12	13	14	16	17	18	19	20
65-69	≥180	21	23	26	29	32	28	31	35	39	43	18	19	20	21	23	27	28	30	32	35
	160-179	17	19	21	24	27	23	25	29	32	36	15	16	17	18	19	22	24	26	27	29
	140-159	13	15	17	19	22	18	21	23	26	30	12	13	14	15	16	19	20	21	23	25
	120-139	11	12	14	16	18	15	17	19	22	25	10	11	12	12	13	16	17	18	19	21
	<120	9	10	11	13	14	12	14	15	18	20	8	9	10	10	11	13	14	15	16	17
60-64	≥180	17	19	22	24	28	24	27	31	35	40	14	15	17	18	19	24	25	27	30	32
	160-179	13	15	17	20	22	19	22	25	29	33	12	13	14	15	16	19	21	23	25	27
	140-159	11	12	14	16	18	16	18	20	23	27	10	10	11	12	13	16	17	19	20	22
	120-139	8	9	11	12	14	12	14	16	19	21	8	8	9	10	11	13	14	15	17	18
	<120	7	7	9	10	11	10	11	13	15	17	6	7	7	8	9	11	12	13	14	15
55-59	≥180	14	16	18	21	24	21	24	28	32	37	12	13	14	15	16	21	23	25	27	30
	160-179	11	12	14	16	19	17	19	22	25	30	9	10	11	12	13	17	18	20	22	25
	140-159	8	9	11	13	15	13	15	17	20	24	7	8	9	10	11	14	15	16	18	20
	120-139	6	7	8	10	12	10	12	14	16	19	6	6	7	8	9	11	12	13	15	16
	<120	5	6	7	8	9	8	9	11	12	15	5	5	6	6	7	9	10	11	12	13
50-54	≥180	12	13	15	17	20	19	21	25	29	34	9	10	11	13	14	18	20	22	25	28
	160-179	9	10	11	13	16	14	17	19	23	27	7	8	9	10	11	15	16	18	20	22
	140-159	7	8	9	10	12	11	13	15	17	21	6	6	7	8	9	12	13	14	16	18
	120-139	5	6	7	8	9	8	10	11	13	16	5	5	6	6	7	9	10	11	13	14
	<120	4	4	5	6	7	6	7	9	10	12	4	4	4	5	5	7	8	9	10	11
45-49	≥180	10	11	12	15	17	16	19	22	26	31	8	8	9	10	12	16	18	20	23	26
	160-179	7	8	9	11	13	12	14	17	20	24	6	7	7	8	9	13	14	16	18	20
	140-159	5	6	7	8	10	9	11	13	15	18	5	5	6	6	7	10	11	12	14	16
	120-139	4	4	5	6	7	7	8	10	11	14	3	4	4	5	5	8	9	10	11	13
	<120	3	3	4	5	6	5	6	7	9	11	3	3	3	4	4	6	7	8	9	10
40-44	≥180	8	9	10	12	15	15	17	20	23	28	6	7	8	9	10	14	16	18	21	24
	160-179	6	7	8	9	11	11	12	15	18	21	5	5	6	7	8	11	12	14	16	19
	140-159	4	5	6	7	8	8	9	11	13	16	4	4	4	5	6	8	9	11	12	14
	120-139	3	3	4	5	6	6	7	8	10	12	3	3	3	4	4	6	7	8	10	11
	<120	2	2	3	4	4	4	5	6	7	9	2	2	3	3	3	5	5	6	7	9

Total cholesterol (mmol/l)

Tropical Latin America

Risk Level: <5% | 5% to <10% | 10% to <20% | 20% to <30% | ≥30%

People without Diabetes

Age (years)	SBP (mmHg)	Men Non-smoker <4	4-4.9	5-5.9	6-6.9	≥ 7	Men Smoker <4	4-4.9	5-5.9	6-6.9	≥ 7	Women Non-smoker <4	4-4.9	5-5.9	6-6.9	≥ 7	Women Smoker <4	4-4.9	5-5.9	6-6.9	≥ 7
70-74	≥180	18	20	21	23	25	23	24	26	29	31	13	13	14	14	15	17	18	19	19	20
	160-179	15	17	18	19	21	19	21	22	24	26	11	11	12	12	13	15	15	16	17	17
	140-159	13	14	15	16	18	16	17	19	20	22	9	10	10	10	11	13	13	14	14	15
	120-139	11	11	12	14	15	13	14	16	17	18	8	8	8	9	9	11	11	12	12	13
	<120	9	9	10	11	12	11	12	13	14	15	7	7	7	8	8	9	10	10	10	11
65-69	≥180	15	16	17	19	21	19	21	23	25	27	10	10	11	11	12	14	15	16	17	18
	160-179	12	13	14	15	17	16	17	19	20	22	8	9	9	10	10	12	13	13	14	15
	140-159	10	11	12	13	14	13	14	15	17	18	7	7	8	8	8	10	11	11	12	13
	120-139	8	9	9	10	11	10	11	12	14	15	6	6	6	7	7	9	9	9	10	11
	<120	6	7	8	8	9	8	9	10	11	12	5	5	5	6	6	7	8	8	8	9
60-64	≥180	12	13	14	15	17	16	18	19	21	23	8	8	9	9	10	12	13	14	14	15
	160-179	9	10	11	12	14	13	14	16	17	19	6	7	7	7	8	10	11	11	12	13
	140-159	7	8	9	10	11	10	11	12	14	15	5	6	6	6	7	8	9	9	10	11
	120-139	6	6	7	8	9	8	9	10	11	12	4	5	5	5	5	7	7	8	8	9
	<120	5	5	6	6	7	7	7	8	9	10	4	4	4	4	4	6	6	6	7	7
55-59	≥180	9	10	11	12	14	14	15	16	18	20	6	6	7	7	8	10	11	12	12	13
	160-179	7	8	9	10	11	11	12	13	14	16	5	5	6	6	6	8	9	9	10	11
	140-159	6	6	7	8	8	8	9	10	11	13	4	4	4	5	5	7	7	8	8	9
	120-139	4	5	5	6	7	6	7	8	9	10	3	3	4	4	4	5	6	6	7	7
	<120	3	4	4	5	5	5	6	6	7	8	3	3	3	3	3	4	5	5	5	6
50-54	≥180	7	8	9	10	11	11	13	14	16	18	5	5	5	6	6	9	9	10	11	12
	160-179	6	6	7	8	9	9	10	11	12	14	4	4	4	5	5	7	7	8	9	9
	140-159	4	5	5	6	7	7	7	8	9	11	3	3	3	4	4	5	6	6	7	7
	120-139	3	4	4	4	5	5	6	6	7	8	2	3	3	3	3	4	5	5	5	6
	<120	2	3	3	3	4	4	4	5	6	6	2	2	2	2	2	3	4	4	4	5
45-49	≥180	6	6	7	8	9	10	11	12	13	15	4	4	4	5	5	7	8	8	9	10
	160-179	4	5	5	6	7	7	8	9	10	12	3	3	3	4	4	6	6	7	7	8
	140-159	3	4	4	4	5	5	6	7	8	9	2	2	3	3	3	4	5	5	6	6
	120-139	2	3	3	3	4	4	4	5	6	7	2	2	2	2	2	3	4	4	4	5
	<120	2	2	2	3	3	3	3	4	4	5	1	1	2	2	2	3	3	3	3	4
40-44	≥180	5	5	6	6	7	8	9	10	11	13	3	3	4	4	4	6	7	7	8	9
	160-179	3	4	4	5	5	6	7	7	8	10	2	2	3	3	3	5	5	6	6	7
	140-159	2	3	3	3	4	4	5	5	6	7	2	2	2	2	2	4	4	4	5	5
	120-139	2	2	2	3	3	3	4	4	5	6	1	1	2	2	2	3	3	3	4	4
	<120	1	1	2	2	2	2	3	3	3	4	1	1	1	1	1	2	2	2	3	3

Total cholesterol (mmol/l)

Tropical Latin America

Risk Level: <5% | 5% to <10% | 10% to <20% | 20% to <30% | ≥30%

People with Diabetes

Age (years)	SBP (mmHg)	Men Non-smoker <4	4-4.9	5-5.9	6-6.9	≥ 7	Men Smoker <4	4-4.9	5-5.9	6-6.9	≥ 7	Women Non-smoker <4	4-4.9	5-5.9	6-6.9	≥ 7	Women Smoker <4	4-4.9	5-5.9	6-6.9	≥ 7
70-74	≥180	24	26	28	30	33	29	32	34	37	40	18	19	20	21	22	25	26	27	28	29
	160-179	20	22	23	26	28	25	27	29	31	34	16	16	17	18	19	21	22	23	24	25
	140-159	17	18	20	21	23	21	22	24	27	29	13	14	15	15	16	18	19	20	21	22
	120-139	14	15	16	18	20	17	19	21	22	25	11	12	12	13	14	16	16	17	18	19
	<120	12	13	14	15	16	14	16	17	19	21	10	10	11	11	12	13	14	15	15	16
65-69	≥180	20	22	24	26	29	26	29	31	34	37	15	16	17	18	19	22	23	24	26	27
	160-179	17	18	20	22	24	22	24	26	28	31	13	13	14	15	16	19	20	21	22	23
	140-159	14	15	16	18	20	18	20	21	24	26	11	11	12	13	13	16	17	18	19	20
	120-139	11	12	13	15	16	15	16	18	19	22	9	9	10	11	11	13	14	15	16	17
	<120	9	10	11	12	13	12	13	14	16	18	8	8	8	9	9	11	12	12	13	14
60-64	≥180	17	19	21	23	25	24	26	28	31	34	13	13	14	15	16	20	21	22	24	26
	160-179	14	15	17	18	20	19	21	23	25	28	10	11	12	12	13	16	17	19	20	21
	140-159	11	12	13	15	16	15	17	19	21	23	9	9	10	10	11	14	14	15	17	18
	120-139	9	10	11	12	13	12	14	15	17	19	7	7	8	8	9	11	12	13	14	15
	<120	7	8	9	10	11	10	11	12	14	15	6	6	7	7	7	9	10	11	11	12
55-59	≥180	15	16	18	19	22	21	23	26	28	32	10	11	12	13	14	18	19	20	22	24
	160-179	12	13	14	15	17	17	18	20	23	26	8	9	10	10	11	14	15	17	18	20
	140-159	9	10	11	12	14	13	15	16	18	21	7	7	8	8	9	12	13	14	15	16
	120-139	7	8	9	10	11	10	12	13	15	16	6	6	6	7	7	9	10	11	12	13
	<120	5	6	7	8	9	8	9	10	11	13	4	5	5	6	6	8	8	9	10	11
50-54	≥180	13	14	15	17	19	19	21	23	26	29	9	9	10	11	12	16	17	18	20	22
	160-179	10	11	12	13	15	15	16	18	20	23	7	7	8	9	9	13	14	15	16	18
	140-159	7	8	9	10	11	11	13	14	16	18	6	6	6	7	8	10	11	12	13	14
	120-139	6	6	7	8	9	9	10	11	12	14	4	5	5	5	6	8	9	10	10	12
	<120	4	5	5	6	7	7	7	8	10	11	3	4	4	4	5	6	7	8	8	9
45-49	≥180	11	12	13	14	16	17	19	21	24	27	7	8	9	9	10	14	15	17	19	21
	160-179	8	9	10	11	12	13	14	16	18	21	6	6	7	7	8	11	12	13	15	16
	140-159	6	7	7	8	9	10	11	12	14	16	4	5	5	6	6	9	9	10	12	13
	120-139	4	5	5	6	7	7	8	9	11	12	3	4	4	4	5	7	7	8	9	10
	<120	3	4	4	5	5	5	6	7	8	10	3	3	3	3	4	5	6	6	7	8
40-44	≥180	9	10	11	12	14	15	17	19	22	25	6	7	7	8	9	13	14	15	17	19
	160-179	7	7	8	9	11	11	13	14	16	19	5	5	6	6	7	10	11	12	13	15
	140-159	5	5	6	7	8	8	9	11	12	14	4	4	4	5	5	7	8	9	10	12
	120-139	4	4	4	5	6	6	7	8	9	11	3	3	3	4	4	6	6	7	8	9
	<120	3	3	3	4	4	5	5	6	7	8	2	2	2	3	3	4	5	5	6	7

Total cholesterol (mmol/l)

East Asia

Risk Level: <5% | 5% to <10% | 10% to <20% | 20% to <30% | ≥30%

People without Diabetes

Age (years)	SBP (mmHg)	Men										Women									
		Non-smoker					Smoker					Non-smoker					Smoker				
		<4	4-4.9	5-5.9	6-6.9	≥ 7	<4	4-4.9	5-5.9	6-6.9	≥ 7	<4	4-4.9	5-5.9	6-6.9	≥ 7	<4	4-4.9	5-5.9	6-6.9	≥ 7
	≥180	33	35	37	40	43	41	43	46	49	52	26	26	27	28	29	35	36	37	38	39
	160-179	27	29	31	33	35	33	36	38	41	44	22	22	23	24	25	30	31	31	33	34
70-74	140-159	22	23	25	27	29	27	29	31	34	36	19	19	20	20	21	25	26	27	28	29
	120-139	18	19	20	22	23	22	24	25	27	30	16	16	17	17	18	21	22	23	24	24
	<120	14	15	16	17	19	18	19	21	22	24	13	14	14	14	15	18	19	19	20	21
	≥180	26	28	30	32	35	34	37	39	42	45	20	21	22	22	23	29	30	32	33	34
	160-179	21	22	24	26	28	27	29	31	34	37	17	17	18	19	19	24	25	26	28	29
65-69	140-159	16	17	19	20	22	22	23	25	27	30	14	14	15	15	16	20	21	22	23	24
	120-139	13	14	15	16	17	17	18	20	22	24	11	12	12	13	13	17	17	18	19	20
	<120	10	11	11	12	13	13	14	16	17	19	9	10	10	10	11	14	14	15	16	16
	≥180	21	22	24	25	28	29	31	33	36	39	16	16	17	18	19	25	26	27	28	30
	160-179	16	17	18	20	21	22	24	26	28	31	13	13	14	14	15	20	21	22	23	25
60-64	140-159	12	13	14	15	16	17	18	20	22	24	10	11	11	12	12	16	17	18	19	20
	120-139	9	10	11	11	13	13	14	15	17	19	8	9	9	9	10	13	14	14	15	16
	<120	7	7	8	9	10	10	11	12	13	14	7	7	7	7	8	10	11	12	12	13
	≥180	16	17	19	20	22	24	26	28	30	33	12	13	14	14	15	21	22	23	25	26
	160-179	12	13	14	15	16	18	19	21	23	25	10	10	11	11	12	16	17	18	20	21
55-59	140-159	9	10	10	11	12	14	15	16	17	19	8	8	8	9	9	13	14	15	15	17
	120-139	7	7	8	8	9	10	11	12	13	14	6	6	6	7	7	10	11	11	12	13
	<120	5	5	6	6	7	7	8	9	10	11	5	5	5	5	6	8	8	9	10	10
	≥180	13	14	15	16	17	20	22	23	25	28	10	10	11	11	12	17	18	20	21	23
	160-179	9	10	11	11	13	15	16	17	19	21	7	8	8	9	9	13	14	15	16	18
50-54	140-159	7	7	8	8	9	11	11	12	14	15	6	6	6	7	7	10	11	12	13	14
	120-139	5	5	5	6	7	8	8	9	10	11	4	4	5	5	5	8	8	9	10	11
	<120	3	4	4	4	5	5	6	6	7	8	3	3	4	4	4	6	6	7	7	8
	≥180	10	11	11	12	13	17	18	19	21	24	8	8	8	9	10	14	16	17	18	20
	160-179	7	7	8	9	10	12	13	14	15	17	6	6	6	7	7	11	12	13	14	15
45-49	140-159	5	5	6	6	7	8	9	10	11	12	4	4	5	5	5	8	9	9	10	11
	120-139	3	4	4	4	5	6	6	7	8	9	3	3	3	4	4	6	6	7	8	8
	<120	2	2	3	3	3	4	4	5	5	6	2	2	3	3	3	4	5	5	6	6
	≥180	8	8	9	10	10	14	15	16	18	20	6	6	7	7	8	12	13	14	15	17
	160-179	5	6	6	6	7	10	10	11	12	14	4	5	5	5	6	9	10	10	11	13
40-44	140-159	4	4	4	4	5	6	7	8	8	10	3	3	3	4	4	6	7	8	8	9
	120-139	2	3	3	3	3	4	5	5	6	7	2	2	2	3	3	5	5	6	6	7
	<120	2	2	2	2	2	3	3	4	4	5	2	2	2	2	2	3	4	4	4	5

Total cholesterol (mmol/l)

East Asia

Risk Level: <5% | 5% to <10% | 10% to <20% | 20% to <30% | ≥30%

People with Diabetes

Age (years)	SBP (mmHg)	Men										Women									
		Non-smoker					Smoker					Non-smoker					Smoker				
		<4	4-4.9	5-5.9	6-6.9	≥ 7	<4	4-4.9	5-5.9	6-6.9	≥ 7	<4	4-4.9	5-5.9	6-6.9	≥ 7	<4	4-4.9	5-5.9	6-6.9	≥ 7
	≥180	42	44	47	51	54	51	54	57	61	65	37	38	39	40	42	48	50	51	53	55
	160-179	35	37	40	43	46	43	45	49	52	56	32	33	34	35	36	42	43	45	46	48
70-74	140-159	28	30	33	35	38	35	38	41	44	47	27	28	29	30	31	36	38	39	40	42
	120-139	23	25	27	29	31	29	31	34	36	39	23	24	24	25	26	31	32	33	35	36
	<120	18	20	22	23	26	23	25	27	30	32	19	20	21	21	22	27	27	28	30	31
	≥180	36	38	41	44	48	46	49	53	56	60	31	32	33	35	36	44	46	47	49	52
	160-179	29	31	33	36	39	38	40	43	47	51	26	27	28	29	31	37	39	40	42	44
65-69	140-159	23	25	27	29	31	30	33	35	38	42	22	22	23	24	26	31	33	34	36	38
	120-139	18	19	21	23	25	24	26	28	31	34	18	19	19	20	21	26	27	29	30	32
	<120	14	15	17	18	20	19	21	23	25	27	15	15	16	17	18	22	23	24	25	26
	≥180	31	33	35	38	41	42	45	48	52	56	26	27	28	30	31	40	41	44	46	48
	160-179	24	26	28	30	33	33	36	39	42	46	21	22	23	24	26	33	34	36	38	41
60-64	140-159	18	20	21	23	26	26	28	31	33	37	17	18	19	20	21	27	28	30	32	34
	120-139	14	15	16	18	20	20	22	24	26	29	14	14	15	16	17	22	23	25	26	28
	<120	11	12	13	14	15	15	17	18	20	23	11	12	12	13	14	18	19	20	21	23
	≥180	26	28	30	33	36	38	41	44	48	52	22	23	24	25	27	36	38	40	42	45
	160-179	20	21	23	25	28	29	32	34	38	41	17	18	19	20	22	29	31	33	35	37
55-59	140-159	15	16	17	19	21	22	24	26	29	32	14	14	15	16	17	23	25	26	28	30
	120-139	11	12	13	14	16	17	18	20	22	25	11	11	12	13	14	18	20	21	23	24
	<120	8	9	10	11	12	12	14	15	17	19	8	9	9	10	11	15	16	17	18	19
	≥180	23	24	26	28	31	35	37	40	44	48	18	19	20	22	23	32	34	36	39	42
	160-179	17	18	19	21	23	26	28	30	33	37	14	15	16	17	18	25	27	29	31	34
50-54	140-159	12	13	14	15	17	19	21	23	25	28	11	11	12	13	14	20	21	23	25	27
	120-139	9	9	10	11	12	14	15	17	19	21	8	9	9	10	11	15	16	18	19	21
	<120	6	7	7	8	9	10	11	12	14	16	6	7	7	8	8	12	13	14	15	17
	≥180	20	21	22	24	26	31	34	36	40	44	15	16	17	18	20	29	31	33	36	39
	160-179	14	15	16	17	19	23	25	27	30	33	11	12	13	14	15	22	24	26	28	31
45-49	140-159	10	10	11	12	14	16	18	19	22	25	8	9	10	11	11	17	18	20	22	24
	120-139	7	7	8	9	10	11	12	14	16	18	6	7	7	8	9	13	14	15	17	19
	<120	5	5	5	6	7	8	9	10	11	13	5	5	5	6	6	10	10	11	13	14
	≥180	17	18	19	20	22	29	30	33	36	40	13	13	15	16	17	26	28	30	33	37
	160-179	11	12	13	14	16	20	22	24	26	30	9	10	11	12	13	19	21	23	25	28
40-44	140-159	8	8	9	10	11	14	15	17	19	21	7	7	8	8	9	14	16	17	19	21
	120-139	5	6	6	7	8	10	10	11	13	15	5	5	6	6	7	10	12	13	14	16
	<120	4	4	4	5	5	6	7	8	9	11	3	4	4	4	5	8	8	9	11	12

Total cholesterol (mmol/l)

South Asia

Risk Level: <5% | 5% to <10% | 10% to <20% | 20% to <30% | ≥30%

People without Diabetes

Total cholesterol (mmol/l)

Age (years)	SBP (mmHg)	Men Non-smoker					Men Smoker					Women Non-smoker					Women Smoker				
		<4	4-4.9	5-5.9	6-6.9	≥ 7	<4	4-4.9	5-5.9	6-6.9	≥ 7	<4	4-4.9	5-5.9	6-6.9	≥ 7	<4	4-4.9	5-5.9	6-6.9	≥ 7
70-74	≥180	22	24	27	30	33	28	31	34	37	41	18	19	20	21	22	25	26	28	29	30
	160-179	18	20	23	25	28	23	26	28	31	35	15	16	17	18	19	21	22	24	25	26
	140-159	15	17	19	21	23	19	21	24	27	29	13	14	14	15	16	18	19	20	21	22
	120-139	13	14	16	17	19	16	18	20	22	25	11	12	12	13	13	15	16	17	18	19
	<120	10	12	13	14	16	13	15	17	18	21	9	10	10	11	11	13	14	14	15	16
65-69	≥180	17	19	21	24	27	23	26	29	32	36	14	15	16	17	18	21	22	23	25	27
	160-179	14	16	18	20	22	19	21	24	27	30	12	12	13	14	15	17	19	20	21	22
	140-159	11	13	14	16	18	16	17	20	22	25	10	10	11	12	12	15	16	17	18	19
	120-139	9	10	12	13	15	13	14	16	18	20	8	8	9	10	10	12	13	14	15	16
	<120	8	8	9	11	12	10	12	13	15	17	7	7	7	8	8	10	11	11	12	13
60-64	≥180	13	15	17	19	22	19	22	24	28	32	11	11	12	13	14	17	19	20	21	23
	160-179	11	12	14	15	18	15	17	20	23	26	9	9	10	11	12	14	15	16	18	19
	140-159	9	10	11	12	14	12	14	16	18	21	7	8	8	9	9	12	13	14	15	16
	120-139	7	8	9	10	11	10	11	13	15	17	6	6	7	7	8	10	10	11	12	13
	<120	5	6	7	8	9	8	9	10	12	14	5	5	5	6	6	8	8	9	10	11
55-59	≥180	10	12	13	15	18	16	18	21	24	27	8	9	9	10	11	14	16	17	18	20
	160-179	8	9	11	12	14	12	14	16	19	22	7	7	8	8	9	12	13	14	15	16
	140-159	6	7	8	9	11	10	11	13	15	18	5	6	6	7	7	9	10	11	12	13
	120-139	5	6	6	7	9	8	9	10	12	14	4	4	5	5	6	7	8	9	10	11
	<120	4	4	5	6	7	6	7	8	9	11	3	4	4	4	5	6	6	7	8	9
50-54	≥180	8	9	11	12	14	13	15	17	20	24	6	7	7	8	9	12	13	14	16	17
	160-179	6	7	8	9	11	10	12	14	16	19	5	5	6	6	7	9	10	11	13	14
	140-159	5	5	6	7	9	8	9	10	12	15	4	4	5	5	5	7	8	9	10	11
	120-139	4	4	5	6	7	6	7	8	9	11	3	3	4	4	4	6	6	7	8	9
	<120	3	3	4	4	5	5	5	6	7	9	2	3	3	3	3	5	5	6	6	7
45-49	≥180	6	7	8	10	11	11	13	15	17	21	5	5	6	6	7	10	11	12	13	15
	160-179	5	5	6	7	9	8	9	11	13	16	4	4	4	5	5	8	8	9	10	12
	140-159	4	4	5	6	7	6	7	8	10	12	3	3	3	4	4	6	7	7	8	9
	120-139	3	3	4	4	5	5	5	6	8	9	2	2	3	3	3	5	5	6	6	7
	<120	2	2	3	3	4	3	4	5	6	7	2	2	2	2	2	3	4	4	5	6
40-44	≥180	5	6	7	8	9	9	10	12	15	18	4	4	4	5	6	8	9	10	11	13
	160-179	4	4	5	6	7	7	8	9	11	13	3	3	3	4	4	6	7	8	9	10
	140-159	3	3	4	4	5	5	6	7	8	10	2	2	3	3	3	5	5	6	7	8
	120-139	2	2	3	3	4	4	4	5	6	8	2	2	2	2	2	3	4	4	5	6
	<120	1	2	2	2	3	3	3	4	5	6	1	1	1	2	2	3	3	3	4	5

South Asia

Risk Level: <5% | 5% to <10% | 10% to <20% | 20% to <30% | ≥30%

People with Diabetes

Total cholesterol (mmol/l)

Age (years)	SBP (mmHg)	Men Non-smoker					Men Smoker					Women Non-smoker					Women Smoker				
		<4	4-4.9	5-5.9	6-6.9	≥ 7	<4	4-4.9	5-5.9	6-6.9	≥ 7	<4	4-4.9	5-5.9	6-6.9	≥ 7	<4	4-4.9	5-5.9	6-6.9	≥ 7
70-74	≥180	30	33	36	40	44	37	41	45	50	54	27	28	29	31	33	36	38	40	42	44
	160-179	25	28	31	34	38	32	35	39	43	47	23	24	25	27	28	31	33	35	36	38
	140-159	21	23	26	29	32	27	30	33	36	40	20	21	22	23	24	27	28	30	31	33
	120-139	18	19	22	24	27	22	25	28	31	34	17	17	18	19	20	23	24	26	27	28
	<120	15	16	18	20	23	19	21	23	26	29	14	15	16	16	17	20	21	22	23	24
65-69	≥180	25	28	31	35	39	33	37	41	45	51	22	23	25	26	28	32	34	36	39	41
	160-179	20	23	26	29	32	27	31	34	38	43	18	20	21	22	24	28	29	31	33	35
	140-159	17	19	21	24	27	23	25	29	32	36	15	16	17	19	20	23	25	26	28	30
	120-139	14	15	17	20	22	19	21	24	27	30	13	14	15	16	17	20	21	22	24	25
	<120	11	12	14	16	18	15	17	19	22	25	11	11	12	13	14	16	17	19	20	21
60-64	≥180	20	23	26	30	34	29	33	37	42	47	18	19	21	22	24	29	31	33	36	38
	160-179	16	19	21	24	28	24	27	30	35	39	15	16	17	18	20	24	26	28	30	32
	140-159	13	15	17	20	22	19	22	25	28	32	12	13	14	15	16	20	21	23	25	27
	120-139	11	12	14	16	18	15	18	20	23	27	10	11	12	12	13	16	18	19	21	23
	<120	8	10	11	13	15	12	14	16	19	22	8	9	9	10	11	14	15	16	17	19
55-59	≥180	17	19	22	25	29	25	29	33	38	44	15	16	17	19	20	26	28	30	33	36
	160-179	13	15	17	20	23	20	23	27	31	36	12	13	14	15	17	21	23	25	27	30
	140-159	10	12	14	16	19	16	19	21	25	29	10	10	11	12	13	17	19	20	22	25
	120-139	8	9	11	13	15	13	15	17	20	23	8	8	9	10	11	14	15	17	18	20
	<120	6	7	8	10	12	10	12	13	16	19	6	7	7	8	9	11	12	13	15	16
50-54	≥180	14	16	18	21	25	22	26	30	35	40	12	13	14	16	17	23	25	27	30	33
	160-179	11	12	14	17	20	18	20	24	28	32	10	10	11	13	14	18	20	22	25	27
	140-159	8	10	11	13	15	14	16	18	22	26	7	8	9	10	11	15	16	18	20	22
	120-139	6	7	9	10	12	11	12	14	17	20	6	6	7	8	9	12	13	14	16	18
	<120	5	6	7	8	9	8	9	11	13	16	5	5	6	6	7	9	10	11	13	14
45-49	≥180	12	13	15	18	21	20	23	27	31	37	10	11	12	13	15	20	22	25	28	31
	160-179	9	10	12	14	16	15	18	21	25	29	8	8	9	10	12	16	18	20	22	25
	140-159	7	8	9	11	13	12	13	16	19	23	6	6	7	8	9	12	14	16	18	20
	120-139	5	6	7	8	10	9	10	12	15	18	4	5	6	6	7	10	11	12	14	16
	<120	4	4	5	6	7	7	8	9	11	14	3	4	4	5	5	8	8	10	11	12
40-44	≥180	10	11	13	15	18	17	20	24	28	34	8	9	10	11	13	18	20	23	25	29
	160-179	7	8	10	11	14	13	15	18	22	27	6	7	8	9	10	14	16	18	20	23
	140-159	5	6	7	9	10	10	11	14	17	20	5	5	6	7	7	11	12	14	16	18
	120-139	4	5	5	6	8	7	9	10	12	15	3	4	4	5	6	8	9	11	12	14
	<120	3	3	4	5	6	5	6	8	9	12	3	3	3	4	4	6	7	8	9	11

Southeast Asia

Risk Level: <5% | 5% to <10% | 10% to <20% | 20% to <30% | ≥30%

People without Diabetes

Age (years)	SBP (mmHg)	Men										Women									
		Non-smoker					Smoker					Non-smoker					Smoker				
		<4	4-4.9	5-5.9	6-6.9	≥ 7	<4	4-4.9	5-5.9	6-6.9	≥ 7	<4	4-4.9	5-5.9	6-6.9	≥ 7	<4	4-4.9	5-5.9	6-6.9	≥ 7
70-74	≥180	24	26	29	32	35	30	33	36	40	44	18	19	20	21	22	26	27	28	30	31
	160-179	19	21	23	26	29	25	27	30	33	37	16	16	17	18	18	22	23	24	25	26
	140-159	16	17	19	21	23	20	22	25	27	30	13	14	14	15	15	18	19	20	21	22
	120-139	13	14	15	17	19	16	18	20	22	25	11	11	12	12	13	15	16	17	18	19
	<120	10	11	12	14	15	13	15	16	18	20	9	9	10	10	11	13	13	14	15	16
65-69	≥180	19	20	22	25	28	25	28	31	34	38	14	15	16	16	17	21	23	24	25	27
	160-179	15	16	18	20	22	20	22	25	27	31	12	12	13	13	14	18	19	20	21	22
	140-159	11	13	14	16	18	16	18	20	22	25	9	10	10	11	12	15	15	16	17	18
	120-139	9	10	11	12	14	12	14	15	17	20	8	8	9	9	10	12	13	13	14	15
	<120	7	8	9	10	11	10	11	12	14	16	6	7	7	7	8	10	10	11	12	12
60-64	≥180	14	16	17	19	22	21	23	26	29	33	11	11	12	13	14	18	19	20	21	23
	160-179	11	12	13	15	17	16	18	20	23	26	9	9	10	10	11	14	15	16	17	19
	140-159	8	9	10	12	13	12	14	15	18	20	7	7	8	8	9	11	12	13	14	15
	120-139	6	7	8	9	10	9	11	12	14	16	5	6	6	7	7	9	10	10	11	12
	<120	5	5	6	7	8	7	8	9	10	12	4	5	5	5	6	7	8	8	9	10
55-59	≥180	11	12	13	15	17	17	19	21	24	28	8	9	9	10	11	15	16	17	18	20
	160-179	8	9	10	11	13	13	14	16	18	21	6	7	7	8	8	11	12	13	14	16
	140-159	6	7	8	9	10	10	11	12	14	16	5	5	6	6	7	9	10	10	11	12
	120-139	5	5	6	6	7	7	8	9	11	12	4	4	4	5	5	7	8	8	9	10
	<120	3	4	4	5	5	5	6	7	8	9	3	3	3	4	4	5	6	6	7	8
50-54	≥180	9	9	10	12	13	14	15	18	20	23	6	7	7	8	8	12	13	14	15	17
	160-179	6	7	8	9	10	10	11	13	15	18	5	5	5	6	6	9	10	11	12	13
	140-159	4	5	6	6	7	7	8	10	11	13	4	4	4	5	5	7	8	8	9	10
	120-139	3	4	4	5	5	5	6	7	8	10	3	3	3	3	4	5	6	6	7	8
	<120	2	3	3	3	4	4	4	5	6	7	2	2	2	3	3	4	4	5	5	6
45-49	≥180	7	7	8	9	10	11	13	14	17	20	5	5	6	6	7	10	11	12	13	14
	160-179	5	5	6	6	8	8	9	10	12	14	4	4	4	5	5	7	8	9	10	11
	140-159	3	4	4	5	5	6	6	7	9	10	3	3	3	3	4	5	6	7	7	8
	120-139	2	3	3	3	4	4	5	5	6	8	2	2	2	2	3	4	4	5	6	6
	<120	2	2	2	2	3	3	3	4	4	5	1	2	2	2	2	3	3	4	4	5
40-44	≥180	5	6	6	7	8	9	10	12	14	17	4	4	4	5	5	8	9	10	11	12
	160-179	4	4	4	5	6	6	7	8	10	12	3	3	3	3	4	6	7	7	8	9
	140-159	2	3	3	3	4	4	5	6	7	8	2	2	2	2	3	4	5	5	6	7
	120-139	2	2	2	2	3	3	3	4	5	6	1	1	2	2	2	3	3	4	4	5
	<120	1	1	1	2	2	2	2	3	3	4	1	1	1	1	1	2	3	3	3	4

Total cholesterol (mmol/l)

Southeast Asia

Risk Level: <5% | 5% to <10% | 10% to <20% | 20% to <30% | ≥30%

People with Diabetes

Age (years)	SBP (mmHg)	Men										Women									
		Non-smoker					Smoker					Non-smoker					Smoker				
		<4	4-4.9	5-5.9	6-6.9	≥ 7	<4	4-4.9	5-5.9	6-6.9	≥ 7	<4	4-4.9	5-5.9	6-6.9	≥ 7	<4	4-4.9	5-5.9	6-6.9	≥ 7
70-74	≥180	32	36	39	43	47	41	45	49	54	59	28	29	30	32	33	38	40	42	44	46
	160-179	27	29	32	36	40	34	37	41	45	50	23	25	26	27	28	33	34	36	38	40
	140-159	22	24	26	29	33	28	31	34	38	42	20	21	22	23	24	28	29	31	32	34
	120-139	17	19	22	24	27	23	25	28	31	35	17	17	18	19	20	23	25	26	27	29
	<120	14	16	17	19	22	18	20	23	25	29	14	15	15	16	17	20	21	22	23	24
65-69	≥180	27	30	33	37	41	36	40	44	49	54	23	24	25	27	28	34	36	38	40	43
	160-179	21	24	26	30	33	29	32	36	40	45	19	20	21	22	24	28	30	32	34	36
	140-159	17	19	21	24	27	23	26	29	33	37	15	16	17	18	19	24	25	27	28	30
	120-139	13	15	17	19	21	19	21	23	26	30	13	13	14	15	16	20	21	22	24	25
	<120	10	12	13	15	17	15	16	19	21	24	10	11	12	12	13	16	17	18	19	21
60-64	≥180	22	25	27	31	35	32	35	40	44	50	19	20	21	22	24	30	32	34	37	40
	160-179	17	19	21	24	28	25	28	32	36	41	15	16	17	18	20	25	26	28	31	33
	140-159	13	15	17	19	22	20	22	25	28	33	12	13	14	15	16	20	21	23	25	27
	120-139	10	11	13	15	17	15	17	19	22	26	10	10	11	12	13	16	17	19	20	22
	<120	8	9	10	11	13	12	13	15	17	20	8	8	9	9	10	13	14	15	16	18
55-59	≥180	18	20	23	26	30	28	31	35	40	46	15	16	17	19	20	27	29	31	34	37
	160-179	14	15	17	20	23	22	24	28	32	37	12	13	14	15	16	21	23	25	27	30
	140-159	10	12	13	15	18	16	19	21	25	29	9	10	11	12	13	17	18	20	22	24
	120-139	8	9	10	11	13	12	14	16	19	22	7	8	8	9	10	13	15	16	17	19
	<120	6	7	7	9	10	9	11	12	14	17	6	6	7	7	8	10	11	13	14	15
50-54	≥180	15	17	19	22	25	25	28	32	36	42	12	13	14	16	17	24	26	28	31	34
	160-179	11	13	14	16	19	19	21	24	28	33	9	10	11	12	13	18	20	22	24	27
	140-159	8	9	10	12	14	14	16	18	21	25	7	8	9	9	10	14	16	17	19	21
	120-139	6	7	8	9	10	10	12	13	16	19	6	6	7	7	8	11	12	13	15	17
	<120	4	5	6	6	8	7	8	10	12	14	4	5	5	5	6	8	9	10	12	13
45-49	≥180	13	14	16	18	21	22	25	28	33	38	10	11	12	13	15	21	23	25	28	32
	160-179	9	10	11	13	16	16	18	21	24	29	8	8	9	10	11	16	18	20	22	25
	140-159	7	7	8	10	11	12	13	15	18	22	6	6	7	7	8	12	13	15	17	19
	120-139	5	5	6	7	8	8	9	11	13	16	4	5	5	6	6	9	10	11	13	15
	<120	3	4	4	5	6	6	7	8	10	12	3	3	4	4	5	7	8	9	10	11
40-44	≥180	11	12	13	15	18	19	22	25	29	35	8	9	10	11	12	18	20	23	26	29
	160-179	7	8	9	11	13	14	16	18	21	26	6	7	7	8	9	14	15	17	19	22
	140-159	5	6	6	8	9	10	11	13	15	19	4	5	5	6	7	10	11	13	15	17
	120-139	4	4	4	5	6	7	8	9	11	14	3	3	4	4	5	7	8	10	11	13
	<120	2	3	3	4	4	5	5	6	8	10	2	3	3	3	4	5	6	7	8	9

Total cholesterol (mmol/l)

Central Asia

Risk Level: <5% | 5% to <10% | 10% to <20% | 20% to <30% | ≥30%

People without Diabetes

Age (years)	SBP (mmHg)	Men										Women									
		Non-smoker					Smoker					Non-smoker					Smoker				
	Total cholesterol (mmol/l)	<4	4-4.9	5-5.9	6-6.9	≥ 7	<4	4-4.9	5-5.9	6-6.9	≥ 7	<4	4-4.9	5-5.9	6-6.9	≥ 7	<4	4-4.9	5-5.9	6-6.9	≥ 7
70-74	≥180	43	50	57	65	73	58	66	73	81	87	33	35	39	43	47	52	56	61	66	71
	160-179	34	40	46	53	61	47	54	62	70	78	26	28	31	34	37	42	46	50	55	59
	140-159	27	31	37	43	50	38	44	51	58	66	20	22	24	26	29	33	37	40	44	48
	120-139	21	24	29	34	40	29	34	40	47	55	16	17	19	21	23	26	29	32	35	39
	<120	16	19	22	26	31	23	27	32	37	44	12	13	15	16	18	21	23	25	27	30
65-69	≥180	31	36	43	51	59	46	54	62	71	80	22	24	27	30	33	40	44	49	55	61
	160-179	24	28	33	40	47	36	42	50	59	68	17	18	20	23	26	31	35	39	44	49
	140-159	18	21	25	30	37	28	33	39	47	55	13	14	16	17	20	24	27	30	34	38
	120-139	13	16	19	23	28	21	25	30	36	44	10	11	12	13	15	19	21	23	26	30
	<120	10	12	14	17	21	16	19	23	27	33	7	8	9	10	11	14	16	18	20	23
60-64	≥180	22	26	31	38	46	36	43	51	61	71	15	16	18	21	24	30	34	39	45	51
	160-179	16	19	23	28	35	27	33	40	48	58	11	12	14	15	18	23	26	30	34	39
	140-159	12	14	17	21	26	20	24	30	37	45	8	9	10	11	13	17	20	22	26	30
	120-139	9	10	12	15	19	15	18	22	27	34	6	7	8	8	10	13	15	17	19	22
	<120	6	7	9	11	14	11	13	16	20	25	5	5	6	6	7	10	11	12	14	16
55-59	≥180	15	18	22	28	35	28	34	41	51	62	10	11	13	14	16	23	26	31	36	42
	160-179	11	13	16	20	25	20	25	31	38	48	7	8	9	10	12	17	19	23	26	31
	140-159	8	9	11	14	18	14	18	22	28	36	5	6	7	8	9	12	14	16	19	23
	120-139	6	7	8	10	13	10	13	16	20	26	4	4	5	5	6	9	10	12	14	17
	<120	4	5	6	7	9	7	9	11	14	18	3	3	3	4	4	6	7	9	10	12
50-54	≥180	11	13	16	20	26	21	26	33	42	52	7	8	9	10	12	17	20	24	28	34
	160-179	8	9	11	14	18	15	18	23	30	39	5	6	6	7	8	12	14	17	20	24
	140-159	5	6	8	10	12	10	13	16	21	28	4	4	4	5	6	9	10	12	14	17
	120-139	4	4	5	7	9	7	9	11	15	19	3	3	3	4	4	6	7	9	10	12
	<120	3	3	4	5	6	5	6	8	10	13	2	2	2	2	3	4	5	6	7	9
45-49	≥180	8	9	11	14	19	16	20	26	34	44	5	6	6	7	8	13	15	18	22	27
	160-179	5	6	8	10	13	11	14	18	23	31	3	4	4	5	6	9	11	13	15	19
	140-159	4	4	5	7	9	8	9	12	16	22	2	3	3	3	4	6	7	9	11	13
	120-139	2	3	3	4	6	5	6	8	11	15	2	2	2	2	3	4	5	6	7	9
	<120	2	2	2	3	4	3	4	5	7	10	1	1	1	2	2	3	3	4	5	6
40-44	≥180	6	7	8	10	14	12	16	20	27	36	4	4	4	5	6	10	12	14	17	21
	160-179	4	4	5	7	9	8	10	13	18	25	2	3	3	3	4	7	8	10	12	15
	140-159	3	3	4	4	6	5	7	9	12	16	2	2	2	2	3	5	5	6	8	10
	120-139	2	2	2	3	4	4	4	6	8	11	1	1	1	2	2	3	4	4	5	7
	<120	1	1	1	2	2	2	3	4	5	7	1	1	1	1	1	2	2	3	4	4

Total cholesterol (mmol/l)

Central Asia

Risk Level: <5% | 5% to <10% | 10% to <20% | 20% to <30% | ≥30%

People with Diabetes

Age (years)	SBP (mmHg)	Men										Women									
		Non-smoker					Smoker					Non-smoker					Smoker				
	Total cholesterol (mmol/l)	<4	4-4.9	5-5.9	6-6.9	≥ 7	<4	4-4.9	5-5.9	6-6.9	≥ 7	<4	4-4.9	5-5.9	6-6.9	≥ 7	<4	4-4.9	5-5.9	6-6.9	≥ 7
70-74	≥180	64	72	80	86	92	80	87	92	96	98	57	61	66	71	76	80	85	88	92	94
	160-179	53	61	69	76	84	69	77	84	90	94	46	51	55	60	65	70	75	79	84	88
	140-159	43	49	57	65	73	58	65	73	81	88	37	41	45	49	54	59	64	68	73	78
	120-139	33	39	46	53	62	47	54	62	70	78	29	32	36	39	43	48	52	57	62	67
	<120	26	31	36	43	50	37	43	51	58	67	23	25	28	31	34	39	42	46	51	56
65-69	≥180	51	59	68	77	85	71	79	87	93	97	43	48	53	58	64	72	77	83	87	92
	160-179	40	47	55	64	73	58	67	76	84	91	34	38	42	47	52	60	66	72	77	83
	140-159	31	37	44	52	61	47	55	64	73	82	26	29	33	37	41	48	54	60	66	72
	120-139	23	28	34	41	49	36	43	51	60	70	20	22	25	28	32	38	43	48	54	60
	<120	17	21	26	31	38	28	33	40	48	57	15	17	19	22	25	30	33	38	42	48
60-64	≥180	39	47	56	65	75	61	71	80	88	94	32	36	41	46	53	63	69	76	82	88
	160-179	30	36	43	52	62	48	58	67	77	86	24	28	31	36	41	50	57	63	70	77
	140-159	22	27	33	40	49	37	45	54	64	75	18	21	24	27	31	39	45	51	58	65
	120-139	16	20	24	30	37	28	34	42	51	61	14	15	18	20	23	30	34	40	45	52
	<120	12	14	18	22	28	21	25	31	39	48	10	11	13	15	17	23	26	30	35	40
55-59	≥180	30	36	44	54	65	52	62	73	83	91	24	27	31	36	42	54	61	69	76	84
	160-179	22	27	33	41	51	40	48	59	70	81	17	20	23	27	32	42	48	55	63	71
	140-159	16	19	24	30	38	29	36	45	56	67	13	15	17	20	23	31	37	43	50	58
	120-139	11	14	17	22	28	21	27	34	42	53	9	11	12	14	17	23	27	32	38	45
	<120	8	10	12	16	20	15	19	24	31	40	7	8	9	10	12	17	20	24	28	34
50-54	≥180	23	28	35	44	55	44	54	65	77	87	17	20	23	28	33	45	53	61	70	79
	160-179	16	20	25	32	41	32	40	50	62	74	13	14	17	20	24	34	40	47	56	65
	140-159	11	14	18	23	29	23	29	37	47	59	9	10	12	14	17	25	30	35	43	51
	120-139	8	10	12	16	21	16	20	27	35	45	6	7	8	10	12	18	21	26	31	38
	<120	5	7	8	11	14	11	14	19	24	32	4	5	6	7	8	13	15	18	23	28
45-49	≥180	18	22	27	35	45	37	46	57	70	83	13	15	18	21	26	38	45	54	63	73
	160-179	12	15	19	24	32	26	33	42	54	68	9	11	12	15	18	28	33	40	49	58
	140-159	8	10	13	17	22	18	23	30	40	52	6	7	9	10	13	20	24	29	36	44
	120-139	6	7	9	11	15	12	16	21	28	37	4	5	6	7	9	14	17	21	26	32
	<120	4	5	6	8	10	8	11	14	19	26	3	3	4	5	6	9	12	14	18	23
40-44	≥180	14	17	21	27	37	31	39	50	63	77	10	11	13	16	20	32	39	47	57	68
	160-179	9	11	14	19	25	21	27	35	47	61	7	8	9	11	14	22	27	34	42	52
	140-159	6	7	9	12	17	14	18	24	33	45	5	5	6	7	9	15	19	24	30	38
	120-139	4	5	6	8	11	9	12	16	22	31	3	4	4	5	6	10	13	16	21	27
	<120	3	3	4	5	7	6	8	11	15	21	2	2	3	3	4	7	9	11	14	19

Total cholesterol (mmol/l)

High-income Asia Pacific

Risk Level: <5% | 5% to <10% | 10% to <20% | 20% to <30% | ≥30%

People without Diabetes

Total cholesterol (mmol/l)

Age (years)	SBP (mmHg)	Men Non-smoker <4	4-4.9	5-5.9	6-6.9	≥ 7	Men Smoker <4	4-4.9	5-5.9	6-6.9	≥ 7	Women Non-smoker <4	4-4.9	5-5.9	6-6.9	≥ 7	Women Smoker <4	4-4.9	5-5.9	6-6.9	≥ 7
70-74	≥180	21	22	24	26	28	25	27	29	32	34	16	17	18	19	21	24	25	27	28	30
	160-179	18	19	21	22	24	21	23	25	27	30	13	14	15	16	17	20	21	22	24	25
	140-159	15	16	17	19	21	18	20	21	23	25	11	12	12	13	14	16	17	18	20	21
	120-139	13	14	15	16	18	15	17	18	20	22	9	10	10	11	11	14	14	15	16	17
	<120	11	12	13	14	15	13	14	15	17	18	8	8	8	9	9	11	12	12	13	14
65-69	≥180	17	18	20	22	24	21	23	25	28	30	12	13	14	15	16	19	21	22	24	26
	160-179	14	15	17	18	20	18	20	21	23	26	10	10	11	12	13	16	17	18	19	21
	140-159	12	13	14	15	17	15	16	18	20	22	8	8	9	10	10	13	14	15	16	17
	120-139	10	11	12	13	14	13	14	15	17	18	6	7	7	8	8	10	11	12	13	14
	<120	8	9	10	11	12	10	11	13	14	15	5	5	6	6	7	8	9	9	10	11
60-64	≥180	14	15	16	18	20	18	20	22	24	27	9	10	10	11	12	15	17	18	20	22
	160-179	11	12	13	15	16	15	17	18	20	22	7	8	8	9	10	12	13	14	16	17
	140-159	9	10	11	12	13	12	14	15	17	19	6	6	6	7	8	10	11	11	13	14
	120-139	7	8	9	10	11	10	11	12	14	15	4	5	5	5	6	8	8	9	10	11
	<120	6	7	7	8	9	8	9	10	11	13	3	4	4	4	5	6	7	7	8	9
55-59	≥180	11	12	13	15	16	16	17	19	21	24	7	7	8	8	9	12	14	15	17	18
	160-179	9	10	11	12	13	13	14	15	17	19	5	6	6	7	7	10	11	12	13	14
	140-159	7	8	9	10	11	10	11	13	14	16	4	4	5	5	6	8	8	9	10	11
	120-139	6	6	7	8	9	8	9	10	11	13	3	3	4	4	4	6	6	7	8	9
	<120	5	5	6	6	7	7	7	8	9	10	2	3	3	3	3	4	5	5	6	7
50-54	≥180	9	10	11	12	14	13	15	16	18	21	5	5	6	6	7	10	11	12	14	16
	160-179	7	8	9	10	11	11	12	13	15	17	4	4	4	5	5	8	8	9	11	12
	140-159	6	6	7	8	9	8	9	10	12	13	3	3	3	4	4	6	6	7	8	9
	120-139	4	5	5	6	7	7	7	8	9	11	2	2	3	3	3	4	5	5	6	7
	<120	3	4	4	5	5	5	6	7	7	8	2	2	2	2	2	3	4	4	5	5
45-49	≥180	7	8	9	10	11	11	13	14	16	18	4	4	4	5	5	8	9	10	11	13
	160-179	6	6	7	8	9	9	10	11	13	14	3	3	3	4	4	6	7	8	9	10
	140-159	4	5	5	6	7	7	8	9	10	11	2	2	2	3	3	4	5	6	6	7
	120-139	3	4	4	5	5	5	6	7	8	9	2	2	2	2	2	3	4	4	5	5
	<120	3	3	3	4	4	4	5	5	6	7	1	1	1	1	2	2	3	3	3	4
40-44	≥180	6	7	7	8	9	10	11	12	14	16	3	3	3	4	4	7	7	8	10	11
	160-179	5	5	6	6	7	7	8	9	11	12	2	2	2	3	3	5	5	6	7	8
	140-159	3	4	4	5	5	6	6	7	8	10	1	2	2	2	2	3	4	4	5	6
	120-139	3	3	3	4	4	4	5	5	6	7	1	1	1	1	2	2	3	3	4	4
	<120	2	2	2	3	3	3	4	4	5	6	1	1	1	1	1	2	2	2	3	3

High-income Asia Pacific

Risk Level: <5% | 5% to <10% | 10% to <20% | 20% to <30% | ≥30%

People with Diabetes

Total cholesterol (mmol/l)

Age (years)	SBP (mmHg)	Men Non-smoker <4	4-4.9	5-5.9	6-6.9	≥ 7	Men Smoker <4	4-4.9	5-5.9	6-6.9	≥ 7	Women Non-smoker <4	4-4.9	5-5.9	6-6.9	≥ 7	Women Smoker <4	4-4.9	5-5.9	6-6.9	≥ 7
70-74	≥180	26	29	31	34	37	32	35	38	41	44	26	27	29	31	33	37	39	42	44	47
	160-179	23	25	27	29	32	28	30	32	35	38	21	23	24	26	27	31	33	35	38	40
	140-159	19	21	23	25	27	24	26	28	30	33	18	19	20	21	23	26	28	30	32	34
	120-139	16	18	19	21	23	20	22	24	26	29	15	16	17	18	19	22	23	25	26	28
	<120	14	15	17	18	20	17	19	20	22	24	12	13	14	14	15	18	19	20	22	23
65-69	≥180	23	25	27	29	32	29	31	34	38	41	20	22	24	25	27	32	35	37	40	44
	160-179	19	21	23	25	27	24	27	29	32	35	17	18	19	21	22	27	29	31	34	36
	140-159	16	17	19	21	23	21	22	25	27	30	13	14	16	17	18	22	24	25	28	30
	120-139	13	15	16	18	19	17	19	21	23	25	11	12	13	14	15	18	19	21	23	25
	<120	11	12	13	15	16	14	16	17	19	21	9	9	10	11	12	14	16	17	18	20
60-64	≥180	19	21	23	26	29	26	28	31	35	38	16	18	19	21	23	28	31	34	37	40
	160-179	16	18	19	21	24	22	24	26	29	32	13	14	15	17	18	23	25	27	30	33
	140-159	13	14	16	18	20	18	20	22	24	27	10	11	12	13	14	18	20	22	24	27
	120-139	11	12	13	15	16	15	16	18	20	23	8	9	10	10	11	15	16	17	19	21
	<120	9	10	11	12	13	12	13	15	17	19	6	7	8	8	9	12	13	14	15	17
55-59	≥180	17	18	20	22	25	23	26	29	32	36	13	14	15	17	19	25	27	30	33	37
	160-179	13	15	16	18	21	19	21	24	26	30	10	11	12	13	15	19	21	24	27	30
	140-159	11	12	13	15	17	16	17	19	22	24	8	8	9	10	11	15	17	19	21	24
	120-139	9	10	11	12	14	13	14	16	18	20	6	7	7	8	9	12	13	15	16	18
	<120	7	8	9	10	11	10	11	13	14	16	5	5	6	6	7	9	10	11	13	14
50-54	≥180	14	16	17	19	22	21	23	26	29	33	10	11	12	14	16	21	24	27	30	34
	160-179	11	12	14	16	18	17	19	21	24	27	8	9	10	11	12	16	18	21	23	27
	140-159	9	10	11	12	14	13	15	17	19	22	6	7	7	8	9	13	14	16	18	21
	120-139	7	8	9	10	11	11	12	14	15	18	4	5	5	6	7	10	11	12	14	16
	<120	6	6	7	8	9	8	10	11	12	14	3	4	4	5	5	7	8	9	11	12
45-49	≥180	12	14	15	17	19	19	21	24	27	31	8	9	10	11	13	19	21	24	27	31
	160-179	10	11	12	13	15	15	17	19	22	25	6	7	8	8	10	14	16	18	21	24
	140-159	7	8	9	10	12	12	13	15	17	20	5	5	6	6	7	10	12	14	16	18
	120-139	6	6	7	8	9	9	10	12	13	16	3	4	4	5	5	8	9	10	12	14
	<120	4	5	6	6	7	7	8	9	11	12	2	3	3	3	4	6	7	8	9	10
40-44	≥180	11	12	13	15	17	17	19	22	25	29	7	7	8	9	11	16	18	21	24	28
	160-179	8	9	10	11	13	13	15	17	19	23	5	5	6	7	8	12	14	16	18	22
	140-159	6	7	8	9	10	10	11	13	15	18	3	4	4	5	6	9	10	12	14	16
	120-139	5	5	6	7	8	8	9	10	12	14	3	3	3	4	4	6	7	9	10	12
	<120	4	4	4	5	6	6	7	8	9	11	2	2	2	3	3	5	5	6	7	9

Western Europe

Risk Level: <5% | 5% to <10% | 10% to <20% | 20% to <30% | ≥30%

People without Diabetes

Age (years)	SBP (mmHg)	Men Non-smoker					Men Smoker					Women Non-smoker					Women Smoker				
		<4	4-4.9	5-5.9	6-6.9	≥ 7	<4	4-4.9	5-5.9	6-6.9	≥ 7	<4	4-4.9	5-5.9	6-6.9	≥ 7	<4	4-4.9	5-5.9	6-6.9	≥ 7
70-74	≥180	27	29	33	36	40	34	37	41	45	49	20	21	23	25	27	31	34	36	39	42
	160-179	22	24	27	30	33	28	31	34	38	42	16	17	18	20	21	25	27	29	32	34
	140-159	18	20	22	25	28	23	26	29	32	35	12	13	14	15	17	20	22	23	25	27
	120-139	15	16	18	20	23	19	21	24	26	29	10	10	11	12	13	16	17	18	20	22
	<120	12	13	15	17	19	15	17	19	22	24	8	8	9	10	10	12	13	15	16	17
65-69	≥180	21	23	26	29	32	28	31	35	39	43	14	15	16	18	19	24	26	29	32	35
	160-179	16	18	21	23	26	23	25	28	32	36	10	11	12	14	15	19	21	23	25	28
	140-159	13	15	17	19	21	18	20	23	26	30	8	9	9	10	11	15	16	18	19	22
	120-139	10	12	13	15	17	14	16	19	21	24	6	7	7	8	9	11	12	14	15	17
	<120	8	9	11	12	14	12	13	15	17	19	5	5	5	6	7	9	9	10	12	13
60-64	≥180	16	18	20	23	26	23	26	29	33	38	9	10	11	12	14	19	21	23	26	29
	160-179	12	14	16	18	21	18	21	23	27	31	7	8	8	9	10	14	16	17	20	22
	140-159	9	11	12	14	16	14	16	19	21	25	5	6	6	7	8	11	12	13	15	17
	120-139	7	8	10	11	13	11	13	15	17	20	4	4	5	5	6	8	9	10	11	13
	<120	6	6	7	9	10	9	10	11	13	16	3	3	3	4	4	6	7	7	8	10
55-59	≥180	12	13	15	18	21	19	21	25	28	33	6	7	8	9	10	14	16	18	21	24
	160-179	9	10	12	14	16	14	17	19	22	26	5	5	6	6	7	10	12	13	15	18
	140-159	7	8	9	11	12	11	13	15	17	20	3	4	4	5	5	8	9	10	11	13
	120-139	5	6	7	8	10	8	10	11	13	16	2	3	3	3	4	5	6	7	8	10
	<120	4	4	5	6	7	6	7	9	10	12	2	2	2	2	3	4	5	5	6	7
50-54	≥180	9	10	12	14	16	15	18	20	24	28	4	5	5	6	7	11	12	14	16	19
	160-179	7	8	9	10	12	11	13	16	18	22	3	3	4	4	5	8	9	10	12	14
	140-159	5	6	7	8	9	8	10	12	14	17	2	2	3	3	4	5	6	7	8	10
	120-139	4	4	5	6	7	6	7	9	11	13	1	2	2	2	2	4	4	5	6	7
	<120	3	3	4	4	5	5	6	7	8	10	1	1	1	1	2	3	3	4	4	5
45-49	≥180	7	8	9	11	13	12	14	17	20	24	3	3	4	4	5	8	9	11	13	16
	160-179	5	6	7	8	10	9	11	13	15	18	2	2	3	3	3	6	7	8	9	11
	140-159	3	4	5	6	7	7	8	9	11	14	1	2	2	2	2	4	5	5	6	8
	120-139	2	3	4	4	5	5	6	7	8	10	1	1	1	1	2	3	3	4	4	5
	<120	2	2	3	3	4	3	4	5	6	8	1	1	1	1	1	2	2	3	3	4
40-44	≥180	5	6	7	8	10	10	12	14	17	21	2	2	3	3	4	6	7	9	10	12
	160-179	4	4	5	6	7	7	8	10	12	15	1	2	2	2	2	4	5	6	7	9
	140-159	2	3	4	4	5	5	6	7	9	11	1	1	1	1	2	3	3	4	5	6
	120-139	2	2	3	3	4	4	4	5	7	8	1	1	1	1	1	2	2	3	3	4
	<120	1	1	2	2	3	3	3	4	5	6	0	0	0	1	1	1	1	2	2	3

Total cholesterol (mmol/l)

Western Europe

Risk Level: <5% | 5% to <10% | 10% to <20% | 20% to <30% | ≥30%

People with Diabetes

Age (years)	SBP (mmHg)	Men Non-smoker					Men Smoker					Women Non-smoker					Women Smoker				
		<4	4-4.9	5-5.9	6-6.9	≥ 7	<4	4-4.9	5-5.9	6-6.9	≥ 7	<4	4-4.9	5-5.9	6-6.9	≥ 7	<4	4-4.9	5-5.9	6-6.9	≥ 7
70-74	≥180	36	40	44	48	53	45	50	54	59	64	34	37	40	43	46	52	55	59	63	66
	160-179	30	34	37	41	45	38	42	46	51	56	28	30	32	35	38	43	46	49	53	57
	140-159	25	28	31	34	38	32	35	39	43	48	22	24	26	28	30	35	38	41	44	47
	120-139	21	23	26	29	32	27	30	33	37	41	18	19	21	22	24	28	31	33	36	39
	<120	17	19	21	24	27	22	24	27	31	34	14	15	16	18	19	23	24	26	29	31
65-69	≥180	30	33	37	42	46	40	44	49	54	60	26	29	31	34	38	45	49	53	57	62
	160-179	24	27	31	34	39	33	37	41	46	51	20	22	25	27	30	36	39	43	47	51
	140-159	20	22	25	28	32	27	30	34	38	43	16	17	19	21	23	29	31	34	38	42
	120-139	16	18	20	23	26	22	25	28	32	36	12	13	15	16	18	22	25	27	30	33
	<120	12	14	16	19	21	18	20	23	26	30	9	10	11	13	14	17	19	21	24	26
60-64	≥180	25	28	31	36	40	35	40	44	50	56	20	22	24	27	30	38	42	47	52	57
	160-179	19	22	25	29	33	28	32	36	41	47	15	17	18	21	23	30	33	37	41	46
	140-159	15	17	20	23	26	23	26	29	34	39	11	12	14	16	18	23	26	29	33	37
	120-139	12	14	16	18	21	18	21	24	27	31	8	9	10	12	13	18	20	22	25	29
	<120	9	11	12	14	17	14	16	19	22	25	6	7	8	9	10	13	15	17	19	22
55-59	≥180	20	23	26	30	35	31	35	40	46	52	15	17	19	21	24	33	37	41	46	52
	160-179	15	18	20	24	28	24	28	32	37	42	11	12	14	16	18	25	28	32	36	41
	140-159	12	14	16	18	22	19	22	25	29	34	8	9	10	12	13	18	21	24	28	32
	120-139	9	10	12	14	17	15	17	20	23	27	6	6	7	8	10	14	16	18	21	24
	<120	7	8	9	11	13	11	13	15	18	22	4	5	5	6	7	10	12	13	16	18
50-54	≥180	17	19	22	25	30	27	31	36	42	48	11	13	14	16	19	28	32	36	42	48
	160-179	12	14	17	19	23	21	24	28	33	39	8	9	10	12	14	20	23	27	32	37
	140-159	9	11	13	15	18	16	18	22	26	30	6	6	7	8	10	15	17	20	24	28
	120-139	7	8	9	11	14	12	14	17	20	24	4	4	5	6	7	11	12	15	17	21
	<120	5	6	7	8	10	9	11	13	15	18	3	3	4	4	5	8	9	11	13	15
45-49	≥180	13	16	18	21	25	24	28	32	38	44	8	9	11	13	15	23	27	32	37	43
	160-179	10	11	13	16	19	18	21	25	29	35	6	7	8	9	11	17	19	23	27	33
	140-159	7	8	10	12	14	13	15	18	22	27	4	4	5	6	7	12	14	17	20	24
	120-139	5	6	7	9	11	10	11	14	17	21	3	3	4	4	5	8	10	12	14	17
	<120	4	4	5	6	8	7	8	10	13	16	2	2	2	3	4	6	7	8	10	12
40-44	≥180	11	13	15	18	21	21	24	29	34	41	6	7	8	10	12	20	23	27	33	39
	160-179	8	9	11	13	16	15	18	21	26	31	4	5	6	7	8	13	16	19	24	29
	140-159	5	6	8	9	12	11	13	16	19	24	3	3	4	5	6	9	11	14	17	21
	120-139	4	5	6	7	9	8	9	12	14	18	2	2	3	3	4	6	8	9	12	15
	<120	3	3	4	5	6	6	7	8	11	13	1	1	2	2	3	4	5	6	8	10

Total cholesterol (mmol/l)

Central Europe

Risk Level: <5% | 5% to <10% | 10% to <20% | 20% to <30% | ≥30%

People without Diabetes

Age (years)	SBP (mmHg)	Men Non-smoker <4	4-4.9	5-5.9	6-6.9	≥ 7	Men Smoker <4	4-4.9	5-5.9	6-6.9	≥ 7	Women Non-smoker <4	4-4.9	5-5.9	6-6.9	≥ 7	Women Smoker <4	4-4.9	5-5.9	6-6.9	≥ 7
70-74	≥180	34	37	40	44	49	43	47	51	55	60	29	30	32	35	37	45	48	51	54	58
	160-179	27	30	33	37	40	35	38	42	47	51	22	24	26	27	30	36	38	41	44	47
	140-159	22	24	27	30	33	28	31	35	38	43	17	19	20	21	23	29	31	33	35	38
	120-139	17	19	21	24	27	23	25	28	31	35	14	14	15	17	18	22	24	26	28	30
	<120	14	15	17	19	22	18	20	23	25	29	10	11	12	13	14	17	19	20	22	24
65-69	≥180	26	28	32	35	39	35	39	43	48	53	19	21	23	25	27	35	38	41	44	49
	160-179	20	22	25	28	32	28	31	35	39	44	15	16	17	19	21	27	29	32	35	38
	140-159	15	17	19	22	25	22	24	28	31	35	11	12	13	14	16	21	22	25	27	30
	120-139	12	13	15	17	20	17	19	22	25	28	8	9	10	11	12	16	17	19	21	23
	<120	9	10	12	13	15	13	15	17	19	22	6	7	7	8	9	12	13	14	15	17
60-64	≥180	19	22	24	27	31	29	32	36	41	46	13	14	16	17	19	27	29	32	36	40
	160-179	15	16	18	21	24	22	25	28	32	37	9	10	11	13	14	20	22	24	27	31
	140-159	11	12	14	16	19	17	19	22	25	29	7	7	8	9	10	15	16	18	20	23
	120-139	8	9	11	12	14	13	14	17	19	22	5	5	6	7	7	11	12	13	15	17
	<120	6	7	8	9	11	9	11	13	15	17	4	4	4	5	5	8	9	10	11	12
55-59	≥180	14	16	18	21	24	23	26	30	34	39	9	10	11	12	13	20	23	25	29	33
	160-179	11	12	14	16	18	17	20	23	26	31	6	7	8	8	10	15	16	18	21	24
	140-159	8	9	10	12	14	13	15	17	20	23	4	5	5	6	7	10	12	13	15	17
	120-139	5	6	7	9	10	9	11	13	15	18	3	3	4	4	5	7	8	9	11	12
	<120	4	5	5	6	7	7	8	9	11	13	2	2	3	3	3	5	6	7	8	9
50-54	≥180	11	12	14	16	19	19	21	24	29	34	6	7	7	8	9	15	17	20	23	27
	160-179	8	9	10	12	14	13	15	18	21	25	4	4	5	6	6	11	12	14	16	19
	140-159	5	6	7	8	10	10	11	13	16	19	3	3	3	4	4	7	8	10	11	13
	120-139	4	4	5	6	7	7	8	9	11	14	2	2	2	3	3	5	6	7	8	9
	<120	3	3	4	4	5	5	6	7	8	10	1	1	1	2	2	3	4	4	5	6
45-49	≥180	8	9	10	12	14	15	17	20	24	28	4	5	5	6	7	12	13	15	18	21
	160-179	5	6	7	9	10	10	12	14	17	21	3	3	3	4	4	8	9	10	12	15
	140-159	4	4	5	6	7	7	8	10	12	15	2	2	2	2	3	5	6	7	8	10
	120-139	3	3	3	4	5	5	6	7	9	11	1	1	1	2	2	3	4	5	5	7
	<120	2	2	2	3	4	3	4	5	6	8	1	1	1	1	1	2	3	3	4	4
40-44	≥180	6	7	8	9	11	12	14	16	19	24	3	3	3	4	5	9	10	12	14	17
	160-179	4	4	5	6	8	8	9	11	14	17	2	2	2	3	3	6	6	8	9	11
	140-159	3	3	3	4	5	5	6	8	10	12	1	1	1	2	2	3	4	5	6	7
	120-139	2	2	2	3	4	4	4	5	7	8	1	1	1	1	1	2	3	3	4	5
	<120	1	1	2	2	2	2	3	4	5	6	0	0	1	1	1	1	2	2	3	3

Total cholesterol (mmol/l)

Central Europe

Risk Level: <5% | 5% to <10% | 10% to <20% | 20% to <30% | ≥30%

People with Diabetes

Age (years)	SBP (mmHg)	Men Non-smoker <4	4-4.9	5-5.9	6-6.9	≥ 7	Men Smoker <4	4-4.9	5-5.9	6-6.9	≥ 7	Women Non-smoker <4	4-4.9	5-5.9	6-6.9	≥ 7	Women Smoker <4	4-4.9	5-5.9	6-6.9	≥ 7
70-74	≥180	45	50	54	59	64	56	61	66	71	76	49	52	55	59	63	70	74	77	81	84
	160-179	37	41	45	50	55	47	52	57	62	67	40	42	45	48	52	60	63	67	71	74
	140-159	30	34	38	42	46	39	43	48	53	58	32	34	36	39	42	49	53	56	60	64
	120-139	25	27	31	34	38	32	36	40	44	49	25	27	29	31	34	40	43	46	49	53
	<120	20	22	25	28	31	26	29	33	36	41	19	21	22	24	26	32	34	37	40	43
65-69	≥180	38	42	46	51	57	50	55	60	66	72	38	41	44	48	52	62	66	70	75	79
	160-179	30	33	37	42	47	41	46	50	56	62	29	32	35	38	41	51	55	59	64	68
	140-159	24	26	30	34	38	33	37	41	46	52	22	24	27	29	32	40	44	48	52	57
	120-139	18	21	24	27	31	26	29	33	38	43	17	18	20	22	25	31	34	38	42	46
	<120	14	16	18	21	24	20	23	27	30	35	13	14	15	17	19	24	27	29	32	36
60-64	≥180	31	34	39	44	49	45	49	55	61	67	28	31	34	38	42	54	59	64	69	75
	160-179	24	27	30	35	39	35	40	45	50	56	21	23	26	29	32	42	47	51	57	62
	140-159	18	21	23	27	31	27	31	35	40	46	16	17	19	22	24	32	36	40	45	50
	120-139	14	16	18	21	24	21	24	28	32	37	11	13	14	16	18	24	27	31	35	39
	<120	10	12	14	16	19	16	19	21	25	29	8	9	10	12	13	18	20	23	26	30
55-59	≥180	25	28	32	37	42	39	44	50	56	63	21	24	26	30	34	46	51	57	63	70
	160-179	19	21	24	28	33	30	34	39	45	51	15	17	19	22	25	35	39	44	50	56
	140-159	14	16	18	21	25	23	26	30	35	41	11	12	14	16	18	26	29	33	38	44
	120-139	10	12	14	16	19	17	20	23	27	32	8	9	10	11	13	19	21	25	29	33
	<120	7	9	10	12	14	13	15	17	21	25	5	6	7	8	9	13	15	18	21	24
50-54	≥180	21	23	27	31	36	35	39	44	51	58	16	18	20	23	27	40	45	50	57	64
	160-179	15	17	20	23	27	26	29	34	40	46	11	12	14	16	19	29	33	38	44	50
	140-159	11	12	14	17	20	19	22	26	30	36	7	8	10	11	13	20	24	28	32	38
	120-139	8	9	10	12	15	14	16	19	23	27	5	6	7	8	9	14	17	20	23	28
	<120	5	6	7	9	11	10	12	14	17	20	3	4	4	5	6	10	12	14	17	20
45-49	≥180	17	19	22	25	30	31	35	40	46	54	12	13	15	18	21	33	38	44	51	59
	160-179	12	13	16	18	22	22	25	29	35	42	8	9	10	12	14	23	27	32	38	45
	140-159	8	9	11	13	16	15	18	21	26	31	5	6	7	8	10	16	19	23	27	33
	120-139	6	6	8	9	11	11	13	15	19	23	3	4	4	5	6	11	13	16	19	23
	<120	4	4	5	7	8	8	9	11	14	17	2	3	3	4	4	7	9	11	13	16
40-44	≥180	14	16	18	21	25	27	31	35	42	49	9	10	12	14	16	28	33	39	46	54
	160-179	9	11	12	15	18	19	22	25	31	37	6	6	8	9	11	19	22	27	33	40
	140-159	6	7	8	10	13	13	15	18	22	27	4	4	5	6	7	12	15	18	23	28
	120-139	4	5	6	7	9	9	10	13	16	20	2	3	3	4	5	8	10	12	15	19
	<120	3	3	4	5	6	6	7	9	11	14	1	2	2	2	3	5	6	8	10	13

Total cholesterol (mmol/l)

Eastern Europe

Risk Level: <5% | 5% to <10% | 10% to <20% | 20% to <30% | ≥30%

People without Diabetes

Age (years)	SBP (mmHg)	Men Non-smoker <4	4-4.9	5-5.9	6-6.9	≥ 7	Men Smoker <4	4-4.9	5-5.9	6-6.9	≥ 7	Women Non-smoker <4	4-4.9	5-5.9	6-6.9	≥ 7	Women Smoker <4	4-4.9	5-5.9	6-6.9	≥ 7
70-74	≥180	36	39	41	45	48	43	46	49	53	56	33	34	36	39	41	47	49	52	55	58
	160-179	31	33	36	38	41	37	40	43	46	50	27	28	30	32	34	39	42	44	47	50
	140-159	26	28	30	33	36	32	34	37	40	43	22	23	25	26	28	33	35	37	39	42
	120-139	22	24	26	28	31	27	29	32	34	37	18	19	20	22	23	27	29	31	33	35
	<120	18	20	22	24	26	23	25	27	29	32	15	16	17	18	19	22	24	25	27	29
65-69	≥180	29	32	34	37	41	37	40	43	47	51	24	25	27	29	32	38	41	44	47	50
	160-179	24	26	29	31	34	31	34	37	40	44	19	21	22	24	26	31	34	36	39	42
	140-159	20	22	24	26	29	26	28	31	34	37	15	16	18	19	21	25	27	29	32	34
	120-139	16	18	20	22	24	22	24	26	29	32	12	13	14	15	16	20	22	24	26	28
	<120	13	15	16	18	20	18	20	22	24	27	10	10	11	12	13	16	18	19	21	22
60-64	≥180	23	26	28	31	34	32	35	38	42	46	17	19	20	22	24	31	34	36	40	43
	160-179	19	21	23	25	28	26	29	32	35	38	14	15	16	17	19	25	27	29	32	35
	140-159	15	17	19	21	23	21	23	26	29	32	10	11	12	14	15	19	21	23	25	28
	120-139	12	14	15	17	19	17	19	21	24	27	8	9	10	10	12	15	17	18	20	22
	<120	10	11	12	14	16	14	16	17	20	22	6	7	7	8	9	12	13	14	16	17
55-59	≥180	19	21	23	25	28	27	30	33	37	41	13	14	15	17	18	25	27	30	33	37
	160-179	15	16	18	20	23	22	24	27	30	34	10	10	11	13	14	19	21	23	26	29
	140-159	12	13	15	16	19	17	19	22	24	28	7	8	9	10	11	15	16	18	20	23
	120-139	9	10	12	13	15	14	15	17	20	22	5	6	6	7	8	11	12	14	16	18
	<120	7	8	9	10	12	11	12	14	16	18	4	4	5	5	6	8	9	11	12	13
50-54	≥180	15	16	18	21	24	23	25	28	32	36	9	10	11	12	14	20	22	25	28	31
	160-179	11	13	14	16	19	18	20	23	26	29	7	7	8	9	10	15	17	19	21	24
	140-159	9	10	11	13	15	14	16	18	20	23	5	5	6	7	8	11	12	14	16	18
	120-139	7	8	9	10	12	11	12	14	16	19	4	4	4	5	6	8	9	10	12	14
	<120	5	6	7	8	9	8	10	11	13	15	3	3	3	4	4	6	7	8	9	10
45-49	≥180	12	13	15	17	19	19	22	24	28	32	7	7	8	9	10	16	18	20	23	26
	160-179	9	10	11	13	15	15	17	19	22	25	5	5	6	7	7	11	13	15	17	20
	140-159	7	8	9	10	12	11	13	15	17	20	3	4	4	5	5	8	9	11	13	15
	120-139	5	6	7	8	9	9	10	11	13	16	2	3	3	3	4	6	7	8	9	11
	<120	4	4	5	6	7	6	7	9	10	12	2	2	2	2	3	4	5	6	7	8
40-44	≥180	9	10	12	14	16	16	18	21	24	28	5	5	6	7	8	13	14	16	19	22
	160-179	7	8	9	10	12	12	14	16	19	22	3	4	4	5	5	9	10	12	14	16
	140-159	5	6	7	8	9	9	10	12	14	17	2	3	3	3	4	6	7	8	10	12
	120-139	4	4	5	6	7	7	8	9	11	13	2	2	2	2	3	4	5	6	7	8
	<120	3	3	4	4	5	5	6	7	8	10	1	1	1	2	2	3	4	4	5	6

Total cholesterol (mmol/l)

Eastern Europe

Risk Level: <5% | 5% to <10% | 10% to <20% | 20% to <30% | ≥30%

People with Diabetes

Age (years)	SBP (mmHg)	Men Non-smoker <4	4-4.9	5-5.9	6-6.9	≥ 7	Men Smoker <4	4-4.9	5-5.9	6-6.9	≥ 7	Women Non-smoker <4	4-4.9	5-5.9	6-6.9	≥ 7	Women Smoker <4	4-4.9	5-5.9	6-6.9	≥ 7
70-74	≥180	45	49	52	56	59	54	57	61	65	68	50	53	56	59	62	68	71	74	77	80
	160-179	39	42	45	49	52	47	50	54	58	61	42	45	48	50	53	59	62	65	68	72
	140-159	33	36	39	42	46	41	44	47	51	54	36	38	40	43	45	51	54	57	60	63
	120-139	28	31	34	37	40	35	38	41	44	48	29	31	33	36	38	43	46	48	51	54
	<120	24	26	29	31	34	30	32	35	38	42	24	26	27	29	31	36	38	41	43	46
65-69	≥180	39	42	46	50	54	49	53	57	61	65	41	43	46	50	53	61	64	68	72	76
	160-179	33	36	39	43	46	42	45	49	53	57	33	36	38	41	45	52	55	59	63	67
	140-159	28	30	33	36	40	36	39	42	46	50	27	29	31	34	37	43	46	50	54	57
	120-139	23	25	28	31	34	30	33	36	39	43	22	23	25	27	30	36	38	41	45	48
	<120	19	21	23	26	28	25	27	30	33	37	17	19	20	22	24	29	31	34	37	40
60-64	≥180	34	37	40	44	48	45	48	52	57	61	32	35	38	41	45	54	58	63	67	72
	160-179	28	30	33	37	41	37	41	45	49	53	26	28	31	33	37	45	49	53	57	62
	140-159	23	25	28	31	34	31	34	38	41	46	20	22	24	27	29	37	40	44	48	52
	120-139	18	20	23	25	28	25	28	31	35	39	16	17	19	21	23	29	32	35	39	43
	<120	15	17	19	21	23	21	23	26	29	32	12	14	15	16	18	23	26	28	31	35
55-59	≥180	29	32	35	39	43	41	44	48	53	58	26	28	31	34	38	48	52	57	62	68
	160-179	23	26	28	32	35	33	37	40	45	49	20	22	24	27	30	39	43	47	52	57
	140-159	18	21	23	26	29	27	30	33	37	42	15	17	19	21	23	31	34	38	42	47
	120-139	15	16	18	21	24	22	24	27	31	35	12	13	14	16	18	24	27	30	33	38
	<120	12	13	15	17	19	17	19	22	25	28	9	10	11	12	14	18	21	23	26	30
50-54	≥180	25	27	30	34	38	37	40	45	49	55	20	22	25	28	31	42	47	52	57	63
	160-179	19	21	24	27	31	29	33	36	41	46	15	17	19	21	24	33	37	41	47	52
	140-159	15	17	19	22	25	23	26	29	33	38	11	13	14	16	18	25	29	32	37	42
	120-139	12	13	15	17	20	18	21	24	27	31	8	9	11	12	14	19	22	25	29	33
	<120	9	10	12	13	16	14	16	19	22	25	6	7	8	9	10	14	17	19	22	25
45-49	≥180	21	23	26	29	33	33	37	41	46	51	16	18	20	23	26	37	41	47	53	59
	160-179	16	18	20	23	26	26	29	33	37	42	12	13	15	17	19	28	32	36	42	48
	140-159	12	14	16	18	21	20	23	26	30	34	8	9	11	12	14	21	24	28	32	37
	120-139	9	10	12	14	16	15	18	20	24	27	6	7	8	9	10	15	18	21	24	29
	<120	7	8	9	11	13	12	14	16	19	22	4	5	6	6	8	11	13	15	18	22
40-44	≥180	18	20	22	25	29	30	33	37	42	48	13	14	16	18	21	32	37	42	48	55
	160-179	13	15	17	19	23	23	26	29	34	39	9	10	11	13	15	24	27	32	37	43
	140-159	10	11	13	15	17	17	20	23	26	31	6	7	8	9	11	17	20	24	28	33
	120-139	7	8	10	11	13	13	15	17	21	24	4	5	6	7	8	12	15	17	21	25
	<120	5	6	7	9	10	10	11	13	16	19	3	3	4	5	6	9	10	13	15	18

Total cholesterol (mmol/l)

Oceania

Risk Level: <5% | 5% to <10% | 10% to <20% | 20% to <30% | ≥30%

People without Diabetes

Age (years)	SBP (mmHg)	Men Non-smoker <4	4-4.9	5-5.9	6-6.9	≥ 7	Men Smoker <4	4-4.9	5-5.9	6-6.9	≥ 7	Women Non-smoker <4	4-4.9	5-5.9	6-6.9	≥ 7	Women Smoker <4	4-4.9	5-5.9	6-6.9	≥ 7
70-74	≥180	24	26	28	29	31	29	31	33	35	38	20	20	21	22	22	26	27	28	29	30
	160-179	20	22	23	25	27	25	26	28	30	32	17	17	18	18	19	23	23	24	25	26
	140-159	17	18	19	21	22	21	22	24	26	27	14	15	15	16	16	19	20	21	21	22
	120-139	14	15	16	17	19	17	19	20	21	23	12	13	13	13	14	17	17	18	18	19
	<120	12	13	14	15	16	14	16	17	18	19	10	11	11	11	12	14	15	15	16	16
65-69	≥180	20	21	23	24	26	25	27	29	31	33	15	16	17	17	18	22	23	24	25	27
	160-179	16	17	19	20	22	21	22	24	26	28	13	13	14	15	15	19	20	20	21	22
	140-159	13	14	15	16	18	17	18	20	21	23	11	11	12	12	13	16	16	17	18	19
	120-139	11	11	12	13	15	14	15	16	18	19	9	9	10	10	11	13	14	14	15	16
	<120	9	9	10	11	12	11	12	13	14	16	7	8	8	8	9	11	11	12	12	13
60-64	≥180	16	17	18	20	22	21	23	25	27	29	12	13	13	14	15	19	20	21	22	23
	160-179	13	14	15	16	17	17	19	20	22	24	10	10	11	11	12	15	16	17	18	19
	140-159	10	11	12	13	14	14	15	16	18	19	8	8	9	9	10	13	13	14	15	16
	120-139	8	9	9	10	11	11	12	13	14	16	7	7	7	8	8	10	11	11	12	13
	<120	6	7	7	8	9	9	10	10	11	13	5	6	6	6	6	8	9	9	10	11
55-59	≥180	13	14	15	16	18	18	20	21	23	26	9	10	11	11	12	16	17	18	19	20
	160-179	10	11	12	13	14	14	16	17	19	20	8	8	8	9	9	13	13	14	15	16
	140-159	8	8	9	10	11	11	12	13	15	16	6	6	7	7	8	10	11	12	12	13
	120-139	6	6	7	8	9	9	10	10	12	13	5	5	5	6	6	8	9	9	10	11
	<120	5	5	6	6	7	7	7	8	9	10	4	4	4	4	5	6	7	7	8	8
50-54	≥180	10	11	12	13	14	16	17	18	20	22	7	8	8	9	10	13	14	15	16	18
	160-179	8	8	9	10	11	12	13	14	16	17	6	6	7	7	7	10	11	12	13	14
	140-159	6	6	7	8	9	9	10	11	12	14	4	5	5	5	6	8	9	9	10	11
	120-139	4	5	5	6	7	7	8	8	9	11	3	4	4	4	5	6	7	7	8	9
	<120	3	4	4	5	5	5	6	6	7	8	3	3	3	3	4	5	5	6	6	7
45-49	≥180	8	9	10	11	12	13	14	16	17	19	6	6	7	7	8	11	12	13	14	15
	160-179	6	7	7	8	9	10	11	12	13	15	4	5	5	5	6	9	9	10	11	12
	140-159	5	5	5	6	7	7	8	9	10	11	3	4	4	4	4	7	7	8	8	9
	120-139	3	4	4	4	5	5	6	7	8	9	3	3	3	3	3	5	5	6	6	7
	<120	2	3	3	3	4	4	5	5	6	7	2	2	2	2	3	4	4	4	5	5
40-44	≥180	7	7	8	9	10	11	12	13	15	17	5	5	5	6	6	9	10	11	12	13
	160-179	5	5	6	6	7	8	9	10	11	13	3	4	4	4	5	7	8	8	9	10
	140-159	3	4	4	5	5	6	7	7	8	9	2	3	3	3	3	5	6	6	7	8
	120-139	2	3	3	3	4	4	5	5	6	7	2	2	2	2	3	4	4	5	5	6
	<120	2	2	2	3	3	3	3	4	5	5	1	1	2	2	2	3	3	3	4	4

Total cholesterol (mmol/l)

Oceania

Risk Level: <5% | 5% to <10% | 10% to <20% | 20% to <30% | ≥30%

People with Diabetes

Age (years)	SBP (mmHg)	Men Non-smoker <4	4-4.9	5-5.9	6-6.9	≥ 7	Men Smoker <4	4-4.9	5-5.9	6-6.9	≥ 7	Women Non-smoker <4	4-4.9	5-5.9	6-6.9	≥ 7	Women Smoker <4	4-4.9	5-5.9	6-6.9	≥ 7
70-74	≥180	30	33	35	37	40	37	39	42	44	47	28	29	30	31	32	37	38	40	41	43
	160-179	26	28	30	32	34	31	33	36	38	41	24	25	26	27	28	32	33	34	36	37
	140-159	22	23	25	27	29	26	28	30	33	35	21	21	22	23	24	28	29	30	31	32
	120-139	18	19	21	23	24	22	24	26	28	30	18	18	19	20	20	24	25	26	27	28
	<120	15	16	18	19	21	19	20	22	23	25	15	16	16	17	17	20	21	22	23	24
65-69	≥180	26	28	30	33	35	33	36	38	41	44	24	24	26	27	28	33	35	36	38	40
	160-179	22	23	25	27	29	28	30	32	35	37	20	21	22	23	24	28	30	31	33	34
	140-159	18	19	21	23	25	23	25	27	29	32	17	17	18	19	20	24	25	26	28	29
	120-139	15	16	17	19	20	19	21	22	24	26	14	14	15	16	17	20	21	22	23	25
	<120	12	13	14	15	17	16	17	18	20	22	12	12	13	13	14	17	18	19	20	21
60-64	≥180	23	24	26	28	31	30	33	35	38	41	20	21	22	23	24	30	32	33	35	37
	160-179	18	20	21	23	25	25	27	29	31	34	16	17	18	19	20	25	26	28	30	31
	140-159	15	16	17	19	21	20	22	24	26	28	13	14	15	16	16	21	22	23	25	26
	120-139	12	13	14	15	17	16	18	19	21	23	11	11	12	13	14	17	18	19	20	22
	<120	9	10	11	12	14	13	14	16	17	19	9	9	10	10	11	14	15	16	17	18
55-59	≥180	20	21	23	25	27	28	30	32	35	38	16	17	18	20	21	27	29	31	33	35
	160-179	15	17	18	20	22	22	24	26	28	31	13	14	15	16	17	22	23	25	27	29
	140-159	12	13	14	16	17	17	19	21	23	25	11	11	12	13	14	18	19	20	22	24
	120-139	9	10	11	12	14	14	15	17	18	20	8	9	10	10	11	14	15	17	18	19
	<120	7	8	9	10	11	11	12	13	15	16	7	7	8	8	9	12	12	13	14	16
50-54	≥180	17	18	20	22	24	25	27	29	32	35	14	15	16	17	18	24	26	28	30	33
	160-179	13	14	15	17	19	20	21	23	26	28	11	11	12	13	14	19	21	22	24	26
	140-159	10	11	12	13	15	15	17	18	20	23	8	9	10	10	11	15	17	18	19	21
	120-139	8	8	9	10	11	12	13	14	16	18	7	7	8	8	9	12	13	14	15	17
	<120	6	6	7	8	9	9	10	11	12	14	5	5	6	6	7	9	10	11	12	13
45-49	≥180	15	16	17	19	21	23	25	27	30	33	12	12	13	14	16	22	24	26	28	30
	160-179	11	12	13	14	16	17	19	21	23	26	9	9	10	11	12	17	18	20	22	24
	140-159	8	9	10	11	12	13	14	16	18	20	7	7	8	8	9	13	14	16	17	19
	120-139	6	7	7	8	9	10	11	12	14	16	5	5	6	6	7	10	11	12	13	15
	<120	4	5	5	6	7	7	8	9	10	12	4	4	5	5	5	8	9	9	10	12
40-44	≥180	13	14	15	16	18	21	23	25	27	30	10	10	11	12	13	20	21	23	26	28
	160-179	9	10	11	12	14	15	17	19	21	23	7	8	8	9	10	15	16	18	20	22
	140-159	7	7	8	9	10	11	12	14	16	18	5	6	6	7	8	11	12	14	15	17
	120-139	5	5	6	7	8	8	9	10	12	14	4	4	5	5	6	8	9	10	12	13
	<120	4	4	4	5	6	6	7	8	9	10	3	3	3	4	4	6	7	8	9	10

Total cholesterol (mmol/l)

Australasia

Risk Level: <5% | 5% to <10% | 10% to <20% | 20% to <30% | ≥30%

People without Diabetes

Age (years)	SBP (mmHg)	Men Non-smoker <4	4-4.9	5-5.9	6-6.9	≥ 7	Men Smoker <4	4-4.9	5-5.9	6-6.9	≥ 7	Women Non-smoker <4	4-4.9	5-5.9	6-6.9	≥ 7	Women Smoker <4	4-4.9	5-5.9	6-6.9	≥ 7
70-74	≥180	26	28	31	34	36	32	35	38	41	44	18	19	20	21	23	27	28	30	32	34
	160-179	22	24	26	28	31	27	30	32	35	38	14	15	16	17	18	22	23	25	26	28
	140-159	18	20	22	24	26	23	25	27	30	33	11	12	13	14	15	18	19	20	21	23
	120-139	15	17	18	20	22	19	21	23	25	28	9	10	10	11	12	14	15	16	17	19
	<120	13	14	15	17	19	16	17	19	21	23	7	8	8	9	10	11	12	13	14	15
65-69	≥180	21	23	25	27	30	27	30	33	36	39	13	13	14	16	17	21	23	25	27	29
	160-179	17	19	21	23	25	22	25	27	30	33	10	11	11	12	13	17	18	20	21	23
	140-159	14	15	17	19	21	18	20	23	25	28	8	8	9	10	10	13	14	15	17	18
	120-139	11	12	14	16	17	15	17	19	21	23	6	6	7	8	8	10	11	12	13	15
	<120	9	10	11	13	14	12	14	15	17	19	5	5	5	6	6	8	9	10	10	11
60-64	≥180	16	18	20	22	25	23	25	28	31	35	9	10	10	11	13	17	18	20	22	24
	160-179	13	14	16	18	20	18	20	23	26	29	7	7	8	9	10	13	14	15	17	19
	140-159	10	12	13	15	17	15	17	19	21	24	5	6	6	7	7	10	11	12	13	15
	120-139	8	9	11	12	14	12	13	15	17	19	4	4	5	5	6	8	8	9	10	11
	<120	7	7	8	10	11	10	11	12	14	16	3	3	3	4	4	6	6	7	8	9
55-59	≥180	13	14	16	18	21	19	21	24	27	30	6	7	7	8	9	13	14	16	18	20
	160-179	10	11	13	14	17	15	17	19	22	25	5	5	6	6	7	10	11	12	14	15
	140-159	8	9	10	11	13	12	13	15	18	20	3	4	4	5	5	7	8	9	10	12
	120-139	6	7	8	9	11	9	11	12	14	16	2	3	3	3	4	5	6	7	8	9
	<120	5	5	6	7	9	7	8	10	11	13	2	2	2	3	3	4	5	5	6	7
50-54	≥180	10	11	13	15	17	16	18	20	23	27	4	5	5	6	7	10	11	13	15	17
	160-179	7	8	10	11	13	12	14	16	18	21	3	3	4	4	5	7	8	9	11	12
	140-159	6	7	8	9	10	9	11	13	15	17	2	2	3	3	4	5	6	7	8	9
	120-139	4	5	6	7	8	7	8	10	12	14	2	2	2	2	3	4	4	5	6	7
	<120	3	4	5	6	7	6	7	8	9	11	1	1	1	2	2	3	3	4	4	5
45-49	≥180	7	9	10	12	14	13	15	17	20	23	3	3	4	4	5	8	9	10	12	14
	160-179	6	6	8	9	11	10	11	13	16	18	2	2	3	3	4	6	6	7	9	10
	140-159	4	5	6	7	8	7	9	10	12	14	1	2	2	2	2	4	5	5	6	7
	120-139	3	4	4	5	6	6	7	8	10	11	1	1	1	2	2	3	3	4	4	5
	<120	2	3	3	4	5	4	5	6	7	9	1	1	1	1	1	2	2	3	3	4
40-44	≥180	6	7	8	9	11	11	12	15	17	20	2	2	3	3	4	6	7	8	10	11
	160-179	4	5	6	7	8	8	9	11	13	16	1	2	2	2	3	4	5	6	7	8
	140-159	3	4	4	5	7	6	7	8	10	12	1	1	1	1	2	3	3	4	5	6
	120-139	2	3	3	4	5	4	5	6	8	10	1	1	1	1	1	2	2	3	3	4
	<120	2	2	3	3	4	3	4	5	6	7	0	0	1	1	1	1	2	2	2	3

Total cholesterol (mmol/l)

Australasia

Risk Level: <5% | 5% to <10% | 10% to <20% | 20% to <30% | ≥30%

People with Diabetes

Age (years)	SBP (mmHg)	Men Non-smoker <4	4-4.9	5-5.9	6-6.9	≥ 7	Men Smoker <4	4-4.9	5-5.9	6-6.9	≥ 7	Women Non-smoker <4	4-4.9	5-5.9	6-6.9	≥ 7	Women Smoker <4	4-4.9	5-5.9	6-6.9	≥ 7
70-74	≥180	34	37	40	43	47	42	45	48	52	56	29	31	33	35	37	43	45	48	50	53
	160-179	29	31	34	37	41	36	39	42	45	49	24	25	27	29	31	36	38	40	42	45
	140-159	24	27	29	32	35	30	33	36	39	42	19	21	22	23	25	29	31	33	35	38
	120-139	20	22	25	27	30	26	28	31	34	37	16	17	18	19	20	24	26	27	29	31
	<120	17	19	21	23	25	21	24	26	29	31	13	13	14	15	16	20	21	22	24	25
65-69	≥180	29	32	35	38	42	37	41	44	48	52	23	25	26	28	31	37	40	43	46	49
	160-179	24	26	29	32	35	31	34	38	41	45	18	20	21	23	25	30	33	35	38	41
	140-159	20	22	24	27	30	26	29	32	35	39	14	15	17	18	20	24	26	28	31	33
	120-139	16	18	20	22	25	22	24	27	29	33	11	12	13	14	16	19	21	23	25	27
	<120	13	15	17	19	21	18	20	22	25	28	9	10	10	11	12	15	17	18	20	22
60-64	≥180	24	27	30	33	37	34	37	41	45	49	18	19	21	23	25	32	35	38	41	45
	160-179	20	22	24	27	30	27	30	34	37	41	14	15	16	18	20	26	28	30	33	37
	140-159	16	18	20	22	25	22	25	28	31	35	10	11	13	14	15	20	22	24	26	29
	120-139	13	14	16	18	21	18	20	23	26	29	8	9	10	11	12	15	17	19	21	23
	<120	10	12	13	15	17	15	17	19	21	24	6	7	7	8	9	12	13	15	16	18
55-59	≥180	20	23	25	29	32	30	33	37	41	46	14	15	17	18	21	28	31	34	37	41
	160-179	16	18	20	23	26	24	27	30	34	38	10	11	13	14	16	21	24	26	29	33
	140-159	13	14	16	19	21	19	22	24	28	31	8	8	9	11	12	16	18	20	23	26
	120-139	10	11	13	15	17	15	17	20	23	26	6	6	7	8	9	12	14	16	18	20
	<120	8	9	10	12	14	12	14	16	18	21	4	5	5	6	7	9	10	12	13	15
50-54	≥180	17	19	22	25	28	27	30	34	38	43	11	12	13	15	17	24	27	30	34	38
	160-179	13	15	17	19	22	21	24	27	31	35	8	8	10	11	12	18	20	23	26	29
	140-159	10	11	13	15	18	16	19	21	25	28	5	6	7	8	9	13	15	17	20	22
	120-139	8	9	10	12	14	13	15	17	20	23	4	4	5	6	7	10	11	13	15	17
	<120	6	7	8	10	11	10	11	13	16	18	3	3	4	4	5	7	8	10	11	13
45-49	≥180	14	16	18	21	24	24	27	30	35	40	8	9	10	12	13	21	23	27	30	35
	160-179	11	12	14	16	19	18	21	24	28	32	6	6	7	8	10	15	17	20	23	26
	140-159	8	9	11	13	15	14	16	19	22	25	4	4	5	6	7	11	12	14	17	20
	120-139	6	7	8	10	12	10	12	14	17	20	3	3	4	4	5	8	9	10	12	15
	<120	5	5	6	8	9	8	9	11	13	16	2	2	3	3	4	6	6	8	9	11
40-44	≥180	12	13	15	18	21	21	24	28	32	37	6	7	8	9	11	18	20	23	27	32
	160-179	9	10	12	14	16	16	18	21	25	29	4	5	6	6	8	12	14	17	20	23
	140-159	6	7	9	10	13	12	14	16	19	23	3	3	4	5	5	9	10	12	14	17
	120-139	5	5	7	8	10	9	10	12	15	18	2	2	3	3	4	6	7	9	10	13
	<120	3	4	5	6	7	7	8	9	12	14	1	2	2	2	3	4	5	6	7	9

Total cholesterol (mmol/l)

WHO cardiovascular disease risk non-laboratory-based charts

North Africa and Middle East

Risk Level: <5% | 5% to <10% | 10% to <20% | 20% to <30% | ≥30%

Non-laboratory based risk chart

Age (years)	SBP (mmHg)	Men Non-smoker					Men Smoker					Women Non-smoker					Women Smoker				
	Body mass index (kg/m^2)	<20	20-24	25-29	30-35	≥ 35	<20	20-24	25-29	30-35	≥ 35	<20	20-24	25-29	30-35	≥ 35	<20	20-24	25-29	30-35	≥ 35
70-74	≥180	40	42	44	47	50	49	51	54	57	60	33	34	35	36	37	43	44	46	47	48
	160-179	33	35	38	40	42	42	44	47	49	52	29	29	30	31	32	38	39	40	41	42
	140-159	28	30	32	34	36	35	37	40	42	45	25	25	26	27	28	33	34	35	36	37
	120-139	23	25	26	28	30	29	31	33	36	38	21	22	23	23	24	29	30	30	31	32
	<120	19	20	22	23	25	24	26	28	30	32	18	19	19	20	21	25	26	26	27	28
65-69	≥180	31	34	36	39	42	42	45	48	51	55	27	28	29	30	31	38	39	41	42	43
	160-179	26	28	30	32	35	35	37	40	43	47	23	24	24	25	26	33	34	35	36	37
	140-159	21	22	24	27	29	28	31	33	36	39	19	20	21	21	22	28	29	30	31	32
	120-139	17	18	20	22	23	23	25	27	30	32	16	17	17	18	19	24	25	25	26	27
	<120	13	15	16	17	19	19	20	22	24	26	13	14	15	15	16	20	21	22	22	23
60-64	≥180	25	27	30	32	36	35	39	42	46	50	22	23	23	24	25	34	35	36	37	39
	160-179	19	21	24	26	29	28	31	34	38	41	18	19	19	20	21	28	29	30	32	33
	140-159	15	17	19	21	23	23	25	28	31	34	15	15	16	17	17	23	24	25	27	28
	120-139	12	13	15	16	18	18	20	22	25	27	12	13	13	14	14	19	20	21	22	23
	<120	9	10	12	13	14	14	16	18	20	22	10	10	11	11	12	16	17	18	18	19
55-59	≥180	19	21	24	27	30	30	33	37	41	45	18	18	19	20	21	29	31	32	33	35
	160-179	15	16	18	21	23	23	26	29	33	37	14	15	15	16	17	24	25	26	28	29
	140-159	11	13	14	16	18	18	20	23	26	29	11	12	12	13	14	20	21	22	23	24
	120-139	9	10	11	12	14	14	16	18	20	23	9	9	10	10	11	16	17	18	18	19
	<120	6	7	8	9	11	11	12	14	16	18	7	8	8	8	9	13	13	14	15	16
50-54	≥180	15	17	19	22	25	25	28	32	36	41	14	15	15	16	17	26	27	28	29	31
	160-179	11	13	14	16	19	19	22	25	28	32	11	12	12	13	13	20	21	23	24	25
	140-159	8	9	11	12	14	14	16	19	22	25	9	9	10	10	11	16	17	18	19	20
	120-139	6	7	8	9	11	11	12	14	17	19	7	7	7	8	8	13	14	14	15	16
	<120	4	5	6	7	8	8	9	11	13	15	5	6	6	6	7	10	11	11	12	13
45-49	≥180	11	13	15	17	20	21	24	27	32	37	11	12	12	13	14	22	23	25	26	28
	160-179	8	10	11	13	15	15	18	21	24	28	9	9	10	10	11	17	18	19	21	22
	140-159	6	7	8	9	11	11	13	15	18	21	7	7	7	8	8	13	14	15	16	17
	120-139	4	5	6	7	8	8	10	11	14	16	5	5	6	6	6	10	11	12	13	14
	<120	3	4	4	5	6	6	7	8	10	12	4	4	4	5	5	8	9	9	10	11
40-44	≥180	9	10	12	14	17	17	20	24	28	33	9	9	10	11	11	19	20	22	23	24
	160-179	6	7	8	10	12	12	15	17	21	25	7	7	7	8	8	15	16	17	18	19
	140-159	4	5	6	7	9	9	11	13	15	18	5	5	6	6	6	11	12	13	14	15
	120-139	3	4	4	5	6	6	8	9	11	13	4	4	4	5	5	8	9	10	10	11
	<120	2	3	3	4	4	4	5	7	8	10	3	3	3	3	4	6	7	7	8	9

Body mass index (kg/m^2)

Central Sub-Saharan Africa

Risk Level: <5% | 5% to <10% | 10% to <20% | 20% to <30% | ≥30%

Non-laboratory based risk chart

Age (years)	SBP (mmHg)	Men Non-smoker BMI <20	20-24	25-29	30-35	≥ 35	Men Smoker BMI <20	20-24	25-29	30-35	≥ 35	Women Non-smoker BMI <20	20-24	25-29	30-35	≥ 35	Women Smoker BMI <20	20-24	25-29	30-35	≥ 35
70-74	≥180	25	26	28	30	32	31	33	35	37	39	24	25	26	27	28	34	36	37	39	40
	160-179	20	22	23	25	26	26	28	29	31	33	20	20	21	22	23	29	30	31	32	34
	140-159	17	18	19	20	22	21	23	24	26	28	16	17	18	18	19	24	25	26	27	28
	120-139	14	15	16	17	18	18	19	20	22	23	13	14	14	15	16	20	21	21	22	23
	<120	11	12	13	14	15	14	15	17	18	19	11	11	12	12	13	16	17	18	18	19
65-69	≥180	19	21	22	24	26	26	28	30	33	35	18	19	20	21	22	29	30	32	33	35
	160-179	15	17	18	20	21	21	23	25	27	29	15	15	16	17	17	24	25	26	27	29
	140-159	12	13	15	16	17	17	18	20	22	24	12	12	13	13	14	19	20	21	22	23
	120-139	10	11	12	13	14	14	15	16	18	19	9	10	10	11	11	15	16	17	18	19
	<120	8	8	9	10	11	11	12	13	14	15	7	8	8	9	9	12	13	14	14	15
60-64	≥180	15	16	18	20	22	22	24	26	29	32	14	14	15	16	17	24	26	27	28	30
	160-179	12	13	14	16	17	17	19	21	23	25	11	11	12	12	13	19	20	21	23	24
	140-159	9	10	11	12	13	13	15	16	18	20	8	9	9	10	10	15	16	17	18	19
	120-139	7	8	9	9	11	11	12	13	14	16	6	7	7	8	8	12	13	13	14	15
	<120	5	6	7	7	8	8	9	10	11	13	5	5	6	6	6	9	10	10	11	12
55-59	≥180	11	13	14	16	18	18	20	22	25	28	10	11	12	12	13	20	21	23	24	26
	160-179	9	10	11	12	14	14	16	17	20	22	8	8	9	9	10	16	17	18	19	20
	140-159	7	7	8	9	11	11	12	13	15	17	6	6	7	7	7	12	13	14	14	15
	120-139	5	6	6	7	8	8	9	10	12	13	4	5	5	5	6	9	10	10	11	12
	<120	4	4	5	5	6	6	7	8	9	10	3	4	4	4	4	7	7	8	8	9
50-54	≥180	9	10	11	13	15	15	17	19	22	25	8	8	9	9	10	17	18	19	21	22
	160-179	7	7	8	10	11	11	13	15	17	19	6	6	6	7	7	13	13	14	16	17
	140-159	5	5	6	7	8	8	10	11	13	15	4	4	5	5	5	9	10	11	12	13
	120-139	4	4	5	5	6	6	7	8	9	11	3	3	3	4	4	7	7	8	9	9
	<120	3	3	3	4	5	5	5	6	7	8	2	2	3	3	3	5	6	6	6	7
45-49	≥180	7	8	9	10	12	12	14	16	19	22	6	6	7	7	8	14	15	16	17	19
	160-179	5	6	6	7	9	9	10	12	14	17	4	5	5	5	5	10	11	12	13	14
	140-159	4	4	5	5	6	7	8	9	10	12	3	3	3	4	4	7	8	9	9	10
	120-139	3	3	3	4	5	5	6	6	8	9	2	2	2	3	3	5	6	6	7	7
	<120	2	2	2	3	3	3	4	5	6	7	2	2	2	2	2	4	4	5	5	5
40-44	≥180	5	6	7	8	10	10	12	14	16	19	5	5	5	5	6	12	13	14	15	16
	160-179	4	4	5	6	7	7	8	10	12	14	3	3	4	4	4	8	9	10	10	11
	140-159	3	3	3	4	5	5	6	7	9	10	2	2	2	3	3	6	6	7	7	8
	120-139	2	2	2	3	3	4	4	5	6	7	1	2	2	2	2	4	4	5	5	6
	<120	1	1	2	2	2	3	3	4	4	5	1	1	1	1	1	3	3	3	4	4

Body mass index (kg/m^2)

Eastern Sub-Saharan Africa

Risk Level: <5% | 5% to <10% | 10% to <20% | 20% to <30% | ≥30%

Non-laboratory based risk chart

Men

Age (years)	SBP (mmHg)	Non-smoker BMI <20	Non-smoker 20-24	Non-smoker 25-29	Non-smoker 30-35	Non-smoker ≥ 35	Smoker BMI <20	Smoker 20-24	Smoker 25-29	Smoker 30-35	Smoker ≥ 35
70-74	≥180	24	25	27	29	30	30	32	34	36	38
	160-179	20	21	22	24	25	25	26	28	30	32
	140-159	16	17	18	20	21	21	22	23	25	27
	120-139	13	14	15	16	17	17	18	19	21	22
	<120	11	11	12	13	14	14	15	16	17	18
65-69	≥180	19	20	22	23	25	25	27	29	31	34
	160-179	15	16	17	19	20	20	22	24	26	28
	140-159	12	13	14	15	16	16	18	19	21	23
	120-139	9	10	11	12	13	13	14	15	17	18
	<120	8	8	9	10	11	10	11	12	14	15
60-64	≥180	14	16	17	19	21	21	23	25	28	30
	160-179	11	12	14	15	16	17	18	20	22	24
	140-159	9	10	11	12	13	13	14	16	17	19
	120-139	7	7	8	9	10	10	11	12	14	15
	<120	5	6	6	7	8	8	9	10	11	12
55-59	≥180	11	12	14	15	17	17	19	22	24	27
	160-179	8	9	11	12	13	13	15	17	19	21
	140-159	6	7	8	9	10	10	11	13	15	16
	120-139	5	5	6	7	8	8	9	10	11	13
	<120	4	4	5	5	6	6	7	8	9	10
50-54	≥180	9	10	11	12	14	14	16	18	21	24
	160-179	6	7	8	9	11	11	12	14	16	18
	140-159	5	5	6	7	8	8	9	11	12	14
	120-139	3	4	4	5	6	6	7	8	9	10
	<120	3	3	3	4	4	4	5	6	7	8
45-49	≥180	7	8	9	10	11	12	14	16	18	21
	160-179	5	5	6	7	8	9	10	12	14	16
	140-159	3	4	5	5	6	6	7	9	10	12
	120-139	2	3	3	4	4	5	5	6	7	9
	<120	2	2	2	3	3	3	4	5	5	6
40-44	≥180	5	6	7	8	9	10	11	13	16	19
	160-179	4	4	5	6	7	7	8	10	11	14
	140-159	2	3	3	4	5	5	6	7	8	10
	120-139	2	2	2	3	3	3	4	5	6	7
	<120	1	1	2	2	2	2	3	4	4	5

Women

Age (years)	SBP (mmHg)	Non-smoker BMI <20	Non-smoker 20-24	Non-smoker 25-29	Non-smoker 30-35	Non-smoker ≥ 35	Smoker BMI <20	Smoker 20-24	Smoker 25-29	Smoker 30-35	Smoker ≥ 35
70-74	≥180	18	18	19	20	20	25	26	26	27	28
	160-179	15	15	16	16	17	21	22	22	23	24
	140-159	12	13	13	14	14	18	18	19	19	20
	120-139	10	11	11	11	12	15	15	16	16	17
	<120	9	9	9	10	10	12	13	13	14	14
65-69	≥180	14	14	15	16	16	21	22	23	24	24
	160-179	11	12	12	13	13	17	18	19	20	20
	140-159	9	10	10	10	11	14	15	15	16	17
	120-139	8	8	8	8	9	12	12	13	13	14
	<120	6	6	7	7	7	10	10	10	11	11
60-64	≥180	11	11	12	12	13	18	19	20	20	21
	160-179	9	9	9	10	10	15	15	16	16	17
	140-159	7	7	7	8	8	12	12	13	13	14
	120-139	5	6	6	6	6	9	10	10	11	11
	<120	4	4	5	5	5	7	8	8	8	9
55-59	≥180	9	9	9	10	10	15	16	17	18	19
	160-179	7	7	7	8	8	12	13	13	14	15
	140-159	5	5	6	6	6	9	10	10	11	11
	120-139	4	4	4	4	5	7	8	8	8	9
	<120	3	3	3	3	4	6	6	6	7	7
50-54	≥180	7	7	7	8	8	13	14	14	15	16
	160-179	5	5	5	6	6	10	10	11	12	12
	140-159	4	4	4	4	5	8	8	8	9	9
	120-139	3	3	3	3	3	6	6	6	7	7
	<120	2	2	2	2	3	4	5	5	5	6
45-49	≥180	5	5	6	6	6	11	12	12	13	14
	160-179	4	4	4	4	5	8	9	9	10	10
	140-159	3	3	3	3	3	6	6	7	7	8
	120-139	2	2	2	2	3	4	5	5	5	6
	<120	1	2	2	2	2	3	4	4	4	4
40-44	≥180	4	4	4	5	5	9	10	11	11	12
	160-179	3	3	3	3	4	7	7	8	8	9
	140-159	2	2	2	2	3	5	5	6	6	6
	120-139	1	2	2	2	2	3	4	4	4	5
	<120	1	1	1	1	1	3	3	3	3	3

Body mass index (kg/m^2)

Southern Sub-Saharan Africa

Risk Level: <5% | 5% to <10% | 10% to <20% | 20% to <30% | ≥30%

Non-laboratory based risk chart

Age (years)	SBP (mmHg)	Men										Women									
		Non-smoker					Smoker					Non-smoker					Smoker				
		<20	20-24	25-29	30-35	≥ 35	<20	20-24	25-29	30-35	≥ 35	<20	20-24	25-29	30-35	≥ 35	<20	20-24	25-29	30-35	≥ 35
70-74	≥180	23	24	26	28	30	29	31	33	35	38	20	20	21	22	22	27	28	29	30	31
	160-179	19	20	21	23	25	24	26	28	29	31	17	17	18	18	19	23	24	25	26	26
	140-159	15	16	18	19	20	20	21	23	24	26	14	14	15	15	16	20	20	21	22	22
	120-139	12	13	14	15	17	16	17	19	20	21	12	12	12	13	13	16	17	18	18	19
	<120	10	11	12	12	13	13	14	15	16	18	10	10	10	11	11	14	14	15	15	16
65-69	≥180	18	19	21	23	25	24	26	29	31	34	15	16	17	17	18	24	24	25	26	27
	160-179	14	15	17	18	20	20	21	23	25	27	13	13	14	14	15	19	20	21	22	23
	140-159	11	12	13	14	16	16	17	19	20	22	10	11	11	11	12	16	17	17	18	19
	120-139	9	10	11	12	13	12	14	15	16	18	8	9	9	9	10	13	14	14	15	15
	<120	7	8	8	9	10	10	11	12	13	14	7	7	7	8	8	11	11	11	12	12
60-64	≥180	14	15	16	18	20	20	22	24	27	30	12	13	13	14	14	20	21	22	23	24
	160-179	11	12	13	14	16	16	17	19	21	24	10	10	10	11	11	16	17	18	18	19
	140-159	8	9	10	11	12	12	14	15	17	19	8	8	8	9	9	13	13	14	15	15
	120-139	6	7	8	9	10	10	11	12	13	15	6	6	7	7	7	10	11	11	12	12
	<120	5	5	6	7	7	7	8	9	10	12	5	5	5	5	6	8	9	9	9	10
55-59	≥180	10	12	13	15	16	17	19	21	23	26	9	10	10	11	11	17	18	19	20	21
	160-179	8	9	10	11	13	13	14	16	18	21	7	8	8	8	9	13	14	15	15	16
	140-159	6	7	7	8	10	10	11	12	14	16	6	6	6	6	7	10	11	11	12	13
	120-139	4	5	6	6	7	7	8	9	11	12	4	5	5	5	5	8	8	9	9	10
	<120	3	4	4	5	5	5	6	7	8	9	3	3	4	4	4	6	7	7	7	8
50-54	≥180	8	9	10	12	13	14	16	18	20	23	7	8	8	8	9	14	15	16	17	18
	160-179	6	7	8	9	10	10	12	13	15	18	6	6	6	6	7	11	12	12	13	14
	140-159	4	5	6	6	7	7	9	10	12	13	4	4	5	5	5	8	9	9	10	11
	120-139	3	4	4	5	5	6	6	7	9	10	3	3	3	4	4	6	7	7	8	8
	<120	2	3	3	3	4	4	5	5	6	7	2	2	3	3	3	5	5	5	6	6
45-49	≥180	6	7	8	9	11	11	13	15	18	21	6	6	6	7	7	12	13	14	15	15
	160-179	4	5	6	7	8	8	9	11	13	15	4	4	5	5	5	9	10	10	11	12
	140-159	3	4	4	5	6	6	7	8	9	11	3	3	3	4	4	7	7	8	8	9
	120-139	2	3	3	3	4	4	5	6	7	8	2	2	2	3	3	5	5	6	6	7
	<120	2	2	2	3	3	3	4	4	5	6	2	2	2	2	2	4	4	4	5	5
40-44	≥180	5	5	6	7	9	9	11	13	15	18	4	5	5	5	6	10	11	12	12	13
	160-179	3	4	4	5	6	6	8	9	11	13	3	3	4	4	4	7	8	9	9	10
	140-159	2	3	3	4	4	5	5	6	8	9	2	2	2	3	3	5	6	6	7	7
	120-139	2	2	2	3	3	3	4	5	6	7	2	2	2	2	2	4	4	4	5	5
	<120	1	1	2	2	2	2	3	3	4	5	1	1	1	1	1	3	3	3	4	4

Body mass index (kg/m^2)

Western Sub-Saharan Africa

Risk Level: <5% | 5% to <10% | 10% to <20% | 20% to <30% | ≥30%

Non-laboratory based risk chart

Age (years)	SBP (mmHg)	Men Non-smoker					Men Smoker					Women Non-smoker					Women Smoker				
	BMI (kg/m^2)	<20	20-24	25-29	30-35	≥ 35	<20	20-24	25-29	30-35	≥ 35	<20	20-24	25-29	30-35	≥ 35	<20	20-24	25-29	30-35	≥ 35
70-74	≥180	23	24	26	28	30	29	31	33	36	38	20	21	21	22	23	29	30	31	32	33
70-74	160-179	19	20	21	23	25	24	26	28	30	32	17	17	18	18	19	24	25	26	27	28
70-74	140-159	15	16	17	19	20	20	21	23	24	26	14	14	15	15	16	20	21	21	22	23
70-74	120-139	12	13	14	15	16	16	17	18	20	21	11	12	12	13	13	17	17	18	18	19
70-74	<120	10	11	11	12	13	13	14	15	16	17	9	10	10	10	11	14	14	15	15	16
65-69	≥180	17	19	21	22	24	24	26	29	31	34	15	16	17	17	18	24	25	26	27	29
65-69	160-179	14	15	16	18	20	19	21	23	25	27	12	13	13	14	15	20	21	21	22	23
65-69	140-159	11	12	13	14	16	15	17	18	20	22	10	10	11	11	12	16	17	17	18	19
65-69	120-139	9	9	10	11	12	12	13	15	16	18	8	8	9	9	9	13	13	14	15	15
65-69	<120	7	7	8	9	10	10	11	12	13	14	6	7	7	7	8	10	11	11	12	12
60-64	≥180	13	15	16	18	20	20	22	24	27	30	12	12	13	14	14	20	21	22	24	25
60-64	160-179	10	11	13	14	16	16	17	19	21	24	9	10	10	11	11	16	17	18	19	20
60-64	140-159	8	9	10	11	12	12	13	15	17	19	7	8	8	8	9	13	13	14	15	16
60-64	120-139	6	7	7	8	9	9	10	12	13	15	6	6	6	6	7	10	11	11	12	12
60-64	<120	5	5	6	6	7	7	8	9	10	11	4	5	5	5	5	8	8	9	9	10
55-59	≥180	10	11	13	14	16	16	18	21	23	26	9	10	10	11	11	17	18	19	20	21
55-59	160-179	8	9	10	11	12	12	14	16	18	20	7	7	8	8	8	13	14	15	16	16
55-59	140-159	6	6	7	8	9	9	11	12	14	16	5	5	6	6	6	10	11	11	12	13
55-59	120-139	4	5	5	6	7	7	8	9	10	12	4	4	4	5	5	8	8	9	9	10
55-59	<120	3	4	4	5	5	5	6	7	8	9	3	3	3	3	4	6	6	7	7	7
50-54	≥180	8	9	10	11	13	13	15	18	20	23	7	7	8	8	9	14	15	16	17	18
50-54	160-179	6	6	7	8	10	10	11	13	15	18	5	5	6	6	6	11	11	12	13	14
50-54	140-159	4	5	5	6	7	7	8	10	11	13	4	4	4	4	5	8	9	9	10	10
50-54	120-139	3	3	4	5	5	5	6	7	8	10	3	3	3	3	3	6	6	7	7	8
50-54	<120	2	2	3	3	4	4	5	5	6	7	2	2	2	2	3	4	5	5	5	6
45-49	≥180	6	7	8	9	11	11	13	15	17	20	5	6	6	6	7	12	13	14	15	16
45-49	160-179	4	5	6	7	8	8	9	11	13	15	4	4	4	5	5	9	9	10	11	11
45-49	140-159	3	3	4	5	5	6	7	8	9	11	3	3	3	3	3	6	7	7	8	8
45-49	120-139	2	2	3	3	4	4	5	6	7	8	2	2	2	2	2	5	5	5	6	6
45-49	<120	1	2	2	2	3	3	3	4	5	6	1	1	2	2	2	3	4	4	4	4
40-44	≥180	5	5	6	7	9	9	11	13	15	18	4	4	5	5	5	10	11	12	12	13
40-44	160-179	3	4	4	5	6	6	7	9	11	13	3	3	3	3	4	7	8	8	9	10
40-44	140-159	2	3	3	4	4	4	5	6	8	9	2	2	2	2	3	5	5	6	6	7
40-44	120-139	1	2	2	2	3	3	4	4	5	6	1	1	2	2	2	4	4	4	4	5
40-44	<120	1	1	1	2	2	2	3	3	4	5	1	1	1	1	1	2	3	3	3	3

Body mass index (kg/m^2)

Southern Latin America

Risk Level: <5% | 5% to <10% | 10% to <20% | 20% to <30% | ≥30%

Non-laboratory based risk chart

Age (years)	SBP (mmHg)	Men Non-smoker					Men Smoker					Women Non-smoker					Women Smoker				
	Body mass index (kg/m^2)	<20	20-24	25-29	30-35	≥ 35	<20	20-24	25-29	30-35	≥ 35	<20	20-24	25-29	30-35	≥ 35	<20	20-24	25-29	30-35	≥ 35
70-74	≥180	26	27	29	31	33	32	34	37	39	41	19	20	21	22	23	29	31	32	34	36
	160-179	21	23	24	26	28	27	29	31	33	35	15	16	17	18	18	24	25	26	28	29
	140-159	18	19	20	22	23	23	24	26	28	30	12	13	13	14	15	19	20	21	22	24
	120-139	15	16	17	18	19	19	20	22	23	25	10	10	11	11	12	15	16	17	18	19
	<120	12	13	14	15	16	16	17	18	19	21	8	8	8	9	9	12	13	14	14	15
65-69	≥180	20	22	24	26	28	27	29	32	34	37	14	14	15	16	17	24	25	27	28	30
	160-179	16	18	19	21	23	22	24	26	28	31	11	11	12	13	13	19	20	21	22	24
	140-159	13	14	16	17	19	18	20	21	23	25	8	9	9	10	10	15	16	17	18	19
	120-139	11	12	13	14	15	15	16	18	19	21	6	7	7	8	8	11	12	13	14	15
	<120	8	9	10	11	12	12	13	14	16	17	5	5	5	6	6	9	9	10	11	11
60-64	≥180	16	17	19	21	23	23	25	28	30	33	10	11	11	12	13	19	21	22	24	25
	160-179	12	14	15	17	18	18	20	22	25	27	8	8	8	9	10	15	16	17	18	19
	140-159	10	11	12	13	15	14	16	18	20	22	6	6	6	7	7	11	12	13	14	15
	120-139	8	8	9	10	12	11	13	14	16	18	4	4	5	5	5	8	9	10	10	11
	<120	6	7	7	8	9	9	10	11	13	14	3	3	4	4	4	6	7	7	8	8
55-59	≥180	12	13	15	17	19	19	21	24	27	30	7	8	8	9	9	16	17	18	20	21
	160-179	9	10	12	13	15	15	17	19	21	24	5	6	6	6	7	12	12	13	15	16
	140-159	7	8	9	10	12	11	13	15	17	19	4	4	4	5	5	9	9	10	11	12
	120-139	5	6	7	8	9	9	10	11	13	15	3	3	3	3	4	6	7	7	8	9
	<120	4	5	5	6	7	7	8	9	10	11	2	2	2	2	3	5	5	5	6	6
50-54	≥180	9	11	12	14	16	16	18	20	23	27	5	6	6	6	7	13	14	15	16	18
	160-179	7	8	9	10	12	12	14	16	18	21	4	4	4	5	5	9	10	11	12	13
	140-159	5	6	7	8	9	9	10	12	14	16	3	3	3	3	4	6	7	8	8	9
	120-139	4	4	5	6	7	7	8	9	11	12	2	2	2	2	2	5	5	5	6	7
	<120	3	3	4	4	5	5	6	7	8	9	1	1	2	2	2	3	4	4	4	5
45-49	≥180	7	8	10	11	13	13	15	18	20	24	4	4	4	5	5	10	11	12	13	15
	160-179	5	6	7	8	10	10	11	13	15	18	3	3	3	3	4	7	8	8	9	10
	140-159	4	4	5	6	7	7	8	10	12	14	2	2	2	2	2	5	5	6	6	7
	120-139	3	3	4	4	5	5	6	7	9	10	1	1	1	2	2	3	4	4	4	5
	<120	2	2	3	3	4	4	5	5	6	8	1	1	1	1	1	2	3	3	3	3
40-44	≥180	5	6	8	9	11	11	13	15	18	21	3	3	3	4	4	8	9	10	11	12
	160-179	4	5	5	6	8	8	9	11	13	16	2	2	2	2	3	6	6	7	7	8
	140-159	3	3	4	5	6	6	7	8	10	12	1	1	1	2	2	4	4	5	5	6
	120-139	2	2	3	3	4	4	5	6	7	9	1	1	1	1	1	3	3	3	3	4
	<120	1	2	2	2	3	3	4	4	5	6	1	1	1	1	1	2	2	2	2	3

Body mass index (kg/m^2)

High-income North America

Risk Level: <5% | 5% to <10% | 10% to <20% | 20% to <30% | ≥30%

Non-laboratory based risk chart

Age (years)	SBP (mmHg)	Men Non-smoker					Men Smoker					Women Non-smoker					Women Smoker				
	BMI (kg/m^2)	<20	20-24	25-29	30-35	≥ 35	<20	20-24	25-29	30-35	≥ 35	<20	20-24	25-29	30-35	≥ 35	<20	20-24	25-29	30-35	≥ 35
70-74	≥180	31	33	35	37	39	38	40	42	45	47	25	26	27	28	29	36	37	38	40	41
70-74	160-179	26	28	29	31	33	32	34	36	38	40	21	21	22	23	24	30	31	32	33	35
70-74	140-159	22	23	25	26	28	27	29	31	33	35	17	18	18	19	20	25	26	27	28	29
70-74	120-139	18	20	21	22	24	23	25	26	28	29	14	15	15	16	16	21	22	22	23	24
70-74	<120	15	16	17	19	20	19	21	22	23	25	11	12	12	13	13	17	18	19	19	20
65-69	≥180	25	27	29	31	33	33	35	37	40	43	19	20	21	22	23	30	32	33	34	36
65-69	160-179	20	22	24	25	27	27	29	31	34	36	15	16	17	18	18	25	26	27	28	29
65-69	140-159	17	18	19	21	23	22	24	26	28	30	12	13	13	14	15	20	21	22	23	24
65-69	120-139	14	15	16	17	19	18	20	22	23	25	10	10	11	11	12	16	17	18	19	20
65-69	<120	11	12	13	14	15	15	16	18	19	21	8	8	9	9	9	13	14	14	15	16
60-64	≥180	20	21	23	25	28	28	30	33	36	39	14	15	16	17	18	25	27	28	29	31
60-64	160-179	16	17	19	21	23	22	25	27	29	32	11	12	12	13	14	20	21	22	24	25
60-64	140-159	13	14	15	17	18	18	20	22	24	26	9	9	10	10	11	16	17	18	19	20
60-64	120-139	10	11	12	13	15	15	16	18	20	22	7	7	8	8	8	12	13	14	15	16
60-64	<120	8	9	10	11	12	12	13	14	16	17	5	6	6	6	7	10	10	11	12	12
55-59	≥180	15	17	19	21	23	23	26	29	32	35	11	12	12	13	14	21	23	24	25	27
55-59	160-179	12	13	15	17	19	19	21	23	26	28	8	9	9	10	10	16	17	18	20	21
55-59	140-159	9	10	12	13	15	15	16	18	20	23	6	7	7	7	8	13	13	14	15	16
55-59	120-139	7	8	9	10	12	12	13	15	16	18	5	5	5	6	6	10	10	11	12	13
55-59	<120	6	6	7	8	9	9	10	12	13	15	4	4	4	4	5	7	8	8	9	10
50-54	≥180	12	14	15	17	20	20	22	25	28	32	8	9	9	10	10	18	19	20	21	23
50-54	160-179	9	10	12	13	15	15	17	20	22	25	6	6	7	7	8	13	14	15	16	17
50-54	140-159	7	8	9	10	12	12	13	15	17	20	4	5	5	5	6	10	11	11	12	13
50-54	120-139	5	6	7	8	9	9	10	12	14	16	3	3	4	4	4	7	8	9	9	10
50-54	<120	4	5	5	6	7	7	8	9	11	12	2	3	3	3	3	5	6	6	7	7
45-49	≥180	9	11	12	14	16	17	19	22	25	28	6	7	7	8	8	15	16	17	18	20
45-49	160-179	7	8	9	11	12	13	14	17	19	22	4	5	5	5	6	11	12	12	13	15
45-49	140-159	5	6	7	8	9	9	11	13	15	17	3	3	4	4	4	8	8	9	10	11
45-49	120-139	4	5	5	6	7	7	8	10	11	13	2	2	3	3	3	6	6	7	7	8
45-49	<120	3	3	4	5	5	5	6	7	9	10	2	2	2	2	2	4	4	5	5	6
40-44	≥180	7	8	10	12	14	14	16	19	22	26	5	5	5	6	6	12	13	14	15	17
40-44	160-179	5	6	7	9	10	10	12	14	17	20	3	3	4	4	4	9	9	10	11	12
40-44	140-159	4	5	5	6	8	8	9	11	13	15	2	2	3	3	3	6	7	7	8	9
40-44	120-139	3	3	4	5	6	6	7	8	9	11	2	2	2	2	2	4	5	5	6	6
40-44	<120	2	2	3	3	4	4	5	6	7	9	1	1	1	1	1	3	3	4	4	5

Body mass index (kg/m^2)

Caribbean

Risk Level: <5% | 5% to <10% | 10% to <20% | 20% to <30% | ≥30%

Non-laboratory based risk chart

Body mass index (kg/m^2)

Age (years)	SBP (mmHg)	Men Non-smoker <20	Men Non-smoker 20-24	Men Non-smoker 25-29	Men Non-smoker 30-35	Men Non-smoker ≥ 35	Men Smoker <20	Men Smoker 20-24	Men Smoker 25-29	Men Smoker 30-35	Men Smoker ≥ 35	Women Non-smoker <20	Women Non-smoker 20-24	Women Non-smoker 25-29	Women Non-smoker 30-35	Women Non-smoker ≥ 35	Women Smoker <20	Women Smoker 20-24	Women Smoker 25-29	Women Smoker 30-35	Women Smoker ≥ 35
70-74	≥180	23	25	27	29	31	30	32	34	37	39	19	20	21	22	22	28	29	30	31	32
	160-179	19	20	22	23	25	25	26	28	30	33	16	17	17	18	19	23	24	25	26	27
	140-159	15	17	18	19	21	20	22	23	25	27	14	14	15	15	16	20	20	21	22	23
	120-139	13	13	15	16	17	16	18	19	20	22	11	12	12	13	13	16	17	18	18	19
	<120	10	11	12	13	14	13	14	15	17	18	9	10	10	11	11	14	14	15	15	16
65-69	≥180	18	19	21	23	25	25	27	29	32	35	15	16	16	17	18	23	24	25	27	28
	160-179	14	15	17	18	20	20	22	24	26	28	12	13	13	14	15	19	20	21	22	23
	140-159	11	12	13	15	16	16	17	19	21	23	10	10	11	11	12	16	17	17	18	19
	120-139	9	10	11	12	13	13	14	15	17	18	8	8	9	9	10	13	14	14	15	16
	<120	7	8	8	9	10	10	11	12	13	14	7	7	7	8	8	11	11	12	12	13
60-64	≥180	14	15	17	18	21	20	23	25	28	31	12	12	13	13	14	20	21	22	23	24
	160-179	11	12	13	14	16	16	18	20	22	24	9	10	10	11	11	16	17	18	19	20
	140-159	8	9	10	11	12	12	14	15	17	19	7	8	8	8	9	13	13	14	15	16
	120-139	6	7	8	9	10	10	11	12	13	15	6	6	6	7	7	10	11	11	12	13
	<120	5	5	6	7	7	7	8	9	10	12	5	5	5	5	6	8	9	9	9	10
55-59	≥180	10	12	13	15	17	17	19	21	24	27	9	9	10	10	11	17	18	19	20	21
	160-179	8	9	10	11	13	13	14	16	19	21	7	7	8	8	9	13	14	15	16	17
	140-159	6	7	7	8	10	10	11	12	14	16	5	6	6	6	7	10	11	11	12	13
	120-139	4	5	6	6	7	7	8	9	11	12	4	4	5	5	5	8	8	9	10	10
	<120	3	4	4	5	5	5	6	7	8	9	3	3	4	4	4	6	7	7	7	8
50-54	≥180	8	9	10	12	14	14	16	18	21	24	7	7	8	8	9	14	15	16	17	18
	160-179	6	7	8	9	10	10	12	13	16	18	5	6	6	6	7	11	11	12	13	14
	140-159	4	5	6	6	7	7	9	10	12	14	4	4	4	5	5	8	9	9	10	11
	120-139	3	4	4	5	5	6	6	7	9	10	3	3	3	4	4	6	7	7	8	8
	<120	2	3	3	3	4	4	5	5	6	7	2	2	3	3	3	5	5	5	6	6
45-49	≥180	6	7	8	9	11	11	13	15	18	21	5	6	6	6	7	12	13	14	14	16
	160-179	4	5	6	7	8	8	9	11	13	16	4	4	4	5	5	9	9	10	11	12
	140-159	3	4	4	5	6	6	7	8	10	11	3	3	3	4	4	7	7	8	8	9
	120-139	2	3	3	3	4	4	5	6	7	8	2	2	2	3	3	5	5	6	6	7
	<120	2	2	2	2	3	3	3	4	5	6	2	2	2	2	2	4	4	4	5	5
40-44	≥180	5	5	6	7	9	9	11	13	15	19	4	5	5	5	5	10	11	12	12	13
	160-179	3	4	4	5	6	6	8	9	11	13	3	3	3	4	4	7	8	8	9	10
	140-159	2	3	3	4	4	5	5	6	8	9	2	2	2	3	3	5	6	6	7	7
	120-139	2	2	2	3	3	3	4	5	5	7	2	2	2	2	2	4	4	4	5	5
	<120	1	1	1	2	2	2	3	3	4	5	1	1	1	1	1	3	3	3	4	4

Andean Latin America

Risk Level: <5% | 5% to <10% | 10% to <20% | 20% to <30% | ≥30%

Non-laboratory based risk chart

Age (years)	SBP (mmHg)	Men Non-smoker					Men Smoker					Women Non-smoker					Women Smoker				
	Body mass index (kg/m^2)	<20	20-24	25-29	30-35	≥ 35	<20	20-24	25-29	30-35	≥ 35	<20	20-24	25-29	30-35	≥ 35	<20	20-24	25-29	30-35	≥ 35
70-74	≥180	18	19	20	22	23	22	24	26	27	29	14	14	15	15	16	19	20	21	22	22
70-74	160-179	14	16	17	18	19	19	20	21	23	25	12	12	12	13	13	16	17	18	18	19
70-74	140-159	12	13	14	15	16	15	16	18	19	20	10	10	11	11	11	14	14	15	16	16
70-74	120-139	10	10	11	12	13	13	14	15	16	17	8	9	9	9	10	12	12	13	13	14
70-74	<120	8	9	9	10	11	10	11	12	13	14	7	7	7	8	8	10	10	11	11	11
65-69	≥180	14	15	16	17	19	19	20	22	24	26	11	11	12	12	13	17	17	18	19	20
65-69	160-179	11	12	13	14	15	15	16	18	20	21	9	9	10	10	10	14	14	15	16	16
65-69	140-159	9	10	10	11	12	12	13	14	16	17	7	8	8	8	9	11	12	12	13	13
65-69	120-139	7	8	8	9	10	10	11	12	13	14	6	6	7	7	7	9	10	10	11	11
65-69	<120	6	6	7	7	8	8	9	9	10	11	5	5	5	6	6	8	8	8	9	9
60-64	≥180	11	12	13	14	16	16	17	19	21	23	9	9	9	10	10	14	15	15	16	17
60-64	160-179	8	9	10	11	12	12	14	15	17	19	7	7	7	8	8	11	12	13	13	14
60-64	140-159	7	7	8	9	10	10	11	12	13	15	6	6	6	6	7	9	10	10	11	11
60-64	120-139	5	6	6	7	8	8	8	9	10	12	4	5	5	5	5	7	8	8	9	9
60-64	<120	4	4	5	5	6	6	7	7	8	9	4	4	4	4	4	6	6	7	7	7
55-59	≥180	8	9	10	11	13	13	14	16	18	21	7	7	7	8	8	12	13	13	14	15
55-59	160-179	6	7	8	9	10	10	11	13	14	16	5	6	6	6	6	10	10	11	11	12
55-59	140-159	5	5	6	7	8	8	9	10	11	12	4	4	5	5	5	8	8	8	9	9
55-59	120-139	4	4	5	5	6	6	7	7	8	10	3	3	4	4	4	6	6	7	7	7
55-59	<120	3	3	3	4	4	4	5	6	6	7	3	3	3	3	3	5	5	5	6	6
50-54	≥180	6	7	8	9	10	11	12	14	16	18	5	6	6	6	6	10	11	11	12	13
50-54	160-179	5	5	6	7	8	8	9	10	12	14	4	4	4	5	5	8	8	9	9	10
50-54	140-159	4	4	5	5	6	6	7	8	9	11	3	3	3	4	4	6	6	7	7	8
50-54	120-139	3	3	3	4	4	5	5	6	7	8	2	3	3	3	3	5	5	5	6	6
50-54	<120	2	2	3	3	3	3	4	4	5	6	2	2	2	2	2	4	4	4	4	5
45-49	≥180	5	6	6	7	9	9	10	12	14	16	4	4	5	5	5	9	9	10	10	11
45-49	160-179	4	4	5	5	6	6	7	9	10	12	3	3	3	4	4	7	7	7	8	9
45-49	140-159	3	3	3	4	5	5	5	6	8	9	2	2	3	3	3	5	5	6	6	6
45-49	120-139	2	2	3	3	3	3	4	5	6	7	2	2	2	2	2	4	4	4	5	5
45-49	<120	1	2	2	2	2	3	3	3	4	5	1	1	1	2	2	3	3	3	3	4
40-44	≥180	4	4	5	6	7	7	8	10	12	14	3	4	4	4	4	7	8	8	9	10
40-44	160-179	3	3	4	4	5	5	6	7	9	10	2	3	3	3	3	5	6	6	7	7
40-44	140-159	2	2	3	3	4	4	4	5	6	7	2	2	2	2	2	4	4	5	5	5
40-44	120-139	1	2	2	2	3	3	3	4	4	5	1	1	1	2	2	3	3	3	4	4
40-44	<120	1	1	1	2	2	2	2	3	3	4	1	1	1	1	1	2	2	3	3	3

Body mass index (kg/m^2)

Central Latin America

Risk Level: <5% | 5% to <10% | 10% to <20% | 20% to <30% | ≥30%

Non-laboratory based risk chart

Age (years)	SBP (mmHg)	Men										Women									
		Non-smoker					Smoker					Non-smoker					Smoker				
	Body mass index (kg/m^2)	<20	20-24	25-29	30-35	≥ 35	<20	20-24	25-29	30-35	≥ 35	<20	20-24	25-29	30-35	≥ 35	<20	20-24	25-29	30-35	≥ 35
70-74	≥180	20	21	23	25	26	26	28	30	32	34	16	17	18	18	19	23	24	25	26	27
	160-179	16	17	19	20	22	21	23	24	26	28	14	14	15	15	16	20	20	21	22	23
	140-159	13	14	15	16	18	17	19	20	22	23	11	12	12	13	13	16	17	18	19	19
	120-139	11	12	12	13	14	14	15	16	18	19	9	10	10	11	11	14	14	15	16	16
	<120	9	9	10	11	12	11	12	13	14	16	8	8	8	9	9	11	12	12	13	13
65-69	≥180	15	16	18	20	22	21	23	25	28	30	13	13	14	14	15	20	21	22	23	24
	160-179	12	13	14	16	17	17	19	20	22	25	10	11	11	12	12	16	17	18	19	20
	140-159	10	10	11	13	14	14	15	16	18	20	8	9	9	10	10	13	14	15	15	16
	120-139	8	8	9	10	11	11	12	13	14	16	7	7	7	8	8	11	11	12	13	13
	<120	6	7	7	8	9	9	9	10	11	13	6	6	6	6	7	9	9	10	10	11
60-64	≥180	11	13	14	16	18	17	19	22	24	27	10	10	11	11	12	17	17	18	19	21
	160-179	9	10	11	12	14	14	15	17	19	21	8	8	8	9	9	13	14	15	16	17
	140-159	7	8	9	10	11	11	12	13	15	17	6	6	7	7	7	11	11	12	13	13
	120-139	5	6	7	7	8	8	9	10	12	13	5	5	5	6	6	9	9	10	10	11
	<120	4	5	5	6	6	6	7	8	9	10	4	4	4	5	5	7	7	8	8	9
55-59	≥180	9	10	11	13	14	14	16	18	21	24	7	8	8	9	9	14	15	16	17	18
	160-179	7	7	8	10	11	11	12	14	16	18	6	6	6	7	7	11	12	12	13	14
	140-159	5	6	6	7	8	8	9	11	12	14	5	5	5	5	6	9	9	10	10	11
	120-139	4	4	5	5	6	6	7	8	9	11	4	4	4	4	4	7	7	8	8	9
	<120	3	3	4	4	5	5	5	6	7	8	3	3	3	3	3	5	6	6	6	7
50-54	≥180	7	8	9	10	12	12	13	15	18	21	6	6	6	7	7	12	13	13	14	15
	160-179	5	6	6	7	9	9	10	12	13	16	4	5	5	5	6	9	10	10	11	12
	140-159	4	4	5	5	6	6	7	9	10	12	3	4	4	4	4	7	7	8	8	9
	120-139	3	3	3	4	5	5	5	6	7	9	3	3	3	3	3	5	6	6	6	7
	<120	2	2	3	3	3	3	4	5	6	7	2	2	2	2	2	4	4	5	5	5
45-49	≥180	5	6	7	8	9	9	11	13	15	18	5	5	5	5	6	10	11	11	12	13
	160-179	4	4	5	6	7	7	8	10	11	14	3	4	4	4	4	7	8	9	9	10
	140-159	3	3	4	4	5	5	6	7	8	10	2	3	3	3	3	6	6	6	7	7
	120-139	2	2	3	3	4	4	4	5	6	7	2	2	2	2	2	4	4	5	5	6
	<120	1	2	2	2	3	3	3	4	4	5	1	1	2	2	2	3	3	4	4	4
40-44	≥180	4	5	5	6	8	8	9	11	13	16	4	4	4	4	4	8	9	10	10	11
	160-179	3	3	4	4	5	5	7	8	10	12	3	3	3	3	3	6	7	7	8	8
	140-159	2	2	3	3	4	4	5	6	7	8	2	2	2	2	2	4	5	5	6	6
	120-139	1	2	2	2	3	3	3	4	5	6	1	1	2	2	2	3	3	4	4	5
	<120	1	1	1	2	2	2	2	3	3	4	1	1	1	1	1	2	3	3	3	3

Tropical Latin America

Risk Level: <5% | 5% to <10% | 10% to <20% | 20% to <30% | ≥30%

Non-laboratory based risk chart

Age (years)	SBP (mmHg)	Men										Women									
		Non-smoker					Smoker					Non-smoker					Smoker				
		<20	20-24	25-29	30-35	≥ 35	<20	20-24	25-29	30-35	≥ 35	<20	20-24	25-29	30-35	≥ 35	<20	20-24	25-29	30-35	≥ 35
70-74	≥180	20	21	22	23	25	24	26	27	29	31	14	14	15	15	16	19	20	21	21	22
	160-179	16	17	18	19	21	20	22	23	24	26	12	12	13	13	13	16	17	18	18	19
	140-159	13	14	15	16	17	17	18	19	20	22	10	10	11	11	11	14	14	15	15	16
	120-139	11	12	12	13	14	14	15	16	17	18	8	9	9	9	10	12	12	13	13	13
	<120	9	10	10	11	12	12	12	13	14	15	7	7	8	8	8	10	10	11	11	11
65-69	≥180	15	17	18	19	21	21	22	24	26	28	11	11	12	12	13	17	17	18	19	19
	160-179	13	13	14	16	17	17	18	19	21	23	9	10	10	10	11	14	14	15	15	16
	140-159	10	11	12	13	14	14	15	16	17	18	8	8	8	8	9	11	12	12	13	13
	120-139	8	9	9	10	11	11	12	13	14	15	6	6	7	7	7	9	10	10	11	11
	<120	7	7	8	8	9	9	10	10	11	12	5	5	6	6	6	8	8	8	9	9
60-64	≥180	12	13	14	16	17	17	19	21	22	25	9	9	9	10	10	14	15	15	16	17
	160-179	10	10	11	12	14	14	15	16	18	20	7	7	8	8	8	12	12	13	13	14
	140-159	8	8	9	10	11	11	12	13	14	16	6	6	6	6	7	9	10	10	11	11
	120-139	6	6	7	8	8	9	9	10	11	12	5	5	5	5	5	8	8	8	9	9
	<120	5	5	6	6	7	7	7	8	9	10	4	4	4	4	4	6	6	7	7	7
55-59	≥180	10	11	12	13	14	15	16	18	20	22	7	7	8	8	8	12	13	13	14	15
	160-179	7	8	9	10	11	11	12	14	15	17	6	6	6	6	7	10	10	11	11	12
	140-159	6	6	7	8	8	9	10	11	12	13	4	5	5	5	5	8	8	8	9	9
	120-139	4	5	5	6	7	7	7	8	9	10	3	4	4	4	4	6	6	7	7	7
	<120	3	4	4	5	5	5	6	6	7	8	3	3	3	3	3	5	5	5	6	6
50-54	≥180	8	8	9	10	12	12	14	15	17	19	6	6	6	6	7	10	11	12	12	13
	160-179	6	6	7	8	9	9	10	12	13	15	4	5	5	5	5	8	9	9	9	10
	140-159	4	5	5	6	7	7	8	9	10	11	3	3	4	4	4	6	7	7	7	8
	120-139	3	4	4	4	5	5	6	7	8	9	3	3	3	3	3	5	5	5	6	6
	<120	2	3	3	3	4	4	4	5	6	6	2	2	2	2	2	4	4	4	4	5
45-49	≥180	6	7	8	8	10	10	12	13	15	17	5	5	5	5	5	9	9	10	11	11
	160-179	4	5	6	6	7	8	9	10	11	13	3	4	4	4	4	7	7	8	8	9
	140-159	3	4	4	5	5	6	6	7	8	10	3	3	3	3	3	5	5	6	6	6
	120-139	2	3	3	3	4	4	5	5	6	7	2	2	2	2	2	4	4	4	5	5
	<120	2	2	2	2	3	3	3	4	5	5	1	2	2	2	2	3	3	3	3	4
40-44	≥180	5	5	6	7	8	8	10	11	13	15	4	4	4	4	4	8	8	9	9	10
	160-179	3	4	4	5	6	6	7	8	9	11	3	3	3	3	3	6	6	6	7	7
	140-159	2	3	3	4	4	4	5	6	7	8	2	2	2	2	2	4	4	5	5	5
	120-139	2	2	2	3	3	3	4	4	5	6	1	2	2	2	2	3	3	3	4	4
	<120	1	1	2	2	2	2	3	3	4	4	1	1	1	1	1	2	2	3	3	3

Body mass index (kg/m^2)

East Asia

Risk Level: <5% | 5% to <10% | 10% to <20% | 20% to <30% | ≥30%

Non-laboratory based risk chart

Age (years)	SBP (mmHg)	Men Non-smoker					Men Smoker					Women Non-smoker					Women Smoker				
	Body mass index (kg/m^2)	<20	20-24	25-29	30-35	≥ 35	<20	20-24	25-29	30-35	≥ 35	<20	20-24	25-29	30-35	≥ 35	<20	20-24	25-29	30-35	≥ 35
70-74	≥180	34	36	38	40	42	42	45	47	49	52	28	29	30	31	32	39	40	41	42	43
	160-179	28	30	31	33	35	35	37	39	41	43	24	25	25	26	27	33	34	35	36	37
	140-159	23	24	25	27	28	29	30	32	34	36	20	21	21	22	22	28	29	29	30	31
	120-139	18	19	20	22	23	23	24	26	27	29	17	17	18	18	19	23	24	25	26	26
	<120	15	15	16	17	18	19	20	21	22	24	14	14	15	15	16	20	20	21	21	22
65-69	≥180	27	29	31	33	35	36	38	41	43	46	23	23	24	25	25	33	34	35	37	38
	160-179	21	23	24	26	28	29	31	33	35	37	19	19	20	20	21	28	29	29	30	31
	140-159	17	18	19	20	22	23	24	26	28	30	15	16	16	17	17	23	24	24	25	26
	120-139	13	14	15	16	17	18	19	20	22	24	12	13	13	13	14	19	19	20	21	21
	<120	10	11	12	12	13	14	15	16	17	19	10	10	11	11	11	15	16	16	17	17
60-64	≥180	21	23	25	27	29	30	32	35	38	41	18	19	19	20	21	29	30	31	32	33
	160-179	16	17	19	20	22	23	25	27	29	32	14	15	15	16	16	23	24	25	26	27
	140-159	12	13	14	16	17	18	19	21	23	25	11	12	12	13	13	19	19	20	21	22
	120-139	9	10	11	12	13	13	15	16	17	19	9	9	10	10	10	15	15	16	16	17
	<120	7	7	8	9	10	10	11	12	13	14	7	7	7	8	8	12	12	13	13	14
55-59	≥180	17	18	20	21	23	25	27	30	33	36	14	15	15	16	16	25	26	27	28	29
	160-179	12	13	15	16	17	19	20	22	25	27	11	11	12	12	13	19	20	21	22	23
	140-159	9	10	11	12	13	14	15	17	18	21	9	9	9	9	10	15	16	16	17	18
	120-139	7	7	8	9	9	10	11	12	14	15	7	7	7	7	7	12	12	13	13	14
	<120	5	5	6	6	7	7	8	9	10	11	5	5	5	5	6	9	9	10	10	11
50-54	≥180	13	14	16	17	19	21	23	25	28	31	12	12	12	13	13	21	22	23	24	25
	160-179	9	10	11	12	14	15	17	18	21	23	9	9	9	10	10	16	17	18	18	19
	140-159	7	7	8	9	10	11	12	13	15	17	6	7	7	7	7	12	13	13	14	15
	120-139	5	5	6	6	7	8	8	10	11	12	5	5	5	5	5	9	9	10	10	11
	<120	3	4	4	4	5	5	6	7	8	9	3	4	4	4	4	7	7	7	8	8
45-49	≥180	10	11	12	14	15	17	19	21	24	27	9	9	10	10	11	18	19	20	21	22
	160-179	7	8	9	10	11	12	13	15	17	20	7	7	7	7	8	13	14	15	15	16
	140-159	5	5	6	7	7	8	9	11	12	14	5	5	5	5	6	10	10	11	11	12
	120-139	3	4	4	5	5	6	6	7	8	10	3	4	4	4	4	7	7	8	8	9
	<120	2	2	3	3	3	4	4	5	6	7	2	3	3	3	3	5	5	6	6	6
40-44	≥180	8	9	10	11	12	14	16	18	21	24	7	8	8	8	8	16	16	17	18	19
	160-179	5	6	6	7	8	10	11	12	14	16	5	5	5	6	6	11	12	12	13	14
	140-159	3	4	4	5	6	6	7	8	10	11	4	4	4	4	4	8	8	9	9	10
	120-139	2	3	3	3	4	4	5	6	7	8	2	3	3	3	3	6	6	6	6	7
	<120	2	2	2	2	2	3	3	4	4	5	2	2	2	2	2	4	4	4	5	5

South Asia

Risk Level: <5% | 5% to <10% | 10% to <20% | 20% to <30% | ≥30%

Non-laboratory based risk chart

Age (years)	SBP (mmHg)	Men Non-smoker <20	20-24	25-29	30-35	≥ 35	Men Smoker <20	20-24	25-29	30-35	≥ 35	Women Non-smoker <20	20-24	25-29	30-35	≥ 35	Women Smoker <20	20-24	25-29	30-35	≥ 35
70-74	≥180	24	26	28	30	32	31	33	35	37	40	21	21	22	23	24	29	30	31	32	33
	160-179	20	22	23	25	27	26	28	30	32	34	17	18	19	19	20	25	26	26	27	28
	140-159	17	18	19	21	22	22	23	25	27	28	15	15	16	16	17	21	22	22	23	24
	120-139	14	15	16	17	18	18	19	21	22	24	12	13	13	14	14	18	18	19	20	20
	<120	11	12	13	14	15	15	16	17	18	20	10	11	11	12	12	15	15	16	17	17
65-69	≥180	19	20	22	24	26	26	28	30	33	36	16	17	18	18	19	25	26	27	28	29
	160-179	15	17	18	20	22	21	23	25	27	30	13	14	14	15	16	21	21	22	23	24
	140-159	12	14	15	16	18	17	19	21	22	25	11	11	12	12	13	17	18	19	19	20
	120-139	10	11	12	13	14	14	15	17	18	20	9	9	10	10	11	14	15	15	16	17
	<120	8	9	10	11	12	11	12	14	15	16	7	8	8	8	9	12	12	13	13	14
60-64	≥180	15	16	18	20	22	21	24	26	29	32	13	13	14	14	15	21	22	23	24	26
	160-179	12	13	14	16	18	17	19	21	24	26	10	11	11	12	12	17	18	19	20	21
	140-159	9	10	11	13	14	14	15	17	19	21	8	9	9	9	10	14	15	15	16	17
	120-139	7	8	9	10	11	11	12	14	15	17	7	7	7	8	8	11	12	12	13	14
	<120	6	6	7	8	9	9	10	11	12	13	5	5	6	6	6	9	9	10	11	11
55-59	≥180	11	13	14	16	18	18	20	23	26	29	10	10	11	11	12	18	19	20	21	22
	160-179	9	10	11	13	14	14	16	18	20	23	8	8	9	9	10	14	15	16	17	18
	140-159	7	8	9	10	11	11	12	14	16	18	6	6	7	7	7	11	12	13	13	14
	120-139	5	6	7	8	9	9	10	11	12	14	5	5	5	6	6	9	9	10	11	11
	<120	4	5	5	6	7	7	7	8	10	11	4	4	4	4	5	7	7	8	8	9
50-54	≥180	9	10	11	13	15	15	17	20	22	26	8	8	9	9	10	15	16	17	18	19
	160-179	7	8	9	10	11	11	13	15	17	20	6	6	7	7	7	12	13	13	14	15
	140-159	5	6	7	8	9	9	10	12	13	15	5	5	5	5	6	9	10	10	11	12
	120-139	4	4	5	6	7	7	8	9	10	12	3	4	4	4	4	7	7	8	9	9
	<120	3	3	4	4	5	5	6	7	8	9	3	3	3	3	3	5	6	6	7	7
45-49	≥180	7	8	9	11	12	12	14	17	20	23	6	6	7	7	8	13	14	15	16	17
	160-179	5	6	7	8	9	9	11	13	15	17	5	5	5	5	6	10	10	11	12	13
	140-159	4	4	5	6	7	7	8	9	11	13	3	4	4	4	4	7	8	8	9	10
	120-139	3	3	4	4	5	5	6	7	8	10	2	3	3	3	3	6	6	6	7	7
	<120	2	2	3	3	4	4	4	5	6	7	2	2	2	2	2	4	4	5	5	6
40-44	≥180	5	6	7	9	10	10	12	14	17	20	5	5	5	6	6	11	12	13	14	15
	160-179	4	5	5	6	7	8	9	11	13	15	3	4	4	4	4	8	9	9	10	11
	140-159	3	3	4	5	5	5	6	8	9	11	2	3	3	3	3	6	6	7	7	8
	120-139	2	2	3	3	4	4	5	6	7	8	2	2	2	2	2	4	5	5	6	6
	<120	2	2	2	2	3	3	3	4	5	6	1	1	1	2	2	3	3	4	4	4

Body mass index (kg/m^2)

Southeast Asia

Risk Level: <5% | 5% to <10% | 10% to <20% | 20% to <30% | ≥30%

Non-laboratory based risk chart

Age (years)	SBP (mmHg)	Men Non-smoker <20	20-24	25-29	30-35	≥ 35	Men Smoker <20	20-24	25-29	30-35	≥ 35	Women Non-smoker <20	20-24	25-29	30-35	≥ 35	Women Smoker <20	20-24	25-29	30-35	≥ 35
70-74	≥180	26	28	30	32	34	34	36	39	41	44	21	22	22	23	24	30	31	32	33	34
	160-179	21	23	25	26	28	28	30	32	34	36	17	18	19	19	20	25	26	27	28	29
	140-159	17	19	20	21	23	23	24	26	28	30	15	15	16	16	17	21	22	22	23	24
	120-139	14	15	16	17	18	18	20	21	23	24	12	12	13	13	14	17	18	19	19	20
	<120	11	12	13	14	15	15	16	17	18	20	10	10	11	11	11	14	15	15	16	17
65-69	≥180	20	22	24	26	28	28	30	33	36	39	16	17	18	18	19	25	26	27	29	30
	160-179	16	17	19	21	22	22	24	26	29	31	13	14	14	15	15	21	22	22	23	24
	140-159	12	14	15	16	18	18	19	21	23	25	11	11	11	12	12	17	18	18	19	20
	120-139	10	11	12	13	14	14	15	16	18	20	9	9	9	10	10	14	14	15	15	16
	<120	8	8	9	10	11	11	12	13	14	16	7	7	7	8	8	11	11	12	12	13
60-64	≥180	15	17	19	21	23	23	25	28	31	34	13	13	14	14	15	21	22	23	25	26
	160-179	12	13	14	16	18	18	20	22	24	27	10	10	11	11	12	17	18	19	20	21
	140-159	9	10	11	12	14	14	15	17	19	21	8	8	8	9	9	13	14	15	16	16
	120-139	7	8	8	9	10	10	12	13	14	16	6	6	7	7	7	11	11	12	12	13
	<120	5	6	6	7	8	8	9	10	11	12	5	5	5	5	6	8	9	9	10	10
55-59	≥180	12	13	15	16	18	19	21	24	27	30	10	10	11	11	12	18	19	20	21	22
	160-179	9	10	11	12	14	14	16	18	20	23	8	8	8	9	9	14	15	15	16	17
	140-159	7	7	8	9	10	11	12	13	15	17	6	6	6	7	7	11	11	12	13	13
	120-139	5	5	6	7	8	8	9	10	11	13	4	5	5	5	5	8	9	9	10	10
	<120	4	4	4	5	6	6	7	7	8	10	3	3	4	4	4	6	7	7	7	8
50-54	≥180	9	10	11	13	15	15	18	20	23	27	8	8	8	9	9	15	16	17	18	19
	160-179	7	7	8	9	11	11	13	15	17	20	6	6	6	7	7	11	12	13	14	14
	140-159	5	5	6	7	8	8	9	11	12	14	4	4	5	5	5	9	9	10	10	11
	120-139	3	4	4	5	6	6	7	8	9	11	3	3	3	4	4	6	7	7	8	8
	<120	2	3	3	4	4	4	5	6	7	8	2	2	3	3	3	5	5	5	6	6
45-49	≥180	7	8	9	10	12	13	15	17	20	23	6	6	7	7	7	13	14	14	15	16
	160-179	5	6	6	7	8	9	10	12	14	17	4	5	5	5	5	9	10	11	11	12
	140-159	3	4	4	5	6	6	7	9	10	12	3	3	3	4	4	7	7	8	8	9
	120-139	2	3	3	4	4	4	5	6	7	8	2	2	2	3	3	5	5	6	6	6
	<120	2	2	2	2	3	3	4	4	5	6	2	2	2	2	2	4	4	4	4	5
40-44	≥180	5	6	7	8	10	10	12	14	17	20	5	5	5	5	6	11	11	12	13	14
	160-179	4	4	5	6	7	7	8	10	12	14	3	3	4	4	4	8	8	9	9	10
	140-159	2	3	3	4	4	5	6	7	8	10	2	2	3	3	3	5	6	6	7	7
	120-139	2	2	2	3	3	3	4	5	6	7	2	2	2	2	2	4	4	4	5	5
	<120	1	1	2	2	2	2	3	3	4	5	1	1	1	1	1	3	3	3	3	4

Body mass index (kg/m^2)

Central Asia

Risk Level: <5% | 5% to <10% | 10% to <20% | 20% to <30% | ≥30%

Non-laboratory based risk chart

Age (years)	SBP (mmHg)	Men Non-smoker <20	Men Non-smoker 20-24	Men Non-smoker 25-29	Men Non-smoker 30-35	Men Non-smoker ≥ 35	Men Smoker <20	Men Smoker 20-24	Men Smoker 25-29	Men Smoker 30-35	Men Smoker ≥ 35	Women Non-smoker <20	Women Non-smoker 20-24	Women Non-smoker 25-29	Women Non-smoker 30-35	Women Non-smoker ≥ 35	Women Smoker <20	Women Smoker 20-24	Women Smoker 25-29	Women Smoker 30-35	Women Smoker ≥ 35
70-74	≥180	47	51	56	62	67	64	69	74	79	84	38	40	43	46	48	61	64	68	71	74
	160-179	37	41	45	50	55	52	57	62	67	73	30	32	34	36	38	50	53	56	59	62
	140-159	28	31	35	39	43	41	45	50	55	60	23	25	26	28	30	40	42	45	48	51
	120-139	22	24	27	30	33	32	35	39	44	48	18	19	20	22	23	31	33	35	38	40
	<120	16	18	20	23	25	24	27	30	34	38	14	15	16	17	18	24	26	27	29	31
65-69	≥180	33	37	42	48	54	51	58	64	71	77	26	28	30	32	35	50	53	57	60	64
	160-179	25	28	32	37	42	40	45	51	57	64	20	21	23	25	26	39	42	45	48	52
	140-159	18	21	24	27	32	30	34	39	45	51	15	16	17	18	20	30	32	34	37	40
	120-139	13	15	18	20	23	22	25	29	34	39	11	12	13	14	15	22	24	26	28	31
	<120	10	11	13	15	17	16	19	22	25	29	8	9	10	10	11	17	18	19	21	23
60-64	≥180	23	26	31	36	42	40	47	54	62	70	18	19	21	23	25	39	43	46	50	54
	160-179	16	19	22	26	31	30	35	41	48	55	13	14	15	17	18	29	32	35	38	42
	140-159	12	14	16	19	22	21	25	30	35	42	10	10	11	12	13	22	24	26	28	31
	120-139	8	10	11	13	16	15	18	21	26	30	7	8	8	9	9	16	17	19	21	23
	<120	6	7	8	10	11	11	13	15	18	22	5	6	6	6	7	11	12	14	15	16
55-59	≥180	16	19	22	27	32	31	37	44	53	62	13	14	15	16	17	31	34	37	41	45
	160-179	11	13	16	19	23	22	26	32	39	47	9	10	10	11	12	22	24	27	30	33
	140-159	8	9	11	13	16	15	18	23	28	34	6	7	7	8	9	16	17	19	21	24
	120-139	5	6	7	9	11	11	13	16	19	24	5	5	5	6	6	11	12	14	15	17
	<120	4	4	5	6	7	7	9	11	13	16	3	3	4	4	4	8	9	9	11	12
50-54	≥180	11	13	16	20	24	24	29	36	44	54	9	10	10	11	12	24	27	30	33	37
	160-179	8	9	11	13	17	16	20	25	31	39	6	7	7	8	8	17	19	21	23	26
	140-159	5	6	7	9	11	11	13	17	21	27	4	5	5	5	6	12	13	14	16	18
	120-139	4	4	5	6	7	7	9	11	14	18	3	3	3	4	4	8	9	10	11	12
	<120	2	3	3	4	5	5	6	7	9	12	2	2	2	2	3	5	6	7	7	8
45-49	≥180	8	9	12	14	18	18	23	29	37	47	7	7	7	8	9	19	21	23	26	30
	160-179	5	6	8	9	12	12	15	19	25	32	4	5	5	5	6	13	14	16	18	20
	140-159	4	4	5	6	8	8	10	13	16	21	3	3	3	4	4	8	9	11	12	14
	120-139	2	3	3	4	5	5	6	8	11	14	2	2	2	2	3	6	6	7	8	9
	<120	2	2	2	3	3	3	4	5	7	9	1	1	1	2	2	4	4	5	5	6
40-44	≥180	6	7	8	11	14	14	17	23	30	40	5	5	5	6	6	15	16	18	21	24
	160-179	4	4	5	7	9	9	11	15	20	26	3	3	4	4	4	10	11	12	14	16
	140-159	2	3	3	4	5	6	7	9	12	17	2	2	2	2	3	6	7	8	9	10
	120-139	2	2	2	3	3	4	5	6	8	11	1	1	1	2	2	4	4	5	6	6
	<120	1	1	1	2	2	2	3	4	5	7	1	1	1	1	1	3	3	3	4	4

Body mass index (kg/m^2)

High-income Asia Pacific

Risk Level: <5% | 5% to <10% | 10% to <20% | 20% to <30% | ≥30%

Non-laboratory based risk chart

Age (years)	SBP (mmHg)	Men Non-smoker					Men Smoker					Women Non-smoker					Women Smoker				
	BMI (kg/m^2)	<20	20-24	25-29	30-35	≥ 35	<20	20-24	25-29	30-35	≥ 35	<20	20-24	25-29	30-35	≥ 35	<20	20-24	25-29	30-35	≥ 35
70-74	≥180	23	24	25	27	28	28	29	31	33	34	19	20	21	22	23	28	30	31	32	34
	160-179	19	20	21	23	24	24	25	26	28	30	16	16	17	18	19	23	24	26	27	28
	140-159	16	17	18	19	20	20	21	23	24	25	13	13	14	15	15	19	20	21	22	23
	120-139	14	15	15	16	17	17	18	19	20	22	11	11	12	12	13	16	17	17	18	19
	<120	12	12	13	14	15	14	15	16	17	18	9	9	9	10	10	13	14	14	15	16
65-69	≥180	18	20	21	22	24	24	25	27	29	31	14	15	16	17	17	24	25	26	27	29
	160-179	15	16	17	19	20	20	21	23	25	26	12	12	13	13	14	19	20	21	22	23
	140-159	13	14	15	16	17	17	18	19	21	22	9	10	10	11	11	15	16	17	18	19
	120-139	10	11	12	13	14	14	15	16	17	18	7	8	8	9	9	12	13	14	14	15
	<120	9	9	10	11	12	11	12	13	14	15	6	6	6	7	7	10	10	11	11	12
60-64	≥180	15	16	17	19	20	20	22	24	26	28	11	12	12	13	13	20	21	22	23	25
	160-179	12	13	14	15	17	17	18	20	21	23	9	9	9	10	11	15	16	17	18	19
	140-159	10	11	12	13	14	14	15	16	18	19	7	7	7	8	8	12	13	14	14	15
	120-139	8	9	9	10	11	11	12	13	14	16	5	5	6	6	6	9	10	11	11	12
	<120	6	7	8	8	9	9	10	11	12	13	4	4	4	5	5	7	8	8	9	9
55-59	≥180	12	13	14	16	17	17	19	21	23	26	8	9	9	10	10	16	17	18	20	21
	160-179	10	10	11	13	14	14	15	17	19	21	6	7	7	7	8	12	13	14	15	16
	140-159	8	8	9	10	11	11	12	14	15	17	5	5	5	6	6	10	10	11	12	12
	120-139	6	7	7	8	9	9	10	11	12	13	4	4	4	4	5	7	8	8	9	9
	<120	5	5	6	6	7	7	8	9	10	11	3	3	3	3	3	6	6	6	7	7
50-54	≥180	10	11	12	13	15	15	17	18	21	23	6	7	7	8	8	14	14	15	17	18
	160-179	8	8	9	10	12	12	13	15	16	18	5	5	5	6	6	10	11	12	12	13
	140-159	6	7	7	8	9	9	10	11	13	15	4	4	4	4	4	8	8	9	9	10
	120-139	5	5	6	6	7	7	8	9	10	11	3	3	3	3	3	6	6	6	7	7
	<120	4	4	4	5	6	6	6	7	8	9	2	2	2	2	2	4	4	5	5	5
45-49	≥180	8	9	10	11	12	13	14	16	18	21	5	5	5	6	6	11	12	13	14	15
	160-179	6	7	7	8	10	10	11	12	14	16	4	4	4	4	4	8	9	9	10	11
	140-159	5	5	6	6	7	8	9	10	11	13	3	3	3	3	3	6	6	7	7	8
	120-139	4	4	4	5	6	6	7	7	8	10	2	2	2	2	2	4	5	5	5	6
	<120	3	3	3	4	4	4	5	6	7	7	1	1	1	2	2	3	3	4	4	4
40-44	≥180	6	7	8	9	10	11	12	14	16	19	4	4	4	5	5	9	10	11	12	13
	160-179	5	5	6	7	8	8	9	11	12	14	3	3	3	3	3	7	7	8	8	9
	140-159	4	4	5	5	6	6	7	8	9	11	2	2	2	2	2	5	5	5	6	6
	120-139	3	3	3	4	4	5	5	6	7	8	1	1	1	2	2	3	4	4	4	5
	<120	2	2	3	3	3	3	4	5	5	6	1	1	1	1	1	2	2	3	3	3

Body mass index (kg/m^2)

Western Europe

Risk Level: <5% | 5% to <10% | 10% to <20% | 20% to <30% | ≥30%

Non-laboratory based risk chart

Age (years)	SBP (mmHg)	Men Non-smoker <20	20-24	25-29	30-35	≥ 35	Men Smoker <20	20-24	25-29	30-35	≥ 35	Women Non-smoker <20	20-24	25-29	30-35	≥ 35	Women Smoker <20	20-24	25-29	30-35	≥ 35
70-74	≥180	30	32	34	36	39	38	41	43	46	49	24	25	26	28	29	38	40	42	44	46
	160-179	24	26	28	30	32	31	34	36	39	41	19	20	21	22	23	30	32	34	36	38
	140-159	20	21	23	25	27	26	28	30	32	35	15	15	16	17	18	24	25	27	28	30
	120-139	16	17	19	20	22	21	23	25	26	29	11	12	13	13	14	19	20	21	22	24
	<120	13	14	15	16	18	17	18	20	22	23	9	9	10	10	11	15	16	17	18	19
65-69	≥180	23	25	27	29	32	32	34	37	41	44	17	18	19	20	21	31	32	34	37	39
	160-179	18	20	22	24	26	25	28	30	33	36	13	13	14	15	16	24	25	27	29	31
	140-159	14	16	17	19	21	20	22	24	27	30	10	10	11	12	12	18	19	21	22	24
	120-139	11	12	14	15	16	16	18	20	22	24	7	8	8	9	9	14	15	16	17	18
	<120	9	10	11	12	13	13	14	16	17	19	5	6	6	7	7	10	11	12	13	14
60-64	≥180	17	19	21	24	26	26	29	32	36	39	12	12	13	14	15	24	26	28	30	32
	160-179	13	15	16	18	21	20	23	25	28	32	9	9	10	10	11	18	20	21	23	25
	140-159	10	11	13	14	16	16	18	20	22	25	6	7	7	8	8	13	15	16	17	18
	120-139	8	9	10	11	12	12	14	15	17	20	4	5	5	6	6	10	11	12	13	14
	<120	6	7	7	8	10	9	11	12	14	15	3	3	4	4	4	7	8	8	9	10
55-59	≥180	13	15	17	19	21	21	24	27	31	35	8	9	9	10	11	19	21	23	25	27
	160-179	10	11	12	14	16	16	18	21	24	27	6	6	7	7	8	14	15	16	18	20
	140-159	7	8	9	11	12	12	14	16	18	21	4	4	5	5	6	10	11	12	13	14
	120-139	5	6	7	8	9	9	11	12	14	16	3	3	3	4	4	7	8	8	9	10
	<120	4	5	5	6	7	7	8	9	11	12	2	2	2	3	3	5	5	6	7	7
50-54	≥180	10	11	13	15	17	17	20	23	27	31	6	6	7	7	8	15	17	18	20	22
	160-179	7	8	9	11	13	13	15	17	20	24	4	4	5	5	5	11	12	13	14	16
	140-159	5	6	7	8	9	9	11	13	15	18	3	3	3	3	4	7	8	9	10	11
	120-139	4	4	5	6	7	7	8	10	11	13	2	2	2	2	3	5	6	6	7	8
	<120	3	3	4	4	5	5	6	7	8	10	1	1	1	2	2	3	4	4	5	5
45-49	≥180	7	8	10	12	14	14	17	20	23	27	4	4	5	5	6	12	13	15	16	18
	160-179	5	6	7	8	10	10	12	14	17	20	3	3	3	3	4	8	9	10	11	12
	140-159	4	4	5	6	7	7	9	10	12	15	2	2	2	2	2	5	6	7	7	8
	120-139	2	3	4	4	5	5	6	7	9	11	1	1	1	1	2	4	4	4	5	6
	<120	2	2	3	3	4	4	4	5	7	8	1	1	1	1	1	2	3	3	3	4
40-44	≥180	5	6	8	9	11	11	14	16	20	24	3	3	3	4	4	9	10	12	13	15
	160-179	4	4	5	6	8	8	10	12	14	18	2	2	2	2	3	6	7	8	9	10
	140-159	2	3	4	4	5	6	7	8	10	13	1	1	1	1	2	4	4	5	6	6
	120-139	2	2	3	3	4	4	5	6	7	9	1	1	1	1	1	3	3	3	4	4
	<120	1	1	2	2	3	3	3	4	5	6	0	0	1	1	1	2	2	2	2	3

Body mass index (kg/m^2)

Central Europe

Risk Level: <5% | 5% to <10% | 10% to <20% | 20% to <30% | ≥30%

Non-laboratory based risk chart

Age (years)	SBP (mmHg)	Men Non-smoker					Men Smoker					Women Non-smoker					Women Smoker				
		<20	20-24	25-29	30-35	≥ 35	<20	20-24	25-29	30-35	≥ 35	<20	20-24	25-29	30-35	≥ 35	<20	20-24	25-29	30-35	≥ 35
70-74	≥180	35	38	41	44	47	46	49	52	56	59	33	35	36	38	40	52	55	57	60	62
	160-179	28	31	33	35	38	37	40	43	46	49	26	27	29	30	32	42	44	47	49	51
	140-159	23	24	26	28	31	30	32	35	38	41	20	21	22	23	25	33	35	37	39	41
	120-139	18	19	21	23	24	24	26	28	30	33	15	16	17	18	19	26	27	29	31	32
	<120	14	15	16	18	19	19	20	22	24	26	12	12	13	14	14	20	21	22	24	25
65-69	≥180	27	29	32	35	38	38	41	45	49	53	23	25	26	28	29	43	45	48	50	53
	160-179	21	23	25	27	30	30	33	36	39	43	17	18	19	21	22	33	35	37	39	42
	140-159	16	17	19	21	23	23	25	28	31	34	13	13	14	15	16	25	26	28	30	32
	120-139	12	13	15	16	18	18	20	22	24	27	9	10	10	11	12	18	20	21	22	24
	<120	9	10	11	12	14	13	15	17	19	21	7	7	8	8	9	14	14	16	17	18
60-64	≥180	20	22	25	27	31	31	34	38	42	47	16	17	18	19	21	34	36	39	42	44
	160-179	15	16	18	21	23	23	26	29	33	37	11	12	13	14	15	25	27	29	31	33
	140-159	11	12	14	15	17	17	20	22	25	28	8	9	9	10	11	18	19	21	23	25
	120-139	8	9	10	11	13	13	15	17	19	21	6	6	6	7	7	13	14	15	16	18
	<120	6	7	7	8	10	10	11	12	14	16	4	4	5	5	5	9	10	11	12	13
55-59	≥180	15	16	19	21	24	25	28	32	36	41	11	12	13	14	15	27	29	31	34	37
	160-179	10	12	14	16	18	18	21	24	27	31	8	8	9	9	10	19	20	22	24	26
	140-159	7	8	10	11	13	13	15	17	20	23	5	5	6	6	7	13	14	15	17	19
	120-139	5	6	7	8	9	9	11	13	15	17	3	4	4	4	5	9	10	11	12	13
	<120	4	4	5	6	7	7	8	9	11	12	2	2	3	3	3	6	7	7	8	9
50-54	≥180	11	12	14	16	19	20	23	27	31	36	8	8	9	10	10	21	23	25	27	30
	160-179	7	9	10	12	14	14	16	19	22	27	5	5	6	6	7	14	15	17	19	21
	140-159	5	6	7	8	10	10	11	14	16	19	3	3	4	4	4	9	10	11	13	14
	120-139	3	4	5	6	7	7	8	10	11	14	2	2	2	3	3	6	7	7	8	9
	<120	2	3	3	4	5	5	6	7	8	10	1	1	2	2	2	4	4	5	5	6
45-49	≥180	8	9	11	13	15	16	19	22	26	31	5	6	6	7	7	17	18	20	22	24
	160-179	5	6	7	9	10	11	13	15	18	22	3	4	4	4	5	11	12	13	14	16
	140-159	3	4	5	6	7	7	9	11	13	16	2	2	2	3	3	7	7	8	9	10
	120-139	2	3	3	4	5	5	6	7	9	11	1	1	1	2	2	4	5	5	6	7
	<120	2	2	2	3	3	3	4	5	6	7	1	1	1	1	1	3	3	3	4	4
40-44	≥180	6	7	8	10	12	12	15	18	22	27	4	4	4	5	5	13	14	16	17	19
	160-179	4	4	5	6	8	8	10	12	15	19	2	2	3	3	3	8	9	10	11	12
	140-159	2	3	3	4	5	5	7	8	10	13	1	1	2	2	2	5	5	6	7	8
	120-139	2	2	2	3	3	4	4	5	7	9	1	1	1	1	1	3	3	4	4	5
	<120	1	1	1	2	2	2	3	4	4	6	0	0	1	1	1	2	2	2	3	3

Body mass index (kg/m^2)

Eastern Europe

Risk Level: <5% | 5% to <10% | 10% to <20% | 20% to <30% | ≥30%

Non-laboratory based risk chart

Age (years)	SBP (mmHg)	Men Non-smoker BMI <20	20-24	25-29	30-35	≥ 35	Men Smoker BMI <20	20-24	25-29	30-35	≥ 35	Women Non-smoker BMI <20	20-24	25-29	30-35	≥ 35	Women Smoker BMI <20	20-24	25-29	30-35	≥ 35
70-74	≥180	38	40	42	44	46	46	48	51	53	56	37	38	40	42	43	53	55	57	59	62
70-74	160-179	32	34	36	38	40	39	41	44	46	49	30	32	33	34	36	45	47	49	51	53
70-74	140-159	27	29	30	32	34	33	35	37	40	42	25	26	27	28	29	37	39	41	42	44
70-74	120-139	23	24	25	27	29	28	30	32	34	36	20	21	22	23	24	31	32	33	35	37
70-74	<120	19	20	21	23	24	24	25	27	29	30	16	17	18	18	19	25	26	27	29	30
65-69	≥180	30	33	35	37	40	40	42	45	48	51	28	29	30	32	34	45	47	49	52	54
65-69	160-179	25	27	29	31	33	33	35	38	41	44	22	23	24	26	27	37	39	40	43	45
65-69	140-159	21	22	24	26	28	27	29	32	34	37	17	18	19	20	21	29	31	33	35	36
65-69	120-139	17	18	20	21	23	23	24	26	28	31	14	14	15	16	17	23	25	26	28	29
65-69	<120	14	15	16	17	19	18	20	22	23	25	11	11	12	12	13	18	20	21	22	23
60-64	≥180	24	26	29	31	34	34	37	40	43	47	21	22	23	24	26	38	40	42	44	47
60-64	160-179	19	21	23	25	28	28	30	33	36	39	16	17	18	19	20	30	31	33	35	38
60-64	140-159	15	17	19	20	22	22	24	27	29	32	12	13	13	14	15	23	24	26	28	30
60-64	120-139	12	14	15	16	18	18	20	22	24	26	9	10	10	11	12	18	19	20	22	23
60-64	<120	10	11	12	13	14	14	16	17	19	21	7	7	8	8	9	14	14	15	17	18
55-59	≥180	19	21	23	26	29	29	32	35	38	42	15	16	17	18	19	31	33	35	38	40
55-59	160-179	15	17	18	20	23	23	25	28	31	34	11	12	13	14	15	24	25	27	29	31
55-59	140-159	12	13	14	16	18	18	20	22	25	28	8	9	9	10	11	18	19	21	22	24
55-59	120-139	9	10	11	13	14	14	16	18	20	22	6	7	7	7	8	13	14	15	17	18
55-59	<120	7	8	9	10	11	11	12	14	16	18	4	5	5	5	6	10	11	11	12	13
50-54	≥180	15	17	19	21	24	24	27	31	34	38	11	12	13	14	15	26	28	30	32	34
50-54	160-179	11	13	15	16	19	19	21	24	27	31	8	9	9	10	11	19	20	22	24	26
50-54	140-159	9	10	11	13	14	14	16	19	21	24	6	6	7	7	8	14	15	16	18	19
50-54	120-139	7	7	8	10	11	11	13	14	16	19	4	4	5	5	5	10	11	12	13	14
50-54	<120	5	6	6	7	8	8	10	11	13	15	3	3	3	4	4	7	8	8	9	10
45-49	≥180	12	13	15	17	20	20	23	27	30	35	8	9	10	10	11	21	23	25	27	29
45-49	160-179	9	10	11	13	15	15	18	20	23	27	6	6	7	7	8	15	16	18	19	21
45-49	140-159	6	7	9	10	11	12	13	15	18	21	4	4	5	5	5	11	12	13	14	15
45-49	120-139	5	5	6	7	9	9	10	12	14	16	3	3	3	3	4	7	8	9	10	11
45-49	<120	3	4	5	5	6	6	8	9	10	12	2	2	2	2	3	5	6	6	7	8
40-44	≥180	9	11	12	14	17	17	20	23	27	31	6	7	7	8	8	17	19	20	22	25
40-44	160-179	7	8	9	10	12	13	15	17	20	24	4	5	5	5	6	12	13	14	16	17
40-44	140-159	5	6	6	8	9	9	11	13	15	18	3	3	3	3	4	8	9	10	11	12
40-44	120-139	3	4	5	6	7	7	8	9	11	13	2	2	2	2	3	5	6	7	7	8
40-44	<120	2	3	3	4	5	5	6	7	8	10	1	1	1	2	2	4	4	5	5	6

Body mass index (kg/m^2)

Oceania

Risk Level: <5% | 5% to <10% | 10% to <20% | 20% to <30% | ≥30%

Non-laboratory based risk chart

Age (years)	SBP (mmHg)	Men Non-smoker <20	Men Non-smoker 20-24	Men Non-smoker 25-29	Men Non-smoker 30-35	Men Non-smoker ≥ 35	Men Smoker <20	Men Smoker 20-24	Men Smoker 25-29	Men Smoker 30-35	Men Smoker ≥ 35	Women Non-smoker <20	Women Non-smoker 20-24	Women Non-smoker 25-29	Women Non-smoker 30-35	Women Non-smoker ≥ 35	Women Smoker <20	Women Smoker 20-24	Women Smoker 25-29	Women Smoker 30-35	Women Smoker ≥ 35
70-74	≥180	26	27	29	30	32	32	33	35	37	38	22	23	23	24	25	30	31	32	32	33
	160-179	22	23	24	25	27	27	28	30	31	33	19	20	20	21	21	26	26	27	28	29
	140-159	18	19	20	21	23	22	24	25	26	28	16	17	17	18	18	22	23	23	24	25
	120-139	15	16	17	18	19	19	20	21	22	23	14	14	14	15	15	19	19	20	20	21
	<120	13	13	14	15	16	16	17	18	19	20	12	12	12	13	13	16	16	17	17	18
65-69	≥180	21	22	24	25	27	27	29	31	33	35	18	19	19	20	20	26	27	28	29	30
	160-179	17	18	19	21	22	22	24	25	27	29	15	15	16	16	17	22	23	23	24	25
	140-159	14	15	16	17	18	18	20	21	22	24	12	13	13	14	14	18	19	19	20	21
	120-139	11	12	13	14	15	15	16	17	18	20	10	11	11	11	12	15	16	16	17	17
	<120	9	10	11	11	12	12	13	14	15	16	8	9	9	9	10	13	13	13	14	14
60-64	≥180	17	18	19	21	23	23	25	27	29	31	15	15	15	16	17	23	23	24	25	26
	160-179	13	14	16	17	18	19	20	22	23	25	12	12	13	13	13	18	19	20	21	21
	140-159	11	12	12	14	15	15	16	17	19	21	10	10	10	10	11	15	16	16	17	17
	120-139	8	9	10	11	12	12	13	14	15	17	8	8	8	8	9	12	13	13	14	14
	<120	7	7	8	9	9	9	10	11	12	13	6	6	7	7	7	10	10	11	11	12
55-59	≥180	14	15	16	17	19	20	21	23	26	28	12	12	13	13	13	20	20	21	22	23
	160-179	11	11	12	14	15	15	17	18	20	22	9	10	10	10	11	16	16	17	18	18
	140-159	8	9	10	11	12	12	13	15	16	18	7	8	8	8	8	12	13	13	14	15
	120-139	6	7	8	8	9	9	10	11	13	14	6	6	6	6	7	10	10	11	11	12
	<120	5	5	6	6	7	7	8	9	10	11	4	5	5	5	5	8	8	8	9	9
50-54	≥180	11	12	13	14	16	17	18	20	23	25	9	10	10	11	11	17	18	18	19	20
	160-179	8	9	10	11	12	13	14	16	17	19	7	8	8	8	8	13	14	14	15	16
	140-159	6	7	8	8	9	10	11	12	13	15	6	6	6	6	6	10	11	11	12	12
	120-139	5	5	6	6	7	7	8	9	10	12	4	4	5	5	5	8	8	9	9	10
	<120	4	4	4	5	5	6	6	7	8	9	3	3	3	4	4	6	6	7	7	7
45-49	≥180	9	10	11	12	13	14	16	18	20	22	8	8	8	9	9	15	15	16	17	18
	160-179	6	7	8	9	10	11	12	13	15	17	6	6	6	6	7	11	12	12	13	13
	140-159	5	5	6	7	7	8	9	10	11	13	4	4	5	5	5	8	9	9	10	10
	120-139	3	4	4	5	6	6	7	7	8	10	3	3	3	4	4	6	7	7	7	8
	<120	3	3	3	4	4	4	5	6	6	7	2	2	3	3	3	5	5	5	6	6
40-44	≥180	7	8	9	10	11	12	13	15	17	20	6	6	7	7	7	13	13	14	15	15
	160-179	5	6	6	7	8	9	10	11	13	15	4	5	5	5	5	9	10	10	11	12
	140-159	4	4	5	5	6	6	7	8	9	11	3	3	4	4	4	7	7	8	8	9
	120-139	3	3	3	4	4	5	5	6	7	8	2	2	3	3	3	5	5	6	6	6
	<120	2	2	2	3	3	3	4	4	5	6	2	2	2	2	2	4	4	4	4	5

Body mass index (kg/m^2)

Australasia

Risk Level: <5% | 5% to <10% | 10% to <20% | 20% to <30% | ≥30%

Non-laboratory based risk chart

Age (years)	SBP (mmHg)	Men Non-smoker					Men Smoker					Women Non-smoker					Women Smoker				
	Body mass index (kg/m^2)	<20	20-24	25-29	30-35	≥ 35	<20	20-24	25-29	30-35	≥ 35	<20	20-24	25-29	30-35	≥ 35	<20	20-24	25-29	30-35	≥ 35
70-74	≥180	28	30	32	34	36	35	37	39	42	44	21	21	22	23	25	32	33	34	36	37
	160-179	23	25	27	28	30	29	31	33	35	38	16	17	18	19	20	26	27	28	29	31
	140-159	19	21	22	24	25	25	26	28	30	32	13	14	14	15	16	21	22	23	24	25
	120-139	16	17	18	20	21	20	22	23	25	27	10	11	11	12	13	17	17	18	19	20
	<120	13	14	15	16	17	17	18	20	21	22	8	9	9	9	10	13	14	14	15	16
65-69	≥180	22	24	26	28	30	30	32	34	37	40	15	16	17	17	18	26	27	29	30	32
	160-179	18	19	21	23	25	24	26	28	31	33	12	12	13	14	14	20	21	23	24	25
	140-159	14	16	17	19	20	20	22	23	25	28	9	9	10	10	11	16	17	18	19	20
	120-139	12	13	14	15	16	16	18	19	21	23	7	7	8	8	8	12	13	14	15	15
	<120	9	10	11	12	13	13	14	16	17	19	5	5	6	6	7	9	10	11	11	12
60-64	≥180	17	19	21	23	25	25	27	30	33	36	11	11	12	13	14	21	22	24	25	27
	160-179	13	15	16	18	20	20	22	24	27	29	8	9	9	10	10	16	17	18	19	21
	140-159	11	12	13	14	16	16	18	19	21	24	6	6	7	7	8	12	13	14	15	16
	120-139	8	9	10	12	13	13	14	16	17	19	4	5	5	5	6	9	10	10	11	12
	<120	7	7	8	9	10	10	11	12	14	15	3	3	4	4	4	7	7	8	8	9
55-59	≥180	13	15	17	19	21	21	23	26	29	32	8	8	9	9	10	17	18	20	21	22
	160-179	10	11	13	14	16	16	18	20	23	26	6	6	6	7	7	13	13	14	16	17
	140-159	8	9	10	11	13	13	14	16	18	20	4	4	5	5	5	9	10	11	11	12
	120-139	6	7	8	9	10	10	11	13	14	16	3	3	3	4	4	7	7	8	8	9
	<120	5	5	6	7	8	8	9	10	11	13	2	2	2	3	3	5	5	6	6	7
50-54	≥180	10	11	13	15	17	17	20	22	25	29	6	6	6	7	7	14	15	16	17	19
	160-179	8	9	10	11	13	13	15	17	20	23	4	4	4	5	5	10	11	11	12	14
	140-159	6	7	8	9	10	10	12	13	15	18	3	3	3	3	4	7	7	8	9	10
	120-139	4	5	6	7	8	8	9	10	12	14	2	2	2	2	3	5	5	6	6	7
	<120	3	4	4	5	6	6	7	8	9	11	1	1	2	2	2	3	4	4	5	5
45-49	≥180	8	9	10	12	14	14	17	19	22	26	4	4	5	5	5	11	12	13	14	16
	160-179	6	7	8	9	11	11	12	15	17	20	3	3	3	3	4	8	8	9	10	11
	140-159	4	5	6	7	8	8	9	11	13	15	2	2	2	2	2	5	6	6	7	8
	120-139	3	4	4	5	6	6	7	8	10	12	1	1	1	2	2	4	4	4	5	5
	<120	2	3	3	4	5	4	5	6	7	9	1	1	1	1	1	2	3	3	3	4
40-44	≥180	6	7	8	10	12	12	14	16	19	23	3	3	3	4	4	9	10	11	12	13
	160-179	4	5	6	7	9	9	10	12	15	17	2	2	2	2	3	6	6	7	8	9
	140-159	3	4	4	5	6	6	7	9	11	13	1	1	1	2	2	4	4	5	5	6
	120-139	2	3	3	4	5	5	6	7	8	10	1	1	1	1	1	3	3	3	4	4
	<120	2	2	2	3	3	3	4	5	6	7	1	1	1	1	1	2	2	2	2	3

Body mass index (kg/m^2)
